# Skin microbiome correlates with bioclimate and *Batrachochytrium dendrobatidis* infection intensity in Brazil’s Atlantic Forest treefrogs

**DOI:** 10.1038/s41598-020-79130-3

**Published:** 2020-12-18

**Authors:** Katharina Ruthsatz, Mariana L. Lyra, Carolina Lambertini, Anat M. Belasen, Thomas S. Jenkinson, Domingos da Silva Leite, C. Guilherme Becker, Célio F. B. Haddad, Timothy Y. James, Kelly R. Zamudio, Luís Felipe Toledo, Miguel Vences

**Affiliations:** 1 Institute of Zoology, Universität Hamburg, Martin-Luther-King-Platz 3, 20146 Hamburg, Germany; 2 Laboratório de Herpetologia, Depto de Biodiversidade, Instituto de Biociências and Centro de Aquicultura (CAUNESP), Universidade Estadual Paulista - UNESP, Rio Claro, São Paulo Brazil; 3 Laboratório de História Natural de Anfíbios Brasileiros (LaHNAB), Departamento de Biologia Animal, Instituto de Biologia, Universidade Estadual de Campinas, Campinas, São Paulo 13083-862 Brazil; 4 Department of Ecology and Evolutionary Biology, Cornell University, Ithaca, NY 14853-2701 USA; 5 Department of Wildlife, Fish and Conservation Biology, University of California, Davis, Davis, CA USA; 6 Laboratório de Antígenos Bacterianos II, Departamento de Genética, Evolução, Microbiologia e Imunologia, Instituto de Biologia, Universidade Estadual de Campinas, Caixa Postal 6109, Campinas, São Paulo CEP 13083‐862 Brazil; 7 Department of Biological Sciences, The University of Alabama, Tuscaloosa, AL 35847 USA; 8 Department of Ecology and Evolutionary Biology, University of Michigan, Ann Arbor, MI 48109 USA; 9 Zoological Institute, Technische Universität Braunschweig, Mendelssohnstraße 4, 38106 Brunswick, Germany

**Keywords:** Ecology, Microbiology, Molecular biology, Zoology

## Abstract

In Brazil’s Atlantic Forest (AF) biodiversity conservation is of key importance since the fungal pathogen *Batrachochytrium dendrobatidis* (Bd) has led to the rapid loss of amphibian populations here and worldwide. The impact of Bd on amphibians is determined by the host's immune system, of which the skin microbiome is a critical component. The richness and diversity of such cutaneous bacterial communities are known to be shaped by abiotic factors which thus may indirectly modulate host susceptibility to Bd*.* This study aimed to contribute to understanding the environment-host–pathogen interaction determining skin bacterial communities in 819 treefrogs (Anura: Hylidae and Phyllomedusidae) from 71 species sampled across the AF. We investigated whether abiotic factors influence the bacterial community richness and structure on the amphibian skin. We further tested for an association between skin bacterial community structure and Bd co-occurrence. Our data revealed that temperature, precipitation, and elevation consistently correlate with richness and diversity of the skin microbiome and also predict Bd infection status. Surprisingly, our data suggest a weak but significant positive correlation of Bd infection intensity and bacterial richness. We highlight the prospect of future experimental studies on the impact of changing environmental conditions associated with global change on environment-host–pathogen interactions in the AF.

## Introduction

Animals serve as hosts to highly variable and complex communities of microorganisms^1,2^, which play an important role in many processes including development, reproduction, digestion, and immune system function^3–5^. The microbial communities associated with the skin (i.e., skin or cutaneous microbiome) play a significant role in pathogen defense and may, therefore be involved in predisposition to different diseases and contribute to host fitness^6–8^. Due to its direct exposure to the environment, the skin microbiome is thought to be much more dynamic than the microbiome of other parts of the animal such as the gut^9,10^, and consequently, the role of the environment is expected to be strong in shaping its composition, diversity and function^11,12^. Moreover, host-specific factors such pH, sebum production, temperature and moisture are known to shape the skin microbiome and its defensive function^7,13,14^.

Amphibian skin facilitates respiration, water and temperature regulation, excretion, reproduction, anti-predator defense, and immune responses^15–17^ and hosts one of the best-studied animal-associated microbiomes^8^. Both environmental and host-specific factors are predictors of amphibian skin microbiome structure^18,19^. Among the environmental factors influencing the cutaneous bacterial communities of amphibians, life stage transitions^18,20,21^, diet composition^22^, disease^23^, elevation^24^, microbial interactions^25,26^, season^17,27^, temperature^14^, water pH^28^, contaminants^28^, captivity^29,30^, habitat fragmentation^31,32^, trophic network interactions^33^, host assemblages^34^, and precipitation have been identified^35^. Host associated factors are less studied but clearly also play a role because multiple studies have demonstrated that the skin microbiomes of co-occurring species are significantly different^18,29,36,37^. Only few general patterns have been identified for host-associated factors, whereas global-scale patterns in microbial diversity have been tied to environmental factors such as bioclimate and habitat^10,19^. Amphibians depend on microbial environmental reservoirs, such as forest soil, to maintain diverse skin microbiomes^30,38^ and the peculiar conditions of the amphibian skin selects for particular microbial taxa to be integrated in the skin-associated communities^37,39^. Consequently, it is thought that host and environmental factors interact closely to shape the amphibian skin microbiome^2^, but the underlying processes by which this selection occurs are not yet well understood^8^.

**Figure 1:**
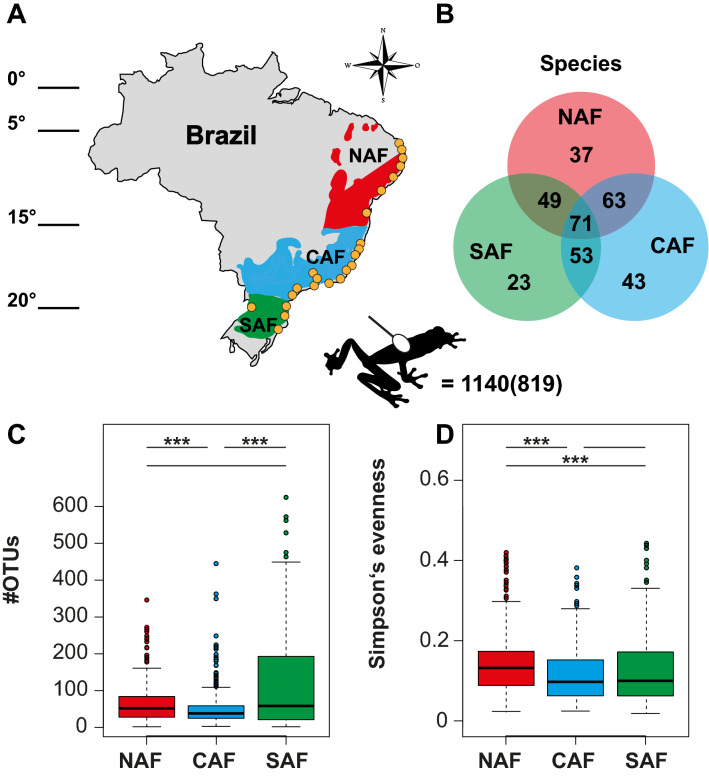
(**A**) Map of the three climatic regions of the Atlantic Forest (sensu^45^). We sampled skin microbiomes of 1140 treefrogs (Anura: Hylidae and Phyllomedusidae) from (**B**) 71 species over a latitudinal transect at the Atlantic Forest from 23 localities (i.e. orange dots) to test for differences in the amphibian skin bacterial community. 819 treefrogs remained in analysis after rarefaction (**C**) Number of operational taxonomic units (OTUs) were calculated as a measure of amphibian skin bacterial richness. (**D**) Simpson’s evenness was calculated as a measure of amphibian skin bacterial abundance. Differences between latitudinal groups were analyzed using a Kruskal–Wallis test, followed by Dunn’s post-hoc tests with Bonferroni correction. The map (**A**) was created with Adobe Illustrator 2021. NAF = North Atlantic Forest. CAF = Central Atlantic Forest. SAF = South Atlantic Forest.

The Brazilian Atlantic forest is home to more than 600 amphibian species, of which more than 80% are endemic^40–43^. This biome covers a large latitudinal range along Brazil’s 4000 km coast from the Equator to 30°S, and consequently experiences highly heterogeneous environmental conditions^44,45^. The Atlantic Forest can be subdivided into three main areas: North Atlantic Forest (NAF) between 5° and 15°S, Central Atlantic Forest (CAF) between 15° and 23°S, and South Atlantic Forest (SAF) from 23° to 30°S^45^ (Fig. 1). These geographical areas, combined with a wide latitudinal and altitudinal range^46^ expose wildlife to a multitude of abiotic factors which in turn may also shape their associated microbial communities. For amphibians, it can therefore be expected that skin microbiomes might differ across the Atlantic Forest and thus, might be more similar in cohabiting species within an area.

The Atlantic Forest is also one of the most threatened tropical ecosystems in the world^47^. Deforestation, habitat fragmentation, invasive species, and emergent infectious diseases^44,48–51^ contribute to observed amphibian declines in the Atlantic Forest^42,52^. A major threat to amphibian populations worldwide and also in the Atlantic Forest^51,53–62^ is the fungal pathogen *Batrachochytrium dendrobatidis* (Bd) which infects keratinized epithelial cells of amphibian skin^63–65^ and causes the disease chytridiomycosis. The emergence of Bd has led to the rapid loss of amphibian populations and entire species worldwide^66,67^, including the Atlantic Forest^51^. Consequently, investigating Bd presence and prevalence in the Atlantic Forest is critical for sustainable conservation actions.

Skin microbiomes constitute a critical component of the amphibian immune system^30,68^ because certain bacterial taxa are capable of producing anti-fungal metabolites that can suppress growth of Bd^33,69–71^ and, more generally, some bacterial taxa compete with each other, which limits the ability of multiple pathogens to infect^20^. Consequently, susceptibility to Bd might differ in amphibians across the Atlantic Forest if the skin bacterial microbiome is shaped differently due to heterogenous abiotic factors (reviews in^2,20,72–76^). Furthermore, Bd prevalence itself might be directly or indirectly affected by abiotic factors such as temperature, precipitation, elevation, and latitude (e.g.,^60,61,76^) that vary significantly among regions of the Atlantic Forest. Conversely, several studies (e.g.,^23,26,34,77^) have also demonstrated that Bd infection intensity can impact the skin bacterial microbiome, often but maybe not exclusively in the form of dysbiosis. Thus, invading pathogens can alter the structure of symbiotic microbial communities^2,23,73^ and might increase host susceptibility to pathogens, and in turn, microbial communities can protect their hosts from pathogens by direct defense mechanisms against the pathogen or increased host immune system capacity^31,69^. Teasing apart these complex interactions requires thorough experimental approaches but to inform the design of these, correlative studies from field settings are of high importance.

The influences of abiotic factors on animal- or plant associated bacterial communities are particularly obvious in the external microbiomes that are in direct contact with the environment^10^ In environmental (non host-associated) microbiomes, pH and salinity have been identified as important drivers of diversity^78,79^, and recent global-scale studies emphasize the importance of climate, with soils in tropical, warm and aseasonal environments hosting bacterial communities of lower richness (e.g.,^80^). Similar patterns have also been found for amphibian cutaneous microbiomes^19^, confirming that these depend on the pool of bacterial taxa recruitable from environmental reservoirs.

Here, we analyze the skin microbiome of 819 treefrogs (Anura: Hylidae and Phyllomedusidae) from 71 species over a latitudinal transect across the Atlantic Forest. We sampled frogs at 23 localities to test whether potential abiotic factors such as temperature, precipitation, seasonality, and latitude influence the bacterial community structures in the amphibian skin. We further tested for an association between microbial diversity and Bd infection which we expect if these abiotic factors are acting in concert with bacterial community structure in the amphibian skin to change disease outcome. Moreover, we assessed whether Bd infection status is associated with host effects (i.e. skin bacterial community and phylogeny) and abiotic factors. Our study is the first large-scale assessment of cutaneous amphibian microbiomes in the highly endangered Atlantic Coastal Forest of Brazil, thus contributing to a better understanding of how environmental factors shape microbial communities of amphibian hosts and environment-host–pathogen interactions.

## Material and methods

### Species sampling

We sampled 1140 frogs (family: Hylidae and Phyllomedusidae) from 23 sites along a latitudinal transect across the Atlantic Forest totaling 13 genera and 71 species (Table S1; Fig. 1). We focused on Hylidae and Phyllomedusidae, since treefrogs have a relatively smooth skin and due to their arboreal habits typically have fewer soil particles attached than many terrestrial frogs, thus increasing the likelihood that the identified bacteria are genuinely part of the cutaneous microbiome. Furthermore, Hylidae is one of the most abundant amphibian families in the Atlantic Forest^40,41^. Anurans were field sampled during the breeding season for each region during the years of 2012–2015. Anuran sampling permits were approved by the Instituto Chico Mendes de Conservação da Biodiversidade (SISBio #27745-13) and the Conselho de Gestão do Patrimônio Genético (SISGen #A1D66BF).

We recorded GPS coordinates in decimal degrees for each sampling location. In two cases where GPS coordinates were not collected, we used the geographic centroid of municipalities where the site was located for geographic analyses. Depending on coordinates, sites were assigned to three geographic groups following^45^: North Atlantic Forest (NAF), Central Atlantic Forest (CAF), and South Atlantic Forest (SAF; Fig. 1).

Each frog was captured using clean non-powdered gloves to control for transmission between animals and sample contamination. The frogs were held upside down and swabbed using a single sterile Medical Wire swab (MW113), targeting the ventral region and limbs according to the standardized procedure of Hyatt et al.^81,82^. Rinsing with distilled, filtered water as in other studies (e.g.,^8^) was not performed because it proved logistically unfeasible to provide all field teams with fresh rinsing water of comparable purity. After swabbing, frogs were immediately released at the site of capture. Skin swabs were placed individually in sterile 1.5 mL cryovials, and stored in standard freezer as soon as possible after swabbing. All samples were transported to the laboratory on ice and kept at -20 ºC until molecular analysis.

We extracted DNA from each swab using 50μL of PrepMan ULTRA (Life Technologies) per swab^72^, following a protocol optimized for *Bd* detection^82,83^. We agitated the tube with the solution for 1 min, boiled it for 10 min, and then cooled it for 2 min. We then centrifuged the tube at 12,000 rpm for 1 min, after which we inverted the swab with flame-sterilized tweezers before centrifuging the tube at 12,000 rpm for 5 min and then discarding the swab. We vortexed and centrifuged the tube for 1 min. We took 2 μl of extract and diluted 1:10 in nuclease free water for Bd infection analyses. We used 1 μl of non-diluted extract as template for bacterial community analyses.

### 16S rDNA sequencing

The V4 region of the 16S rRNA gene was amplified with barcoded primers (515f–806r) using a dual-index approach^84^ we followed the Earth Microbiome Project 16S Illumina Amplicon Protocol. PCR reactions were done in duplicates using conditions described in Bletz et al.^85^, but with Phire Hot Start II DNA Polymerase (Finnzyme, Espoo, Finland). PCR-conditions were: a denaturation step of 98 °C for 30 s, followed by 32 cycles at 98 °C for 5 s, 50 °C for 5 s, and 72 °C for 20 s, and a final extension at 72 °C for 5 min. Negative controls for each PCR mix were included. PCR products of each sample were combined and amplicons were visualized in 1% agarose gel to confirm consistent gel band strength, then samples were pooled in equal volumes to generate the amplicon library. We constructed four distinct libraries for all analyzed samples. Libraries were purified with the DNA Gel Extraction Kit (Norgen Biotek Corp, Thorold, ON, Canada) and final DNA concentration was determined on a Qubit fluorometer using a broad-range dsDNA kit. Each library was sequenced with paired-end 2 × 250 v2 chemistry on an Illumina MiSeq sequencer at TUCF Genomics, Boston, MA, USA.

### Bd infection load and infection presence

Molecular detection and quantification of Bd from extracted DNA was performed using the TaqMan qPCR Assay (Life Technologies)^82,83^). To generate the qPCR standard curve, we used the Atlantic Forest collected strain CLFT 023 as a quantitative standard, serially diluted from 10^3^ to 10^−1^ zoospore genomic equivalents (g.e.). We considered samples with at least one g.e. positive for Bd^86^. Bd infection intensity describes the count of zoopores measured on each sample. Bd infection status describes the individual infection state of a sample (i.e., an individual frog being Bd positive or negative).

### Abiotic variables

For each sampling location, we extracted 19 bioclimatic metrics for temperature and precipitation (BioClim, WorldClim) – Annual Mean Temperature (Bio1), Mean Diurnal Range (Bio 2), Isothermality (Bio 3), Temperature Seasonality (Bio 4), Maximum Temperature of Warmest Month (Bio 5), Minimum Temperature of Coldest Month (Bio 6), Annual Temperature Range (Bio 7), Mean Temperature of Wettest Quarter (Bio 8), Mean Temperature of Driest Quarter (Bio 9), Mean Temperature of Warmest Quarter (Bio 10), Mean Temperature of Coldest Quarter (Bio 11), Annual Precipitation (Bio 12), Precipitation of Wettest Month (Bio 13), Precipitation of Driest Month (Bio 14), Precipitation Seasonality (Bio 15), Precipitation of Wettest Quarter (Bio 16), Precipitation of Driest Quarter (Bio 17), Precipitation of Warmest Quarter (Bio 18), and Precipitation of Coldest Quarter (Bio 19) at a scale of 1 km^2^ for each metric^87^.

### Bacterial community and statistical analyses

Bacterial sequences were quality-filtered and analyzed in Quantitative Insights Into Microbial Ecology (MacQiime 1.9.1), unless otherwise noted, using only forward reads as reverse reads typically suffer from lower quality^88,89^. We used the deblur workflow^90^ for quality filtering (e.g. remove errors, noise and PCR chimeras) and clustering sequences in sub-operational taxonomic units (sOTUs; for simplicity, in the following referred to as OTUs), trimming reads to 150 bp and retaining only OTUs with more than 10 reads across dataset as recommend by Deblur developer team^90^. Taxonomy was assigned with the Ribosomal Database Project (RDP) Classifier using the Greengenes 13.8 reference database (May 2013 release; http://greengenes.lbl.gov/cgi-bin/nphindex.cgi). We discarded sequences identified as chloroplasts or mitochondria and singletons with fewer than 0.005% of total number of reads^91^. After filtering procedures, the dataset comprised 17,593,861 reads and an average of 21,508 reads per sample. To standardize read counts across samples for our main analyses, samples were subsequently rarefied at 1000 reads per sample (post filtering) and had an average of 72 OTUs. Samples with fewer than 1000 reads were removed, and a total of 819 samples were retained for subsequent analysis (Fig. S1). None of the PCR controls were retained after filtering and rarefaction. The most abundant sequence from each OTU was selected as a representative sequence and these representative sequences were aligned using PyNAST^92^. Number of observed OTUs (#OTUs) and Simpson’s evenness were calculated as alpha diversity metrices.

For all statistical tests R 4.0.2^93^ for Windows was used. All plots were constructed using ggplot2^94^ and Adobe Illustrator 2021.

#### Richness (alpha diversity) and compositional (beta diversity) analysis of amphibian skin bacterial communities

##### Alpha diversity

**Figure 2:**
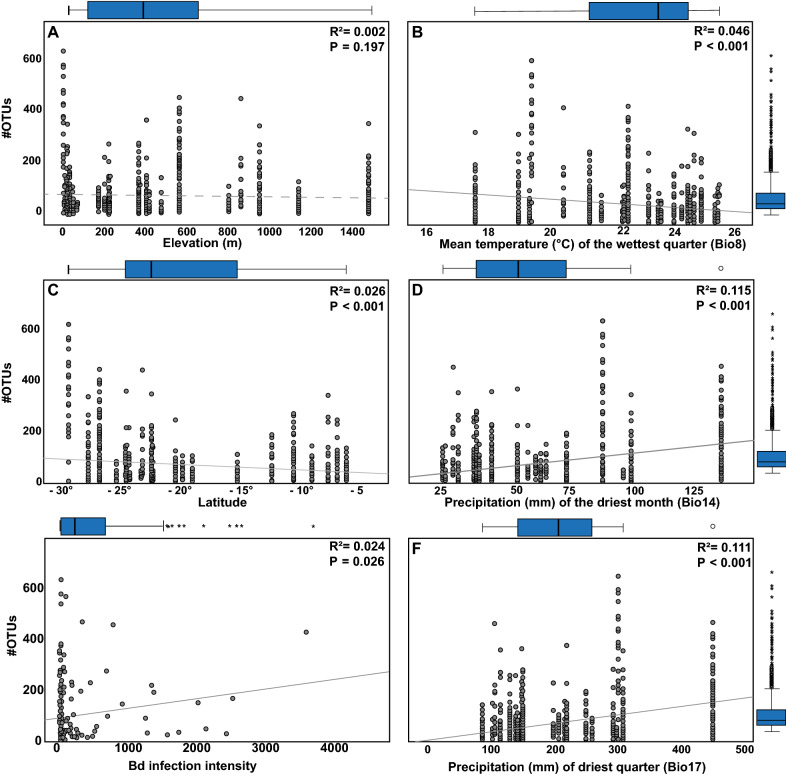
Richness (i.e. #OTUs) of treefrog (N = 819) skin bacterial communities is negatively correlated with (**B**) Mean Temperature (°C) of the Wettest Quarter (Bio8) and (**C**) latitude, but positively associated with (**D**) Precipitation (mm) of the Driest Month (Bio14), and (**F**) Precipitation (mm) of the Driest Quarter (Bio17) as well as (**E**) *Batrachochytrium dendrobatidis* (Bd) infection intensity (zero values and maximum extreme values were removed from analysis **E**). (**A**) Elevation as well as (**E**) *Batrachochytrium dendrobatidis* (Bd) infection intensity (N = 188; zero values were removed from analysis **E**) did not affect alpha diversity in treefrogs in the Atlantic Forest. Solid and dotted regression lines for significant and non-significant correlations, respectively. Error bar = median. Box = 1. and 3. quartiles. Dots = outliers, minimum and maximum values. Whiskers = 1.5-fold interquartile range.

To account for bacterial alpha diversity, we chose two variables that represent the two components of diversity: number of bacterial operational taxonomic units (#OTUs) per sample to represent richness, and Simpson's evenness to represent evenness across species. Initially, we performed single-variable correlations of latitude, elevation, Bd infection intensity, and bioclimatic measures with our two response variables, using simple linear regression models in R as an assessment of the strength of correlation of each predictor to the response of interest (Table S2, S3; Fig. 2).

**Figure 3:**
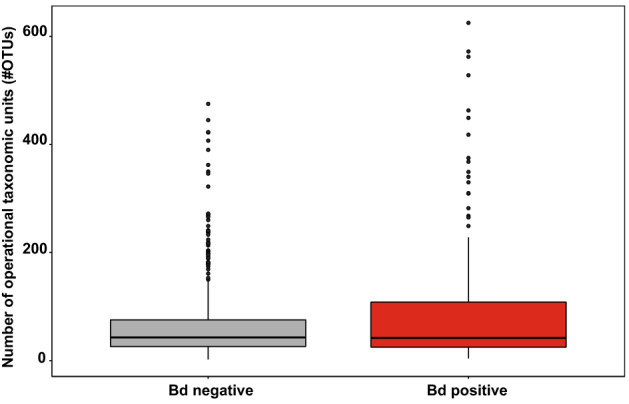
Boxplot of bacterial richness (#OTUs) was overall not different between Bd negative (grey, N = 603) and Bd positive (red, N = 188) treefrogs in the Brazilian Atlantic Forest (Mann–Whitney U-test, Chi^2^ = 0.754, df = 1, N = 791, *P* = 0.3851). Error bar = median. Box = 1. and 3. quartiles. Dots = outliers, minimum and maximum values. Whiskers = 1.5-fold interquartile range.

Differences in alpha diversity metrices between latitudinal groups were analyzed by Kruskal–Wallis test, followed by Dunn’s post-hoc tests with Bonferroni correction (Fig. 1). OTU richness between Bd negative and Bd positive treefrogs was compared by Mann–Whitney U test (Fig. 3).

*Response and predictor screening*: Response and Predictor Screening were performed in JMP 13.0 (SAS Institute) on filtered data set with #OTUs and Simpson’s evenness as response variables (Table S4–S7). Response Screening is a method to assess the strength of correlation of each predictor to the response of interest, whereas Predictor Screening is a method that uses bootstrap forest partitioning to evaluate the contribution of predictors on the response. The resulting partition models are built on multiple predictors. Predictor Screening can identify predictors that might be weak alone, but strong when used in combination with other predictors. In our analyses, all 19 bioclimatic variables were analyzed simultaneously, along with three additional non-categorical predictors (elevation, latitude, and Bd infection intensity).

*Model selection procedures*: Because many of the 19 bioclimatic variables are highly correlated, we applied different strategies to obtain sets of least-correlated variables for our GLMMs objectively and based on informed hypotheses following the procedure of Kueneman et al.^19^. We tried to include variables that at the same time were strong predictors of the data, least-correlated, and potentially biologically informative (based on a priori assumptions). Four alternative strategies were pursued to select bioclimatic variables to be included in candidate models: 1 (Model 1 in Supplementary tables 4 and 6): The five bioclim variables with strongest effect sizes from response screening. 2 (Model 2 in Supplementary tables 5 and 7): The five least-correlated variables taking effect sizes from response screening into account, obtained by manually removing first those variables correlated with a coefficient > 0.7 with the variable with strongest response screening effect for #OTUs, and then iteratively removing variables with the highest correlation coefficients and keeping variables that (i) were *apriori* considered to be potentially biologically informative and (ii) that had high contributions in predictor screening. 3 (Model 3 in Supplementary table 8): The five least-correlated variables, removing all variables with a correlation of r > 0.55 using the caret package in R. 4 (Model 4 in Supplementary tables 9 and 10): A set of variables (bioclimatic and other non-categorical) obtained using a rigorous model selection procedure, as follows: From the set of 19 bioclimatic variables, we selected the 5 least-correlated ones [i.e., latitude (r = − 0.070), elevation (r = − 0.087); Bio8 (r = − 0.130); Bio9 (r = − 0.080); Bio10 (r = − 0.071), Bio17 (r = 0.106)] based on a threshold of r < 0.85 using the caret package in R, and selected the best-fit model with 5 predictors or less using the glmulti package^95^ (https://cran.r-project.org/package=glmulti)in R with the *lmer.glmulti* wrapper function, taking into account the random factor as specified below.

To select best-fit models, we added to Models 1–4 the non-bioclimatic, non-categorical predictors: ‘elevation’, ‘latitude’, and ‘Bd infection intensity’ as fixed factors, and added as nested random factor the categorical predictors ‘location, sampling date, and host species’. We performed Generalized Linear Mixed Models (GLMM) using the *glmer*() function^96^ in the lme4 package (https://cran.r-project.org/package=lme4) in R, with Poisson distribution to test whether selected bioclimatic variables from Model 1–4, elevation, latitude, and Bd infection intensity are associated with richness and abundance of skin bacterial communities in treefrogs (i.e. observed #OTUs and Simpson’s evenness; Supplementary Table 9 and 10). Models 1–4 were compared based on their AIC values, and the model with lowest AIC chosen as the preferred model for each measure of alpha diversity. We further calculated the variance inflation factor (VIF score) and eigenvalues for factors included into Model 1–4 to test for multicollinearity using the vif() function in the VIF package^97^ (https://cran.r-project.org/package=VIF) in R.

As a second step, we excluded Bd-negative samples (N = 603) and samples with unknown Bd status (N = 28) from the analysis to avoid biases due to differences in Bd prevalence to understand the combined effect of pathogen load and bioclimatic factors on the skin microbiome. We then repeated the entire analysis with this reduced data set on 188 Bd-positive samples (Supplementary tables 11–17).

##### Beta diversity

To assess beta diversity, we used the full data set, including samples from non-Bd-infected frogs, to benefit from a larger sample size. We applied nonmetric Multi-Dimensional Scaling (NMDS)^98^ based on the Bray–Curtis measure of dissimilarity^99^ in OTU relative abundances across samples, as a simple way to visualize the composition, abundance, and dominance of bacterial taxa in the community without making assumptions about their genetic relationships. To visualize differences in bacterial community composition (i.e., beta diversity), we performed non metric multidimensional scaling (NMDS) and plotted values for the first two NMDS axes with centroids using the *metaMDS*() function from the vegan package^100^ in R.

**Figure 4:**
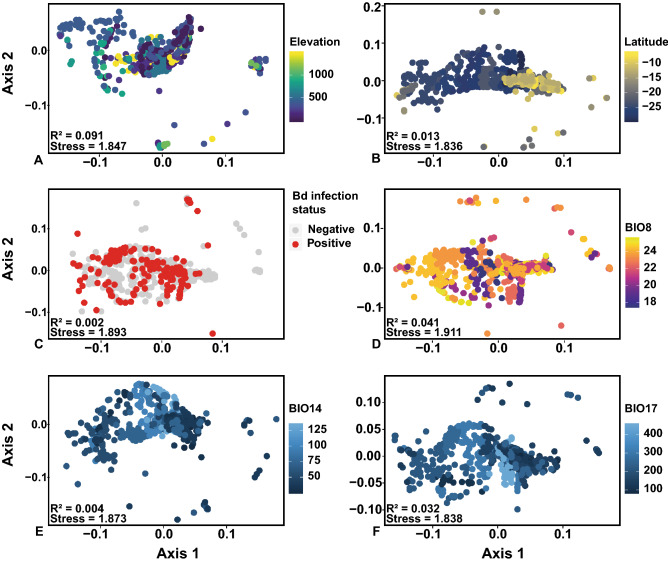
Beta diversity of amphibian skin bacterial communities is significantly structured by (**A**) elevation (m), (**B**) latitude, (**E**) Precipitation of the Wettest Month (mm, Bio14), and (**F**) Precipitation of the Driest Quarter (mm, Bio17), but not by (**C**) Bd infection status across the Brazilian Atlantic Forest and (**D**) Mean Temperature of the Wettest Quarter (°C, Bio8). Non- metric multidimensional scaling (NMDS) plot of Bray–Curtis distances. Each point represents the skin bacterial community of an individual treefrog, symbol color indicates variable gradient.

To test for the influence of latitude, elevation, Bd infection intensity, and bioclimatic variables on beta diversity, we performed permutation multivariate analysis of variance (PERMANOVA) on Bray–Curtis distances using the *adonis*() function^100^ in the vegan package (http://cran.r-project.org/package=vegan) with 9999 random permutations. Factors driving patterns in beta diversity were investigated with PERMANOVA estimating Pseudo-F and *P* values (Table 1; Fig. 4).

**Table 1:** Results of permutation multivariate analysis of variance (PERMANOVA) to examine the effect of selected predictor variables on beta diversity of amphibian skin microbiome in treefrogs of the Brazilian Atlantic Forest.

Predictor variable	Df	SumOfSqs	R^2^	F	Pr(> F)
Elevation	1	2.716	0.091	6.176	**< 0.001**
Latitude	1	2.125	0.013	7.114	**< 0.001**
Bd infection intensity	1	1.128	0.001	5.607	0.477
Bd infection status	1	1.415	0.002	4.582	0.341
Annual mean temperature (Bio1)	1	1.723	0.002	6.716	**< 0.001**
Mean diurnal range (Bio 2)	1	4.316	0.002	11.713	0.389
Isothermality (Bio 3)	1	2.116	0.012	4.005	0.431
Temperature seasonality (Bio 4)	1	1.846	0.026	6.147	**< 0.001**
Maximum temperature of warmest month (Bio 5)	1	1.291	0.004	5.136	0.221
Minimum temperature of coldest month (Bio 6)	1	0.876	0.031	2.637	**< 0.001**
Annual temperature range (Bio 7)	1	2.374	0.003	6.649	0.176
Mean temperature of wettest quarter (Bio 8)	1	2.003	0.041	7.301	0.053
Mean temperature of driest quarter (Bio 9)	1	0.978	0.008	0.916	0.061
Mean temperature of warmest quarter (Bio 10)	1	1.071	0.019	3.843	**< 0.001**
Mean temperature of coldest quarter (Bio 11)	1	1.024	0.028	4.178	0.054
Annual precipitation (Bio 12)	1	1.062	0.020	2.774	**< 0.001**
Precipitation of wettest month (Bio 13)	1	2.779	0.016	9.346	0.276
Precipitation of driest month (Bio 14)	1	1.129	0.004	3.713	**< 0.001**
Precipitation seasonality (Bio 15)	1	3.115	0.041	3.615	**< 0.001**
Precipitation of wettest quarter (Bio 16)	1	0.961	0.003	0.822	0.368
Precipitation of driest quarter (Bio 17)	1	5.611	0.032	13.211	**< 0.001**
Precipitation of warmest quarter (Bio 18)	1	0.995	0.008	1.773	0.731
Precipitation of coldest quarter (Bio 19)	1	1.001	0.021	2.843	0.428

PERMANOVAs on Bray–Curtis distances with 9999 random permutations.

### Host and environmental effects on Bd infection status and Bd infection intensity

We used Response Screening in JMP 13.0 (SAS Institute) on the full data set as an initial assessment of the strength of correlation of each of the 19 bioclimatic predictors plus elevation, latitude and number of bacterial OTUs to the response of interest (Bd infection intensity or Bd infection status; Supplementary tables 18 and 19).

GLMMs using the *glmer*() function^96^ in the lme4 package (https://cran.r-project.org/package=lme4) in R, with Poisson (for *Bd* infection intensity, Supplementary table S20) and binominal (for *Bd* infection status; Table 2) distribution were used to test the effect of the five selected bioclimatic variables from Response Screening in separated models. As further fixed factors we added elevation, latitude, and #OTUs independently from Response Screening results, due to their a priori assumed biological significance. We added as nested random factor the categorical predictors ‘location:sampling date: host species’.’ to correct for differences in microbiome and Bd susceptibility.

**Table 2:** Results of separate generalized mixed models (GLMMs) testing the effect of temperature seasonality (Bio4), minimum temperature of the coldest month (Bio6), mean temperature of the driest quarter (Bio9), mean temperature of the coldest quarter (Bio11), latitude, OTU richness (#OTUs), and elevation on Bd infection status in treefrogs in the Brazilian Atlantic Forest.

Bd infection status	GLMM, family = binomial		
Fixed factors	Intercept (SE)	Coefficient (SE)	*P*
Latitude	− 1.56 (0.17)	− 0.92 (0.19)	**< 0.001**
Elevation	− 1.63 (0.19)	0.48 (0.15)	**0.001**
#OTUs	− 1.82 (0.21)	0.00 (0.00)	**0.015**
BIO11	− 1.58 (0.17)	− 0.97 (0.17)	**< 0.001**
BIO9	− 1.59 (0.17)	− 0.93 (0.17)	**< 0.001**
BIO6	− 1.59 (0.17)	− 00.96 (0.17)	**< 0.001**
BIO4	− 1.49 (0.17)	0.91 (0.19)	**< 0.001**

For each fixed factor in the eight models, intercept, coefficients, standard error and the *P* value are shown. Bold *P* values indicate a significant effect of the fixed factor. Location:sampling date:host species was used as nested random factor. *P* values ware corrected for multiple-comparisons with Bonferroni correction. N = 791.

### Indicator species analysis

We used indicator species analysis using the *multipatt*() function in the indicspecies package^101^ (http://cran.r-project.org/package=indicspecies) to determine which OTUs are significantly (using 9999 permutations) associated with particular sample types, i.e., to Bd positive and Bd negative samples. Outcomes were then compared with the database of Woodhams et al.^68^ at family level to understand if Bd inhibitory or enhancing functions may be more common in infected versus non-infected treefrogs (Table S8). Since some OTUs may have close matches to both functions (Bd inhibitory and Bd enhancing), only distinct matches were used (Supplementary table S21).

### Statement of ethics

The authors have no ethical conflicts to disclose. Anuran sampling permits were approved by Instituto Chico Mendes de Conservação da Biodiversidade (SISBio #27745-13) Conselho de Gestão do Patrimônio Genético (SISGen #A1D66BF). The sampling was conducted under permission from animal care and use committee (Comissão de ética no uso de animais da Universidade Estadual de Campinas-CEUA/UNICAMP #4440-1). All methods were carried out in accordance with relevant guidelines and regulations.

## Results

### Richness of treefrog skin microbiomes weakly correlate with bioclimate but not with Bd infection intensity

The number of OTUs (#OTUs) in the cutaneous microbiomes of treefrogs sampled in the Atlantic Forest ranged from 2 to 625. Simpson’s evenness ranged from 0.016 to 0.636 (Supplementary table S1). We found differences among latitudinal groups in skin bacterial richness (i.e., #OTUs; Kruskal–Wallis test, Chi^2^ = 23.92, df = 2, N = 819, *P* < 0.001; Fig. 1C) and evenness (i.e., Simpson’s evenness, Chi^2^ = 26.63, df = 2, N = 819, *P* < 0.001; Fig. 1D). Treefrogs sampled in the SAF had the highest #OTUs, followed by those from the NAF, and the CAF. No difference in the #OTUs was found between Bd negative and Bd positive treefrogs (i.e., Mann–Whitney-U-test, Chi^2^ = 0.754, df = 1, N = 791, *P* = 0.3851; Fig. 3). Evenness was highest in treefrogs sampled in the NAF, followed by those from the SAF, and the CAF.

Richness (i.e. #OTUs) of treefrog skin bacterial community was determined by latitude, Mean Temperature of the Wettest Quarter (Bio8), Precipitation of the Driest Month (Bio14), and Precipitation of the Driest Quarter (Bio17; Table S2, S4; Fig. 2B–D,F). Bacterial richness increased with increasing precipitation of the driest month (Bio14) and of the driest quarter (Bio17), and decreased with increasing mean temperature of the wettest quarter (Bio8), and with increasing latitude (Fig. 2B–D,F). The Bd infection intensity was also correlated with OTU richness when upper extreme values and zeros were removed (Fig. 2E). Further, log-transformed Bd infection intensity was not correlated with OTU richness in the single-variable correlation (Fig. S5).

Evenness of treefrog skin bacterial community was determined by latitude, elevation, Minimum Temperature of Coldest Month (Bio 6), Annual Temperature Range (Bio 7), and Mean Temperature of Coldest Quarter (Bio 11; Table S3, S6). Bacterial evenness increased with increasing latitude and with (minimum) temperature of the coldest month and quarter (Bio 6 and 11), and decreased with elevation and annual temperature range (Bio 7). Further, log-transformed Bd infection intensity was not correlated with Simpson’s evenness in the single-variable correlation. The Bd infection intensity was correlated with Simpson’s evenness when zeros and upper extreme values were removed (Table S3).

We built generalized linear mixed models (GLMMs) for #OTUs (i.e., number of bacterial sub-operational taxonomic units) and Simpson’s evenness from a combination of biotic and abiotic factors including subsets of least-correlated bioclimatic predictors (Table S9, S10). The best GLMM for #OTUs (Model 1; Table S9) and for Simpson’s evenness (Model 4; Table S10) was selected based on lowest Akaike Information Criterion (AIC) value including maximum five bioclimatic variables, as well as elevation, latitude, and Bd infection intensity while accounting for location, sampling date, and host species as a nested random factor. We found that elevation, as well as the included bioclimatic variable Mean Temperature of Wettest Quarter (Bio 8), significantly explained the richness of treefrog (N = 819) skin bacterial communities (Table S9). Bacterial evenness could be explained by elevation as well as the included bioclimatic variables Mean Diurnal Range (Bio 2), Annual Temperature Range (Bio 7), and Mean Temperature of Coldest Quarter (Bio 11; Table S14).

From Response Screening, the highest coefficient value for #OTUs and Simpson’s evenness corresponded to Precipitation of the Driest Month (Bio14; Table S2) and Minimum Temperature of Coldest Month (Bio 6; Table S4), respectively. Partial effect analyses revealed that bacterial richness is positively related to Bio14 (Spearman’s r = 0.109; *P* = 0.002) and bacterial evenness is positively related to Bio 6 (Spearman’s r = 0.219; *P* < 0.001).

When zero values for Bd infection load were removed, the best GLMMs for #OTUs (Model 1; Table S14) and for Simpson’s evenness (Model 4; Table S17) were selected based on lowest Akaike Information Criterion (AIC) value including five bioclimatic variables, as well as elevation, latitude, and Bd infection intensity while accounting for location, sampling date, and host species as a nested random factor. From Response Screening, the highest coefficient value for #OTUs and Simpson’s evenness corresponded to Precipitation of the Driest Month (Bio14; Table S11) and Minimum Temperature of Coldest Month (Bio 6; Table S15), respectively. We found that, elevation, as well as the included bioclimatic variable Mean Temperature of Wettest Quarter (Bio 8) significantly explained the richness of treefrog (N = 188) skin bacterial communities (Table S11). For this data set, a single-variable analysis (Fig. 2E) revealed that Bd infection load was not correlated with bacterial richness. Bacterial evenness could not be explained by elevation, latitude, Bd infection intensity as well as the included bioclimatic variables (Table S17).

### Bioclimate explains relative abundances of bacterial taxa in the amphibian microbiome

Beta diversity of amphibian skin bacterial communities was significantly influenced by elevation, latitude, and some bioclimate variables (e.g. Annual Mean Temperature, Bio1; Temperature Seasonality, Bio4; Minimum Temperature of the Coldest Month, Bio6; Mean Temperature of the Warmest Quarter, Bio10; Annual Precipitation, Bio12; Precipitation of the Driest Month, Bio14; Precipitation Seasonality, Bio15; and Precipitation of Driest Quarter, Bio 17; Table 1; Fig. 4). Bd infection intensity did not influence the beta diversity of amphibian skin bacterial communities (Table 1; Fig. 4).

### Bd infection intensity and status

Treefrog samples indicated a low Bd infection intensity in general, with a mean value of 287 zoospore equivalents per Bd positive individual and a high individual variability (min: 1, max: 12,104, SD: 604.94, SE: 21.51). Mean zoospore load was even lower (172 zoospores/individual) when excluding two outlier samples with extremely high values (9000 and 12,000). Bd infection status and Bd infection intensity were highest in intermediate elevations between 500 and 600 m (Figs. S2, S3). We found Bd infected treefrogs in all latitudinal groups but not at all localities (Figs. S3, S4).

Response screening revealed that Precipitation of the Driest Month (Bio14) had the strongest effect size on Bd infection intensity (Table S18), whereas Mean Temperature of Coldest Quarter (Bio 11) had the strongest effect size on Bd infection status but overall, the R-values were low (Table S19). GLMMs found no evidence for Bd infection intensity being affected by most of selected bioclimatic variables from Response Screening except of Annual Precipitation (Bio 12) nor by #OTUs or elevation and latitude (Table S12). Bd infection status was significantly negatively affected by selected bioclimatic variables, #OTUs, Elevation, and Latitude (Table 2): Mean Temperature of Coldest Quarter (Bio 11,) Mean Temperature of Driest Quarter (Bio 9), Minimum Temperature of Coldest Month (Bio 6), and Temperature Seasonality (Bio 4).

### Indicator species analysis

Indicator species analyses revealed a large number of OTUs that were differentially abundant across samples; 224 OTUs for latitudinal group NAF, 138 OTUs for latitudinal group CAF, 284 OTUs for latitudinal group SAF, 350 OTUs for Bd positive samples, and 16 OTUs for Bd negative samples (Table S8). The combination of NAF and CAF samples harbored 37 indicator OTUs, whereas the combination of CAF and SAF and NAF and SAF revealed 47 and 31 indicator species, respectively. This further suggests an overall higher similarity of the samples from NAF and SAF, which is also evident from the NMDS graphs (Fig. 3). Among the OTUs significantly more abundant in Bd negative treefrogs as revealed by indicator species analysis, 50% have a probable Bd inhibitory function, whereas among OTUs significantly more abundant in Bd positive samples, only 16% have a probable Bd inhibitory function (Table S8).

## Discussion

This study aimed to understand the environment-host–pathogen interaction as determinant of the skin bacterial richness and diversity of treefrogs sampled across the Atlantic Forest. Our survey revealed that temperature- and precipitation-associated factors, and elevation are consistently linked with richness, evenness, and diversity of the skin microbiome. Bioclimate also was a predictor of Bd infection status, and thereby, of Bd prevalence per site. Although anthropogenic environmental change is currently the most important threat that Atlantic Forest amphibians are facing^44,48,50^, they have been dramatically impacted by Bd in historical times^51^ and the combined effects of Bd and changing environmental conditions are still unclarified. Understanding the link between environmental factors and host susceptibility to pathogens is therefore essential for amphibian conservation.

### Bioclimatic data and elevation shape the amphibian skin microbiome

Beside host-specific effects such as ecology^8^, pathogen load^2,23^, life stage^102^, and species^103^, environmental factors are known to shape skin bacterial richness and diversity in amphibians^25,73,104,105^. Kueneman et al.^19^ examined skin surface microbiomes across 205 amphibian species and found strong correlations with bioclimatic factors such as temperature of the coldest month on a global scale. Woodhams et al.^10^ suggested that daily temperature range and precipitation of the warmest quarter^10^ explains external microbiome communities such as the skin microbiome best. Overall, the trends detected in our study at a more regional scale agree with an overall pattern encountered in bacterial communities, both those associated to the amphibian skin^10,19^ and others (reviewed in^105^): bacterial richness and evenness in general decrease with variables reflecting environmental temperature (e.g., richness: Bio 8; evenness: Bio 2, Bio 7, and Bio 11), although in our study the encountered effects were overall weaker, and the strongest influences were not exerted by the same Bioclim variables as in the global studies of Kueneman et al.^19^ and Woodhams et al.^10^. We further found a decreasing effect of elevation on bacterial richness and evenness. Both measures of alpha diversity were reduced at high elevations. Xu et al.^106^ could also demonstrate a decrease in skin bacterial richness in wild amphibians from the Tibetan Plateau with increasing elevation. However, Medina et al.^24^ found no effect of elevation on neither bacterial richness nor evenness in *Silverstoneia flotator*. We assume that an effect of elevation on bacterial richness is positively related to the range of measured elevations in a given data set, which was relatively wide in the present study with samples sites ranging between 9 and 1487 m above sea level.

The cutaneous microbiome evenness of treefrogs in the Atlantic Forest was not correlated with precipitation variables even if precipitation of the driest month (i.e., Bio 14) had the highest effect size on bacterial richness in Response screening. Consequently, treefrog skin microbiome richness in these frogs apparently is influenced by temperature and slightly by precipitation of dry and rainy season, i.e., skin bacterial species richness is highest at sample sites where precipitation during the dry season is high and temperature during the rainy season is low. Precipitation in the Atlantic Forest is generally high, but even within a rainforest we found a slight but positive effect of precipitation on microbial diversity indicating that rain is a very strong force driving frog microbiomes.

In contrast, our data did not reveal an impact of seasonal climatic changes on the amphibian skin microbial community, a trend that has been investigated in several studies^107–109^. In the present study, we tested the effect of seasonal variation in temperature and precipitation by use of annual variation (i.e. temperature and precipitation seasonality, Bio 4 and Bio 15) and in contrast to other studies^110^, did not find an effect of either temperature nor precipitation seasonality on skin microbial community. Possibly, at the regional scale of the Atlantic Forest, seasonality effects are not strong enough to become apparent, but it also must be stressed that the considering the large sample size, even the statistically significant correlations in our data sets are rather weak (Fig. 2). Considering this and the fact that many climatic factors are closely correlated with each other, teasing apart the exact causal associations between climate and amphibian-associated microbiomes requires further, more experimental approaches.

Our results meet the general pattern found by Kueneman et al.^19^ where higher skin bacterial diversity was found at higher latitudes, likely due to seasonality driving bacterial species turnover among seasons. Even if this result is not novel, it indicates that skin microbiome diversity responds to latitudinal variation even within the tropical range and without the drastic tropical-temperate-boreal shifts. The swabs used in this study were taken without previous rinsing of the frogs, and therefore, our analysis may include a certain proportion of transient bacteria that are no integral part of the amphibian's microbiome. We are convinced this proportion is rather low, as we limited our study to treefrogs which typically are devoid of soil particles on their skin which would constitute an important bias in bacterial communities from non-rinsed terrestrial frogs. In any case, considering the overall high temporal variation and reservoir-dependent composition of the cutaneous bacterial communities of amphibians (e.g., Bletz et al.^85^), a strict separation of transient and resident bacteria in these communities is difficult, and we speculate that even transient bacteria may in some cases exert particular functions, e.g., in the context of inhibiting or favoring Bd infection.

### Amphibian skin bacterial species richness correlates with Bd infection intensity

Symbiotic microbial communities can protect their hosts from diseases by directly inhibiting pathogens or increasing host immune capacity^31,69^. In turn, invading pathogens can alter the structure of these microbiomes^2,23,73^ and might increase host susceptibility to pathogens and thus, reduce their fitness^31^.

In this study, Bd infection intensity was not correlated with bacterial richness when including the entire data set in the study; however, this correlation became significant when excluding samples from uninfected frogs, i.e., zero values. In the infected frogs, individuals with higher Bd loads had microbiomes with slightly higher OTU richness. At first glance this contradicts the finding of other studies that found a reducing effect of Bd infection on bacterial richness on the amphibian skin^2,23,73,105,111^. However, these studies referred to high Bd infection intensities: when Bd load exceeds a certain threshold, it may cause dysbiosis and dramatically alter the host microbial communities^26,31,34,111^ which probably causes opportunistic bacteria to bloom and OTU richness thus to decrease. In the treefrogs sampled here, infected individuals had a low mean zoospore load (i.e., 172 zoospores/individual after excluding outliers), and the positive correlation between Bd load and bacterial richness we encountered in this setting cannot be readily explained. An experimental study found that Bd grows poorly when alongside diverse bacterial communities compared to depauperate bacterial communities^112^, which again would predict a correlation inverse from the one we found. However, our finding, wlthough only weakly supported, might be more than just a spurious result, as Becker et al.^31^, in a frog species from the Atlantic Forest, observed a weak but significant positive correlation of Bd infection load and skin bacterial richness. These authors found that bacterial diversity in frogs was higher in natural forest habitats compared to open disturbed environments^31^ since Bd has its optimal growth conditions in the mild microclimates of natural forests, where microbial diversity in environmental reservoirs is also higher. The correlation found here might therefore be driven by the fact that Bd prevalence and environmental microbial diversity are both higher in natural forests, without a causal link among them.

Correlations of microbiome patterns and Bd infection may be explained by the presence of certain Bd-inhibitory bacteria preventing a frog from getting infected; or by infected hosts increasing microbial diversity by recruiting beneficial bacteria^74,75^, e.g. by a change in behavior that brings them in contact with novel bacterial reservoirs. Recent studies have reported strong inhibitory/stimulatory effects of particular bacterial species on Bd infection^31,72,113,114^. In this study, indicator OTUs of both Bd negative and positive treefrogs belonged to bacterial families that inhibit Bd in vitro^68,115^ (e.g., Comamonadaceae, Pseudomonadaceae, Sphingomonadaceae), but none of the best indicator OTUs of Bd positive treefrogs belonged to families that stimulate Bd infection in vitro^68,115^. Alternatively, a higher bacterial richness in infected frogs could also be due to neutral processes; Bd itself can be seen as a (eukaryotic) member of the microbiome, and its colonization may be facilitated by a host's physical contact with a novel substrate, or with another infected amphibian^34^; such contacts could simultaneously also lead to the recruitment of additional bacterial OTUs, thus leading to an increase of bacterial richness.

## Conclusion

The present study confirms the influence of environmental factors such as temperature, precipitation, and elevation on the amphibian skin bacterial communities, on Bd infection status, and on the bi-directional link between skin bacterial communities and Bd to some extent. Even though the three different regions across the Atlantic Forest display a large heterogeneity, our results reveal general patterns of environmental effects and on this link based on a uniquely large dataset of sampled transects and host species. Determining the impact of environmental factors in general and thus, of changing environmental conditions associated with global (climate) change on this link is a major challenge for amphibian conservation^31,116^. Although shifts in amphibian skin bacterial richness and diversity have recently been linked to stochastic extreme weather events^117^, the effect of large-scale climate fluctuations on bacterial symbionts are not well understood^104^, especially in the Atlantic Forest. Further, only few studies have yet investigated the effect of variation in abiotic factors in general^21,38,74,102^ but not associated with global (climate) change. Although Bd has already caused amphibian declines and extinctions in the Atlantic Forest^51^, it remains unclear how stochastic environmental variation affects amphibian skin bacterial communities and functional diversity^38^. Future studies should focus on the interaction of several abiotic and biotic factors and how seasonal, but also long-term variation of such might impact the host–pathogen interaction of treefrogs in the Atlantic Forest. Furthermore, we flag testing the effects of weak Bd infection on microbiome and vice-versa, under semi-natural conditions, as an important subject for future experimental research.

## Supplementary Information

Supplementary Information.

## Data Availability

Raw data for all samples is available on the Sequence Read Archive (Bioproject PRJNA665482 to be added upon manuscript acceptance). All original collection metadata are provided as supplementary tables. There are no restrictions on data availability. Correspondence and requests for materials should be addressed to M.V. or K.R.
