# Supplementary material for: Skin microbiome correlates with bioclimate and Batrachochytrium dendrobatidis infection intensity in Brazil’s Atlantic Forest treefrogs
Source: Sci Rep. 2020 Dec 18;10:22311. doi: 10.1038/s41598-020-79130-3 (PMC7749163; doi:10.1038/s41598-020-79130-3)
Supplement: Supplementary file 1 — Supplementary Information. [file 41598_2020_79130_MOESM1_ESM.docx]

**Title: Skin microbiome correlates with bioclimate and *Batrachochytrium dendrobatidis* infection intensity in Brazil’s Atlantic Forest treefrogs**

**Running title: Cutaneous microbiome and Bd in treefrogs across Brazil’s Atlantic Forest**

Katharina Ruthsatz^1,9^, Mariana L. Lyra^2^, Carolina Lambertini^3^, Anat M. Belasen^4^, Thomas S. Jenkinson^5^, Domingos da Silva Leite^6^, C. Guilherme Becker^7^, Célio F. B. Haddad^2^, Timothy Y. James^8^, Kelly R. Zamudio^4^, Luís Felipe Toledo^3^, Miguel Vences^9^

*^1^Institute of Zoology, Universität Hamburg, Martin-Luther-King-Platz 3, 20146 Hamburg, Germany; katharina.ruthsatz@uni-hamburg.de*

*²Laboratório de Herpetologia, Depto de Biodiversidade, Instituto de Biociências and Centro de Aquicultura (CAUNESP), Universidade Estadual Paulista - UNESP, Rio Claro, São Paulo, Brazil;* [*marillyra@gmail.com*](mailto:marillyra@gmail.com)*; celio.haddad@unesp.br*

*^3^Laboratório de História Natural de Anfíbios Brasileiros (LaHNAB), Departamento de Biologia Animal, Instituto de Biologia, Universidade Estadual de Campinas, Campinas, São Paulo, 13083-862, Brazil; lambertini.carol@gmail.com; toledosapo@gmail.com*

*^4^Department of Ecology and Evolutionary Biology, Cornell University, Ithaca, NY 14853-2701, USA; amb684@cornell.edu, krz2@cornell.edu*

*^5^Department of Wildlife, Fish & Conservation Biology. University of California, Davis, Davis, CA, USA; tsjenkinson@ucdavis.edu*

*^6^Laboratório de Antígenos Bacterianos II, Departamento de Genética, Evolução, Microbiologia e Imunologia, Instituto de Biologia, Universidade Estadual de Campinas, Caixa Postal 6109, Campinas, São Paulo, CEP 13083‐862, Brazil; domingos@unicamp.br*

*^7^Department of Biological Sciences, The University of Alabama, Tuscaloosa, AL 35847, USA; cgbecker1@ua.edu*

*^8^Department of Ecology and Evolutionary Biology, University of Michigan, Ann Arbor, Michigan, 48109; tyjames@umich.edu*

*^9^Zoological Institute, Technische Universität Braunschweig, Mendelssohnstraße 4, 38106 Braunschweig, Germany; m.vences@tu-braunschweig.de*

Corresponding author: **Katharina Ruthsatz**, katharina.ruthsatz@uni-hamburg.de. ORCID: 0000-0002-3273-2826.

**Supplementary material**

**Supplementary Table 1**. Treefrog species sampled at 23 localities across Brazil’s Atlantic Forest. Localities were assigned to three main groups: North Atlantic Forest (NAF) between 5° and 15°S, Central Atlantic Forest (CAF) between 15° and 23°S, and South Atlantic Forest (SAF) from 23° to 30°S (*sensu*^45^). Reads = microbial sample size. Bd status = Bd infection status of individual. #OTUs = Number of operational taxonomic units. Rarefraction level was 1,000 reads per sample. N= 1140.

| **#SampleID** | **Genus** | **Species** | **State** | **Municipality** | **Latitude** | **Elevation** | **Location** | **Group** | **Sampling date** | **Reads** | **Bd status** | **#OTUs** | **Simpson's eveness** |
| --- | --- | --- | --- | --- | --- | --- | --- | --- | --- | --- | --- | --- | --- |
| SLFT5023 | *Agalychnis* | *Agalychnis granulosa* | Pernambuco | Paulista | -7.93 | 30 | 3 | NAF | 42164 | 34333 | - | 241 | 0.0729 |
| SLFT5025 | *Agalychnis* | *Agalychnis granulosa* | Pernambuco | Paulista | -7.93 | 30 | 3 | NAF | 42164 | 13166 | - | 260 | 0.1819 |
| SLFT0171 | *Aplastodiscus* | *Aplastodiscus eugenoi* | São Paulo | Ubatuba | -23.36 | 76 | 14 | CAF | 40840 | 38585 | + | 47 | 0.0823 |
| SLFT1251 | *Aplastodiscus* | *Aplastodiscus pervidis* | Santa Catarina | São Miguel d' Oeste | -26.76 | 571 | 20 | SAF | 41197 | 7169 | - | 390 | 0.1732 |
| SLFT1253 | *Aplastodiscus* | *Aplastodiscus pervidis* | Santa Catarina | São Miguel d' Oeste | -26.76 | 571 | 20 | SAF | 41197 | 3156 | + | 211 | 0.1205 |
| SLFT1255 | *Aplastodiscus* | *Aplastodiscus pervidis* | Santa Catarina | São Miguel d' Oeste | -26.76 | 571 | 20 | SAF | 41197 | 4264 | - | 193 | 0.0688 |
| SLFT1257 | *Aplastodiscus* | *Aplastodiscus pervidis* | Santa Catarina | São Miguel d' Oeste | -26.76 | 571 | 20 | SAF | 41197 | 1925 | + | 191 | 0.0397 |
| SLFT1273 | *Aplastodiscus* | *Aplastodiscus pervidis* | Santa Catarina | São Miguel d' Oeste | -26.76 | 571 | 20 | SAF | 41197 | 6794 | + | 282 | 0.2851 |
| SLFT1293 | *Aplastodiscus* | *Aplastodiscus pervidis* | Santa Catarina | São Miguel d' Oeste | -26.76 | 571 | 20 | SAF | 41197 | 10137 | - | 178 | 0.0283 |
| SLFT1297 | *Aplastodiscus* | *Aplastodiscus pervidis* | Santa Catarina | São Miguel d' Oeste | -26.76 | 571 | 20 | SAF | 41197 | 8118 | + | 221 | 0.0380 |
| SLFT1310 | *Aplastodiscus* | *Aplastodiscus pervidis* | Santa Catarina | São Miguel d' Oeste | -26.76 | 571 | 20 | SAF | 41197 | 9064 | + | 147 | 0.0515 |
| SLFT1311 | *Aplastodiscus* | *Aplastodiscus pervidis* | Santa Catarina | São Miguel d' Oeste | -26.76 | 571 | 20 | SAF | 41197 | 5507 | + | 142 | 0.0775 |
| SLFT1313 | *Aplastodiscus* | *Aplastodiscus pervidis* | Santa Catarina | São Miguel d' Oeste | -26.76 | 571 | 20 | SAF | 41197 | 5469 | + | 268 | 0.0843 |
| SLFT1315 | *Aplastodiscus* | *Aplastodiscus pervidis* | Santa Catarina | São Miguel d' Oeste | -26.76 | 571 | 20 | SAF | 41197 | 2732 | + | 100 | 0.0287 |
| SLFT1317 | *Aplastodiscus* | *Aplastodiscus pervidis* | Santa Catarina | São Miguel d' Oeste | -26.76 | 571 | 20 | SAF | 41197 | 2108 | - | 59 | 0.0783 |
| SLFT1319 | *Aplastodiscus* | *Aplastodiscus pervidis* | Santa Catarina | São Miguel d' Oeste | -26.76 | 571 | 20 | SAF | 41197 | 10662 | + | 139 | 0.0251 |
| SLFT1322 | *Aplastodiscus* | *Aplastodiscus pervidis* | Santa Catarina | São Miguel d' Oeste | -26.76 | 571 | 20 | SAF | 41197 | 6088 | + | 138 | 0.1708 |
| SLFT1326 | *Aplastodiscus* | *Aplastodiscus pervidis* | Santa Catarina | São Miguel d' Oeste | -26.76 | 571 | 20 | SAF | 41197 | 1863 | NA | 91 | 0.0628 |
| SLFT1349 | *Aplastodiscus* | *Aplastodiscus pervidis* | Santa Catarina | São Miguel d' Oeste | -26.76 | 571 | 20 | SAF | 41197 | 25251 | - | 26 | 0.0804 |
| SLFT1351 | *Aplastodiscus* | *Aplastodiscus pervidis* | Santa Catarina | São Miguel d' Oeste | -26.76 | 571 | 20 | SAF | 41197 | 6511 | NA | 358 | 0.2356 |
| SLFT1354 | *Aplastodiscus* | *Aplastodiscus pervidis* | Santa Catarina | São Miguel d' Oeste | -26.76 | 571 | 20 | SAF | 41197 | 3849 | NA | 198 | 0.0314 |
| SLFT1355 | *Aplastodiscus* | *Aplastodiscus pervidis* | Santa Catarina | São Miguel d' Oeste | -26.76 | 571 | 20 | SAF | 41197 | 6765 | NA | 291 | 0.0680 |
| SLFT1357 | *Aplastodiscus* | *Aplastodiscus pervidis* | Santa Catarina | São Miguel d' Oeste | -26.76 | 571 | 20 | SAF | 41197 | 3451 | NA | 257 | 0.0240 |
| SLFT1373 | *Aplastodiscus* | *Aplastodiscus pervidis* | Santa Catarina | São Miguel d' Oeste | -26.76 | 571 | 20 | SAF | 41197 | 10196 | NA | 282 | 0.1868 |
| SLFT1942 | *Aplastodiscus* | *Aplastodiscus* sp*.* | Bahia | Camacan | -15.38 | 41 | 7 | NAF | 41647 | 4590 | - | 35 | 0.1799 |
| SLFT2286 | *Aplastodiscus* | *Aplastodiscus arildae* | Espírito Santo | Vargem Alta | -20.48 | 1148 | 10 | CAF | 41653 | 1033 | - | 45 | 0.1232 |
| SLFT2297 | *Aplastodiscus* | *Aplastodiscus arildae* | Espírito Santo | Vargem Alta | -20.48 | 1148 | 10 | CAF | 41653 | 5474 | - | 24 | 0.1783 |
| SLFT2969 | *Aplastodiscus* | *Aplastodiscus* sp*.* | Paraná | Morretes | -25.40 | 426 | 19 | SAF | 41283 | 6906 | - | 29 | 0.0984 |
| SLFT0013 | *Boana* | *Boana albomarginata* | São Paulo | Iporanga | -24.60 | 412 | 18 | CAF | 41281 | 2035 | - | 27 | 0.1438 |
| SLFT0137 | *Boana* | *Boana bischoffi* | Paraná | Morretes | -25.40 | 426 | 19 | SAF | 41283 | 5883 | + | 76 | 0.1467 |
| SLFT0169 | *Boana* | *Boana faber* | São Paulo | Ubatuba | -23.36 | 76 | 14 | CAF | 40840 | 34549 | + | 40 | 0.0638 |
| SLFT0172 | *Boana* | *Boana albomarginata* | São Paulo | Ubatuba | -23.36 | 76 | 14 | CAF | 40840 | 1604 | + | 30 | 0.0511 |
| SLFT0173 | *Boana* | *Boana albomarginata* | São Paulo | Ubatuba | -23.36 | 76 | 14 | CAF | 40840 | 54271 | - | 26 | 0.1398 |
| SLFT0174 | *Boana* | *Boana albomarginata* | São Paulo | Ubatuba | -23.36 | 76 | 14 | CAF | 40840 | 38443 | - | 48 | 0.0888 |
| SLFT0489 | *Boana* | *Boana faber* | São Paulo | Jundiaí | -23.23 | 869 | 13 | CAF | 41294 | 3927 | + | 190 | 0.0779 |
| SLFT0490 | *Boana* | *Boana faber* | São Paulo | Jundiaí | -23.23 | 869 | 13 | CAF | 41294 | 2964 | - | 213 | 0.0231 |
| SLFT0491 | *Boana* | *Boana faber* | São Paulo | Jundiaí | -23.23 | 869 | 13 | CAF | 41294 | 8252 | - | 445 | 0.3576 |
| SLFT0492 | *Boana* | *Boana bischoffi* | São Paulo | Jundiaí | -23.23 | 869 | 13 | CAF | 41294 | 19161 | - | 47 | 0.0748 |
| SLFT0493 | *Boana* | *Boana faber* | São Paulo | Jundiaí | -23.23 | 869 | 13 | CAF | 41294 | 22771 | + | 185 | 0.0237 |
| SLFT0496 | *Boana* | *Boana bischoffi* | São Paulo | Jundiaí | -23.23 | 869 | 13 | CAF | 41294 | 5906 | - | 51 | 0.0361 |
| SLFT0497 | *Boana* | *Boana prasina* | São Paulo | Jundiaí | -23.23 | 869 | 13 | CAF | 41294 | 1188 | - | 134 | 0.0535 |
| SLFT0498 | *Boana* | *Boana faber* | São Paulo | Jundiaí | -23.23 | 869 | 13 | CAF | 41294 | 26040 | - | 89 | 0.0221 |
| SLFT0499 | *Boana* | *Boana bischoffi* | São Paulo | Jundiaí | -23.23 | 869 | 13 | CAF | 41294 | 6400 | - | 53 | 0.0348 |
| SLFT0501 | *Boana* | *Boana bischoffi* | São Paulo | Jundiaí | -23.23 | 869 | 13 | CAF | 41294 | 1694 | - | 72 | 0.0373 |
| SLFT0502 | *Boana* | *Boana prasina* | São Paulo | Jundiaí | -23.23 | 869 | 13 | CAF | 41294 | 4568 | - | 29 | 0.0602 |
| SLFT0503 | *Boana* | *Boana prasina* | São Paulo | Jundiaí | -23.23 | 869 | 13 | CAF | 41294 | 12444 | + | 33 | 0.0684 |
| SLFT0504 | *Boana* | *Boana faber* | São Paulo | Jundiaí | -23.23 | 869 | 13 | CAF | 41294 | 6378 | + | 54 | 0.0504 |
| SLFT0505 | *Boana* | *Boana bischoffi* | São Paulo | Jundiaí | -23.23 | 869 | 13 | CAF | 41294 | 6378 | - | 75 | 0.0426 |
| SLFT0507 | *Boana* | *Boana faber* | São Paulo | Iporanga | -24.60 | 412 | 18 | CAF | 41281 | 21096 | - | 42 | 0.0821 |
| SLFT0508 | *Boana* | *Boana faber* | São Paulo | Iporanga | -24.60 | 412 | 18 | CAF | 41281 | 11704 | - | 13 | 0.1284 |
| SLFT0509 | *Boana* | *Boana faber* | São Paulo | Iporanga | -24.60 | 412 | 18 | CAF | 41281 | 9607 | - | 67 | 0.1018 |
| SLFT0510 | *Boana* | *Boana faber* | São Paulo | Iporanga | -24.60 | 412 | 18 | CAF | 41281 | 14502 | - | 35 | 0.1187 |
| SLFT0511 | *Boana* | *Boana faber* | São Paulo | Iporanga | -24.60 | 412 | 18 | CAF | 41281 | 16947 | + | 218 | 0.2510 |
| SLFT0512 | *Boana* | *Boana faber* | São Paulo | Iporanga | -24.60 | 412 | 18 | CAF | 41281 | 77970 | - | 3 | 0.1452 |
| SLFT0514 | *Boana* | *Boana faber* | São Paulo | Iporanga | -24.60 | 412 | 18 | CAF | 41281 | 13960 | - | 362 | 0.3409 |
| SLFT0516 | *Boana* | *Boana faber* | São Paulo | Iporanga | -24.60 | 412 | 18 | CAF | 41281 | 10950 | - | 47 | 0.0986 |
| SLFT0531 | *Boana* | *Boana albomarginata* | São Paulo | Iporanga | -24.60 | 412 | 18 | CAF | 41281 | 26968 | - | 22 | 0.0814 |
| SLFT0538 | *Boana* | *Boana faber* | São Paulo | Iporanga | -24.60 | 412 | 18 | CAF | 41281 | 9767 | + | 70 | 0.0320 |
| SLFT0539 | *Boana* | *Boana faber* | São Paulo | Iporanga | -24.60 | 412 | 18 | CAF | 41281 | 21500 | - | 109 | 0.1744 |
| SLFT0540 | *Boana* | *Boana faber* | São Paulo | Iporanga | -24.60 | 412 | 18 | CAF | 41281 | 10950 | + | 4 | 0.5010 |
| SLFT0553 | *Boana* | *Boana faber* | São Paulo | Iporanga | -24.60 | 412 | 18 | CAF | 41281 | 14011 | + | 35 | 0.1365 |
| SLFT0555 | *Boana* | *Boana faber* | São Paulo | Iporanga | -24.60 | 412 | 18 | CAF | 41281 | 58369 | - | 18 | 0.1411 |
| SLFT0562 | *Boana* | *Boana faber* | São Paulo | Iporanga | -24.60 | 412 | 18 | CAF | 41281 | 61828 | + | 24 | 0.1611 |
| SLFT0563 | *Boana* | *Boana faber* | São Paulo | Iporanga | -24.60 | 412 | 18 | CAF | 41281 | 79261 | + | 8 | 0.2464 |
| SLFT0572 | *Boana* | *Boana albomarginata* | São Paulo | Iporanga | -24.60 | 412 | 18 | CAF | 41281 | 1174 | - | 40 | 0.1477 |
| SLFT0574 | *Boana* | *Boana albomarginata* | São Paulo | Iporanga | -24.60 | 412 | 18 | CAF | 41281 | 22101 | - | 54 | 0.0525 |
| SLFT0609 | *Boana* | *Boana faber* | São Paulo | Iporanga | -24.60 | 412 | 18 | CAF | 41281 | 38245 | - | 80 | 0.1403 |
| SLFT0622 | *Boana* | *Boana albomarginata* | São Paulo | Iporanga | -24.60 | 412 | 18 | CAF | 41281 | 96601 | - | 4 | 0.2525 |
| SLFT0627 | *Boana* | *Boana albomarginata* | São Paulo | Iporanga | -24.60 | 412 | 18 | CAF | 41281 | 26993 | - | 34 | 0.0783 |
| SLFT0630 | *Boana* | *Boana faber* | São Paulo | Iporanga | -24.60 | 412 | 18 | CAF | 41281 | 44661 | + | 47 | 0.0579 |
| SLFT0640 | *Boana* | *Boana faber* | Paraná | Morretes | -25.40 | 426 | 19 | SAF | 41283 | 17606 | - | 37 | 0.1309 |
| SLFT0642 | *Boana* | *Boana faber* | Paraná | Morretes | -25.40 | 426 | 19 | SAF | 41283 | 17334 | + | 18 | 0.2041 |
| SLFT0664 | *Boana* | *Boana semilineata* | Paraná | Morretes | -25.40 | 426 | 19 | SAF | 41283 | 12423 | - | 9 | 0.0793 |
| SLFT0687 | *Boana* | *Boana semilineata* | Paraná | Morretes | -25.40 | 426 | 19 | SAF | 41283 | 56235 | - | 9 | 0.1137 |
| SLFT0689 | *Boana* | *Boana semilineata* | Paraná | Morretes | -25.40 | 426 | 19 | SAF | 41283 | 50441 | + | 7 | 0.1792 |
| SLFT0723 | *Boana* | *Boana semilineata* | Paraná | Morretes | -25.40 | 426 | 19 | SAF | 41283 | 60373 | + | 13 | 0.1067 |
| SLFT0749 | *Boana* | *Boana faber* | Santa Catarina | Rancho Queimado | -27.67 | 959 | 22 | SAF | 41284 | 34583 | + | 7 | 0.2875 |
| SLFT0758 | *Boana* | *Boana bischoffi* | Santa Catarina | Rancho Queimado | -27.67 | 959 | 22 | SAF | 41284 | 6283 | - | 144 | 0.0833 |
| SLFT0778 | *Boana* | *Boana bischoffi* | Santa Catarina | Rancho Queimado | -27.67 | 959 | 22 | SAF | 41284 | 4088 | - | 94 | 0.0461 |
| SLFT0785 | *Boana* | *Boana faber* | Santa Catarina | Rancho Queimado | -27.67 | 959 | 22 | SAF | 41284 | 11299 | - | 4 | 0.5681 |
| SLFT0792 | *Boana* | *Boana faber* | Santa Catarina | Rancho Queimado | -27.67 | 959 | 22 | SAF | 41284 | 1155 | - | 11 | 0.0978 |
| SLFT0795 | *Boana* | *Boana faber* | Santa Catarina | Rancho Queimado | -27.67 | 959 | 22 | SAF | 41284 | 24908 | + | 16 | 0.0980 |
| SLFT0802 | *Boana* | *Boana faber* | Santa Catarina | Rancho Queimado | -27.67 | 959 | 22 | SAF | 41284 | 28682 | - | 121 | 0.0185 |
| SLFT0803 | *Boana* | *Boana faber* | Santa Catarina | Rancho Queimado | -27.67 | 959 | 22 | SAF | 41284 | 6544 | - | 35 | 0.0891 |
| SLFT0804 | *Boana* | *Boana bischoffi* | Santa Catarina | Rancho Queimado | -27.67 | 959 | 22 | SAF | 41284 | 2097 | + | 47 | 0.0528 |
| SLFT0806 | *Boana* | *Boana faber* | Santa Catarina | Rancho Queimado | -27.67 | 959 | 22 | SAF | 41284 | 7909 | - | 77 | 0.1378 |
| SLFT0807 | *Boana* | *Boana faber* | Santa Catarina | Rancho Queimado | -27.67 | 959 | 22 | SAF | 41284 | 42763 | + | 10 | 0.0735 |
| SLFT0808 | *Boana* | *Boana bischoffi* | Santa Catarina | Rancho Queimado | -27.67 | 959 | 22 | SAF | 41284 | 3940 | - | 51 | 0.0550 |
| SLFT0810 | *Boana* | *Boana bischoffi* | Santa Catarina | Rancho Queimado | -27.67 | 959 | 22 | SAF | 41284 | 13085 | + | 50 | 0.0710 |
| SLFT0813 | *Boana* | *Boana bischoffi* | Santa Catarina | Rancho Queimado | -27.67 | 959 | 22 | SAF | 41284 | 10753 | - | 48 | 0.0505 |
| SLFT0814 | *Boana* | *Boana faber* | Santa Catarina | Rancho Queimado | -27.67 | 959 | 22 | SAF | 41284 | 33830 | - | 2 | 0.1711 |
| SLFT0829 | *Boana* | *Boana faber* | Santa Catarina | Rancho Queimado | -27.67 | 959 | 22 | SAF | 41284 | 88459 | - | 5 | 0.3470 |
| SLFT0831 | *Boana* | *Boana faber* | Santa Catarina | Rancho Queimado | -27.67 | 959 | 22 | SAF | 41284 | 1311 | - | 181 | 0.2197 |
| SLFT0832 | *Boana* | *Boana faber* | Santa Catarina | Rancho Queimado | -27.67 | 959 | 22 | SAF | 41284 | 3161 | - | 268 | 0.1172 |
| SLFT0838 | *Boana* | *Boana faber* | Santa Catarina | Rancho Queimado | -27.67 | 959 | 22 | SAF | 41284 | 20313 | - | 7 | 0.3040 |
| SLFT0840 | *Boana* | *Boana faber* | Santa Catarina | Rancho Queimado | -27.67 | 959 | 22 | SAF | 41284 | 12862 | + | 200 | 0.1071 |
| SLFT0845 | *Boana* | *Boana faber* | Santa Catarina | Rancho Queimado | -27.67 | 959 | 22 | SAF | 41284 | 40448 | - | 9 | 0.1714 |
| SLFT0847 | *Boana* | *Boana bischoffi* | Santa Catarina | Rancho Queimado | -27.67 | 959 | 22 | SAF | 41284 | 10252 | - | 107 | 0.0799 |
| SLFT0855 | *Boana* | *Boana faber* | Santa Catarina | Pomerode | -26.77 | 228 | 21 | SAF | 41285 | 2478 | - | 28 | 0.0392 |
| SLFT0859 | *Boana* | *Boana faber* | Santa Catarina | Pomerode | -26.77 | 228 | 21 | SAF | 41285 | 2742 | - | 129 | 0.0896 |
| SLFT0869 | *Boana* | *Boana faber* | Santa Catarina | Pomerode | -26.77 | 228 | 21 | SAF | 41285 | 1541 | - | 271 | 0.1570 |
| SLFT0879 | *Boana* | *Boana faber* | Santa Catarina | Pomerode | -26.77 | 228 | 21 | SAF | 41285 | 38302 | - | 8 | 0.3143 |
| SLFT0883 | *Boana* | *Boana bischoffi* | Santa Catarina | Pomerode | -26.77 | 228 | 21 | SAF | 41285 | 3959 | - | 204 | 0.2083 |
| SLFT0884 | *Boana* | *Boana bischoffi* | Santa Catarina | Pomerode | -26.77 | 228 | 21 | SAF | 41285 | 9360 | - | 37 | 0.0701 |
| SLFT0886 | *Boana* | *Boana bischoffi* | Santa Catarina | Pomerode | -26.77 | 228 | 21 | SAF | 41285 | 2997 | - | 144 | 0.0257 |
| SLFT0889 | *Boana* | *Boana bischoffi* | Santa Catarina | Pomerode | -26.77 | 228 | 21 | SAF | 41285 | 22983 | - | 9 | 0.1707 |
| SLFT0891 | *Boana* | *Boana bischoffi* | Santa Catarina | Pomerode | -26.77 | 228 | 21 | SAF | 41285 | 2457 | - | 142 | 0.0802 |
| SLFT0892 | *Boana* | *Boana bischoffi* | Santa Catarina | Pomerode | -26.77 | 228 | 21 | SAF | 41285 | 39999 | + | 7 | 0.1932 |
| SLFT0895 | *Boana* | *Boana bischoffi* | Santa Catarina | Pomerode | -26.77 | 228 | 21 | SAF | 41285 | 11623 | - | 61 | 0.0375 |
| SLFT0896 | *Boana* | *Boana bischoffi* | Santa Catarina | Pomerode | -26.77 | 228 | 21 | SAF | 41285 | 1570 | - | 42 | 0.0728 |
| SLFT0897 | *Boana* | *Boana bischoffi* | Santa Catarina | Pomerode | -26.77 | 228 | 21 | SAF | 41285 | 4724 | - | 9 | 0.2392 |
| SLFT0900 | *Boana* | *Boana faber* | Santa Catarina | Pomerode | -26.77 | 228 | 21 | SAF | 41285 | 54018 | - | 2 | 0.5081 |
| SLFT0907 | *Boana* | *Boana bischoffi* | Santa Catarina | Pomerode | -26.77 | 228 | 21 | SAF | 41285 | 11271 | + | 47 | 0.0512 |
| SLFT0909 | *Boana* | *Boana bischoffi* | Santa Catarina | Pomerode | -26.77 | 228 | 21 | SAF | 41285 | 2534 | - | 78 | 0.0551 |
| SLFT0911 | *Boana* | *Boana bischoffi* | Santa Catarina | Pomerode | -26.77 | 228 | 21 | SAF | 41285 | 5776 | - | 76 | 0.1063 |
| SLFT0912 | *Boana* | *Boana bischoffi* | Santa Catarina | Pomerode | -26.77 | 228 | 21 | SAF | 41285 | 1651 | - | 53 | 0.1205 |
| SLFT0913 | *Boana* | *Boana bischoffi* | Santa Catarina | Pomerode | -26.77 | 228 | 21 | SAF | 41285 | 9200 | - | 76 | 0.0862 |
| SLFT0914 | *Boana* | *Boana bischoffi* | Santa Catarina | Pomerode | -26.77 | 228 | 21 | SAF | 41285 | 3429 | - | 203 | 0.1446 |
| SLFT0917 | *Boana* | *Boana bischoffi* | Santa Catarina | Pomerode | -26.77 | 228 | 21 | SAF | 41285 | 1988 | - | 98 | 0.0512 |
| SLFT0918 | *Boana* | *Boana bischoffi* | Santa Catarina | Pomerode | -26.77 | 228 | 21 | SAF | 41285 | 1431 | - | 34 | 0.0862 |
| SLFT0919 | *Boana* | *Boana bischoffi* | Santa Catarina | Pomerode | -26.77 | 228 | 21 | SAF | 41285 | 7913 | - | 53 | 0.0560 |
| SLFT0921 | *Boana* | *Boana bischoffi* | Santa Catarina | Pomerode | -26.77 | 228 | 21 | SAF | 41285 | 3996 | - | 58 | 0.0748 |
| SLFT1026 | *Boana* | *Boana polytaenia* | Rio de Janeiro | Teresópolis | -22.46 | 1487 | 12 | CAF | 41635 | 56955 | - | 95 | 0.0313 |
| SLFT1027 | *Boana* | *Boana polytaenia* | Rio de Janeiro | Teresópolis | -22.46 | 1487 | 12 | CAF | 41635 | 28437 | + | 83 | 0.0614 |
| SLFT1028 | *Boana* | *Boana polytaenia* | Rio de Janeiro | Teresópolis | -22.46 | 1487 | 12 | CAF | 41635 | 14459 | + | 71 | 0.1261 |
| SLFT1032 | *Boana* | *Boana polytaenia* | Rio de Janeiro | Teresópolis | -22.46 | 1487 | 12 | CAF | 41635 | 23374 | - | 169 | 0.0689 |
| SLFT1038 | *Boana* | *Boana polytaenia* | Rio de Janeiro | Teresópolis | -22.46 | 1487 | 12 | CAF | 41635 | 66326 | - | 40 | 0.0476 |
| SLFT1039 | *Boana* | *Boana polytaenia* | Rio de Janeiro | Teresópolis | -22.46 | 1487 | 12 | CAF | 41635 | 29103 | - | 84 | 0.0519 |
| SLFT1041 | *Boana* | *Boana faber* | Rio de Janeiro | Teresópolis | -22.46 | 1487 | 12 | CAF | 41635 | 51794 | + | 42 | 0.0574 |
| SLFT1042 | *Boana* | *Boana faber* | Rio de Janeiro | Teresópolis | -22.46 | 1487 | 12 | CAF | 41635 | 13847 | + | 149 | 0.2969 |
| SLFT1043 | *Boana* | *Boana polytaenia* | Rio de Janeiro | Teresópolis | -22.46 | 1487 | 12 | CAF | 41635 | 19255 | - | 97 | 0.0388 |
| SLFT1045 | *Boana* | *Boana faber* | Rio de Janeiro | Teresópolis | -22.46 | 1487 | 12 | CAF | 41635 | 28931 | + | 14 | 0.2279 |
| SLFT1047 | *Boana* | *Boana pardalis* | Rio de Janeiro | Teresópolis | -22.46 | 1487 | 12 | CAF | 41635 | 19699 | - | 350 | 0.0351 |
| SLFT1053 | *Boana* | *Boana polytaenia* | Rio de Janeiro | Teresópolis | -22.46 | 1487 | 12 | CAF | 41635 | 27893 | - | 91 | 0.0694 |
| SLFT1057 | *Boana* | *Boana polytaenia* | Rio de Janeiro | Teresópolis | -22.46 | 1487 | 12 | CAF | 41635 | 30051 | + | 132 | 0.0573 |
| SLFT1058 | *Boana* | *Boana polytaenia* | Rio de Janeiro | Teresópolis | -22.46 | 1487 | 12 | CAF | 41635 | 33775 | - | 82 | 0.0540 |
| SLFT1059 | *Boana* | *Boana polytaenia* | Rio de Janeiro | Teresópolis | -22.46 | 1487 | 12 | CAF | 41635 | 38478 | + | 51 | 0.0946 |
| SLFT1092 | *Boana* | *Boana albomarginata* | São Paulo | Pedro de Toledo | -24.32 | 209 | 16 | CAF | 41328 | 18439 | - | 219 | 0.0808 |
| SLFT1094 | *Boana* | *Boana albomarginata* | São Paulo | Pedro de Toledo | -24.32 | 209 | 16 | CAF | 41328 | 41882 | - | 117 | 0.0262 |
| SLFT1100 | *Boana* | *Boana albomarginata* | São Paulo | Iguape | -24.50 | 16 | 17 | CAF | 2013 | 35014 | + | 49 | 0.0602 |
| SLFT1106 | *Boana* | *Boana albomarginata* | São Paulo | Iguape | -24.50 | 16 | 17 | CAF | 2013 | 52066 | - | 20 | 0.0684 |
| SLFT1260 | *Boana* | *Boana faber* | Santa Catarina | São Miguel d' Oeste | -26.76 | 571 | 20 | SAF | 41197 | 2583 | + | 206 | 0.0393 |
| SLFT1266 | *Boana* | *Boana curupi* | Santa Catarina | São Miguel d' Oeste | -26.76 | 571 | 20 | SAF | 41197 | 7370 | + | 449 | 0.1067 |
| SLFT1268 | *Boana* | *Boana curupi* | Santa Catarina | São Miguel d' Oeste | -26.76 | 571 | 20 | SAF | 41197 | 6525 | + | 266 | 0.0786 |
| SLFT1312 | *Boana* | *Boana curupi* | Santa Catarina | São Miguel d' Oeste | -26.76 | 571 | 20 | SAF | 41197 | 5399 | + | 165 | 0.0806 |
| SLFT1318 | *Boana* | *Boana faber* | Santa Catarina | São Miguel d' Oeste | -26.76 | 571 | 20 | SAF | 41197 | 8949 | - | 39 | 0.1675 |
| SLFT1345 | *Boana* | *Boana faber* | Santa Catarina | São Miguel d' Oeste | -26.76 | 571 | 20 | SAF | 41197 | 6006 | + | 17 | 0.1353 |
| SLFT1346 | *Boana* | *Boana faber* | Santa Catarina | São Miguel d' Oeste | -26.76 | 571 | 20 | SAF | 41197 | 3871 | + | 14 | 0.0872 |
| SLFT1347 | *Boana* | *Boana curupi* | Santa Catarina | São Miguel d' Oeste | -26.76 | 571 | 20 | SAF | 41197 | 22734 | - | 15 | 0.0930 |
| SLFT1348 | *Boana* | *Boana faber* | Santa Catarina | São Miguel d' Oeste | -26.76 | 571 | 20 | SAF | 41197 | 2803 | - | 70 | 0.1088 |
| SLFT1360 | *Boana* | *Boana faber* | Santa Catarina | São Miguel d' Oeste | -26.76 | 571 | 20 | SAF | 41197 | 11357 | NA | 288 | 0.0381 |
| SLFT1361 | *Boana* | *Boana curupi* | Santa Catarina | São Miguel d' Oeste | -26.76 | 571 | 20 | SAF | 41197 | 5742 | NA | 396 | 0.2577 |
| SLFT1368 | *Boana* | *Boana curupi* | Santa Catarina | São Miguel d' Oeste | -26.76 | 571 | 20 | SAF | 41197 | 16687 | NA | 320 | 0.0639 |
| SLFT1474 | *Boana* | *Boana albomarginata* | São Paulo | Itariri | -24.31 | 180 | 15 | CAF | 41329 | 30242 | - | 42 | 0.2118 |
| SLFT1475 | *Boana* | *Boana albomarginata* | São Paulo | Itariri | -24.31 | 180 | 15 | CAF | 41329 | 11289 | - | 44 | 0.1869 |
| SLFT1476 | *Boana* | *Boana albomarginata* | São Paulo | Itariri | -24.31 | 180 | 15 | CAF | 41329 | 22097 | - | 65 | 0.0878 |
| SLFT1477 | *Boana* | *Boana albomarginata* | São Paulo | Itariri | -24.31 | 180 | 15 | CAF | 41329 | 3376 | - | 59 | 0.0691 |
| SLFT1478 | *Boana* | *Boana albomarginata* | São Paulo | Itariri | -24.31 | 180 | 15 | CAF | 41329 | 29079 | - | 63 | 0.0849 |
| SLFT1479 | *Boana* | *Boana albomarginata* | São Paulo | Itariri | -24.31 | 180 | 15 | CAF | 41329 | 18789 | - | 43 | 0.0647 |
| SLFT1480 | *Boana* | *Boana albomarginata* | São Paulo | Itariri | -24.31 | 180 | 15 | CAF | 41329 | 27364 | - | 88 | 0.1110 |
| SLFT1482 | *Boana* | *Boana albomarginata* | São Paulo | Itariri | -24.31 | 180 | 15 | CAF | 41329 | 38389 | - | 34 | 0.1301 |
| SLFT1483 | *Boana* | *Boana albomarginata* | São Paulo | Itariri | -24.31 | 180 | 15 | CAF | 41329 | 10800 | - | 92 | 0.1258 |
| SLFT1529 | *Boana* | *Boana polytaenia* | Rio de Janeiro | Teresópolis | -22.46 | 1487 | 12 | CAF | 41411 | 8659 | + | 71 | 0.0375 |
| SLFT1531 | *Boana* | *Boana polytaenia* | Rio de Janeiro | Teresópolis | -22.46 | 1487 | 12 | CAF | 41411 | 2906 | + | 88 | 0.1037 |
| SLFT1534 | *Boana* | *Boana faber* | Rio de Janeiro | Teresópolis | -22.46 | 1487 | 12 | CAF | 41411 | 2420 | + | 90 | 0.0343 |
| SLFT1603 | *Boana* | *Boana polytaenia* | Rio de Janeiro | Teresópolis | -22.46 | 1487 | 12 | CAF | 41411 | 1612 | + | 115 | 0.1352 |
| SLFT1604 | *Boana* | *Boana polytaenia* | Rio de Janeiro | Teresópolis | -22.46 | 1487 | 12 | CAF | 41411 | 1331 | - | 146 | 0.1635 |
| SLFT1607 | *Boana* | *Boana polytaenia* | Rio de Janeiro | Teresópolis | -22.46 | 1487 | 12 | CAF | 41411 | 5835 | - | 121 | 0.1038 |
| SLFT1609 | *Boana* | *Boana polytaenia* | Rio de Janeiro | Teresópolis | -22.46 | 1487 | 12 | CAF | 41411 | 3481 | - | 59 | 0.0402 |
| SLFT1612 | *Boana* | *Boana polytaenia* | Rio de Janeiro | Teresópolis | -22.46 | 1487 | 12 | CAF | 41411 | 5000 | - | 224 | 0.0778 |
| SLFT1889 | *Boana* | *Boana exastis* | Alagoas | Murici | -9.21 | 233 | 4 | NAF | 41520 | 10100 | + | 24 | 0.0633 |
| SLFT1919 | *Boana* | *Boana albomarginata* | Alagoas | Murici | -9.21 | 233 | 4 | NAF | 41520 | 82527 | + | 58 | 0.1110 |
| SLFT1924 | *Boana* | *Boana semilineata* | Alagoas | Murici | -9.21 | 233 | 4 | NAF | 41520 | 13014 | - | 58 | 0.1172 |
| SLFT1961 | *Boana* | *Boana faber* | Bahia | Camacan | -15.38 | 376 | 7 | NAF | 41647 | 16001 | - | 60 | 0.0452 |
| SLFT1967 | *Boana* | *Boana faber* | Bahia | Camacan | -15.38 | 376 | 7 | NAF | 41647 | 33068 | - | 15 | 0.2215 |
| SLFT1969 | *Boana* | *Boana faber* | Bahia | Camacan | -15.38 | 376 | 7 | NAF | 41647 | 82939 | + | 5 | 0.4689 |
| SLFT1987 | *Boana* | *Boana albomarginata* | Bahia | Camacan | -15.38 | 376 | 7 | NAF | 41647 | 5548 | - | 18 | 0.0904 |
| SLFT1992 | *Boana* | *Boana albomarginata* | Bahia | Camacan | -15.38 | 376 | 7 | NAF | 41647 | 8983 | - | 62 | 0.0671 |
| SLFT2003 | *Boana* | *Boana semilineata* | Bahia | Camacan | -15.38 | 376 | 7 | NAF | 41647 | 16966 | - | 58 | 0.1680 |
| SLFT2004 | *Boana* | *Boana semilineata* | Bahia | Camacan | -15.38 | 376 | 7 | NAF | 41647 | 9443 | - | 19 | 0.2298 |
| SLFT2204 | *Boana* | *Boana albomarginata* | Espírito Santo | Santa Teresa | -19.92 | 810 | 9 | CAF | 41651 | 7483 | + | 38 | 0.1992 |
| SLFT2220 | *Boana* | *Boana faber* | Espírito Santo | Santa Teresa | -19.92 | 810 | 9 | CAF | 41651 | 7426 | - | 12 | 0.1981 |
| SLFT2228 | *Boana* | *Boana albomarginata* | Espírito Santo | Santa Teresa | -19.92 | 810 | 9 | CAF | 41651 | 16109 | - | 47 | 0.0471 |
| SLFT2229 | *Boana* | *Boana semilineata* | Espírito Santo | Santa Teresa | -19.92 | 810 | 9 | CAF | 41651 | 10353 | - | 43 | 0.0705 |
| SLFT2253 | *Boana* | *Boana semilineata* | Espírito Santo | Santa Teresa | -19.92 | 810 | 9 | CAF | 41651 | 6613 | - | 27 | 0.1704 |
| SLFT2254 | *Boana* | *Boana albomarginata* | Espírito Santo | Santa Teresa | -19.92 | 810 | 9 | CAF | 41651 | 8317 | - | 38 | 0.1128 |
| SLFT2269 | *Boana* | *Boana semilineata* | Espírito Santo | Santa Teresa | -19.92 | 810 | 9 | CAF | 41651 | 4495 | - | 24 | 0.1194 |
| SLFT2272 | *Boana* | *Boana albomarginata* | Espírito Santo | Santa Teresa | -19.92 | 810 | 9 | CAF | 41651 | 11508 | - | 27 | 0.1123 |
| SLFT2277 | *Boana* | *Boana semilineata* | Espírito Santo | Santa Teresa | -19.92 | 810 | 9 | CAF | 41651 | 10999 | + | 38 | 0.0762 |
| SLFT2281 | *Boana* | *Boana faber* | Espírito Santo | Vargem Alta | -20.48 | 1148 | 10 | CAF | 41653 | 25496 | - | 51 | 0.0580 |
| SLFT2298 | *Boana* | *Boana polytaenia* | Espírito Santo | Vargem Alta | -20.48 | 1148 | 10 | CAF | 41653 | 13837 | - | 28 | 0.1163 |
| SLFT2301 | *Boana* | *Boana pardalis* | Espírito Santo | Vargem Alta | -20.48 | 1148 | 10 | CAF | 41653 | 9393 | + | 28 | 0.1244 |
| SLFT2302 | *Boana* | *Boana faber* | Espírito Santo | Vargem Alta | -20.48 | 1148 | 10 | CAF | 41653 | 16747 | + | 249 | 0.0428 |
| SLFT2308 | *Boana* | *Boana faber* | Espírito Santo | Vargem Alta | -20.48 | 1148 | 10 | CAF | 41653 | 8173 | - | 28 | 0.2229 |
| SLFT2312 | *Boana* | *Boana polytaenia* | Espírito Santo | Vargem Alta | -20.48 | 1148 | 10 | CAF | 41653 | 3545 | - | 18 | 0.2084 |
| SLFT2315 | *Boana* | *Boana polytaenia* | Espírito Santo | Vargem Alta | -20.48 | 1148 | 10 | CAF | 41653 | 2368 | - | 45 | 0.1556 |
| SLFT2332 | *Boana* | *Boana polytaenia* | Espírito Santo | Vargem Alta | -20.48 | 1148 | 10 | CAF | 41653 | 6122 | - | 33 | 0.1404 |
| SLFT2352 | *Boana* | *Boana faber* | Espírito Santo | Vargem Alta | -20.48 | 1148 | 10 | CAF | 41653 | 5138 | + | 88 | 0.0293 |
| SLFT2361 | *Boana* | *Boana polytaenia* | Espírito Santo | Vargem Alta | -20.48 | 1148 | 10 | CAF | 41653 | 10999 | - | 11 | 0.2783 |
| SLFT2362 | *Boana* | *Boana polytaenia* | Espírito Santo | Vargem Alta | -20.48 | 1148 | 10 | CAF | 41653 | 8901 | - | 19 | 0.1282 |
| SLFT2366 | *Boana* | *Boana polytaenia* | Espírito Santo | Vargem Alta | -20.48 | 1148 | 10 | CAF | 41653 | 6374 | NA | 24 | 0.1209 |
| SLFT2846 | *Boana* | *Boana albopunctata* | Rio de Janeiro | Macaé, Barra do Sana | -22.36 | 483 | 11 | CAF | 41654 | 3302 | - | 12 | 0.1781 |
| SLFT2881 | *Boana* | *Boana faber* | Rio de Janeiro | Macaé, Barra do Sana | -22.36 | 483 | 11 | CAF | 41654 | 6252 | - | 10 | 0.2956 |
| SLFT2888 | *Boana* | *Boana albopunctata* | Rio de Janeiro | Macaé, Barra do Sana | -22.36 | 483 | 11 | CAF | 41654 | 10870 | - | 22 | 0.0844 |
| SLFT2889 | *Boana* | *Boana albopunctata* | Rio de Janeiro | Macaé, Barra do Sana | -22.36 | 483 | 11 | CAF | 41654 | 5510 | - | 48 | 0.0506 |
| SLFT2892 | *Boana* | *Boana albopunctata* | Rio de Janeiro | Macaé, Barra do Sana | -22.36 | 483 | 11 | CAF | 41654 | 4533 | - | 142 | 0.0543 |
| SLFT2895 | *Boana* | *Boana albopunctata* | Rio de Janeiro | Macaé, Barra do Sana | -22.36 | 483 | 11 | CAF | 41654 | 8872 | - | 83 | 0.0287 |
| SLFT2901 | *Boana* | *Boana albopunctata* | Rio de Janeiro | Macaé, Barra do Sana | -22.36 | 483 | 11 | CAF | 41654 | 3883 | - | 7 | 0.2112 |
| SLFT2902 | *Boana* | *Boana albopunctata* | Rio de Janeiro | Macaé, Barra do Sana | -22.36 | 483 | 11 | CAF | 41654 | 17101 | - | 26 | 0.1005 |
| SLFT2903 | *Boana* | *Boana faber* | Rio de Janeiro | Macaé, Barra do Sana | -22.36 | 483 | 11 | CAF | 41654 | 9270 | - | 41 | 0.0794 |
| SLFT2905 | *Boana* | *Boana albopunctata* | Rio de Janeiro | Macaé, Barra do Sana | -22.36 | 483 | 11 | CAF | 41654 | 2395 | - | 50 | 0.0337 |
| SLFT2911 | *Boana* | *Boana albopunctata* | Rio de Janeiro | Macaé, Barra do Sana | -22.36 | 483 | 11 | CAF | 41654 | 14585 | - | 27 | 0.0583 |
| SLFT4773 | *Boana* | *Boana* sp. | Rio Grande do Norte | Baía Formosa | -6.41 | 23 | 1 | NAF | 42157 | 105315 | - | 11 | 0.2950 |
| SLFT4797 | *Boana* | *Boana crepitans* | Rio Grande do Norte | Baía Formosa | -6.41 | 23 | 1 | NAF | 42157 | 129817 | - | 44 | 0.0423 |
| SLFT4798 | *Boana* | *Boana crepitans* | Rio Grande do Norte | Baía Formosa | -6.41 | 23 | 1 | NAF | 42157 | 104917 | - | 22 | 0.1524 |
| SLFT4886 | *Boana* | *Boana crepitans* | Rio Grande do Norte | Baía Formosa | -6.41 | 23 | 1 | NAF | 42157 | 2959 | - | 119 | 0.3078 |
| SLFT4895 | *Boana* | *Boana albomarginata* | Paraíba | João Pessoa | -7.12 | 26 | 2 | NAF | 42161 | 31542 | - | 9 | 0.0756 |
| SLFT4926 | *Boana* | *Boana albomarginata* | Paraíba | João Pessoa | -7.12 | 26 | 2 | NAF | 42161 | 77095 | - | 9 | 0.2525 |
| SLFT4966 | *Boana* | *Boana albomarginata* | Paraíba | João Pessoa | -7.12 | 26 | 2 | NAF | 42161 | 21549 | - | 119 | 0.0472 |
| SLFT4983 | *Boana* | *Boana albomarginata* | Paraíba | João Pessoa | -7.12 | 26 | 2 | NAF | 42161 | 71209 | NA | 11 | 0.2311 |
| SLFT4984 | *Boana* | *Boana albomarginata* | Paraíba | João Pessoa | -7.12 | 26 | 2 | NAF | 42161 | 20700 | - | 15 | 0.0884 |
| SLFT5017 | *Boana* | *Boana albomarginata* | Pernambuco | Paulista | -7.93 | 30 | 3 | NAF | 42164 | 88711 | - | 2 | 0.5071 |
| SLFT5027 | *Boana* | *Boana atlantica* | Pernambuco | Paulista | -7.93 | 30 | 3 | NAF | 42164 | 30118 | - | 51 | 0.0813 |
| SLFT5032 | *Boana* | *Boana atlantica* | Pernambuco | Paulista | -7.93 | 30 | 3 | NAF | 42164 | 14508 | - | 110 | 0.0632 |
| SLFT5034 | *Boana* | *Boana atlantica* | Pernambuco | Paulista | -7.93 | 30 | 3 | NAF | 42164 | 23630 | - | 44 | 0.1605 |
| SLFT5044 | *Boana* | *Boana atlantica* | Pernambuco | Paulista | -7.93 | 30 | 3 | NAF | 42164 | 1139 | - | 65 | 0.1195 |
| SLFT5058 | *Boana* | *Boana atlantica* | Pernambuco | Paulista | -7.93 | 30 | 3 | NAF | 42164 | 25720 | - | 346 | 0.1312 |
| SLFT5059 | *Boana* | *Boana atlantica* | Pernambuco | Paulista | -7.93 | 30 | 3 | NAF | 42164 | 6691 | - | 72 | 0.0845 |
| SLFT5062 | *Boana* | *Boana atlantica* | Pernambuco | Paulista | -7.93 | 30 | 3 | NAF | 42164 | 117670 | - | 2 | 0.5020 |
| SLFT5063 | *Boana* | *Boana atlantica* | Pernambuco | Paulista | -7.93 | 30 | 3 | NAF | 42164 | 7481 | - | 4 | 0.1687 |
| SLFT5075 | *Boana* | *Boana atlantica* | Pernambuco | Paulista | -7.93 | 30 | 3 | NAF | 42164 | 81548 | - | 18 | 0.0715 |
| SLFT5077 | *Boana* | *Boana atlantica* | Pernambuco | Paulista | -7.93 | 30 | 3 | NAF | 42164 | 40747 | - | 119 | 0.0671 |
| SLFT5080 | *Boana* | *Boana atlantica* | Pernambuco | Paulista | -7.93 | 30 | 3 | NAF | 42164 | 55414 | - | 26 | 0.0838 |
| SLFT5089 | *Boana* | *Boana atlantica* | Pernambuco | Paulista | -7.93 | 30 | 3 | NAF | 42164 | 30325 | - | 66 | 0.1076 |
| SLFT5091 | *Boana* | *Boana atlantica* | Pernambuco | Paulista | -7.93 | 30 | 3 | NAF | 42164 | 52759 | - | 76 | 0.0640 |
| SLFT5095 | *Boana* | *Boana atlantica* | Pernambuco | Paulista | -7.93 | 30 | 3 | NAF | 42164 | 49508 | - | 45 | 0.1048 |
| SLFT5106 | *Boana* | *Boana albomarginata* | Sergipe | Areia Branca | -10.76 | 373 | 5 | NAF | 42165 | 27160 | - | 189 | 0.1502 |
| SLFT5111 | *Boana* | *Boana albomarginata* | Sergipe | Areia Branca | -10.76 | 373 | 5 | NAF | 42165 | 21467 | - | 124 | 0.1432 |
| SLFT5116 | *Boana* | *Boana albomarginata* | Sergipe | Areia Branca | -10.76 | 373 | 5 | NAF | 42165 | 2154 | - | 28 | 0.1797 |
| SLFT5123 | *Boana* | *Boana albomarginata* | Sergipe | Areia Branca | -10.76 | 373 | 5 | NAF | 42165 | 21331 | - | 50 | 0.1488 |
| SLFT5138 | *Boana* | *Boana albomarginata* | Sergipe | Areia Branca | -10.76 | 373 | 5 | NAF | 42165 | 21322 | - | 266 | 0.2190 |
| SLFT5144 | *Boana* | *Boana albomarginata* | Sergipe | Areia Branca | -10.76 | 373 | 5 | NAF | 42165 | 20006 | - | 29 | 0.1614 |
| SLFT5146 | *Boana* | *Boana semilineata* | Sergipe | Areia Branca | -10.76 | 373 | 5 | NAF | 42165 | 5403 | - | 95 | 0.1663 |
| SLFT5147 | *Boana* | *Boana faber* | Sergipe | Areia Branca | -10.76 | 373 | 5 | NAF | 42165 | 126662 | - | 7 | 0.2515 |
| SLFT5165 | *Boana* | *Boana albomarginata* | Sergipe | Areia Branca | -10.76 | 373 | 5 | NAF | 42165 | 112128 | - | 8 | 0.3360 |
| SLFT5166 | *Boana* | *Boana semilineata* | Sergipe | Areia Branca | -10.76 | 373 | 5 | NAF | 42165 | 43708 | - | 12 | 0.3432 |
| SLFT5176 | *Boana* | *Boana albomarginata* | Sergipe | Areia Branca | -10.76 | 373 | 5 | NAF | 42165 | 36171 | - | 33 | 0.0978 |
| SLFT5205 | *Boana* | *Boana albomarginata* | Sergipe | Areia Branca | -10.76 | 373 | 5 | NAF | 42165 | 13396 | - | 74 | 0.1858 |
| SLFT5241 | *Boana* | *Boana semilineata* | Bahia | Mata de São João | -12.56 | 41 | 6 | NAF | 42168 | 11788 | - | 28 | 0.1443 |
| SLFT5258 | *Boana* | *Boana semilineata* | Bahia | Mata de São João | -12.56 | 41 | 6 | NAF | 42168 | 17223 | - | 150 | 0.1333 |
| SLFT5283 | *Boana* | *Boana semilineata* | Bahia | Mata de São João | -12.56 | 41 | 6 | NAF | 42168 | 4125 | - | 31 | 0.2372 |
| SLFT5286 | *Boana* | *Boana albomarginata* | Bahia | Mata de São João | -12.56 | 41 | 6 | NAF | 42168 | 15215 | - | 182 | 0.2559 |
| SLFT5287 | *Boana* | *Boana albomarginata* | Bahia | Mata de São João | -12.56 | 41 | 6 | NAF | 42168 | 21116 | - | 141 | 0.1188 |
| SLFT5288 | *Boana* | *Boana albomarginata* | Bahia | Mata de São João | -12.56 | 41 | 6 | NAF | 42168 | 17026 | - | 70 | 0.0936 |
| SLFT5290 | *Boana* | *Boana albomarginata* | Bahia | Mata de São João | -12.56 | 41 | 6 | NAF | 42168 | 18412 | - | 153 | 0.1038 |
| SLFT5292 | *Boana* | *Boana albomarginata* | Bahia | Mata de São João | -12.56 | 41 | 6 | NAF | 42168 | 18271 | - | 190 | 0.1304 |
| SLFT5293 | *Boana* | *Boana albomarginata* | Bahia | Mata de São João | -12.56 | 41 | 6 | NAF | 42168 | 36449 | - | 116 | 0.0529 |
| SLFT5295 | *Boana* | *Boana albomarginata* | Bahia | Mata de São João | -12.56 | 41 | 6 | NAF | 42168 | 105276 | - | 7 | 0.1091 |
| SLFT5296 | *Boana* | *Boana albomarginata* | Bahia | Mata de São João | -12.56 | 41 | 6 | NAF | 42168 | 32641 | - | 105 | 0.1149 |
| SLFT5324 | *Boana* | *Boana albomarginata* | Bahia | Mata de São João | -12.56 | 41 | 6 | NAF | 42168 | 85704 | - | 2 | 0.3476 |
| SLFT0136 | *Bokermannohyla* | *Bokermannohyla hylax* | Paraná | Morretes | -25.40 | 426 | 19 | SAF | 41283 | 28667 | - | 86 | 0.0700 |
| SLFT1555 | *Bokermannohyla* | *Bokermannohyla* sp. | Rio de Janeiro | Teresópolis | -22.46 | 1487 | 12 | CAF | 41411 | 8597 | + | 10 | 0.1003 |
| SLFT1573 | *Bokermannohyla* | *Bokermannohyla* sp*.* | Rio de Janeiro | Teresópolis | -22.46 | 1487 | 12 | CAF | 41411 | 13759 | + | 28 | 0.0294 |
| SLFT1574 | *Bokermannohyla* | *Bokermannohyla* sp. | Rio de Janeiro | Teresópolis | -22.46 | 1487 | 12 | CAF | 41411 | 4795 | - | 85 | 0.0700 |
| SLFT1577 | *Bokermannohyla* | *Bokermannohyla circumdata* | Rio de Janeiro | Teresópolis | -22.46 | 1487 | 12 | CAF | 41411 | 2870 | - | 198 | 0.2030 |
| SLFT1599 | *Bokermannohyla* | *Bokermannohyla* sp*.* | Rio de Janeiro | Teresópolis | -22.46 | 1487 | 12 | CAF | 41411 | 16823 | - | 8 | 0.1258 |
| SLFT2009 | *Bokermannohyla* | *Bokermannohyla circumdata* | Bahia | Camacan | -15.38 | 376 | 7 | NAF | 41647 | 11826 | - | 16 | 0.1909 |
| SLFT5185 | *Corythomantis* | *Corythomantis greeningi* | Sergipe | Areia Branca | -10.76 | 373 | 5 | NAF | 42165 | 32085 | - | 36 | 0.1149 |
| SLFT0118 | *Dendropsophus* | *Dendropsophus elegans* | São Paulo | Iguape | -24.50 | 16 | 17 | CAF | 2013 | 62764 | + | 108 | 0.0306 |
| SLFT0121 | *Dendropsophus* | *Dendropsophus microps* | São Paulo | Iguape | -24.50 | 16 | 17 | CAF | 2013 | 62764 | - | 112 | 0.1490 |
| SLFT0123 | *Dendropsophus* | *Dendropsophus minutus* | São Paulo | Iguape | -24.50 | 16 | 17 | CAF | 2013 | 62764 | - | 62 | 0.0264 |
| SLFT0517 | *Dendropsophus* | *Dendropsophus elegans* | São Paulo | Iporanga | -24.60 | 412 | 18 | CAF | 41281 | 77543 | - | 13 | 0.1106 |
| SLFT0518 | *Dendropsophus* | *Dendropsophus elegans* | São Paulo | Iporanga | -24.60 | 412 | 18 | CAF | 41281 | 22038 | - | 147 | 0.1171 |
| SLFT0519 | *Dendropsophus* | *Dendropsophus elegans* | São Paulo | Iporanga | -24.60 | 412 | 18 | CAF | 41281 | 19754 | - | 34 | 0.2610 |
| SLFT0520 | *Dendropsophus* | *Dendropsophus elegans* | São Paulo | Iporanga | -24.60 | 412 | 18 | CAF | 41281 | 18561 | - | 63 | 0.0864 |
| SLFT0522 | *Dendropsophus* | *Dendropsophus elegans* | São Paulo | Iporanga | -24.60 | 412 | 18 | CAF | 41281 | 68249 | - | 25 | 0.0802 |
| SLFT0524 | *Dendropsophus* | *Dendropsophus elegans* | São Paulo | Iporanga | -24.60 | 412 | 18 | CAF | 41281 | 14000 | - | 42 | 0.0896 |
| SLFT0536 | *Dendropsophus* | *Dendropsophus elegans* | São Paulo | Iporanga | -24.60 | 412 | 18 | CAF | 41281 | 31965 | - | 35 | 0.0583 |
| SLFT0548 | *Dendropsophus* | *Dendropsophus elegans* | São Paulo | Iporanga | -24.60 | 412 | 18 | CAF | 41281 | 8904 | + | 38 | 0.0611 |
| SLFT0550 | *Dendropsophus* | *Dendropsophus elegans* | São Paulo | Iporanga | -24.60 | 412 | 18 | CAF | 41281 | 27179 | - | 37 | 0.0673 |
| SLFT0551 | *Dendropsophus* | *Dendropsophus elegans* | São Paulo | Iporanga | -24.60 | 412 | 18 | CAF | 41281 | 53507 | - | 16 | 0.0798 |
| SLFT0561 | *Dendropsophus* | *Dendropsophus elegans* | São Paulo | Iporanga | -24.60 | 412 | 18 | CAF | 41281 | 33670 | + | 42 | 0.1687 |
| SLFT0570 | *Dendropsophus* | *Dendropsophus elegans* | São Paulo | Iporanga | -24.60 | 412 | 18 | CAF | 41281 | 27699 | - | 33 | 0.1077 |
| SLFT0571 | *Dendropsophus* | *Dendropsophus elegans* | São Paulo | Iporanga | -24.60 | 412 | 18 | CAF | 41281 | 5166 | - | 73 | 0.0821 |
| SLFT0575 | *Dendropsophus* | *Dendropsophus elegans* | São Paulo | Iporanga | -24.60 | 412 | 18 | CAF | 41281 | 55502 | - | 33 | 0.1210 |
| SLFT0576 | *Dendropsophus* | *Dendropsophus elegans* | São Paulo | Iporanga | -24.60 | 412 | 18 | CAF | 41281 | 4715 | - | 75 | 0.0580 |
| SLFT0579 | *Dendropsophus* | *Dendropsophus werneri* | São Paulo | Iporanga | -24.60 | 412 | 18 | CAF | 41281 | 5892 | - | 29 | 0.0750 |
| SLFT0581 | *Dendropsophus* | *Dendropsophus elegans* | São Paulo | Iporanga | -24.60 | 412 | 18 | CAF | 41281 | 6911 | - | 27 | 0.0805 |
| SLFT0583 | *Dendropsophus* | *Dendropsophus elegans* | São Paulo | Iporanga | -24.60 | 412 | 18 | CAF | 41281 | 7224 | - | 35 | 0.0526 |
| SLFT0585 | *Dendropsophus* | *Dendropsophus elegans* | São Paulo | Iporanga | -24.60 | 412 | 18 | CAF | 41281 | 64496 | - | 14 | 0.2131 |
| SLFT0588 | *Dendropsophus* | *Dendropsophus elegans* | São Paulo | Iporanga | -24.60 | 412 | 18 | CAF | 41281 | 17760 | - | 28 | 0.1605 |
| SLFT0589 | *Dendropsophus* | *Dendropsophus elegans* | São Paulo | Iporanga | -24.60 | 412 | 18 | CAF | 41281 | 9523 | - | 41 | 0.1250 |
| SLFT0590 | *Dendropsophus* | *Dendropsophus elegans* | São Paulo | Iporanga | -24.60 | 412 | 18 | CAF | 41281 | 33335 | - | 14 | 0.1132 |
| SLFT0596 | *Dendropsophus* | *Dendropsophus elegans* | São Paulo | Iporanga | -24.60 | 412 | 18 | CAF | 41281 | 27149 | - | 68 | 0.0375 |
| SLFT0600 | *Dendropsophus* | *Dendropsophus elegans* | São Paulo | Iporanga | -24.60 | 412 | 18 | CAF | 41281 | 3859 | - | 86 | 0.0843 |
| SLFT0602 | *Dendropsophus* | *Dendropsophus elegans* | São Paulo | Iporanga | -24.60 | 412 | 18 | CAF | 41281 | 47908 | - | 32 | 0.0775 |
| SLFT0603 | *Dendropsophus* | *Dendropsophus elegans* | São Paulo | Iporanga | -24.60 | 412 | 18 | CAF | 41281 | 9531 | - | 21 | 0.1113 |
| SLFT0604 | *Dendropsophus* | *Dendropsophus elegans* | São Paulo | Iporanga | -24.60 | 412 | 18 | CAF | 41281 | 19729 | - | 35 | 0.0420 |
| SLFT0605 | *Dendropsophus* | *Dendropsophus elegans* | São Paulo | Iporanga | -24.60 | 412 | 18 | CAF | 41281 | 12159 | - | 62 | 0.0491 |
| SLFT0606 | *Dendropsophus* | *Dendropsophus elegans* | São Paulo | Iporanga | -24.60 | 412 | 18 | CAF | 41281 | 20995 | - | 25 | 0.1002 |
| SLFT0613 | *Dendropsophus* | *Dendropsophus werneri* | São Paulo | Iporanga | -24.60 | 412 | 18 | CAF | 41281 | 57861 | + | 33 | 0.1144 |
| SLFT0615 | *Dendropsophus* | *Dendropsophus werneri* | São Paulo | Iporanga | -24.60 | 412 | 18 | CAF | 41281 | 19999 | - | 33 | 0.0831 |
| SLFT0617 | *Dendropsophus* | *Dendropsophus elegans* | São Paulo | Iporanga | -24.60 | 412 | 18 | CAF | 41281 | 3032 | + | 71 | 0.0415 |
| SLFT0618 | *Dendropsophus* | *Dendropsophus elegans* | São Paulo | Iporanga | -24.60 | 412 | 18 | CAF | 41281 | 38660 | - | 27 | 0.1042 |
| SLFT0619 | *Dendropsophus* | *Dendropsophus werneri* | São Paulo | Iporanga | -24.60 | 412 | 18 | CAF | 41281 | 27043 | - | 44 | 0.0465 |
| SLFT0624 | *Dendropsophus* | *Dendropsophus elegans* | São Paulo | Iporanga | -24.60 | 412 | 18 | CAF | 41281 | 51853 | + | 26 | 0.0766 |
| SLFT0628 | *Dendropsophus* | *Dendropsophus elegans* | São Paulo | Iporanga | -24.60 | 412 | 18 | CAF | 41281 | 29969 | + | 50 | 0.1661 |
| SLFT0629 | *Dendropsophus* | *Dendropsophus elegans* | São Paulo | Iporanga | -24.60 | 412 | 18 | CAF | 41281 | 27718 | - | 43 | 0.1237 |
| SLFT0631 | *Dendropsophus* | *Dendropsophus elegans* | São Paulo | Iporanga | -24.60 | 412 | 18 | CAF | 41281 | 6959 | + | 92 | 0.0617 |
| SLFT0634 | *Dendropsophus* | *Dendropsophus elegans* | São Paulo | Iporanga | -24.60 | 412 | 18 | CAF | 41281 | 10495 | - | 55 | 0.0765 |
| SLFT0636 | *Dendropsophus* | *Dendropsophus elegans* | São Paulo | Iporanga | -24.60 | 412 | 18 | CAF | 41281 | 11358 | NA | 93 | 0.0390 |
| SLFT0638 | *Dendropsophus* | *Dendropsophus werneri* | São Paulo | Iporanga | -24.60 | 412 | 18 | CAF | 41281 | 37416 | - | 23 | 0.2356 |
| SLFT0639 | *Dendropsophus* | *Dendropsophus elegans* | São Paulo | Iporanga | -24.60 | 412 | 18 | CAF | 41281 | 11960 | - | 24 | 0.0725 |
| SLFT0662 | *Dendropsophus* | *Dendropsophus elegans* | Paraná | Morretes | -25.40 | 426 | 19 | SAF | 41283 | 94247 | - | 14 | 0.1228 |
| SLFT0665 | *Dendropsophus* | *Dendropsophus elegans* | Paraná | Morretes | -25.40 | 426 | 19 | SAF | 41283 | 1290 | - | 50 | 0.0316 |
| SLFT0672 | *Dendropsophus* | *Dendropsophus elegans* | Paraná | Morretes | -25.40 | 426 | 19 | SAF | 41283 | 1364 | - | 34 | 0.1168 |
| SLFT0673 | *Dendropsophus* | *Dendropsophus minutus* | Paraná | Morretes | -25.40 | 426 | 19 | SAF | 41283 | 30162 | NA | 23 | 0.1568 |
| SLFT0674 | *Dendropsophus* | *Dendropsophus elegans* | Paraná | Morretes | -25.40 | 426 | 19 | SAF | 41283 | 1741 | + | 35 | 0.1002 |
| SLFT0676 | *Dendropsophus* | *Dendropsophus elegans* | Paraná | Morretes | -25.40 | 426 | 19 | SAF | 41283 | 1415 | + | 24 | 0.0861 |
| SLFT0677 | *Dendropsophus* | *Dendropsophus elegans* | Paraná | Morretes | -25.40 | 426 | 19 | SAF | 41283 | 32300 | + | 26 | 0.1667 |
| SLFT0679 | *Dendropsophus* | *Dendropsophus elegans* | Paraná | Morretes | -25.40 | 426 | 19 | SAF | 41283 | 2236 | - | 34 | 0.1409 |
| SLFT0682 | *Dendropsophus* | *Dendropsophus elegans* | Paraná | Morretes | -25.40 | 426 | 19 | SAF | 41283 | 27372 | + | 16 | 0.2638 |
| SLFT0683 | *Dendropsophus* | *Dendropsophus elegans* | Paraná | Morretes | -25.40 | 426 | 19 | SAF | 41283 | 11834 | + | 27 | 0.1751 |
| SLFT0684 | *Dendropsophus* | *Dendropsophus minutus* | Paraná | Morretes | -25.40 | 426 | 19 | SAF | 41283 | 55198 | + | 20 | 0.1017 |
| SLFT0686 | *Dendropsophus* | *Dendropsophus minutus* | Paraná | Morretes | -25.40 | 426 | 19 | SAF | 41283 | 29796 | - | 12 | 0.1571 |
| SLFT0688 | *Dendropsophus* | *Dendropsophus elegans* | Paraná | Morretes | -25.40 | 426 | 19 | SAF | 41283 | 45912 | + | 17 | 0.0775 |
| SLFT0690 | *Dendropsophus* | *Dendropsophus elegans* | Paraná | Morretes | -25.40 | 426 | 19 | SAF | 41283 | 39423 | + | 29 | 0.1852 |
| SLFT0691 | *Dendropsophus* | *Dendropsophus elegans* | Paraná | Morretes | -25.40 | 426 | 19 | SAF | 41283 | 34707 | + | 26 | 0.2077 |
| SLFT0692 | *Dendropsophus* | *Dendropsophus elegans* | Paraná | Morretes | -25.40 | 426 | 19 | SAF | 41283 | 24441 | - | 24 | 0.1356 |
| SLFT0693 | *Dendropsophus* | *Dendropsophus elegans* | Paraná | Morretes | -25.40 | 426 | 19 | SAF | 41283 | 4572 | + | 20 | 0.1002 |
| SLFT0694 | *Dendropsophus* | *Dendropsophus minutus* | Paraná | Morretes | -25.40 | 426 | 19 | SAF | 41283 | 15009 | + | 25 | 0.3121 |
| SLFT0695 | *Dendropsophus* | *Dendropsophus minutus* | Paraná | Morretes | -25.40 | 426 | 19 | SAF | 41283 | 22809 | - | 73 | 0.0758 |
| SLFT0696 | *Dendropsophus* | *Dendropsophus elegans* | Paraná | Morretes | -25.40 | 426 | 19 | SAF | 41283 | 68431 | + | 14 | 0.1717 |
| SLFT0697 | *Dendropsophus* | *Dendropsophus elegans* | Paraná | Morretes | -25.40 | 426 | 19 | SAF | 41283 | 11351 | - | 21 | 0.1015 |
| SLFT0698 | *Dendropsophus* | *Dendropsophus elegans* | Paraná | Morretes | -25.40 | 426 | 19 | SAF | 41283 | 8938 | + | 37 | 0.1807 |
| SLFT0699 | *Dendropsophus* | *Dendropsophus elegans* | Paraná | Morretes | -25.40 | 426 | 19 | SAF | 41283 | 30542 | + | 11 | 0.3222 |
| SLFT0701 | *Dendropsophus* | *Dendropsophus elegans* | Paraná | Morretes | -25.40 | 426 | 19 | SAF | 41283 | 3572 | + | 23 | 0.0582 |
| SLFT0702 | *Dendropsophus* | *Dendropsophus elegans* | Paraná | Morretes | -25.40 | 426 | 19 | SAF | 41283 | 49292 | + | 16 | 0.2214 |
| SLFT0703 | *Dendropsophus* | *Dendropsophus elegans* | Paraná | Morretes | -25.40 | 426 | 19 | SAF | 41283 | 25614 | - | 50 | 0.1902 |
| SLFT0705 | *Dendropsophus* | *Dendropsophus elegans* | Paraná | Morretes | -25.40 | 426 | 19 | SAF | 41283 | 3272 | + | 30 | 0.0920 |
| SLFT0707 | *Dendropsophus* | *Dendropsophus elegans* | Paraná | Morretes | -25.40 | 426 | 19 | SAF | 41283 | 27138 | + | 27 | 0.2392 |
| SLFT0708 | *Dendropsophus* | *Dendropsophus minutus* | Paraná | Morretes | -25.40 | 426 | 19 | SAF | 41283 | 33608 | NA | 9 | 0.2345 |
| SLFT0710 | *Dendropsophus* | *Dendropsophus elegans* | Paraná | Morretes | -25.40 | 426 | 19 | SAF | 41283 | 59574 | - | 21 | 0.0804 |
| SLFT0713 | *Dendropsophus* | *Dendropsophus elegans* | Paraná | Morretes | -25.40 | 426 | 19 | SAF | 41283 | 2907 | - | 20 | 0.1673 |
| SLFT0714 | *Dendropsophus* | *Dendropsophus minutus* | Paraná | Morretes | -25.40 | 426 | 19 | SAF | 41283 | 6053 | + | 25 | 0.2144 |
| SLFT0716 | *Dendropsophus* | *Dendropsophus elegans* | Paraná | Morretes | -25.40 | 426 | 19 | SAF | 41283 | 73553 | + | 17 | 0.0810 |
| SLFT0717 | *Dendropsophus* | *Dendropsophus elegans* | Paraná | Morretes | -25.40 | 426 | 19 | SAF | 41283 | 42529 | + | 12 | 0.1278 |
| SLFT0719 | *Dendropsophus* | *Dendropsophus elegans* | Paraná | Morretes | -25.40 | 426 | 19 | SAF | 41283 | 24710 | + | 11 | 0.1524 |
| SLFT0721 | *Dendropsophus* | *Dendropsophus elegans* | Paraná | Morretes | -25.40 | 426 | 19 | SAF | 41283 | 42880 | + | 11 | 0.1498 |
| SLFT0724 | *Dendropsophus* | *Dendropsophus elegans* | Paraná | Morretes | -25.40 | 426 | 19 | SAF | 41283 | 20342 | NA | 12 | 0.2174 |
| SLFT0729 | *Dendropsophus* | *Dendropsophus elegans* | Paraná | Morretes | -25.40 | 426 | 19 | SAF | 41283 | 3314 | - | 36 | 0.0979 |
| SLFT0732 | *Dendropsophus* | *Dendropsophus elegans* | Paraná | Morretes | -25.40 | 426 | 19 | SAF | 41283 | 16540 | + | 8 | 0.3814 |
| SLFT0733 | *Dendropsophus* | *Dendropsophus elegans* | Paraná | Morretes | -25.40 | 426 | 19 | SAF | 41283 | 20477 | - | 14 | 0.3321 |
| SLFT0735 | *Dendropsophus* | *Dendropsophus elegans* | Paraná | Morretes | -25.40 | 426 | 19 | SAF | 41283 | 13729 | + | 13 | 0.2105 |
| SLFT0739 | *Dendropsophus* | *Dendropsophus elegans* | Paraná | Morretes | -25.40 | 426 | 19 | SAF | 41283 | 1671 | + | 20 | 0.1140 |
| SLFT0750 | *Dendropsophus* | *Dendropsophus minutus* | Santa Catarina | Rancho Queimado | -27.67 | 959 | 22 | SAF | 41284 | 11471 | - | 182 | 0.0490 |
| SLFT0755 | *Dendropsophus* | *Dendropsophus minutus* | Santa Catarina | Rancho Queimado | -27.67 | 959 | 22 | SAF | 41284 | 1393 | - | 102 | 0.1040 |
| SLFT0761 | *Dendropsophus* | *Dendropsophus minutus* | Santa Catarina | Rancho Queimado | -27.67 | 959 | 22 | SAF | 41284 | 5782 | - | 44 | 0.0677 |
| SLFT0762 | *Dendropsophus* | *Dendropsophus minutus* | Santa Catarina | Rancho Queimado | -27.67 | 959 | 22 | SAF | 41284 | 3674 | - | 46 | 0.0739 |
| SLFT0769 | *Dendropsophus* | *Dendropsophus minutus* | Santa Catarina | Rancho Queimado | -27.67 | 959 | 22 | SAF | 41284 | 1992 | - | 144 | 0.1193 |
| SLFT0791 | *Dendropsophus* | *Dendropsophus minutus* | Santa Catarina | Rancho Queimado | -27.67 | 959 | 22 | SAF | 41284 | 1318 | - | 24 | 0.1239 |
| SLFT0797 | *Dendropsophus* | *Dendropsophus minutus* | Santa Catarina | Rancho Queimado | -27.67 | 959 | 22 | SAF | 41284 | 13479 | - | 237 | 0.0411 |
| SLFT0815 | *Dendropsophus* | *Dendropsophus minutus* | Santa Catarina | Rancho Queimado | -27.67 | 959 | 22 | SAF | 41284 | 29865 | + | 160 | 0.0327 |
| SLFT0833 | *Dendropsophus* | *Dendropsophus minutus* | Santa Catarina | Rancho Queimado | -27.67 | 959 | 22 | SAF | 41284 | 7469 | + | 340 | 0.1152 |
| SLFT0834 | *Dendropsophus* | *Dendropsophus minutus* | Santa Catarina | Rancho Queimado | -27.67 | 959 | 22 | SAF | 41284 | 1223 | - | 238 | 0.0962 |
| SLFT0836 | *Dendropsophus* | *Dendropsophus minutus* | Santa Catarina | Rancho Queimado | -27.67 | 959 | 22 | SAF | 41284 | 12421 | - | 61 | 0.0408 |
| SLFT0846 | *Dendropsophus* | *Dendropsophus minutus* | Santa Catarina | Rancho Queimado | -27.67 | 959 | 22 | SAF | 41284 | 35630 | - | 6 | 0.3925 |
| SLFT1034 | *Dendropsophus* | *Dendropsophus minutus* | Rio de Janeiro | Teresópolis | -22.46 | 1487 | 12 | CAF | 41635 | 35765 | - | 95 | 0.0766 |
| SLFT1035 | *Dendropsophus* | *Dendropsophus minutus* | Rio de Janeiro | Teresópolis | -22.46 | 1487 | 12 | CAF | 41635 | 54912 | + | 43 | 0.0324 |
| SLFT1036 | *Dendropsophus* | *Dendropsophus minutus* | Rio de Janeiro | Teresópolis | -22.46 | 1487 | 12 | CAF | 41635 | 32701 | - | 52 | 0.0422 |
| SLFT1051 | *Dendropsophus* | *Dendropsophus minutus* | Rio de Janeiro | Teresópolis | -22.46 | 1487 | 12 | CAF | 41635 | 32546 | - | 37 | 0.0536 |
| SLFT1097 | *Dendropsophus* | *Dendropsophus berthalutzae* | São Paulo | Pedro de Toledo | -24.32 | 209 | 16 | CAF | 41328 | 27219 | - | 38 | 0.0648 |
| SLFT1098 | *Dendropsophus* | *Dendropsophus berthalutzae* | São Paulo | Pedro de Toledo | -24.32 | 209 | 16 | CAF | 41328 | 23047 | - | 45 | 0.0552 |
| SLFT1099 | *Dendropsophus* | *Dendropsophus werneri* | São Paulo | Itariri | -24.31 | 180 | 15 | CAF | 41329 | 54505 | - | 75 | 0.0566 |
| SLFT1102 | *Dendropsophus* | *Dendropsophus werneri* | São Paulo | Iguape | -24.50 | 16 | 17 | CAF | 2013 | 16806 | - | 61 | 0.0938 |
| SLFT1103 | *Dendropsophus* | *Dendropsophus elegans* | São Paulo | Iguape | -24.50 | 16 | 17 | CAF | 2013 | 8554 | - | 60 | 0.1261 |
| SLFT1104 | *Dendropsophus* | *Dendropsophus elegans* | São Paulo | Iguape | -24.50 | 16 | 17 | CAF | 2013 | 13788 | - | 53 | 0.0651 |
| SLFT1105 | *Dendropsophus* | *Dendropsophus elegans* | São Paulo | Iguape | -24.50 | 16 | 17 | CAF | 2013 | 31882 | - | 37 | 0.0613 |
| SLFT1107 | *Dendropsophus* | *Dendropsophus elegans* | São Paulo | Iguape | -24.50 | 16 | 17 | CAF | 2013 | 50581 | - | 41 | 0.0732 |
| SLFT1108 | *Dendropsophus* | *Dendropsophus elegans* | São Paulo | Iguape | -24.50 | 16 | 17 | CAF | 2013 | 57555 | - | 48 | 0.0388 |
| SLFT1109 | *Dendropsophus* | *Dendropsophus elegans* | São Paulo | Iguape | -24.50 | 16 | 17 | CAF | 2013 | 62764 | - | 51 | 0.0802 |
| SLFT1265 | *Dendropsophus* | *Dendropsophus minutus* | Santa Catarina | São Miguel d' Oeste | -26.76 | 571 | 20 | SAF | 41197 | 10443 | - | 136 | 0.0783 |
| SLFT1279 | *Dendropsophus* | *Dendropsophus minutus* | Santa Catarina | São Miguel d' Oeste | -26.76 | 571 | 20 | SAF | 41197 | 3657 | - | 174 | 0.1545 |
| SLFT1302 | *Dendropsophus* | *Dendropsophus minutus* | Santa Catarina | São Miguel d' Oeste | -26.76 | 571 | 20 | SAF | 41197 | 9408 | - | 114 | 0.0323 |
| SLFT1303 | *Dendropsophus* | *Dendropsophus minutus* | Santa Catarina | São Miguel d' Oeste | -26.76 | 571 | 20 | SAF | 41197 | 17869 | - | 41 | 0.0263 |
| SLFT1304 | *Dendropsophus* | *Dendropsophus minutus* | Santa Catarina | São Miguel d' Oeste | -26.76 | 571 | 20 | SAF | 41197 | 13114 | - | 113 | 0.0167 |
| SLFT1307 | *Dendropsophus* | *Dendropsophus minutus* | Santa Catarina | São Miguel d' Oeste | -26.76 | 571 | 20 | SAF | 41197 | 8744 | - | 200 | 0.0330 |
| SLFT1309 | *Dendropsophus* | *Dendropsophus minutus* | Santa Catarina | São Miguel d' Oeste | -26.76 | 571 | 20 | SAF | 41197 | 6032 | - | 79 | 0.0376 |
| SLFT1320 | *Dendropsophus* | *Dendropsophus minutus* | Santa Catarina | São Miguel d' Oeste | -26.76 | 571 | 20 | SAF | 41197 | 2314 | - | 132 | 0.0781 |
| SLFT1379 | *Dendropsophus* | *Dendropsophus minutus* | Santa Catarina | São Miguel d' Oeste | -26.76 | 571 | 20 | SAF | 41197 | 10729 | NA | 228 | 0.0466 |
| SLFT1402 | *Dendropsophus* | *Dendropsophus minutus* | Santa Catarina | São Miguel d' Oeste | -26.76 | 571 | 20 | SAF | 41197 | 22243 | NA | 202 | 0.0274 |
| SLFT1404 | *Dendropsophus* | *Dendropsophus minutus* | Santa Catarina | São Miguel d' Oeste | -26.76 | 571 | 20 | SAF | 41197 | 1790 | NA | 81 | 0.0259 |
| SLFT1455 | *Dendropsophus* | *Dendropsophus elegans* | São Paulo | Itariri | -24.31 | 180 | 15 | CAF | 41329 | 10472 | - | 30 | 0.1566 |
| SLFT1456 | *Dendropsophus* | *Dendropsophus elegans* | São Paulo | Itariri | -24.31 | 180 | 15 | CAF | 41329 | 11754 | - | 41 | 0.1454 |
| SLFT1459 | *Dendropsophus* | *Dendropsophus elegans* | São Paulo | Itariri | -24.31 | 180 | 15 | CAF | 41329 | 9482 | - | 51 | 0.1007 |
| SLFT1461 | *Dendropsophus* | *Dendropsophus elegans* | São Paulo | Itariri | -24.31 | 180 | 15 | CAF | 41329 | 1650 | - | 45 | 0.1798 |
| SLFT1462 | *Dendropsophus* | *Dendropsophus elegans* | São Paulo | Itariri | -24.31 | 180 | 15 | CAF | 41329 | 17794 | - | 43 | 0.1249 |
| SLFT1463 | *Dendropsophus* | *Dendropsophus elegans* | São Paulo | Itariri | -24.31 | 180 | 15 | CAF | 41329 | 3953 | - | 76 | 0.1410 |
| SLFT1464 | *Dendropsophus* | *Dendropsophus elegans* | São Paulo | Itariri | -24.31 | 180 | 15 | CAF | 41329 | 4532 | - | 42 | 0.0815 |
| SLFT1465 | *Dendropsophus* | *Dendropsophus elegans* | São Paulo | Itariri | -24.31 | 180 | 15 | CAF | 41329 | 17073 | + | 11 | 0.2629 |
| SLFT1466 | *Dendropsophus* | *Dendropsophus elegans* | São Paulo | Itariri | -24.31 | 180 | 15 | CAF | 41329 | 5305 | - | 36 | 0.0714 |
| SLFT1467 | *Dendropsophus* | *Dendropsophus elegans* | São Paulo | Itariri | -24.31 | 180 | 15 | CAF | 41329 | 30285 | - | 52 | 0.0764 |
| SLFT1468 | *Dendropsophus* | *Dendropsophus elegans* | São Paulo | Itariri | -24.31 | 180 | 15 | CAF | 41329 | 34144 | - | 43 | 0.1082 |
| SLFT1469 | *Dendropsophus* | *Dendropsophus elegans* | São Paulo | Itariri | -24.31 | 180 | 15 | CAF | 41329 | 50636 | - | 29 | 0.0967 |
| SLFT1470 | *Dendropsophus* | *Dendropsophus elegans* | São Paulo | Itariri | -24.31 | 180 | 15 | CAF | 41329 | 42888 | - | 44 | 0.1248 |
| SLFT1471 | *Dendropsophus* | *Dendropsophus elegans* | São Paulo | Itariri | -24.31 | 180 | 15 | CAF | 41329 | 52884 | - | 30 | 0.0854 |
| SLFT1472 | *Dendropsophus* | *Dendropsophus elegans* | São Paulo | Itariri | -24.31 | 180 | 15 | CAF | 41329 | 41593 | - | 54 | 0.1534 |
| SLFT1473 | *Dendropsophus* | *Dendropsophus elegans* | São Paulo | Itariri | -24.31 | 180 | 15 | CAF | 41329 | 29662 | - | 56 | 0.1715 |
| SLFT1490 | *Dendropsophus* | *Dendropsophus elegans* | São Paulo | Pedro de Toledo | -24.32 | 209 | 16 | CAF | 41328 | 43935 | - | 53 | 0.1040 |
| SLFT1491 | *Dendropsophus* | *Dendropsophus elegans* | São Paulo | Pedro de Toledo | -24.32 | 209 | 16 | CAF | 41328 | 13866 | - | 50 | 0.0467 |
| SLFT1492 | *Dendropsophus* | *Dendropsophus elegans* | São Paulo | Pedro de Toledo | -24.32 | 209 | 16 | CAF | 41328 | 20069 | - | 17 | 0.1920 |
| SLFT1493 | *Dendropsophus* | *Dendropsophus elegans* | São Paulo | Pedro de Toledo | -24.32 | 209 | 16 | CAF | 41328 | 47194 | NA | 39 | 0.0914 |
| SLFT1494 | *Dendropsophus* | *Dendropsophus elegans* | São Paulo | Pedro de Toledo | -24.32 | 209 | 16 | CAF | 41328 | 2225 | - | 26 | 0.0551 |
| SLFT1495 | *Dendropsophus* | *Dendropsophus werneri* | São Paulo | Pedro de Toledo | -24.32 | 209 | 16 | CAF | 41328 | 22425 | - | 33 | 0.1550 |
| SLFT1496 | *Dendropsophus* | *Dendropsophus elegans* | São Paulo | Pedro de Toledo | -24.32 | 209 | 16 | CAF | 41328 | 7403 | - | 42 | 0.1500 |
| SLFT1500 | *Dendropsophus* | *Dendropsophus* sp*.* | Rio Grande do Sul | Torres | -29.33 | 9 | 23 | SAF | 41356 | 20639 | + | 625 | 0.6366 |
| SLFT1501 | *Dendropsophus* | *Dendropsophus* sp*.* | Rio Grande do Sul | Torres | -29.33 | 9 | 23 | SAF | 41356 | 30083 | - | 422 | 0.1698 |
| SLFT1503 | *Dendropsophus* | *Dendropsophus* sp. | Rio Grande do Sul | Torres | -29.33 | 9 | 23 | SAF | 41356 | 22540 | + | 562 | 0.4325 |
| SLFT1506 | *Dendropsophus* | *Dendropsophus* sp. | Rio Grande do Sul | Torres | -29.33 | 9 | 23 | SAF | 41356 | 64781 | - | 5 | 0.2298 |
| SLFT1561 | *Dendropsophus* | *Dendropsophus minutus* | Rio de Janeiro | Teresópolis | -22.46 | 1487 | 12 | CAF | 41411 | 2496 | + | 168 | 0.0800 |
| SLFT1914 | *Dendropsophus* | *Dendropsophus minutus* | Alagoas | Murici | -9.21 | 233 | 4 | NAF | 41520 | 3183 | - | 83 | 0.0963 |
| SLFT1915 | *Dendropsophus* | *Dendropsophus minutus* | Alagoas | Murici | -9.21 | 233 | 4 | NAF | 41520 | 2737 | + | 102 | 0.0945 |
| SLFT1952 | *Dendropsophus* | *Dendropsophus elegans* | Bahia | Camacan | -15.38 | 376 | 7 | NAF | 41647 | 26750 | - | 22 | 0.0567 |
| SLFT1955 | *Dendropsophus* | *Dendropsophus minutus* | Bahia | Camacan | -15.38 | 376 | 7 | NAF | 41647 | 4146 | - | 21 | 0.0868 |
| SLFT1956 | *Dendropsophus* | *Dendropsophus haddadi* | Bahia | Camacan | -15.38 | 376 | 7 | NAF | 41647 | 16222 | - | 22 | 0.1054 |
| SLFT1958 | *Dendropsophus* | *Dendropsophus minutus* | Bahia | Camacan | -15.38 | 376 | 7 | NAF | 41647 | 4820 | - | 18 | 0.0607 |
| SLFT1960 | *Dendropsophus* | *Dendropsophus bipunctatus* | Bahia | Camacan | -15.38 | 376 | 7 | NAF | 41647 | 1902 | - | 31 | 0.0946 |
| SLFT1968 | *Dendropsophus* | *Dendropsophus elegans* | Bahia | Camacan | -15.38 | 376 | 7 | NAF | 41647 | 14199 | - | 35 | 0.1470 |
| SLFT1971 | *Dendropsophus* | *Dendropsophus haddadi* | Bahia | Camacan | -15.38 | 376 | 7 | NAF | 41647 | 41056 | - | 23 | 0.1393 |
| SLFT1973 | *Dendropsophus* | *Dendropsophus oliveirai* | Bahia | Camacan | -15.38 | 376 | 7 | NAF | 41647 | 17794 | - | 46 | 0.1113 |
| SLFT1974 | *Dendropsophus* | *Dendropsophus elegans* | Bahia | Camacan | -15.38 | 376 | 7 | NAF | 41647 | 12962 | - | 56 | 0.0912 |
| SLFT1975 | *Dendropsophus* | *Dendropsophus minutus* | Bahia | Camacan | -15.38 | 376 | 7 | NAF | 41647 | 26900 | - | 51 | 0.1202 |
| SLFT1976 | *Dendropsophus* | *Dendropsophus elegans* | Bahia | Camacan | -15.38 | 376 | 7 | NAF | 41647 | 7734 | - | 25 | 0.0906 |
| SLFT1977 | *Dendropsophus* | *Dendropsophus haddadi* | Bahia | Camacan | -15.38 | 376 | 7 | NAF | 41647 | 2573 | - | 31 | 0.1664 |
| SLFT1978 | *Dendropsophus* | *Dendropsophus haddadi* | Bahia | Camacan | -15.38 | 376 | 7 | NAF | 41647 | 4450 | - | 33 | 0.1678 |
| SLFT1979 | *Dendropsophus* | *Dendropsophus elegans* | Bahia | Camacan | -15.38 | 376 | 7 | NAF | 41647 | 15749 | - | 47 | 0.1046 |
| SLFT1981 | *Dendropsophus* | *Dendropsophus elegans* | Bahia | Camacan | -15.38 | 376 | 7 | NAF | 41647 | 13199 | - | 35 | 0.0701 |
| SLFT1982 | *Dendropsophus* | *Dendropsophus minutus* | Bahia | Camacan | -15.38 | 376 | 7 | NAF | 41647 | 11840 | - | 27 | 0.1249 |
| SLFT1983 | *Dendropsophus* | *Dendropsophus elegans* | Bahia | Camacan | -15.38 | 376 | 7 | NAF | 41647 | 10421 | - | 27 | 0.0947 |
| SLFT1984 | *Dendropsophus* | *Dendropsophus haddadi* | Bahia | Camacan | -15.38 | 376 | 7 | NAF | 41647 | 25724 | - | 61 | 0.1218 |
| SLFT1986 | *Dendropsophus* | *Dendropsophus elegans* | Bahia | Camacan | -15.38 | 376 | 7 | NAF | 41647 | 17493 | - | 36 | 0.1187 |
| SLFT1988 | *Dendropsophus* | *Dendropsophus haddadi* | Bahia | Camacan | -15.38 | 376 | 7 | NAF | 41647 | 19057 | - | 20 | 0.1559 |
| SLFT1994 | *Dendropsophus* | *Dendropsophus branneri* | Bahia | Camacan | -15.38 | 376 | 7 | NAF | 41647 | 14332 | - | 52 | 0.0407 |
| SLFT1995 | *Dendropsophus* | *Dendropsophus elegans* | Bahia | Camacan | -15.38 | 376 | 7 | NAF | 41647 | 6807 | - | 55 | 0.0667 |
| SLFT1996 | *Dendropsophus* | *Dendropsophus elegans* | Bahia | Camacan | -15.38 | 376 | 7 | NAF | 41647 | 8851 | - | 52 | 0.1368 |
| SLFT1997 | *Dendropsophus* | *Dendropsophus elegans* | Bahia | Camacan | -15.38 | 376 | 7 | NAF | 41647 | 17498 | - | 57 | 0.1144 |
| SLFT1998 | *Dendropsophus* | *Dendropsophus elegans* | Bahia | Camacan | -15.38 | 376 | 7 | NAF | 41647 | 7016 | - | 38 | 0.0863 |
| SLFT1999 | *Dendropsophus* | *Dendropsophus elegans* | Bahia | Camacan | -15.38 | 376 | 7 | NAF | 41647 | 20981 | - | 21 | 0.1533 |
| SLFT2000 | *Dendropsophus* | *Dendropsophus elegans* | Bahia | Camacan | -15.38 | 376 | 7 | NAF | 41647 | 25215 | - | 45 | 0.1575 |
| SLFT2030 | *Dendropsophus* | *Dendropsophus haddadi* | Bahia | Camacan | -15.38 | 376 | 7 | NAF | 41647 | 2324 | - | 54 | 0.1096 |
| SLFT2037 | *Dendropsophus* | *Dendropsophus elegans* | Espírito Santo | Linhares | -19.14 | 56 | 8 | CAF | 41649 | 5775 | - | 29 | 0.0674 |
| SLFT2038 | *Dendropsophus* | *Dendropsophus elegans* | Espírito Santo | Linhares | -19.14 | 56 | 8 | CAF | 41649 | 2272 | - | 28 | 0.1897 |
| SLFT2042 | *Dendropsophus* | *Dendropsophus elegans* | Espírito Santo | Linhares | -19.14 | 56 | 8 | CAF | 41649 | 3213 | - | 25 | 0.2616 |
| SLFT2058 | *Dendropsophus* | *Dendropsophus elegans* | Espírito Santo | Linhares | -19.14 | 56 | 8 | CAF | 41649 | 1859 | - | 105 | 0.1030 |
| SLFT2064 | *Dendropsophus* | *Dendropsophus bipunctatus* | Espírito Santo | Linhares | -19.14 | 56 | 8 | CAF | 41649 | 7556 | - | 29 | 0.2583 |
| SLFT2065 | *Dendropsophus* | *Dendropsophus bipunctatus* | Espírito Santo | Linhares | -19.14 | 56 | 8 | CAF | 41649 | 7070 | - | 37 | 0.2327 |
| SLFT2066 | *Dendropsophus* | *Dendropsophus bipunctatus* | Espírito Santo | Linhares | -19.14 | 56 | 8 | CAF | 41649 | 12557 | - | 28 | 0.0592 |
| SLFT2132 | *Dendropsophus* | *Dendropsophus elegans* | Espírito Santo | Linhares | -19.14 | 56 | 8 | CAF | 41649 | 7029 | - | 23 | 0.1863 |
| SLFT2133 | *Dendropsophus* | *Dendropsophus bipunctatus* | Espírito Santo | Linhares | -19.14 | 56 | 8 | CAF | 41649 | 1969 | - | 18 | 0.0795 |
| SLFT2134 | *Dendropsophus* | *Dendropsophus elegans* | Espírito Santo | Linhares | -19.14 | 56 | 8 | CAF | 41649 | 16049 | - | 87 | 0.1061 |
| SLFT2135 | *Dendropsophus* | *Dendropsophus bipunctatus* | Espírito Santo | Linhares | -19.14 | 56 | 8 | CAF | 41649 | 10904 | - | 5 | 0.2571 |
| SLFT2136 | *Dendropsophus* | *Dendropsophus minutus* | Espírito Santo | Linhares | -19.14 | 56 | 8 | CAF | 41649 | 2498 | - | 35 | 0.1905 |
| SLFT2137 | *Dendropsophus* | *Dendropsophus elegans* | Espírito Santo | Linhares | -19.14 | 56 | 8 | CAF | 41649 | 5723 | - | 29 | 0.1618 |
| SLFT2144 | *Dendropsophus* | *Dendropsophus elegans* | Espírito Santo | Linhares | -19.14 | 56 | 8 | CAF | 41649 | 5341 | - | 87 | 0.0604 |
| SLFT2154 | *Dendropsophus* | *Dendropsophus giesleri* | Espírito Santo | Santa Teresa | -19.92 | 810 | 9 | CAF | 41651 | 2835 | - | 80 | 0.0789 |
| SLFT2180 | *Dendropsophus* | *Dendropsophus giesleri* | Espírito Santo | Santa Teresa | -19.92 | 810 | 9 | CAF | 41651 | 5407 | - | 108 | 0.0939 |
| SLFT2202 | *Dendropsophus* | *Dendropsophus cf. branneri* | Espírito Santo | Santa Teresa | -19.92 | 810 | 9 | CAF | 41651 | 5500 | + | 46 | 0.1301 |
| SLFT2210 | *Dendropsophus* | *Dendropsophus sp.* | Espírito Santo | Santa Teresa | -19.92 | 810 | 9 | CAF | 41651 | 15497 | - | 21 | 0.1039 |
| SLFT2233 | *Dendropsophus* | *Dendropsophus elegans* | Espírito Santo | Santa Teresa | -19.92 | 810 | 9 | CAF | 41651 | 14054 | - | 20 | 0.1460 |
| SLFT2234 | *Dendropsophus* | *Dendropsophus elegans* | Espírito Santo | Santa Teresa | -19.92 | 810 | 9 | CAF | 41651 | 11696 | - | 33 | 0.1886 |
| SLFT2236 | *Dendropsophus* | *Dendropsophus bipunctatus* | Espírito Santo | Santa Teresa | -19.92 | 810 | 9 | CAF | 41651 | 4932 | + | 48 | 0.1238 |
| SLFT2237 | *Dendropsophus* | *Dendropsophus branneri* | Espírito Santo | Santa Teresa | -19.92 | 810 | 9 | CAF | 41651 | 12870 | - | 34 | 0.0988 |
| SLFT2238 | *Dendropsophus* | *Dendropsophus bipunctatus* | Espírito Santo | Santa Teresa | -19.92 | 810 | 9 | CAF | 41651 | 10555 | + | 25 | 0.2064 |
| SLFT2239 | *Dendropsophus* | *Dendropsophus bipunctatus* | Espírito Santo | Santa Teresa | -19.92 | 810 | 9 | CAF | 41651 | 18573 | - | 16 | 0.2198 |
| SLFT2245 | *Dendropsophus* | *Dendropsophus bipunctatus* | Espírito Santo | Santa Teresa | -19.92 | 810 | 9 | CAF | 41651 | 3390 | + | 38 | 0.1340 |
| SLFT2250 | *Dendropsophus* | *Dendropsophus bipunctatus* | Espírito Santo | Santa Teresa | -19.92 | 810 | 9 | CAF | 41651 | 13467 | + | 63 | 0.0786 |
| SLFT2251 | *Dendropsophus* | *Dendropsophus bipunctatus* | Espírito Santo | Santa Teresa | -19.92 | 810 | 9 | CAF | 41651 | 7346 | - | 29 | 0.1367 |
| SLFT2252 | *Dendropsophus* | *Dendropsophus bipunctatus* | Espírito Santo | Santa Teresa | -19.92 | 810 | 9 | CAF | 41651 | 7042 | - | 54 | 0.1239 |
| SLFT2255 | *Dendropsophus* | *Dendropsophus bipunctatus* | Espírito Santo | Santa Teresa | -19.92 | 810 | 9 | CAF | 41651 | 8629 | - | 52 | 0.1189 |
| SLFT2256 | *Dendropsophus* | *Dendropsophus bipunctatus* | Espírito Santo | Santa Teresa | -19.92 | 810 | 9 | CAF | 41651 | 11371 | - | 64 | 0.1465 |
| SLFT2258 | *Dendropsophus* | *Dendropsophus bipunctatus* | Espírito Santo | Santa Teresa | -19.92 | 810 | 9 | CAF | 41651 | 3930 | - | 80 | 0.0931 |
| SLFT2259 | *Dendropsophus* | *Dendropsophus bipunctatus* | Espírito Santo | Santa Teresa | -19.92 | 810 | 9 | CAF | 41651 | 13952 | - | 25 | 0.0942 |
| SLFT2260 | *Dendropsophus* | *Dendropsophus bipunctatus* | Espírito Santo | Santa Teresa | -19.92 | 810 | 9 | CAF | 41651 | 9905 | - | 34 | 0.1071 |
| SLFT2261 | *Dendropsophus* | *Dendropsophus bipunctatus* | Espírito Santo | Santa Teresa | -19.92 | 810 | 9 | CAF | 41651 | 22156 | + | 11 | 0.0806 |
| SLFT2263 | *Dendropsophus* | *Dendropsophus bipunctatus* | Espírito Santo | Santa Teresa | -19.92 | 810 | 9 | CAF | 41651 | 16344 | + | 42 | 0.0862 |
| SLFT2264 | *Dendropsophus* | *Dendropsophus branneri* | Espírito Santo | Santa Teresa | -19.92 | 810 | 9 | CAF | 41651 | 10830 | + | 42 | 0.1217 |
| SLFT2268 | *Dendropsophus* | *Dendropsophus elegans* | Espírito Santo | Santa Teresa | -19.92 | 810 | 9 | CAF | 41651 | 8503 | - | 38 | 0.0895 |
| SLFT2270 | *Dendropsophus* | *Dendropsophus branneri* | Espírito Santo | Santa Teresa | -19.92 | 810 | 9 | CAF | 41651 | 4917 | - | 26 | 0.2137 |
| SLFT2271 | *Dendropsophus* | *Dendropsophus bipunctatus* | Espírito Santo | Santa Teresa | -19.92 | 810 | 9 | CAF | 41651 | 12249 | - | 30 | 0.0950 |
| SLFT2273 | *Dendropsophus* | *Dendropsophus bipunctatus* | Espírito Santo | Santa Teresa | -19.92 | 810 | 9 | CAF | 41651 | 4911 | - | 20 | 0.2542 |
| SLFT2274 | *Dendropsophus* | *Dendropsophus bipunctatus* | Espírito Santo | Santa Teresa | -19.92 | 810 | 9 | CAF | 41651 | 6771 | + | 42 | 0.0730 |
| SLFT2275 | *Dendropsophus* | *Dendropsophus bipunctatus* | Espírito Santo | Santa Teresa | -19.92 | 810 | 9 | CAF | 41651 | 13163 | - | 40 | 0.1195 |
| SLFT2276 | *Dendropsophus* | *Dendropsophus bipunctatus* | Espírito Santo | Santa Teresa | -19.92 | 810 | 9 | CAF | 41651 | 9542 | - | 51 | 0.0992 |
| SLFT2278 | *Dendropsophus* | *Dendropsophus bipunctatus* | Espírito Santo | Santa Teresa | -19.92 | 810 | 9 | CAF | 41651 | 11147 | - | 36 | 0.1174 |
| SLFT2279 | *Dendropsophus* | *Dendropsophus branneri* | Espírito Santo | Vargem Alta | -20.48 | 1148 | 10 | CAF | 41653 | 7559 | - | 24 | 0.1443 |
| SLFT2280 | *Dendropsophus* | *Dendropsophus minutus* | Espírito Santo | Vargem Alta | -20.48 | 1148 | 10 | CAF | 41653 | 5100 | - | 24 | 0.1882 |
| SLFT2283 | *Dendropsophus* | *Dendropsophus minutus* | Espírito Santo | Vargem Alta | -20.48 | 1148 | 10 | CAF | 41653 | 15866 | - | 20 | 0.2152 |
| SLFT2287 | *Dendropsophus* | *Dendropsophus haddadi* | Espírito Santo | Vargem Alta | -20.48 | 1148 | 10 | CAF | 41653 | 11112 | + | 47 | 0.0814 |
| SLFT2294 | *Dendropsophus* | *Dendropsophus minutus* | Espírito Santo | Vargem Alta | -20.48 | 1148 | 10 | CAF | 41653 | 8151 | - | 73 | 0.0859 |
| SLFT2296 | *Dendropsophus* | *Dendropsophus elegans* | Espírito Santo | Vargem Alta | -20.48 | 1148 | 10 | CAF | 41653 | 7775 | - | 31 | 0.1634 |
| SLFT2299 | *Dendropsophus* | *Dendropsophus haddadi* | Espírito Santo | Vargem Alta | -20.48 | 1148 | 10 | CAF | 41653 | 13173 | - | 97 | 0.0587 |
| SLFT2300 | *Dendropsophus* | *Dendropsophus elegans* | Espírito Santo | Vargem Alta | -20.48 | 1148 | 10 | CAF | 41653 | 4221 | + | 30 | 0.1888 |
| SLFT2310 | *Dendropsophus* | *Dendropsophus minutus* | Espírito Santo | Vargem Alta | -20.48 | 1148 | 10 | CAF | 41653 | 8420 | - | 23 | 0.1410 |
| SLFT2327 | *Dendropsophus* | *Dendropsophus minutus* | Espírito Santo | Vargem Alta | -20.48 | 1148 | 10 | CAF | 41653 | 14618 | - | 32 | 0.1126 |
| SLFT2336 | *Dendropsophus* | *Dendropsophus minutus* | Espírito Santo | Vargem Alta | -20.48 | 1148 | 10 | CAF | 41653 | 2239 | - | 57 | 0.1063 |
| SLFT2338 | *Dendropsophus* | *Dendropsophus minutus* | Espírito Santo | Vargem Alta | -20.48 | 1148 | 10 | CAF | 41653 | 9029 | - | 33 | 0.1969 |
| SLFT2339 | *Dendropsophus* | *Dendropsophus minutus* | Espírito Santo | Vargem Alta | -20.48 | 1148 | 10 | CAF | 41653 | 4185 | + | 33 | 0.1317 |
| SLFT2340 | *Dendropsophus* | *Dendropsophus minutus* | Espírito Santo | Vargem Alta | -20.48 | 1148 | 10 | CAF | 41653 | 8133 | + | 28 | 0.1946 |
| SLFT2341 | *Dendropsophus* | *Dendropsophus minutus* | Espírito Santo | Vargem Alta | -20.48 | 1148 | 10 | CAF | 41653 | 21145 | - | 22 | 0.2046 |
| SLFT2343 | *Dendropsophus* | *Dendropsophus minutus* | Espírito Santo | Vargem Alta | -20.48 | 1148 | 10 | CAF | 41653 | 4659 | + | 20 | 0.1165 |
| SLFT2346 | *Dendropsophus* | *Dendropsophus minutus* | Espírito Santo | Vargem Alta | -20.48 | 1148 | 10 | CAF | 41653 | 13757 | - | 28 | 0.1417 |
| SLFT2347 | *Dendropsophus* | *Dendropsophus minutus* | Espírito Santo | Vargem Alta | -20.48 | 1148 | 10 | CAF | 41653 | 4964 | + | 30 | 0.1498 |
| SLFT2348 | *Dendropsophus* | *Dendropsophus minutus* | Espírito Santo | Vargem Alta | -20.48 | 1148 | 10 | CAF | 41653 | 3323 | - | 32 | 0.1472 |
| SLFT2349 | *Dendropsophus* | *Dendropsophus minutus* | Espírito Santo | Vargem Alta | -20.48 | 1148 | 10 | CAF | 41653 | 4968 | - | 42 | 0.0970 |
| SLFT2354 | *Dendropsophus* | *Dendropsophus haddadi* | Espírito Santo | Vargem Alta | -20.48 | 1148 | 10 | CAF | 41653 | 1533 | + | 34 | 0.1566 |
| SLFT2360 | *Dendropsophus* | *Dendropsophus haddadi* | Espírito Santo | Vargem Alta | -20.48 | 1148 | 10 | CAF | 41653 | 1398 | + | 29 | 0.2140 |
| SLFT2363 | *Dendropsophus* | *Dendropsophus haddadi* | Espírito Santo | Vargem Alta | -20.48 | 1148 | 10 | CAF | 41653 | 16318 | - | 15 | 0.2304 |
| SLFT2364 | *Dendropsophus* | *Dendropsophus elegans* | Espírito Santo | Vargem Alta | -20.48 | 1148 | 10 | CAF | 41653 | 10952 | - | 127 | 0.0532 |
| SLFT2367 | *Dendropsophus* | *Dendropsophus elegans* | Espírito Santo | Vargem Alta | -20.48 | 1148 | 10 | CAF | 41653 | 7740 | NA | 28 | 0.1373 |
| SLFT2370 | *Dendropsophus* | *Dendropsophus minutus* | Espírito Santo | Vargem Alta | -20.48 | 1148 | 10 | CAF | 41653 | 12462 | NA | 43 | 0.0927 |
| SLFT2374 | *Dendropsophus* | *Dendropsophus minutus* | Espírito Santo | Vargem Alta | -20.48 | 1148 | 10 | CAF | 41653 | 7854 | - | 26 | 0.1495 |
| SLFT2375 | *Dendropsophus* | *Dendropsophus branneri* | Espírito Santo | Vargem Alta | -20.48 | 1148 | 10 | CAF | 41653 | 11619 | + | 68 | 0.0747 |
| SLFT2864 | *Dendropsophus* | *Dendropsophus elegans* | Rio de Janeiro | Macaé, Barra do Sana | -22.36 | 483 | 11 | CAF | 41654 | 4980 | - | 25 | 0.0823 |
| SLFT2872 | *Dendropsophus* | *Dendropsophus elegans* | Rio de Janeiro | Macaé, Barra do Sana | -22.36 | 483 | 11 | CAF | 41654 | 8148 | - | 10 | 0.1563 |
| SLFT2913 | *Dendropsophus* | *Dendropsophus elegans* | Rio de Janeiro | Macaé, Barra do Sana | -22.36 | 483 | 11 | CAF | 41654 | 24413 | - | 12 | 0.0973 |
| SLFT2914 | *Dendropsophus* | *Dendropsophus elegans* | Rio de Janeiro | Macaé, Barra do Sana | -22.36 | 483 | 11 | CAF | 41654 | 33161 | + | 9 | 0.2116 |
| SLFT4796 | *Dendropsophus* | *Dendropsophus nanus* | Rio Grande do Norte | Baía Formosa | -6.41 | 23 | 1 | NAF | 42157 | 88909 | - | 25 | 0.1690 |
| SLFT4801 | *Dendropsophus* | *Dendropsophus nanus* | Rio Grande do Norte | Baía Formosa | -6.41 | 23 | 1 | NAF | 42157 | 27666 | - | 8 | 0.1662 |
| SLFT4802 | *Dendropsophus* | *Dendropsophus nanus* | Rio Grande do Norte | Baía Formosa | -6.41 | 23 | 1 | NAF | 42157 | 150486 | - | 17 | 0.0846 |
| SLFT4813 | *Dendropsophus* | *Dendropsophus nanus* | Rio Grande do Norte | Baía Formosa | -6.41 | 23 | 1 | NAF | 42157 | 31262 | - | 55 | 0.1856 |
| SLFT4823 | *Dendropsophus* | *Dendropsophus nanus* | Rio Grande do Norte | Baía Formosa | -6.41 | 23 | 1 | NAF | 42157 | 15662 | - | 45 | 0.3978 |
| SLFT4827 | *Dendropsophus* | *Dendropsophus nanus* | Rio Grande do Norte | Baía Formosa | -6.41 | 23 | 1 | NAF | 42157 | 10804 | - | 56 | 0.5613 |
| SLFT4844 | *Dendropsophus* | *Dendropsophus nanus* | Rio Grande do Norte | Baía Formosa | -6.41 | 23 | 1 | NAF | 42157 | 19908 | - | 68 | 0.1799 |
| SLFT4889 | *Dendropsophus* | *Dendropsophus minutus* | Paraíba | João Pessoa | -7.12 | 26 | 2 | NAF | 42161 | 44436 | - | 17 | 0.0930 |
| SLFT4890 | *Dendropsophus* | *Dendropsophus minutus* | Paraíba | João Pessoa | -7.12 | 26 | 2 | NAF | 42161 | 15417 | - | 108 | 0.1604 |
| SLFT4891 | *Dendropsophus* | *Dendropsophus minutus* | Paraíba | João Pessoa | -7.12 | 26 | 2 | NAF | 42161 | 8977 | - | 52 | 0.2785 |
| SLFT4893 | *Dendropsophus* | *Dendropsophus branneri* | Paraíba | João Pessoa | -7.12 | 26 | 2 | NAF | 42161 | 10284 | - | 28 | 0.1170 |
| SLFT4897 | *Dendropsophus* | *Dendropsophus branneri* | Paraíba | João Pessoa | -7.12 | 26 | 2 | NAF | 42161 | 16779 | - | 42 | 0.1431 |
| SLFT4904 | *Dendropsophus* | *Dendropsophus minutus* | Paraíba | João Pessoa | -7.12 | 26 | 2 | NAF | 42161 | 22790 | - | 12 | 0.3279 |
| SLFT4906 | *Dendropsophus* | *Dendropsophus minutus* | Paraíba | João Pessoa | -7.12 | 26 | 2 | NAF | 42161 | 12615 | - | 70 | 0.4899 |
| SLFT4908 | *Dendropsophus* | *Dendropsophus minutus* | Paraíba | João Pessoa | -7.12 | 26 | 2 | NAF | 42161 | 15438 | - | 95 | 0.4991 |
| SLFT4909 | *Dendropsophus* | *Dendropsophus minutus* | Paraíba | João Pessoa | -7.12 | 26 | 2 | NAF | 42161 | 18186 | - | 97 | 0.3741 |
| SLFT4911 | *Dendropsophus* | *Dendropsophus branneri* | Paraíba | João Pessoa | -7.12 | 26 | 2 | NAF | 42161 | 21374 | - | 82 | 0.1331 |
| SLFT4916 | *Dendropsophus* | *Dendropsophus branneri* | Paraíba | João Pessoa | -7.12 | 26 | 2 | NAF | 42161 | 8593 | - | 51 | 0.1041 |
| SLFT4924 | *Dendropsophus* | *Dendropsophus branneri* | Paraíba | João Pessoa | -7.12 | 26 | 2 | NAF | 42161 | 17955 | - | 45 | 0.4013 |
| SLFT4925 | *Dendropsophus* | *Dendropsophus minutus* | Paraíba | João Pessoa | -7.12 | 26 | 2 | NAF | 42161 | 21151 | - | 104 | 0.4504 |
| SLFT4929 | *Dendropsophus* | *Dendropsophus minutus* | Paraíba | João Pessoa | -7.12 | 26 | 2 | NAF | 42161 | 9304 | - | 84 | 0.1643 |
| SLFT4932 | *Dendropsophus* | *Dendropsophus branneri* | Paraíba | João Pessoa | -7.12 | 26 | 2 | NAF | 42161 | 25774 | - | 233 | 0.1174 |
| SLFT4935 | *Dendropsophus* | *Dendropsophus oliveirai* | Paraíba | João Pessoa | -7.12 | 26 | 2 | NAF | 42161 | 6575 | - | 60 | 0.2068 |
| SLFT4942 | *Dendropsophus* | *Dendropsophus branneri* | Paraíba | João Pessoa | -7.12 | 26 | 2 | NAF | 42161 | 41306 | - | 110 | 0.1915 |
| SLFT4944 | *Dendropsophus* | *Dendropsophus minutus* | Paraíba | João Pessoa | -7.12 | 26 | 2 | NAF | 42161 | 35121 | - | 72 | 0.0745 |
| SLFT4945 | *Dendropsophus* | *Dendropsophus minutus* | Paraíba | João Pessoa | -7.12 | 26 | 2 | NAF | 42161 | 35935 | - | 53 | 0.1357 |
| SLFT4952 | *Dendropsophus* | *Dendropsophus minutus* | Paraíba | João Pessoa | -7.12 | 26 | 2 | NAF | 42161 | 6918 | - | 62 | 0.4820 |
| SLFT4964 | *Dendropsophus* | *Dendropsophus minutus* | Paraíba | João Pessoa | -7.12 | 26 | 2 | NAF | 42161 | 44547 | - | 178 | 0.4062 |
| SLFT4965 | *Dendropsophus* | *Dendropsophus minutus* | Paraíba | João Pessoa | -7.12 | 26 | 2 | NAF | 42161 | 15013 | - | 249 | 0.2462 |
| SLFT4967 | *Dendropsophus* | *Dendropsophus minutus* | Paraíba | João Pessoa | -7.12 | 26 | 2 | NAF | 42161 | 10439 | - | 57 | 0.3906 |
| SLFT4968 | *Dendropsophus* | *Dendropsophus minutus* | Paraíba | João Pessoa | -7.12 | 26 | 2 | NAF | 42161 | 23750 | - | 129 | 0.3319 |
| SLFT4974 | *Dendropsophus* | *Dendropsophus oliveirai* | Paraíba | João Pessoa | -7.12 | 26 | 2 | NAF | 42161 | 32270 | - | 84 | 0.1331 |
| SLFT4977 | *Dendropsophus* | *Dendropsophus branneri* | Paraíba | João Pessoa | -7.12 | 26 | 2 | NAF | 42161 | 12615 | - | 59 | 0.4182 |
| SLFT4986 | *Dendropsophus* | *Dendropsophus branneri* | Paraíba | João Pessoa | -7.12 | 26 | 2 | NAF | 42161 | 27520 | - | 87 | 0.0369 |
| SLFT5035 | *Dendropsophus* | *Dendropsophus haddadi* | Pernambuco | Paulista | -7.93 | 30 | 3 | NAF | 42164 | 8407 | - | 139 | 0.1063 |
| SLFT5081 | *Dendropsophus* | *Dendropsophus* sp*.* | Pernambuco | Paulista | -7.93 | 30 | 3 | NAF | 42164 | 22721 | - | 48 | 0.0517 |
| SLFT5092 | *Dendropsophus* | *Dendropsophus branneri* | Pernambuco | Paulista | -7.93 | 30 | 3 | NAF | 42164 | 1806 | - | 118 | 0.4642 |
| SLFT5109 | *Dendropsophus* | *Dendropsophus minutus* | Sergipe | Areia Branca | -10.76 | 373 | 5 | NAF | 42165 | 30152 | - | 216 | 0.1920 |
| SLFT5110 | *Dendropsophus* | *Dendropsophus minutus* | Sergipe | Areia Branca | -10.76 | 373 | 5 | NAF | 42165 | 18538 | - | 196 | 0.2541 |
| SLFT5113 | *Dendropsophus* | *Dendropsophus minutus* | Sergipe | Areia Branca | -10.76 | 373 | 5 | NAF | 42165 | 21420 | - | 113 | 0.1225 |
| SLFT5117 | *Dendropsophus* | *Dendropsophus minutus* | Sergipe | Areia Branca | -10.76 | 373 | 5 | NAF | 42165 | 40082 | - | 67 | 0.1009 |
| SLFT5121 | *Dendropsophus* | *Dendropsophus minutus* | Sergipe | Areia Branca | -10.76 | 373 | 5 | NAF | 42165 | 47048 | - | 68 | 0.0369 |
| SLFT5125 | *Dendropsophus* | *Dendropsophus minutus* | Sergipe | Areia Branca | -10.76 | 373 | 5 | NAF | 42165 | 35226 | - | 124 | 0.1362 |
| SLFT5168 | *Dendropsophus* | *Dendropsophus haddadi* | Sergipe | Areia Branca | -10.76 | 373 | 5 | NAF | 42165 | 19762 | - | 75 | 0.0949 |
| SLFT5169 | *Dendropsophus* | *Dendropsophus haddadi* | Sergipe | Areia Branca | -10.76 | 373 | 5 | NAF | 42165 | 3793 | - | 49 | 0.1321 |
| SLFT5171 | *Dendropsophus* | *Dendropsophus minutus* | Sergipe | Areia Branca | -10.76 | 373 | 5 | NAF | 42165 | 33125 | - | 91 | 0.1346 |
| SLFT5172 | *Dendropsophus* | *Dendropsophus minutus* | Sergipe | Areia Branca | -10.76 | 373 | 5 | NAF | 42165 | 16590 | - | 59 | 0.1538 |
| SLFT5173 | *Dendropsophus* | *Dendropsophus haddadi* | Sergipe | Areia Branca | -10.76 | 373 | 5 | NAF | 42165 | 43032 | - | 32 | 0.0578 |
| SLFT5174 | *Dendropsophus* | *Dendropsophus minutus* | Sergipe | Areia Branca | -10.76 | 373 | 5 | NAF | 42165 | 14743 | - | 82 | 0.0590 |
| SLFT5179 | *Dendropsophus* | *Dendropsophus minutus* | Sergipe | Areia Branca | -10.76 | 373 | 5 | NAF | 42165 | 71711 | - | 13 | 0.1554 |
| SLFT5181 | *Dendropsophus* | *Dendropsophus minutus* | Sergipe | Areia Branca | -10.76 | 373 | 5 | NAF | 42165 | 48711 | - | 52 | 0.0326 |
| SLFT5183 | *Dendropsophus* | *Dendropsophus haddadi* | Sergipe | Areia Branca | -10.76 | 373 | 5 | NAF | 42165 | 48330 | - | 40 | 0.0380 |
| SLFT5184 | *Dendropsophus* | *Dendropsophus haddadi* | Sergipe | Areia Branca | -10.76 | 373 | 5 | NAF | 42165 | 31406 | - | 86 | 0.1459 |
| SLFT5187 | *Dendropsophus* | *Dendropsophus sp.* | Sergipe | Areia Branca | -10.76 | 373 | 5 | NAF | 42165 | 29412 | - | 89 | 0.0891 |
| SLFT5188 | *Dendropsophus* | *Dendropsophus minutus* | Sergipe | Areia Branca | -10.76 | 373 | 5 | NAF | 42165 | 5668 | - | 52 | 0.1394 |
| SLFT5190 | *Dendropsophus* | *Dendropsophus haddadi* | Sergipe | Areia Branca | -10.76 | 373 | 5 | NAF | 42165 | 20985 | - | 100 | 0.1238 |
| SLFT5191 | *Dendropsophus* | *Dendropsophus haddadi* | Sergipe | Areia Branca | -10.76 | 373 | 5 | NAF | 42165 | 15343 | - | 240 | 0.2548 |
| SLFT5192 | *Dendropsophus* | *Dendropsophus minutus* | Sergipe | Areia Branca | -10.76 | 373 | 5 | NAF | 42165 | 10725 | - | 72 | 0.1930 |
| SLFT5193 | *Dendropsophus* | *Dendropsophus haddadi* | Sergipe | Areia Branca | -10.76 | 373 | 5 | NAF | 42165 | 47458 | - | 72 | 0.0398 |
| SLFT5195 | *Dendropsophus* | *Dendropsophus haddadi* | Sergipe | Areia Branca | -10.76 | 373 | 5 | NAF | 42165 | 24803 | - | 217 | 0.0363 |
| SLFT5196 | *Dendropsophus* | *Dendropsophus haddadi* | Sergipe | Areia Branca | -10.76 | 373 | 5 | NAF | 42165 | 12243 | - | 133 | 0.2281 |
| SLFT5199 | *Dendropsophus* | *Dendropsophus minutus* | Sergipe | Areia Branca | -10.76 | 373 | 5 | NAF | 42165 | 26356 | - | 40 | 0.0639 |
| SLFT5200 | *Dendropsophus* | *Dendropsophus haddadi* | Sergipe | Areia Branca | -10.76 | 373 | 5 | NAF | 42165 | 2287 | - | 70 | 0.1300 |
| SLFT5201 | *Dendropsophus* | *Dendropsophus haddadi* | Sergipe | Areia Branca | -10.76 | 373 | 5 | NAF | 42165 | 17582 | - | 91 | 0.1526 |
| SLFT5202 | *Dendropsophus* | *Dendropsophus haddadi* | Sergipe | Areia Branca | -10.76 | 373 | 5 | NAF | 42165 | 27652 | - | 161 | 0.1605 |
| SLFT5204 | *Dendropsophus* | *Dendropsophus haddadi* | Sergipe | Areia Branca | -10.76 | 373 | 5 | NAF | 42165 | 22893 | - | 53 | 0.1405 |
| SLFT5208 | *Dendropsophus* | *Dendropsophus haddadi* | Sergipe | Areia Branca | -10.76 | 373 | 5 | NAF | 42165 | 37316 | - | 86 | 0.1438 |
| SLFT5210 | *Dendropsophus* | *Dendropsophus haddadi* | Sergipe | Areia Branca | -10.76 | 373 | 5 | NAF | 42165 | 21390 | - | 74 | 0.1747 |
| SLFT5213 | *Dendropsophus* | *Dendropsophus haddadi* | Sergipe | Areia Branca | -10.76 | 373 | 5 | NAF | 42165 | 14786 | - | 272 | 0.1637 |
| SLFT5294 | *Dendropsophus* | *Dendropsophus* sp*.* | Bahia | Mata de São João | -12.56 | 41 | 6 | NAF | 42168 | 25009 | - | 41 | 0.1415 |
| SLFT5299 | *Dendropsophus* | *Dendropsophus branneri* | Bahia | Mata de São João | -12.56 | 41 | 6 | NAF | 42168 | 23490 | - | 140 | 0.1045 |
| SLFT2074 | *Itapotihyla* | *Itapotihyla langsdorffii* | Espírito Santo | Linhares | -19.14 | 56 | 8 | CAF | 41649 | 16353 | - | 17 | 0.1955 |
| SLFT2078 | *Itapotihyla* | *Itapotihyla langsdorffii* | Espírito Santo | Linhares | -19.14 | 56 | 8 | CAF | 41649 | 8095 | - | 34 | 0.1747 |
| SLFT2219 | *Itapotihyla* | *Itapotihyla langsdorffii* | Espírito Santo | Santa Teresa | -19.92 | 810 | 9 | CAF | 41651 | 14584 | - | 19 | 0.1266 |
| SLFT2222 | *Itapotihyla* | *Itapotihyla langsdorffii* | Espírito Santo | Santa Teresa | -19.92 | 810 | 9 | CAF | 41651 | 11700 | - | 24 | 0.1090 |
| SLFT2223 | *Itapotihyla* | *Itapotihyla langsdorffii* | Espírito Santo | Santa Teresa | -19.92 | 810 | 9 | CAF | 41651 | 13846 | - | 23 | 0.0779 |
| SLFT2079 | *Aparasphenodon* | *Aparasphenodon brunoi* | Espírito Santo | Linhares | -19.14 | 56 | 8 | CAF | 41649 | 13744 | - | 12 | 0.1553 |
| SLFT2083 | *Aparasphenodon* | *Aparasphenodon brunoi* | Espírito Santo | Linhares | -19.14 | 56 | 8 | CAF | 41649 | 7003 | - | 36 | 0.1721 |
| SLFT2085 | *Aparasphenodon* | *Aparasphenodon brunoi* | Espírito Santo | Linhares | -19.14 | 56 | 8 | CAF | 41649 | 2646 | - | 20 | 0.1713 |
| SLFT2086 | *Aparasphenodon* | *Aparasphenodon brunoi* | Espírito Santo | Linhares | -19.14 | 56 | 8 | CAF | 41649 | 15884 | - | 30 | 0.0893 |
| SLFT2088 | *Aparasphenodon* | *Aparasphenodon brunoi* | Espírito Santo | Linhares | -19.14 | 56 | 8 | CAF | 41649 | 19594 | - | 22 | 0.2270 |
| SLFT2089 | *Aparasphenodon* | *Aparasphenodon brunoi* | Espírito Santo | Linhares | -19.14 | 56 | 8 | CAF | 41649 | 4093 | - | 23 | 0.1739 |
| SLFT2090 | *Aparasphenodon* | *Aparasphenodon brunoi* | Espírito Santo | Linhares | -19.14 | 56 | 8 | CAF | 41649 | 11101 | - | 12 | 0.0714 |
| SLFT2091 | *Aparasphenodon* | *Aparasphenodon brunoi* | Espírito Santo | Linhares | -19.14 | 56 | 8 | CAF | 41649 | 6964 | - | 23 | 0.1665 |
| SLFT2092 | *Aparasphenodon* | *Aparasphenodon brunoi* | Espírito Santo | Linhares | -19.14 | 56 | 8 | CAF | 41649 | 17012 | - | 22 | 0.2864 |
| SLFT2093 | *Aparasphenodon* | *Aparasphenodon brunoi* | Espírito Santo | Linhares | -19.14 | 56 | 8 | CAF | 41649 | 7553 | - | 17 | 0.2168 |
| SLFT2095 | *Aparasphenodon* | *Aparasphenodon brunoi* | Espírito Santo | Linhares | -19.14 | 56 | 8 | CAF | 41649 | 1600 | - | 45 | 0.2143 |
| SLFT2107 | *Aparasphenodon* | *Aparasphenodon brunoi* | Espírito Santo | Linhares | -19.14 | 56 | 8 | CAF | 41649 | 6611 | - | 21 | 0.1687 |
| SLFT1879 | Phyllodytes | *Phyllodytes gyrinaethes* | Alagoas | Murici | -9.21 | 233 | 4 | NAF | 41520 | 8829 | + | 29 | 0.1035 |
| SLFT1880 | *Phyllodytes* | *Phyllodytes gyrinaethes* | Alagoas | Murici | -9.21 | 233 | 4 | NAF | 41520 | 2802 | + | 38 | 0.1206 |
| SLFT1884 | *Phyllodytes* | *Phyllodytes gyrinaethes* | Alagoas | Murici | -9.21 | 233 | 4 | NAF | 41520 | 6093 | + | 30 | 0.0704 |
| SLFT1885 | *Phyllodytes* | *Phyllodytes gyrinaethes* | Alagoas | Murici | -9.21 | 233 | 4 | NAF | 41520 | 4309 | - | 30 | 0.1197 |
| SLFT1886 | *Phyllodytes* | *Phyllodytes gyrinaethes* | Alagoas | Murici | -9.21 | 233 | 4 | NAF | 41520 | 9249 | - | 16 | 0.1721 |
| SLFT1891 | *Phyllodytes* | *Phyllodytes edelmoi* | Alagoas | Murici | -9.21 | 233 | 4 | NAF | 41520 | 4860 | - | 104 | 0.0965 |
| SLFT1892 | *Phyllodytes* | *Phyllodytes edelmoi* | Alagoas | Murici | -9.21 | 233 | 4 | NAF | 41520 | 15085 | - | 84 | 0.1166 |
| SLFT1894 | *Phyllodytes* | *Phyllodytes edelmoi* | Alagoas | Murici | -9.21 | 233 | 4 | NAF | 41520 | 9576 | + | 41 | 0.0698 |
| SLFT1895 | *Phyllodytes* | *Phyllodytes edelmoi* | Alagoas | Murici | -9.21 | 233 | 4 | NAF | 41520 | 3619 | + | 31 | 0.1434 |
| SLFT1896 | *Phyllodytes* | *Phyllodytes edelmoi* | Alagoas | Murici | -9.21 | 233 | 4 | NAF | 41520 | 4009 | - | 48 | 0.1016 |
| SLFT1897 | *Phyllodytes* | *Phyllodytes edelmoi* | Alagoas | Murici | -9.21 | 233 | 4 | NAF | 41520 | 8345 | - | 61 | 0.0680 |
| SLFT1898 | *Phyllodytes* | *Phyllodytes edelmoi* | Alagoas | Murici | -9.21 | 233 | 4 | NAF | 41520 | 12425 | - | 40 | 0.0562 |
| SLFT1902 | *Phyllodytes* | *Phyllodytes gyrinaethes* | Alagoas | Murici | -9.21 | 233 | 4 | NAF | 41520 | 12639 | + | 32 | 0.1188 |
| SLFT1904 | *Phyllodytes* | *Phyllodytes* cf. *edelmoi* | Alagoas | Murici | -9.21 | 233 | 4 | NAF | 41520 | 19655 | + | 68 | 0.0664 |
| SLFT1906 | *Phyllodytes* | *Phyllodytes edelmoi* | Alagoas | Murici | -9.21 | 233 | 4 | NAF | 41520 | 48417 | + | 11 | 0.1272 |
| SLFT1907 | *Phyllodytes* | *Phyllodytes edelmoi* | Alagoas | Murici | -9.21 | 233 | 4 | NAF | 41520 | 9180 | + | 147 | 0.0212 |
| SLFT1920 | *Phyllodytes* | *Phyllodytes* cf. *acuminatus* | Alagoas | Murici | -9.21 | 233 | 4 | NAF | 41520 | 14804 | + | 81 | 0.0589 |
| SLFT2981 | *Phyllodytes* | *Phyllodytes gyrinaethes* | Alagoas | Murici | -9.21 | 233 | 4 | NAF | 41713 | 3406 | - | 28 | 0.1773 |
| SLFT2988 | *Phyllodytes* | *Phyllodytes edelmoi* | Alagoas | Murici | -9.21 | 233 | 4 | NAF | 41713 | 5488 | - | 79 | 0.1494 |
| SLFT3006 | *Phyllodytes* | *Phyllodytes gyrinaethes* | Alagoas | Murici | -9.21 | 233 | 4 | NAF | 41713 | 3433 | - | 52 | 0.0463 |
| SLFT3019 | *Phyllodytes* | *Phyllodytes gyrinaethes* | Alagoas | Murici | -9.21 | 233 | 4 | NAF | 41713 | 5261 | - | 26 | 0.1725 |
| SLFT5203 | *Phyllodytes* | *Phyllodytes* sp. | Sergipe | Areia Branca | -10.76 | 373 | 5 | NAF | 42165 | 51730 | - | 31 | 0.1093 |
| SLFT5212 | *Phyllodytes* | *Phyllodytes* sp*.* | Sergipe | Areia Branca | -10.76 | 373 | 5 | NAF | 42165 | 77395 | - | 18 | 0.0684 |
| SLFT0547 | *Phyllomedusa* | *Phyllomedusa distincta* | São Paulo | Iporanga | -24.60 | 412 | 18 | CAF | 41281 | 41551 | + | 9 | 0.1179 |
| SLFT0556 | *Phyllomedusa* | *Phyllomedusa distincta* | São Paulo | Iporanga | -24.60 | 412 | 18 | CAF | 41281 | 39173 | - | 11 | 0.1060 |
| SLFT0557 | *Phyllomedusa* | *Phyllomedusa distincta* | São Paulo | Iporanga | -24.60 | 412 | 18 | CAF | 41281 | 13274 | + | 14 | 0.0596 |
| SLFT0559 | *Phyllomedusa* | *Phyllomedusa distincta* | São Paulo | Iporanga | -24.60 | 412 | 18 | CAF | 41281 | 61323 | - | 11 | 0.1799 |
| SLFT0878 | *Phyllomedusa* | *Phyllomedusa distincta* | Santa Catarina | Pomerode | -26.77 | 228 | 21 | SAF | 41285 | 2077 | + | 44 | 0.1463 |
| SLFT1072 | *Phyllomedusa* | *Phyllomedusa distincta* | São Paulo | Pedro de Toledo | -24.32 | 209 | 16 | CAF | 41328 | 42240 | - | 38 | 0.0582 |
| SLFT1073 | *Phyllomedusa* | *Phyllomedusa distincta* | São Paulo | Pedro de Toledo | -24.32 | 209 | 16 | CAF | 41328 | 17359 | - | 31 | 0.0511 |
| SLFT1074 | *Phyllomedusa* | *Phyllomedusa distincta* | São Paulo | Pedro de Toledo | -24.32 | 209 | 16 | CAF | 41328 | 13865 | - | 42 | 0.0558 |
| SLFT1075 | *Phyllomedusa* | *Phyllomedusa distincta* | São Paulo | Pedro de Toledo | -24.32 | 209 | 16 | CAF | 41328 | 34720 | - | 46 | 0.0632 |
| SLFT1077 | *Phyllomedusa* | *Phyllomedusa distincta* | São Paulo | Pedro de Toledo | -24.32 | 209 | 16 | CAF | 41328 | 37679 | - | 45 | 0.0415 |
| SLFT1078 | *Phyllomedusa* | *Phyllomedusa distincta* | São Paulo | Pedro de Toledo | -24.32 | 209 | 16 | CAF | 41328 | 38925 | - | 40 | 0.0512 |
| SLFT1272 | *Phyllomedusa* | *Phyllomedusa tetraploidea* | Santa Catarina | São Miguel d' Oeste | -26.76 | 571 | 20 | SAF | 41197 | 6973 | - | 407 | 0.1799 |
| SLFT1274 | *Phyllomedusa* | *Phyllomedusa tetraploidea* | Santa Catarina | São Miguel d' Oeste | -26.76 | 571 | 20 | SAF | 41197 | 3481 | + | 330 | 0.1135 |
| SLFT1278 | *Phyllomedusa* | *Phyllomedusa tetraploidea* | Santa Catarina | São Miguel d' Oeste | -26.76 | 571 | 20 | SAF | 41197 | 2182 | + | 53 | 0.0267 |
| SLFT1289 | *Phyllomedusa* | *Phyllomedusa tetraploidea* | Santa Catarina | São Miguel d' Oeste | -26.76 | 571 | 20 | SAF | 41197 | 20168 | - | 16 | 0.0606 |
| SLFT1292 | *Phyllomedusa* | *Phyllomedusa tetraploidea* | Santa Catarina | São Miguel d' Oeste | -26.76 | 571 | 20 | SAF | 41197 | 40746 | - | 9 | 0.1463 |
| SLFT1316 | *Phyllomedusa* | *Phyllomedusa tetraploidea* | Santa Catarina | São Miguel d' Oeste | -26.76 | 571 | 20 | SAF | 41197 | 12645 | - | 35 | 0.0707 |
| SLFT1325 | *Phyllomedusa* | *Phyllomedusa tetraploidea* | Santa Catarina | São Miguel d' Oeste | -26.76 | 571 | 20 | SAF | 41197 | 1124 | - | 18 | 0.0603 |
| SLFT1378 | *Phyllomedusa* | *Phyllomedusa tetraploidea* | Santa Catarina | São Miguel d' Oeste | -26.76 | 571 | 20 | SAF | 41197 | 2332 | NA | 185 | 0.0895 |
| SLFT1389 | *Phyllomedusa* | *Phyllomedusa tetraploidea* | Santa Catarina | São Miguel d' Oeste | -26.76 | 571 | 20 | SAF | 41197 | 6743 | NA | 78 | 0.0296 |
| SLFT1972 | *Phyllomedusa* | *Phyllomedusa burmeisteri* | Bahia | Camacan | -15.38 | 376 | 7 | NAF | 41647 | 20863 | - | 27 | 0.1440 |
| SLFT1985 | *Phyllomedusa* | *Phyllomedusa burmeisteri* | Bahia | Camacan | -15.38 | 376 | 7 | NAF | 41647 | 22549 | - | 84 | 0.1006 |
| SLFT1989 | *Phyllomedusa* | *Phyllomedusa burmeisteri* | Bahia | Camacan | -15.38 | 376 | 7 | NAF | 41647 | 15819 | - | 8 | 0.3101 |
| SLFT1993 | *Phyllomedusa* | *Phyllomedusa burmeisteri* | Bahia | Camacan | -15.38 | 376 | 7 | NAF | 41647 | 20727 | - | 54 | 0.1438 |
| SLFT2055 | *Phyllomedusa* | *Phyllomedusa burmeisteri* | Espírito Santo | Linhares | -19.14 | 56 | 8 | CAF | 41649 | 4349 | - | 16 | 0.1558 |
| SLFT2057 | *Phyllomedusa* | *Phyllomedusa burmeisteri* | Espírito Santo | Linhares | -19.14 | 56 | 8 | CAF | 41649 | 2283 | - | 7 | 0.2064 |
| SLFT2061 | *Phyllomedusa* | *Phyllomedusa burmeisteri* | Espírito Santo | Linhares | -19.14 | 56 | 8 | CAF | 41649 | 6921 | - | 57 | 0.2067 |
| SLFT2114 | *Phyllomedusa* | *Phyllomedusa burmeisteri* | Espírito Santo | Linhares | -19.14 | 56 | 8 | CAF | 41649 | 9809 | - | 46 | 0.1606 |
| SLFT5118 | *Phyllomedusa* | *Phyllomedusa bahiana* | Sergipe | Areia Branca | -10.76 | 373 | 5 | NAF | 42165 | 95335 | - | 4 | 0.3347 |
| SLFT1917 | *Pithecopus* | *Pithecopus nordestinus* | Alagoas | Murici | -9.21 | 233 | 4 | NAF | 41520 | 3326 | + | 73 | 0.0869 |
| SLFT1948 | *Pithecopus* | *Pithecopus nordestinus* | Bahia | Camacan | -15.38 | 376 | 7 | NAF | 41647 | 8033 | - | 32 | 0.0860 |
| SLFT1980 | *Pithecopus* | *Pithecopus nordestinus* | Bahia | Camacan | -15.38 | 376 | 7 | NAF | 41647 | 13480 | - | 39 | 0.1915 |
| SLFT2282 | *Pithecopus* | *Pithecopus rhodei* | Espírito Santo | Vargem Alta | -20.48 | 1148 | 10 | CAF | 41653 | 10857 | - | 13 | 0.1528 |
| SLFT2290 | *Pithecopus* | *Pithecopus rhodei* | Espírito Santo | Vargem Alta | -20.48 | 1148 | 10 | CAF | 41653 | 18979 | - | 25 | 0.1504 |
| SLFT2291 | *Pithecopus* | *Pithecopus rhodei* | Espírito Santo | Vargem Alta | -20.48 | 1148 | 10 | CAF | 41653 | 28521 | - | 19 | 0.0789 |
| SLFT2292 | *Pithecopus* | *Pithecopus rhodei* | Espírito Santo | Vargem Alta | -20.48 | 1148 | 10 | CAF | 41653 | 15000 | - | 33 | 0.1110 |
| SLFT2295 | *Pithecopus* | *Pithecopus rhodei* | Espírito Santo | Vargem Alta | -20.48 | 1148 | 10 | CAF | 41653 | 21653 | - | 26 | 0.0862 |
| SLFT2313 | *Pithecopus* | *Pithecopus rhodei* | Espírito Santo | Vargem Alta | -20.48 | 1148 | 10 | CAF | 41653 | 23133 | - | 41 | 0.0832 |
| SLFT2322 | *Pithecopus* | *Pithecopus rhodei* | Espírito Santo | Vargem Alta | -20.48 | 1148 | 10 | CAF | 41653 | 27257 | - | 4 | 0.1457 |
| SLFT0116 | *Scinax* | *Scinax perereca* | São Paulo | Iguape | -24.50 | 16 | 17 | CAF | 2013 | 62764 | + | 149 | 0.1219 |
| SLFT0122 | *Scinax* | *Scinax argyreornatus* | São Paulo | Iguape | -24.50 | 16 | 17 | CAF | 2013 | 62764 | + | 82 | 0.0432 |
| SLFT0165 | *Scinax* | *Scinax* cf. *littoralis* | São Paulo | Ubatuba | -23.36 | 76 | 14 | CAF | 40840 | 40745 | + | 33 | 0.0864 |
| SLFT0166 | *Scinax* | *Scinax* cf. *littoralis* | São Paulo | Ubatuba | -23.36 | 76 | 14 | CAF | 40840 | 67201 | - | 18 | 0.0545 |
| SLFT0175 | *Scinax* | *Scinax* cf. *alter* | São Paulo | Ubatuba | -23.36 | 76 | 14 | CAF | 40840 | 48955 | + | 31 | 0.0583 |
| SLFT0177 | *Scinax* | *Scinax* cf*. alter* | São Paulo | Ubatuba | -23.36 | 76 | 14 | CAF | 40840 | 38211 | + | 40 | 0.1298 |
| SLFT0533 | *Scinax* | *Scinax fuscovarius* | São Paulo | Iporanga | -24.60 | 412 | 18 | CAF | 41281 | 26434 | - | 25 | 0.0795 |
| SLFT0543 | *Scinax* | *Scinax imbegue* | São Paulo | Iporanga | -24.60 | 412 | 18 | CAF | 41281 | 12580 | - | 136 | 0.0824 |
| SLFT0549 | *Scinax* | *Scinax fuscovarius* | São Paulo | Iporanga | -24.60 | 412 | 18 | CAF | 41281 | 9245 | + | 121 | 0.0390 |
| SLFT0558 | *Scinax* | *Scinax fuscovarius* | São Paulo | Iporanga | -24.60 | 412 | 18 | CAF | 41281 | 44290 | + | 34 | 0.1051 |
| SLFT0560 | *Scinax* | *Scinax fuscovarius* | São Paulo | Iporanga | -24.60 | 412 | 18 | CAF | 41281 | 213110 | + | 7 | 0.2000 |
| SLFT0564 | *Scinax* | *Scinax fuscovarius* | São Paulo | Iporanga | -24.60 | 412 | 18 | CAF | 41281 | 40815 | + | 37 | 0.0711 |
| SLFT0577 | *Scinax* | *Scinax imbegue* | São Paulo | Iporanga | -24.60 | 412 | 18 | CAF | 41281 | 48470 | - | 13 | 0.1484 |
| SLFT0578 | *Scinax* | *Scinax imbegue* | São Paulo | Iporanga | -24.60 | 412 | 18 | CAF | 41281 | 8600 | + | 25 | 0.0828 |
| SLFT0598 | *Scinax* | *Scinax imbegue* | São Paulo | Iporanga | -24.60 | 412 | 18 | CAF | 41281 | 11655 | - | 69 | 0.0482 |
| SLFT0620 | *Scinax* | *Scinax imbegue* | São Paulo | Iporanga | -24.60 | 412 | 18 | CAF | 41281 | 5779 | - | 92 | 0.0406 |
| SLFT0621 | *Scinax* | *Scinax imbegue* | São Paulo | Iporanga | -24.60 | 412 | 18 | CAF | 41281 | 6797 | - | 45 | 0.0961 |
| SLFT0700 | *Scinax* | *Scinax imbegue* | Paraná | Morretes | -25.40 | 426 | 19 | SAF | 41283 | 3252 | + | 38 | 0.0706 |
| SLFT0711 | *Scinax* | *Scinax imbegue* | Paraná | Morretes | -25.40 | 426 | 19 | SAF | 41283 | 23955 | - | 14 | 0.1324 |
| SLFT0727 | *Scinax* | *Scinax imbegue* | Paraná | Morretes | -25.40 | 426 | 19 | SAF | 41283 | 11016 | - | 21 | 0.2801 |
| SLFT0728 | *Scinax* | *Scinax tymbamirim* | Paraná | Morretes | -25.40 | 426 | 19 | SAF | 41283 | 49520 | NA | 24 | 0.0800 |
| SLFT0850 | *Scinax* | *Scinax* cf. *perereca* | Santa Catarina | Rancho Queimado | -27.67 | 959 | 22 | SAF | 41284 | 1365 | + | 151 | 0.1683 |
| SLFT0882 | *Scinax* | *Scinax* gr*. ruber* | Santa Catarina | Pomerode | -26.77 | 228 | 21 | SAF | 41285 | 1879 | + | 102 | 0.0361 |
| SLFT0978 | *Scinax* | *Scinax v-signatus* | Rio de Janeiro | Teresópolis | -22.46 | 1487 | 12 | CAF | 41635 | 1246 | + | 60 | 0.0714 |
| SLFT0983 | *Scinax* | *Scinax v-signatus* | Rio de Janeiro | Teresópolis | -22.46 | 1487 | 12 | CAF | 41635 | 111213 | - | 7 | 0.3803 |
| SLFT0985 | *Scinax* | *Scinax v-signatus* | Rio de Janeiro | Teresópolis | -22.46 | 1487 | 12 | CAF | 41635 | 6712 | + | 6 | 0.4537 |
| SLFT1000 | *Scinax* | *Scinax albicans* | Rio de Janeiro | Teresópolis | -22.46 | 1487 | 12 | CAF | 41635 | 52505 | - | 33 | 0.0386 |
| SLFT1030 | *Scinax* | *Scinax hayii* | Rio de Janeiro | Teresópolis | -22.46 | 1487 | 12 | CAF | 41635 | 44161 | + | 54 | 0.0323 |
| SLFT1031 | *Scinax* | *Scinax hayii* | Rio de Janeiro | Teresópolis | -22.46 | 1487 | 12 | CAF | 41635 | 17012 | + | 43 | 0.0421 |
| SLFT1037 | *Scinax* | *Scinax hayii* | Rio de Janeiro | Teresópolis | -22.46 | 1487 | 12 | CAF | 41635 | 45943 | - | 13 | 0.0877 |
| SLFT1040 | *Scinax* | *Scinax hayii* | Rio de Janeiro | Teresópolis | -22.46 | 1487 | 12 | CAF | 41635 | 48560 | - | 74 | 0.0424 |
| SLFT1052 | *Scinax* | *Scinax hayii* | Rio de Janeiro | Teresópolis | -22.46 | 1487 | 12 | CAF | 41635 | 26603 | + | 90 | 0.0704 |
| SLFT1054 | *Scinax* | *Scinax hayii* | Rio de Janeiro | Teresópolis | -22.46 | 1487 | 12 | CAF | 41635 | 33660 | - | 75 | 0.0373 |
| SLFT1055 | *Scinax* | *Scinax hayii* | Rio de Janeiro | Teresópolis | -22.46 | 1487 | 12 | CAF | 41635 | 15715 | - | 71 | 0.0221 |
| SLFT1062 | *Scinax* | *Scinax v-signatus* | Rio de Janeiro | Teresópolis | -22.46 | 1487 | 12 | CAF | 41635 | 19628 | - | 91 | 0.0284 |
| SLFT1064 | *Scinax* | *Scinax fuscovarius* | São Paulo | Pedro de Toledo | -24.32 | 209 | 16 | CAF | 41328 | 35251 | - | 34 | 0.0678 |
| SLFT1065 | *Scinax* | *Scinax cuspidatus* | São Paulo | Pedro de Toledo | -24.32 | 209 | 16 | CAF | 41328 | 33638 | - | 33 | 0.0896 |
| SLFT1066 | *Scinax* | *Scinax cuspidatus* | São Paulo | Pedro de Toledo | -24.32 | 209 | 16 | CAF | 41328 | 26441 | - | 49 | 0.0711 |
| SLFT1068 | *Scinax* | *Scinax cuspidatus* | São Paulo | Pedro de Toledo | -24.32 | 209 | 16 | CAF | 41328 | 28833 | - | 31 | 0.0856 |
| SLFT1069 | *Scinax* | *Scinax cuspidatus* | São Paulo | Pedro de Toledo | -24.32 | 209 | 16 | CAF | 41328 | 37098 | - | 34 | 0.0848 |
| SLFT1070 | *Scinax* | *Scinax cuspidatus* | São Paulo | Pedro de Toledo | -24.32 | 209 | 16 | CAF | 41328 | 56900 | - | 35 | 0.0705 |
| SLFT1071 | *Scinax* | *Scinax cuspidatus* | São Paulo | Pedro de Toledo | -24.32 | 209 | 16 | CAF | 41328 | 50614 | - | 38 | 0.0834 |
| SLFT1093 | *Scinax* | *Scinax fuscovarius* | São Paulo | Pedro de Toledo | -24.32 | 209 | 16 | CAF | 41328 | 49172 | + | 63 | 0.0532 |
| SLFT1095 | *Scinax* | *Scinax perereca* | São Paulo | Pedro de Toledo | -24.32 | 209 | 16 | CAF | 41328 | 43010 | - | 51 | 0.0550 |
| SLFT1101 | *Scinax* | *Scinax cuspidatus* | São Paulo | Iguape | -24.50 | 16 | 17 | CAF | 2013 | 48511 | + | 37 | 0.0500 |
| SLFT1262 | *Scinax* | *Scinax aromothylla* | Santa Catarina | São Miguel d' Oeste | -26.76 | 571 | 20 | SAF | 41197 | 4848 | - | 322 | 0.3968 |
| SLFT1287 | *Scinax* | *Scinax fuscovarius* | Santa Catarina | São Miguel d' Oeste | -26.76 | 571 | 20 | SAF | 41197 | 15002 | + | 22 | 0.0730 |
| SLFT1362 | *Scinax* | *Scinax aromothylla* | Santa Catarina | São Miguel d' Oeste | -26.76 | 571 | 20 | SAF | 41197 | 1624 | NA | 152 | 0.0888 |
| SLFT1380 | *Scinax* | *Scinax fuscovarius* | Santa Catarina | São Miguel d' Oeste | -26.76 | 571 | 20 | SAF | 41197 | 15166 | NA | 282 | 0.0430 |
| SLFT1387 | *Scinax* | *Scinax fuscovarius* | Santa Catarina | São Miguel d' Oeste | -26.76 | 571 | 20 | SAF | 41197 | 9674 | NA | 79 | 0.1225 |
| SLFT1484 | *Scinax* | *Scinax cuspidatus* | São Paulo | Itariri | -24.31 | 180 | 15 | CAF | 41329 | 24120 | - | 32 | 0.0790 |
| SLFT1485 | *Scinax* | *Scinax cuspidatus* | São Paulo | Itariri | -24.31 | 180 | 15 | CAF | 41329 | 49112 | - | 83 | 0.0324 |
| SLFT1486 | *Scinax* | *Scinax cuspidatus* | São Paulo | Itariri | -24.31 | 180 | 15 | CAF | 41329 | 13214 | - | 58 | 0.0383 |
| SLFT1487 | *Scinax* | *Scinax cuspidatus* | São Paulo | Itariri | -24.31 | 180 | 15 | CAF | 41329 | 32110 | - | 72 | 0.0356 |
| SLFT1488 | *Scinax* | *Scinax cuspidatus* | São Paulo | Itariri | -24.31 | 180 | 15 | CAF | 41329 | 27980 | - | 101 | 0.0655 |
| SLFT1489 | *Scinax* | *Scinax cuspidatus* | São Paulo | Itariri | -24.31 | 180 | 15 | CAF | 41329 | 40343 | - | 101 | 0.0396 |
| SLFT1502 | *Scinax* | *Scinax alter* | Rio Grande do Sul | Torres | -29.33 | 9 | 23 | SAF | 41356 | 34172 | - | 475 | 0.3015 |
| SLFT1504 | *Scinax* | *Scinax alter* | Rio Grande do Sul | Torres | -29.33 | 9 | 23 | SAF | 41356 | 22838 | - | 423 | 0.2886 |
| SLFT1507 | *Scinax* | *Scinax alter* | Rio Grande do Sul | Torres | -29.33 | 9 | 23 | SAF | 41356 | 22292 | + | 309 | 0.0690 |
| SLFT1510 | *Scinax* | *Scinax alter* | Rio Grande do Sul | Torres | -29.33 | 9 | 23 | SAF | 41356 | 19592 | + | 572 | 0.4417 |
| SLFT1511 | *Scinax* | *Scinax alter* | Rio Grande do Sul | Torres | -29.33 | 9 | 23 | SAF | 41356 | 29635 | + | 528 | 0.0685 |
| SLFT1512 | *Scinax* | *Scinax alter* | Rio Grande do Sul | Torres | -29.33 | 9 | 23 | SAF | 41356 | 17008 | + | 368 | 0.1456 |
| SLFT1514 | *Scinax* | *Scinax alter* | Rio Grande do Sul | Torres | -29.33 | 9 | 23 | SAF | 41356 | 32346 | + | 375 | 0.2054 |
| SLFT1567 | *Scinax* | *Scinax hayii* | Rio de Janeiro | Teresópolis | -22.46 | 1487 | 12 | CAF | 41411 | 1793 | + | 32 | 0.0532 |
| SLFT1580 | *Scinax* | *Scinax albicans* | Rio de Janeiro | Teresópolis | -22.46 | 1487 | 12 | CAF | 41411 | 4868 | + | 217 | 0.0771 |
| SLFT1584 | *Scinax* | *Scinax v-signatus* | Rio de Janeiro | Teresópolis | -22.46 | 1487 | 12 | CAF | 41411 | 3679 | - | 180 | 0.0310 |
| SLFT1918 | *Scinax* | *Scinax nebulosus* | Alagoas | Murici | -9.21 | 233 | 4 | NAF | 41520 | 3978 | + | 47 | 0.1121 |
| SLFT1921 | *Scinax* | *Scinax nebulosus* | Alagoas | Murici | -9.21 | 233 | 4 | NAF | 41520 | 10949 | - | 55 | 0.1098 |
| SLFT1970 | *Scinax* | *Scinax eurydice* | Bahia | Camacan | -15.38 | 376 | 7 | NAF | 41647 | 10342 | - | 114 | 0.0449 |
| SLFT2006 | *Scinax* | *Scinax juncae* | Bahia | Camacan | -15.38 | 376 | 7 | NAF | 41647 | 5831 | - | 31 | 0.0824 |
| SLFT2007 | *Scinax* | *Scinax juncae* | Bahia | Camacan | -15.38 | 376 | 7 | NAF | 41647 | 10854 | - | 26 | 0.1571 |
| SLFT2008 | *Scinax* | *Scinax juncae* | Bahia | Camacan | -15.38 | 376 | 7 | NAF | 41647 | 31104 | - | 28 | 0.0603 |
| SLFT2012 | *Scinax* | *Scinax strigilatus* | Bahia | Camacan | -15.38 | 376 | 7 | NAF | 41647 | 10011 | - | 28 | 0.1255 |
| SLFT2014 | *Scinax* | *Scinax strigilatus* | Bahia | Camacan | -15.38 | 376 | 7 | NAF | 41647 | 9309 | + | 32 | 0.0767 |
| SLFT2015 | *Scinax* | *Scinax strigilatus* | Bahia | Camacan | -15.38 | 376 | 7 | NAF | 41647 | 8570 | + | 48 | 0.1333 |
| SLFT2016 | *Scinax* | *Scinax strigilatus* | Bahia | Camacan | -15.38 | 376 | 7 | NAF | 41647 | 12512 | - | 24 | 0.1922 |
| SLFT2028 | *Scinax* | *Scinax x-signatus* | Bahia | Camacan | -15.38 | 376 | 7 | NAF | 41647 | 3716 | - | 36 | 0.1717 |
| SLFT2094 | *Scinax* | *Scinax argyreornatus* | Espírito Santo | Linhares | -19.14 | 56 | 8 | CAF | 41649 | 1251 | - | 29 | 0.1035 |
| SLFT2146 | *Scinax* | *Scinax argyreornatus* | Espírito Santo | Linhares | -19.14 | 56 | 8 | CAF | 41649 | 4474 | - | 84 | 0.1691 |
| SLFT2158 | *Scinax* | *Scinax flavogutattus* | Rio de Janeiro | Teresópolis | -22.46 | 1487 | 12 | CAF | 41606 | 2636 | - | 91 | 0.0523 |
| SLFT2305 | *Scinax* | *Scinax alter* | Espírito Santo | Vargem Alta | -20.48 | 1148 | 10 | CAF | 41653 | 3590 | + | 25 | 0.2185 |
| SLFT2317 | *Scinax* | *Scinax x-signatus* | Espírito Santo | Vargem Alta | -20.48 | 1148 | 10 | CAF | 41653 | 16559 | - | 97 | 0.0237 |
| SLFT2323 | *Scinax* | *Scinax heyeri* | Espírito Santo | Vargem Alta | -20.48 | 1148 | 10 | CAF | 41653 | 1587 | - | 21 | 0.2791 |
| SLFT2333 | *Scinax* | *Scinax x-signatus* | Espírito Santo | Vargem Alta | -20.48 | 1148 | 10 | CAF | 41653 | 15663 | - | 59 | 0.0599 |
| SLFT2342 | *Scinax* | *Scinax heyeri* | Espírito Santo | Vargem Alta | -20.48 | 1148 | 10 | CAF | 41653 | 6694 | + | 42 | 0.1261 |
| SLFT2356 | *Scinax* | *Scinax x-signatus* | Espírito Santo | Vargem Alta | -20.48 | 1148 | 10 | CAF | 41653 | 3386 | - | 43 | 0.1652 |
| SLFT2368 | *Scinax* | *Scinax alter* | Espírito Santo | Vargem Alta | -20.48 | 1148 | 10 | CAF | 41653 | 8780 | NA | 9 | 0.2357 |
| SLFT2371 | *Scinax* | *Scinax x-signatus* | Espírito Santo | Vargem Alta | -20.48 | 1148 | 10 | CAF | 41653 | 12216 | - | 40 | 0.0674 |
| SLFT2957 | *Scinax* | *Scinax* sp*.* | Paraná | Morretes | -25.40 | 426 | 19 | SAF | 41283 |  | - | 13 | 0.0775 |
| SLFT3062 | *Scinax* | *Scinax albicans* | Rio de Janeiro | Teresópolis | -22.46 | 1487 | 12 | CAF | 41795 | 2672 | - | 24 | 0.1608 |
| SLFT3063 | *Scinax* | *Scinax albicans* | Rio de Janeiro | Teresópolis | -22.46 | 1487 | 12 | CAF | 41795 | 2458 | - | 4 | 0.6128 |
| SLFT3070 | *Scinax* | *Scinax hayii* | Rio de Janeiro | Teresópolis | -22.46 | 1487 | 12 | CAF | 41795 | 1454 | - | 15 | 0.2012 |
| SLFT4791 | *Scinax* | *Scinax nebulosus* | Rio Grande do Norte | Baía Formosa | -6.41 | 23 | 1 | NAF | 42157 | 45489 | - | 83 | 0.2608 |
| SLFT4803 | *Scinax* | *Scinax nebulosus* | Rio Grande do Norte | Baía Formosa | -6.41 | 23 | 1 | NAF | 42157 | 48967 | - | 37 | 0.0650 |
| SLFT4816 | *Scinax* | *Scinax nebulosus* | Rio Grande do Norte | Baía Formosa | -6.41 | 23 | 1 | NAF | 42157 | 33262 | - | 71 | 0.0768 |
| SLFT4818 | *Scinax* | *Scinax nebulosus* | Rio Grande do Norte | Baía Formosa | -6.41 | 23 | 1 | NAF | 42157 | 25480 | + | 27 | 0.2033 |
| SLFT4819 | *Scinax* | *Scinax nebulosus* | Rio Grande do Norte | Baía Formosa | -6.41 | 23 | 1 | NAF | 42157 | 61132 | - | 45 | 0.1310 |
| SLFT4821 | *Scinax* | *Scinax nebulosus* | Rio Grande do Norte | Baía Formosa | -6.41 | 23 | 1 | NAF | 42157 | 23956 | - | 102 | 0.1013 |
| SLFT4824 | *Scinax* | *Scinax nebulosus* | Rio Grande do Norte | Baía Formosa | -6.41 | 23 | 1 | NAF | 42157 | 16398 | - | 75 | 0.1707 |
| SLFT4825 | *Scinax* | *Scinax nebulosus* | Rio Grande do Norte | Baía Formosa | -6.41 | 23 | 1 | NAF | 42157 | 15683 | - | 136 | 0.0326 |
| SLFT4828 | *Scinax* | *Scinax nebulosus* | Rio Grande do Norte | Baía Formosa | -6.41 | 23 | 1 | NAF | 42157 | 7568 | - | 70 | 0.3734 |
| SLFT4831 | *Scinax* | *Scinax nebulosus* | Rio Grande do Norte | Baía Formosa | -6.41 | 23 | 1 | NAF | 42157 | 56392 | - | 73 | 0.0833 |
| SLFT4833 | *Scinax* | *Scinax nebulosus* | Rio Grande do Norte | Baía Formosa | -6.41 | 23 | 1 | NAF | 42157 | 1753 | - | 30 | 0.0477 |
| SLFT4834 | *Scinax* | *Scinax nebulosus* | Rio Grande do Norte | Baía Formosa | -6.41 | 23 | 1 | NAF | 42157 | 13843 | - | 75 | 0.1240 |
| SLFT4836 | *Scinax* | *Scinax nebulosus* | Rio Grande do Norte | Baía Formosa | -6.41 | 23 | 1 | NAF | 42157 | 38375 | - | 24 | 0.2104 |
| SLFT4837 | *Scinax* | *Scinax nebulosus* | Rio Grande do Norte | Baía Formosa | -6.41 | 23 | 1 | NAF | 42157 | 26617 | - | 20 | 0.2112 |
| SLFT4840 | *Scinax* | *Scinax nebulosus* | Rio Grande do Norte | Baía Formosa | -6.41 | 23 | 1 | NAF | 42157 | 39762 | - | 26 | 0.1134 |
| SLFT5031 | *Scinax* | *Scinax fuscovarius* | Pernambuco | Paulista | -7.93 | 30 | 3 | NAF | 42164 | 49224 | - | 118 | 0.0493 |
| SLFT5122 | *Scinax* | *Scinax cretatus* | Sergipe | Areia Branca | -10.76 | 373 | 5 | NAF | 42165 | 43423 | - | 22 | 0.1407 |
| SLFT5139 | *Scinax* | *Scinax pachycrus* | Sergipe | Areia Branca | -10.76 | 373 | 5 | NAF | 42165 | 95337 | - | 7 | 0.1565 |
| SLFT5159 | *Scinax* | *Scinax pachycrus* | Sergipe | Areia Branca | -10.76 | 373 | 5 | NAF | 42165 | 17219 | - | 94 | 0.0770 |
| SLFT5160 | *Scinax* | *Scinax pachycrus* | Sergipe | Areia Branca | -10.76 | 373 | 5 | NAF | 42165 | 29441 | - | 6 | 0.1551 |
| SLFT5161 | *Scinax* | *Scinax pachycrus* | Sergipe | Areia Branca | -10.76 | 373 | 5 | NAF | 42165 | 35942 | - | 60 | 0.1552 |
| SLFT5178 | *Scinax* | *Scinax pachycrus* | Sergipe | Areia Branca | -10.76 | 373 | 5 | NAF | 42165 | 32015 | - | 140 | 0.1631 |
| SLFT5289 | *Scinax* | *Scinax fuscomarginatus* | Bahia | Mata de São João | -12.56 | 41 | 6 | NAF | 42168 | 17135 | - | 58 | 0.0885 |
| SLFT5291 | *Scinax* | *Scinax fuscomarginatus* | Bahia | Mata de São João | -12.56 | 41 | 6 | NAF | 42168 | 30969 | - | 101 | 0.0843 |
| SLFT5297 | *Scinax* | *Scinax fuscomarginatus* | Bahia | Mata de São João | -12.56 | 41 | 6 | NAF | 42168 | 22845 | - | 53 | 0.1137 |
| SLFT5298 | *Scinax* | *Scinax fuscomarginatus* | Bahia | Mata de São João | -12.56 | 41 | 6 | NAF | 42168 | 19791 | - | 62 | 0.1388 |
| SLFT0569 | *Sphaenorhynchus* | *Sphaenorhynchus caramaschii* | São Paulo | Iporanga | -24.60 | 412 | 18 | CAF | 41281 | 18578 | - | 9 | 0.0896 |
| SLFT0573 | *Sphaenorhynchus* | *Sphaenorhynchus caramaschii* | São Paulo | Iporanga | -24.60 | 412 | 18 | CAF | 41281 | 36030 | - | 53 | 0.0368 |
| SLFT0580 | *Sphaenorhynchus* | *Sphaenorhynchus caramaschii* | São Paulo | Iporanga | -24.60 | 412 | 18 | CAF | 41281 | 26425 | - | 20 | 0.1483 |
| SLFT0582 | *Sphaenorhynchus* | *Sphaenorhynchus caramaschii* | São Paulo | Iporanga | -24.60 | 412 | 18 | CAF | 41281 | 32660 | - | 46 | 0.0377 |
| SLFT0587 | *Sphaenorhynchus* | *Sphaenorhynchus caramaschii* | São Paulo | Iporanga | -24.60 | 412 | 18 | CAF | 41281 | 21577 | - | 33 | 0.0727 |
| SLFT0591 | *Sphaenorhynchus* | *Sphaenorhynchus caramaschii* | São Paulo | Iporanga | -24.60 | 412 | 18 | CAF | 41281 | 7860 | - | 34 | 0.1127 |
| SLFT0601 | *Sphaenorhynchus* | *Sphaenorhynchus caramaschii* | São Paulo | Iporanga | -24.60 | 412 | 18 | CAF | 41281 | 110280 | - | 6 | 0.3347 |
| SLFT0608 | *Sphaenorhynchus* | *Sphaenorhynchus caramaschii* | São Paulo | Iporanga | -24.60 | 412 | 18 | CAF | 41281 | 18854 | + | 49 | 0.1006 |
| SLFT0610 | *Sphaenorhynchus* | *Sphaenorhynchus caramaschii* | São Paulo | Iporanga | -24.60 | 412 | 18 | CAF | 41281 | 66946 | + | 9 | 0.2214 |
| SLFT0623 | *Sphaenorhynchus* | *Sphaenorhynchus caramaschii* | São Paulo | Iporanga | -24.60 | 412 | 18 | CAF | 41281 | 11097 | - | 51 | 0.0384 |
| SLFT0632 | *Sphaenorhynchus* | *Sphaenorhynchus caramaschii* | São Paulo | Iporanga | -24.60 | 412 | 18 | CAF | 41281 | 14569 | + | 25 | 0.0670 |
| SLFT0633 | *Sphaenorhynchus* | *Sphaenorhynchus caramaschii* | São Paulo | Iporanga | -24.60 | 412 | 18 | CAF | 41281 | 3993 | - | 40 | 0.1877 |
| SLFT0637 | *Sphaenorhynchus* | *Sphaenorhynchus caramaschii* | São Paulo | Iporanga | -24.60 | 412 | 18 | CAF | 41281 | 14113 | - | 24 | 0.1883 |
| SLFT0775 | *Sphaenorhynchus* | *Sphaenorhynchus surdus* | Santa Catarina | Rancho Queimado | -27.67 | 959 | 22 | SAF | 41284 | 8077 | - | 48 | 0.0500 |
| SLFT0780 | *Sphaenorhynchus* | *Sphaenorhynchus surdus* | Santa Catarina | Rancho Queimado | -27.67 | 959 | 22 | SAF | 41284 | 15160 | + | 109 | 0.0357 |
| SLFT1505 | *Sphaenorhynchus* | *Sphaenorhynchus surdus* | Rio Grande do Sul | Torres | -29.33 | 9 | 23 | SAF | 41356 | 26087 | + | 463 | 0.0246 |
| SLFT1508 | *Sphaenorhynchus* | *Sphaenorhynchus surdus* | Rio Grande do Sul | Torres | -29.33 | 9 | 23 | SAF | 41356 | 31994 | + | 183 | 0.0620 |
| SLFT1509 | *Sphaenorhynchus* | *Sphaenorhynchus surdus* | Rio Grande do Sul | Torres | -29.33 | 9 | 23 | SAF | 41356 | 32016 | + | 310 | 0.3209 |
| SLFT1513 | *Sphaenorhynchus* | *Sphaenorhynchus surdus* | Rio Grande do Sul | Torres | -29.33 | 9 | 23 | SAF | 41356 | 13981 | + | 418 | 0.0170 |
| SLFT1515 | *Sphaenorhynchus* | *Sphaenorhynchus surdus* | Rio Grande do Sul | Torres | -29.33 | 9 | 23 | SAF | 41356 | 26628 | + | 265 | 0.2160 |
| SLFT1516 | *Sphaenorhynchus* | *Sphaenorhynchus surdus* | Rio Grande do Sul | Torres | -29.33 | 9 | 23 | SAF | 41356 | 31799 | + | 228 | 0.0406 |
| SLFT1517 | *Sphaenorhynchus* | *Sphaenorhynchus surdus* | Rio Grande do Sul | Torres | -29.33 | 9 | 23 | SAF | 41356 | 29427 | + | 349 | 0.1801 |
| SLFT1518 | *Sphaenorhynchus* | *Sphaenorhynchus surdus* | Rio Grande do Sul | Torres | -29.33 | 9 | 23 | SAF | 41356 | 36243 | - | 81 | 0.1390 |
| SLFT1519 | *Sphaenorhynchus* | *Sphaenorhynchus surdus* | Rio Grande do Sul | Torres | -29.33 | 9 | 23 | SAF | 41356 | 38375 | + | 199 | 0.0349 |
| SLFT1523 | *Sphaenorhynchus* | *Sphaenorhynchus surdus* | Rio Grande do Sul | Torres | -29.33 | 9 | 23 | SAF | 41356 | 23983 | + | 215 | 0.0821 |

**Supplementary Table 2** Results of initial single-variable correlations of latitude, elevation, Bd infection intensity, and bioclimatic measures with #OTUs, using simple linear regression models as an assessment of the strength of correlation of each predictor to the response of interest. N = 819 except for Bd infection intensity with removed zero and maximum extreme values (N =186). All variables were scaled before analyses.

| **Independent variable** | **k** | **R²** | **P** |
| --- | --- | --- | --- |
| Elevation | -3.84 | 0.002 | **0.197** |
| Latitude | -13.66 | 0.026 | **<0.001** |
| Bd infection intensity | 5.49 | 0.004 | 0.061 |
| Bd infection intensity  (zeros and maximum extreme values removed) | 0.02 | 0.015 | 0.081 |
| Annual Mean Temperature (BIO1) | -13.05 | 0.024 | **<0.001** |
| Mean Diurnal Range (BIO 2) | 6.05 | 0.005 | **0.042** |
| Isothermality (BIO 3) | -9.62 | 0.013 | **0.001** |
| Temperature Seasonality (BIO 4) | 12.14 | 0.020 | **<0.001** |
| Maximum Temperature of Warmest Month (BIO 5) | -7.31 | 0.007 | **0.014** |
| Minimum Temperature of Coldest Month (BIO 6) | -11.16 | 0.017 | **<0.001** |
| Annual Temperature Range (BIO 7) | 9.42 | 0.012 | **0.001** |
| Mean Temperature of Wettest Quarter (BIO 8) | -18.42 | 0.047 | **<0.001** |
| Mean Temperature of Driest Quarter (BIO 9) | -12.83 | 0.023 | **<0.001** |
| Mean Temperature of Warmest Quarter (BIO 10) | -10.43 | 0.015 | **<0.001** |
| Mean Temperature of Coldest Quarter (BIO 11) | -14.18 | 0.028 | **<0.001** |
| Annual Precipitation (BIO 12) | 10.01 | 0.014 | **0.001** |
| Precipitation of Wettest Month (BIO 13) | -10.78 | 0.016 | **<0.001** |
| Precipitation of Driest Month (BIO 14) | 28.90 | 0.115 | **<0.001** |
| Precipitation Seasonality (BIO 15) | -26.60 | 0.098 | **<0.001** |
| Precipitation of Wettest Quarter (BIO 16) | -13.11 | 0.024 | **<0.001** |
| Precipitation of Driest Quarter (BIO 17) | 28.32 | 0.111 | **<0.001** |
| Precipitation of Warmest Quarter (BIO 18) | -6.29 | 0.005 | **0.034** |
| Precipitation of Coldest Quarter (BIO 19) | 14.46 | 0.029 | **<0.001** |

**Supplementary Table 3** Results of initial single-variable correlations of latitude, elevation, Bd infection intensity, and bioclimatic measures with Simpson’s evenness, using simple linear regression models as an assessment of the strength of correlation of each predictor to the response of interest. N = 819 except for Bd infection intensity with removed zero and maximum extreme values (N =186). All variables were scaled before analyses.

| **Independent variable** | **k** | **R²** | **P** |
| --- | --- | --- | --- |
| Elevation | -0.01 | 0.018 | **<0.001** |
| Latitude | 0.02 | 0.035 | **<0.001** |
| Bd infection intensity | -0.00 | 0.001 | 0.522 |
| Bd infection intensity  (zeros and maximum extreme values removed) | 0.17 | 0.210 | **<0.001** |
| Annual Mean Temperature (BIO1) | 0.02 | 0.030 | **<0.001** |
| Mean Diurnal Range (BIO 2) | -0.02 | 0.031 | **0.001** |
| Isothermality (BIO 3) | 0.01 | 0.013 | **0.001** |
| Temperature Seasonality (BIO 4) | -0.01 | 0.024 | **<0.001** |
| Maximum Temperature of Warmest Month (BIO 5) | 0.01 | 0.010 | **0.004** |
| Minimum Temperature of Coldest Month (BIO 6) | 0.02 | 0.038 | **<0.001** |
| Annual Temperature Range (BIO 7) | -0.02 | 0.033 | **<0.001** |
| Mean Temperature of Wettest Quarter (BIO 8) | 0.01 | 0.004 | 0.072 |
| Mean Temperature of Driest Quarter (BIO 9) | 0.02 | 0.034 | **<0.001** |
| Mean Temperature of Warmest Quarter (BIO 10) | 0.01 | 0.017 | **<0.001** |
| Mean Temperature of Coldest Quarter (BIO 11) | 0.02 | 0.034 | **<0.001** |
| Annual Precipitation (BIO 12) | -0.00 | 0.002 | 0.217 |
| Precipitation of Wettest Month (BIO 13) | 0.00 | 0.002 | 0.265 |
| Precipitation of Driest Month (BIO 14) | -0.01 | 0.013 | **0.001** |
| Precipitation Seasonality (BIO 15) | 0.01 | 0.007 | **0.014** |
| Precipitation of Wettest Quarter (BIO 16) | 0.00 | 0.001 | 0.307 |
| Precipitation of Driest Quarter (BIO 17) | -0.01 | 0.013 | **0.001** |
| Precipitation of Warmest Quarter (BIO 18) | -0.02 | 0.029 | **<0.001** |
| Precipitation of Coldest Quarter (BIO 19) | 0.02 | 0.029 | **<0.001** |

**Supplementary Table 4**. Results of Response screening (calculated in JMP 13.0) for all potential predictor variables considered in this study, for the response variable ‘number of observed OTUs’ from the filtered data set of amphibian skin bacterial communities. N = 819 except for Bd infection intensity (N = 791). Variables sorted by decreasing LogWorth (-log10 transformation of P-values); higher values correspond to lower P-values. FDR refers to P-values adjusted to control the false discovery rate for multiple tests, calculated using the Benjamini-Hochberg technique. Bio 1 = Annual Mean Temperature. Bio 2= Mean Diurnal Range. Bio 3 = Isothermality. Bio 4 = Temperature Seasonality. Bio 5 = Maximum Temperature of Warmest Month. Bio 6 = Minimum Temperature of Coldest Month. Bio 7 = Annual Temperature Range. Bio 8 = Mean Temperature of Wettest Quarter. Bio 9 = Mean Temperature of Driest Quarter. Bio 10 = Mean Temperature of Warmest Quarter. Bio 11 = Mean Temperature of Coldest Quarter. Bio 12 = Annual Precipitation. Bio 13 = Precipitation of Wettest Month. Bio 14 = Precipitation of Driest Month. Bio 15 = Precipitation Seasonality. Bio 16 = Precipitation of Wettest Quarter. Bio 17 = Precipitation of Driest Quarter. Bio 18 = Precipitation of Warmest Quarter. Bio 19 = Precipitation of Coldest Quarter. All variables were scaled before analyses.

| Y (response variable) | X (predictor variable) | P value | Log Worth (P value) | FDR P value | Log Worth (FDR P value) | Effect Size | F Ratio | R² |
| --- | --- | --- | --- | --- | --- | --- | --- | --- |
| **OTU** | **BIO14** | **0.000** | **22.849** | **0.000** | **21.507** | **0.672** | **106.613** | **0.115** |
| **OTU** | **BIO17** | **0.000** | **21.921** | **0.000** | **20.879** | **0.658** | **101.836** | **0.111** |
| **OTU** | **BIO15** | **0.000** | **19.310** | **0.000** | **18.445** | **0.618** | **88.549** | **0.098** |
| **OTU** | **BIO8** | **0.000** | **9.423** | **0.000** | **8.682** | **0.428** | **40.205** | **0.047** |
| **OTU** | **BIO19** | **0.000** | **6.005** | **0.000** | **5.361** | **0.336** | **24.319** | **0.029** |
| OTU | BIO11 | 0.000 | 5.797 | 0.000 | 5.233 | 0.330 | 23.370 | 0.028 |
| OTU | Latitude | 0.000 | 5.417 | 0.000 | 4.919 | 0.318 | 21.643 | 0.026 |
| OTU | BIO16 | 0.000 | 5.027 | 0.000 | 4.602 | 0.305 | 19.881 | 0.024 |
| OTU | BIO1 | 0.000 | 4.990 | 0.000 | 4.602 | 0.303 | 19.713 | 0.024 |
| OTU | BIO9 | 0.000 | 4.833 | 0.000 | 4.490 | 0.298 | 19.004 | 0.023 |
| OTU | BIO4 | 0.000 | 4.384 | 0.000 | 4.083 | 0.282 | 16.994 | 0.020 |
| OTU | BIO6 | 0.000 | 3.781 | 0.000 | 3.518 | 0.259 | 14.318 | 0.017 |
| OTU | BIO13 | 0.000 | 3.557 | 0.000 | 3.328 | 0.251 | 13.333 | 0.016 |
| OTU | BIO10 | 0.000 | 3.359 | 0.001 | 3.162 | 0.242 | 12.465 | 0.015 |
| OTU | BIO12 | 0.001 | 3.130 | 0.001 | 2.963 | 0.233 | 11.469 | 0.014 |
| OTU | BIO3 | 0.001 | 2.926 | 0.002 | 2.787 | 0.224 | 10.586 | 0.013 |
| OTU | BIO7 | 0.001 | 2.826 | 0.002 | 2.715 | 0.219 | 10.158 | 0.012 |
| OTU | BIO5 | 0.014 | 1.855 | 0.017 | 1.768 | 0.170 | 6.070 | 0.007 |
| OTU | BIO18 | 0.034 | 1.466 | 0.040 | 1.402 | 0.146 | 4.500 | 0.005 |
| OTU | BIO2 | 0.042 | 1.381 | 0.046 | 1.340 | 0.141 | 4.166 | 0.005 |
| OTU | BDinfection | 0.061 | 1.218 | 0.063 | 1.198 | 0.128 | 3.533 | 0.004 |
| OTU | Elevation | 0.197 | 0.705 | 0.197 | 0.705 | 0.089 | 1.667 | 0.002 |

**Supplementary Table 5**. Results of Predictor screening (calculated in JMP 13.0) for non-categorical predictor variables on the response variable #OTU richness, for the full data set. N = 819 except for Bd infection intensity (N=791). Bio 1 = Annual Mean Temperature. Bio 2 = Mean Diurnal Range. Bio 3 = Isothermality. Bio 4 = Temperature Seasonality. Bio 5 = Maximum Temperature of Warmest Month. Bio 6 = Minimum Temperature of Coldest Month. Bio 7 = Annual Temperature Range. Bio 8 = Mean Temperature of Wettest Quarter. Bio 9 = Mean Temperature of Driest Quarter. Bio 10 = Mean Temperature of Warmest Quarter. Bio 11 = Mean Temperature of Coldest Quarter. Bio 12 = Annual Precipitation. Bio 13 = Precipitation of Wettest Month. Bio 14 = Precipitation of Driest Month. Bio 15 = Precipitation Seasonality. Bio 16 = Precipitation of Wettest Quarter. Bio 17 = Precipitation of Driest Quarter. Bio 18 = Precipitation of Warmest Quarter. Bio 19 = Precipitation of Coldest Quarter. All variables were scaled before analyses.

| Predictor | Contribution | Portion | Rank |
| --- | --- | --- | --- |
| **BIO15** | **392892** | **0.2381** | **1** |
| **Elevation** | **195235** | **0.1183** | **2** |
| **BIO11** | **145086** | **0.0879** | **3** |
| **BIO3** | **141898** | **0.086** | **4** |
| **Latitude** | **134219** | **0.0813** | **5** |
| BIO13 | 66124 | 0.0401 | 6 |
| BIO17 | 65547 | 0.0397 | 7 |
| BIO9 | 63728 | 0.0386 | 8 |
| BIO2 | 48778 | 0.0296 | 9 |
| BIO1 | 43023 | 0.0261 | 10 |
| BIO18 | 38460 | 0.0233 | 11 |
| BIO7 | 36345 | 0.022 | 12 |
| BIO12 | 35426 | 0.0215 | 13 |
| BIO14 | 33386 | 0.0202 | 14 |
| BDstatus | 33038 | 0.02 | 15 |
| BIO16 | 32657 | 0.0198 | 16 |
| BIO5 | 27850 | 0.0169 | 17 |
| BIO19 | 24542 | 0.0149 | 18 |
| Bdinfection | 21622 | 0.0131 | 19 |
| BIO8 | 17653 | 0.0107 | 20 |
| BIO6 | 15639 | 0.0095 | 21 |

**Supplementary Table 6**. Results of Response screening (calculated in JMP 13.0) for all potential predictor variables considered in this study, for the response variable ‘Simpson’s evenness’ from the filtered data set of amphibian skin bacterial communities. N = 819 except for Bd infection intensity (N = 791). Variables sorted by decreasing LogWorth (-log10 transformation of P-values); higher values correspond to lower P-values. FDR refers to P-values adjusted to control the false discovery rate for multiple tests, calculated using the Benjamini-Hochberg technique. Bio 1 = Annual Mean Temperature. Bio 2 = Mean Diurnal Range. Bio 3 = Isothermality. Bio 4 = Temperature Seasonality. Bio 5 = Maximum Temperature of Warmest Month. Bio 6 = Minimum Temperature of Coldest Month. Bio 7 = Annual Temperature Range. Bio 8 = Mean Temperature of Wettest Quarter. Bio 9 = Mean Temperature of Driest Quarter. Bio 10 = Mean Temperature of Warmest Quarter. Bio 11 = Mean Temperature of Coldest Quarter. Bio 12 = Annual Precipitation. Bio 13 = Precipitation of Wettest Month. Bio 14 = Precipitation of Driest Month. Bio 15 = Precipitation Seasonality. Bio 16 = Precipitation of Wettest Quarter. Bio 17 = Precipitation of Driest Quarter. Bio 18 = Precipitation of Warmest Quarter. Bio 19 = Precipitation of Coldest Quarter. All variables were scaled before analyses.

| Y (response variable) | X (predictor variable) | P value | Log Worth (P value) | FDR P value | Log Worth (FDR P value) | Effect Size | F Ratio | R² |
| --- | --- | --- | --- | --- | --- | --- | --- | --- |
| **Simpson’s evenness** | **BIO6** | **0.000** | **7.696** | **0.000** | **6.971** | **0.253** | **32.114** | **0.038** |
| **Simpson’s evenness** | **Latitude** | **0.000** | **7.112** | **0.000** | **6.429** | **0.242** | **29.409** | **0.035** |
| **Simpson’s evenness** | **BIO9** | **0.000** | **7.031** | **0.000** | **6.372** | **0.241** | **29.034** | **0.034** |
| **Simpson’s evenness** | **BIO11** | **0.000** | **7.017** | **0.000** | **6.372** | **0.240** | **28.968** | **0.034** |
| **Simpson’s evenness** | **BIO7** | **0.000** | **6.755** | **0.000** | **6.127** | **0.236** | **27.759** | **0.033** |
| Simpson’s evenness | BIO2 | 0.000 | 6.338 | 0.000 | 5.728 | 0.228 | 25.846 | 0.031 |
| Simpson’s evenness | BIO1 | 0.000 | 6.123 | 0.000 | 5.574 | 0.223 | 24.861 | 0.030 |
| Simpson’s evenness | BIO18 | 0.000 | 6.113 | 0.000 | 5.574 | 0.223 | 24.813 | 0.029 |
| Simpson’s evenness | BIO19 | 0.000 | 6.108 | 0.000 | 5.574 | 0.223 | 24.788 | 0.029 |
| Simpson’s evenness | BIO4 | 0.000 | 5.038 | 0.000 | 4.593 | 0.201 | 19.931 | 0.024 |
| Simpson’s evenness | Elevation | 0.000 | 3.931 | 0.000 | 3.596 | 0.174 | 14.981 | 0.018 |
| Simpson’s evenness | BIO10 | 0.000 | 3.788 | 0.000 | 3.462 | 0.171 | 14.349 | 0.017 |
| Simpson’s evenness | BIO14 | 0.001 | 3.003 | 0.002 | 2.726 | 0.149 | 10.919 | 0.013 |
| Simpson’s evenness | BIO17 | 0.001 | 2.975 | 0.002 | 2.706 | 0.148 | 10.799 | 0.013 |
| Simpson’s evenness | BIO3 | 0.001 | 2.874 | 0.002 | 2.619 | 0.145 | 10.362 | 0.013 |
| Simpson’s evenness | BIO5 | 0.004 | 2.443 | 0.006 | 2.217 | 0.132 | 8.520 | 0.010 |
| Simpson’s evenness | BIO15 | 0.014 | 1.857 | 0.022 | 1.656 | 0.112 | 6.076 | 0.007 |
| Simpson’s evenness | BIO8 | 0.071 | 1.146 | 0.098 | 1.007 | 0.082 | 3.258 | 0.004 |
| Simpson’s evenness | BIO12 | 0.217 | 0.663 | 0.268 | 0.572 | 0.056 | 1.526 | 0.002 |
| Simpson’s evenness | BIO13 | 0.265 | 0.577 | 0.317 | 0.499 | 0.051 | 1.246 | 0.002 |
| Simpson’s evenness | BIO16 | 0.307 | 0.513 | 0.361 | 0.442 | 0.046 | 1.045 | 0.001 |
| Simpson’s evenness | Bdinfection | 0.522 | 0.282 | 0.565 | 0.248 | 0.030 | 0.410 | 0.001 |

**Supplementary Table 7**. Results of Predictor screening (calculated in JMP 13.0) for non-categorical predictor variables on the response variable Simpson’s evenness, for the full data set. N = 819 except for Bd infection intensity (N = 791). Bio 1 = Annual Mean Temperature. Bio 2 = Mean Diurnal Range. Bio 3 = Isothermality. Bio 4 = Temperature Seasonality. Bio 5 = Maximum Temperature of Warmest Month. Bio 6 = Minimum Temperature of Coldest Month. Bio 7 = Annual Temperature Range. Bio 8 = Mean Temperature of Wettest Quarter. Bio 9 = Mean Temperature of Driest Quarter. Bio 10 = Mean Temperature of Warmest Quarter. Bio 11 = Mean Temperature of Coldest Quarter. Bio 12 = Annual Precipitation. Bio 13 = Precipitation of Wettest Month. Bio 14 = Precipitation of Driest Month. Bio 15 = Precipitation Seasonality. Bio 16 = Precipitation of Wettest Quarter. Bio 17 = Precipitation of Driest Quarter. Bio 18 = Precipitation of Warmest Quarter. Bio 19 = Precipitation of Coldest Quarter. All variables were scaled before analyses.

| Predictor | Contribution | Portion | Rank |
| --- | --- | --- | --- |
| **BIO13** | **0.064** | **0.091** | **1** |
| **Latitude** | **0.062** | **0.089** | **2** |
| **BIO10** | **0.058** | **0.082** | **3** |
| **BIO4** | **0.042** | **0.060** | **4** |
| **BIO18** | **0.039** | **0.055** | **5** |
| BIO5 | 0.036 | 0.052 | 6 |
| BIO6 | 0.036 | 0.051 | 7 |
| BIO1 | 0.035 | 0.049 | 8 |
| Elevation | 0.035 | 0.049 | 9 |
| BIO3 | 0.033 | 0.048 | 10 |
| BIO9 | 0.031 | 0.045 | 11 |
| BIO11 | 0.030 | 0.043 | 12 |
| BIO19 | 0.028 | 0.040 | 13 |
| BIO7 | 0.027 | 0.039 | 14 |
| BIO2 | 0.022 | 0.032 | 15 |
| BIO14 | 0.017 | 0.025 | 16 |
| BIO12 | 0.017 | 0.025 | 17 |
| BIO16 | 0.017 | 0.024 | 18 |
| BIO17 | 0.016 | 0.023 | 19 |
| BIO8 | 0.013 | 0.018 | 20 |
| BIO15 | 0.011 | 0.016 | 21 |

**Supplementary Table 8**. Correlation coefficients between bioclimatic predictor variables, latitude, elevation, Bd infection intensity with #OTUs and Simpson’s evenness for all sampling sites included in this study. Bio 1 = Annual Mean Temperature. Bio 2 = Mean Diurnal Range. Bio 3 = Isothermality. Bio 4 = Temperature Seasonality. Bio 5 = Maximum Temperature of Warmest Month. Bio 6 = Minimum Temperature of Coldest Month. Bio 7 = Annual Temperature Range. Bio 8 = Mean Temperature of Wettest Quarter. Bio 9 = Mean Temperature of Driest Quarter. Bio 10 = Mean Temperature of Warmest Quarter. Bio 11 = Mean Temperature of Coldest Quarter. Bio 12 = Annual Precipitation. Bio 13 = Precipitation of Wettest Month. Bio 14 = Precipitation of Driest Month. Bio 15 = Precipitation Seasonality. Bio 16 = Precipitation of Wettest Quarter. Bio 17 = Precipitation of Driest Quarter. Bio 18 = Precipitation of Warmest Quarter. Bio 19 = Precipitation of Coldest Quarter. N = 819 except for Bd infection intensity (N = 791). P-values ware corrected for multiple-comparisons with Bonferroni correction. All variables were scaled before analyses.

|  | #OTUs | Simpson’s evenness | Latitude | Elevation | Bdinfection | BIO1 | BIO2 | BIO3 | BIO4 | BIO5 | BIO6 | BIO7 | BIO8 | BIO9 | BIO10 | BIO11 | BIO12 | BIO13 | BIO14 | BIO15 | BIO16 | BIO17 | BIO18 | BIO19 |
| --- | --- | --- | --- | --- | --- | --- | --- | --- | --- | --- | --- | --- | --- | --- | --- | --- | --- | --- | --- | --- | --- | --- | --- | --- |
| #OTUs |  | 0.000 | 0.046 | 0.013 | 0.459 | 0.078 | 0.060 | 0.236 | 0.674 | 0.863 | 0.421 | 0.157 | 0.000 | 0.022 | 0.042 | 0.002 | 0.000 | 0.214 | 0.002 | 0.375 | 0.742 | 0.002 | 0.101 | 0.000 |
| Simpson’s evenness | -0.320 |  | 0.000 | 0.000 | 0.051 | 0.000 | 0.000 | 0.000 | 0.000 | 0.019 | 0.000 | 0.000 | 0.003 | 0.000 | 0.000 | 0.000 | 0.001 | 0.844 | 0.000 | 0.002 | 0.678 | 0.001 | 0.000 | 0.005 |
| Latitude | -0.070 | 0.195 |  | 0.000 | 0.000 | 0.000 | 0.000 | 0.000 | 0.000 | 0.000 | 0.000 | 0.000 | 0.000 | 0.000 | 0.000 | 0.000 | 0.000 | 0.000 | 0.000 | 0.000 | 0.000 | 0.000 | 0.000 | 0.000 |
| Elevation | -0.087 | -0.149 | -0.338 |  | 0.000 | 0.000 | 0.000 | 0.000 | 0.000 | 0.000 | 0.000 | 0.000 | 0.000 | 0.000 | 0.000 | 0.000 | 0.027 | 0.008 | 0.041 | 0.000 | 0.000 | 0.004 | 0.000 | 0.000 |
| Bdinfection | 0.026 | -0.070 | -0.302 | 0.216 |  | 0.000 | 0.000 | 0.000 | 0.000 | 0.000 | 0.000 | 0.000 | 0.000 | 0.000 | 0.000 | 0.000 | 0.011 | 0.031 | 0.000 | 0.000 | 0.019 | 0.000 | 0.000 | 0.556 |
| BIO1 | -0.062 | 0.199 | 0.692 | -0.831 | -0.314 |  | 0.000 | 0.000 | 0.000 | 0.000 | 0.000 | 0.000 | 0.000 | 0.000 | 0.000 | 0.000 | 0.002 | 0.000 | 0.000 | 0.000 | 0.000 | 0.000 | 0.000 | 0.000 |
| BIO2 | -0.066 | -0.163 | -0.464 | 0.711 | 0.157 | -0.662 |  | 0.000 | 0.000 | 0.000 | 0.000 | 0.000 | 0.000 | 0.000 | 0.000 | 0.000 | 0.390 | 0.000 | 0.002 | 0.000 | 0.000 | 0.000 | 0.000 | 0.000 |
| BIO3 | 0.041 | 0.149 | 0.923 | -0.228 | -0.296 | 0.553 | -0.329 |  | 0.000 | 0.000 | 0.000 | 0.000 | 0.680 | 0.000 | 0.000 | 0.000 | 0.000 | 0.000 | 0.000 | 0.000 | 0.000 | 0.000 | 0.000 | 0.000 |
| BIO4 | -0.015 | -0.187 | -0.926 | 0.358 | 0.315 | -0.604 | 0.525 | -0.898 |  | 0.188 | 0.000 | 0.000 | 0.074 | 0.000 | 0.000 | 0.000 | 0.000 | 0.000 | 0.000 | 0.000 | 0.000 | 0.000 | 0.000 | 0.000 |
| BIO5 | 0.006 | 0.082 | 0.180 | -0.682 | -0.156 | 0.684 | -0.156 | 0.145 | -0.046 |  | 0.000 | 0.628 | 0.000 | 0.000 | 0.000 | 0.000 | 0.000 | 0.158 | 0.710 | 0.699 | 0.914 | 0.460 | 0.000 | 0.000 |
| BIO6 | -0.028 | 0.219 | 0.743 | -0.834 | -0.321 | 0.958 | -0.736 | 0.625 | -0.710 | 0.538 |  | 0.000 | 0.000 | 0.000 | 0.000 | 0.000 | 0.000 | 0.000 | 0.000 | 0.000 | 0.000 | 0.000 | 0.000 | 0.000 |
| BIO7 | -0.049 | -0.194 | -0.832 | 0.508 | 0.263 | -0.652 | 0.775 | -0.753 | 0.901 | -0.017 | -0.789 |  | 0.150 | 0.000 | 0.000 | 0.000 | 0.000 | 0.000 | 0.000 | 0.000 | 0.000 | 0.000 | 0.000 | 0.000 |
| BIO8 | -0.130 | 0.102 | 0.187 | -0.725 | -0.197 | 0.727 | -0.249 | 0.014 | -0.062 | 0.874 | 0.577 | -0.050 |  | 0.000 | 0.000 | 0.000 | 0.000 | 0.986 | 0.320 | 0.958 | 0.003 | 0.249 | 0.360 | 0.000 |
| BIO9 | -0.080 | 0.214 | 0.832 | -0.718 | -0.338 | 0.947 | -0.621 | 0.711 | -0.757 | 0.522 | 0.972 | -0.766 | 0.560 |  | 0.000 | 0.000 | 0.000 | 0.000 | 0.000 | 0.000 | 0.000 | 0.000 | 0.000 | 0.000 |
| BIO10 | -0.071 | 0.144 | 0.468 | -0.845 | -0.252 | 0.922 | -0.528 | 0.304 | -0.346 | 0.843 | 0.814 | -0.376 | 0.900 | 0.795 |  | 0.000 | 0.327 | 0.000 | 0.000 | 0.000 | 0.000 | 0.000 | 0.000 | 0.000 |
| BIO11 | -0.107 | 0.219 | 0.789 | -0.759 | -0.341 | 0.968 | -0.633 | 0.645 | -0.715 | 0.585 | 0.972 | -0.735 | 0.650 | 0.983 | 0.848 |  | 0.000 | 0.000 | 0.000 | 0.000 | 0.000 | 0.000 | 0.000 | 0.000 |
| BIO12 | 0.182 | -0.119 | -0.326 | -0.077 | 0.090 | -0.110 | -0.030 | -0.299 | 0.387 | 0.219 | -0.192 | 0.281 | 0.174 | -0.254 | 0.034 | -0.238 |  | 0.000 | 0.000 | 0.068 | 0.000 | 0.000 | 0.000 | 0.000 |
| BIO13 | 0.043 | 0.007 | 0.383 | -0.093 | -0.077 | 0.178 | -0.360 | 0.269 | -0.288 | -0.049 | 0.200 | -0.359 | 0.001 | 0.215 | 0.124 | 0.174 | 0.540 |  | 0.000 | 0.000 | 0.000 | 0.000 | 0.000 | 0.000 |
| BIO14 | 0.109 | -0.148 | -0.768 | 0.071 | 0.241 | -0.364 | 0.106 | -0.680 | 0.665 | 0.013 | -0.437 | 0.536 | -0.035 | -0.545 | -0.209 | -0.480 | 0.373 | -0.379 |  | 0.000 | 0.000 | 0.000 | 0.000 | 0.000 |
| BIO15 | -0.031 | 0.108 | 0.778 | -0.141 | -0.190 | 0.376 | -0.303 | 0.685 | -0.663 | -0.014 | 0.452 | -0.626 | -0.002 | 0.524 | 0.221 | 0.460 | -0.064 | 0.710 | -0.882 |  | 0.000 | 0.000 | 0.000 | 0.361 |
| BIO16 | 0.012 | 0.015 | 0.398 | -0.139 | -0.083 | 0.211 | -0.322 | 0.253 | -0.283 | -0.004 | 0.227 | -0.341 | 0.102 | 0.251 | 0.187 | 0.216 | 0.529 | 0.971 | -0.445 | 0.738 |  | 0.000 | 0.000 | 0.000 |
| BIO17 | 0.106 | -0.116 | -0.807 | 0.101 | 0.225 | -0.364 | 0.171 | -0.710 | 0.729 | 0.026 | -0.455 | 0.604 | -0.040 | -0.552 | -0.221 | -0.507 | 0.382 | -0.382 | 0.944 | -0.879 | -0.448 |  | 0.000 | 0.000 |
| BIO18 | -0.057 | -0.243 | -0.651 | 0.328 | 0.175 | -0.522 | 0.414 | -0.751 | 0.696 | -0.171 | -0.617 | 0.665 | 0.032 | -0.629 | -0.272 | -0.573 | 0.537 | 0.196 | 0.373 | -0.277 | 0.251 | 0.353 |  | 0.000 |
| BIO19 | 0.229 | 0.099 | 0.168 | -0.437 | -0.021 | 0.407 | -0.554 | 0.267 | -0.189 | 0.360 | 0.396 | -0.276 | 0.146 | 0.324 | 0.348 | 0.280 | 0.359 | 0.223 | 0.151 | 0.032 | 0.126 | 0.215 | -0.452 |  |

**Supplementary Table 9**. Comparison of generalized linear mixed models (GLMMs) for the response variable OTU richness, with predictor variables obtained via strategies 1-4 as described above according to the procedure of Kueneman et al. (2019) on full data set. location:sampling date:host species was used as nested random factor. For each fixed factor in the model, coefficients, standard error and the P value are shown. Bold P values indicate a significant effect of the fixed factor. Empty cells indicate the respective predictor was not included in the model. Bio 1 = Annual Mean Temperature. Bio 2 = Mean Diurnal Range. Bio 3 = Isothermality. Bio 4 = Temperature Seasonality. Bio 5 = Maximum Temperature of Warmest Month. Bio 6 = Minimum Temperature of Coldest Month. Bio 7 = Annual Temperature Range. Bio 8 = Mean Temperature of Wettest Quarter. Bio 9 = Mean Temperature of Driest Quarter. Bio 10 = Mean Temperature of Warmest Quarter. Bio 11 = Mean Temperature of Coldest Quarter. Bio 12 = Annual Precipitation. Bio 13 = Precipitation of Wettest Month. Bio 14 = Precipitation of Driest Month. Bio 15 = Precipitation Seasonality. Bio 16 = Precipitation of Wettest Quarter. Bio 17 = Precipitation of Driest Quarter. Bio 18 = Precipitation of Warmest Quarter. Bio 19 = Precipitation of Coldest Quarter. All variables were scaled before analyses. N = 819 except for Bd infection intensity (N = 791).

|  | **Model 1** | | | | **Model 2** | | | | **Model 3** | | | | **Model 4** | | | |
| --- | --- | --- | --- | --- | --- | --- | --- | --- | --- | --- | --- | --- | --- | --- | --- | --- |
| AIC | 8751.47 | | | | 8776.12 | | | | 8759.125 | | | | 8767.42 | | | |
| Intercept (SE) | 73.33 (8.96) | | | | 77.30 (12.07) | | | | 70.35 (8.14) | | | | 69.84 (7.89) | | | |
| Effect | Coef. | SE | P | VIF score | Coef. | SE | P | VIF score | Coef. | SE | P | VIF score | Coef. | SE | P | VIF score |
| Elevation | -82.77 | 20.88 | **<0.001** | 4.81 | -117.90 | 41.30 | **<0.001** | 3.37 | -101.60 | 33.30 | **0.006** | 17.55 | -111.38 | 23.99 | **<0.001** | 9.51 |
| Latitude | -0.25 | 35.77 | 0.994 | 2.17 | 47.38 | 35.93 | 0.203 | 2.15 | 45.47 | 44.17 | 0.316 | 33.69 | 42.07 | 42.82 | 0.336 | 33.63 |
| Bd infection intensity | 0.87 | 2.26 | 0.388 | 1.10 | 0.81 | 2.26 | 0.720 | 1.02 | 0.97 | 2.25 | 0.665 | 1.06 | 0.985 | 2.25 | 0.661 | 1.05 |
| BIO1 |  |  |  |  |  |  |  |  |  |  |  |  |  |  |  |  |
| BIO2 |  |  |  |  |  |  |  |  |  |  |  |  |  |  |  |  |
| BIO3 |  |  |  |  | 56.12 | 37.42 | 0.150 | 1.10 |  |  |  |  |  |  |  |  |
| BIO4 |  |  |  |  |  |  |  |  |  |  |  |  |  |  |  |  |
| BIO5 |  |  |  |  |  |  |  |  |  |  |  |  |  |  |  |  |
| BIO6 |  |  |  |  |  |  |  |  |  |  |  |  |  |  |  |  |
| BIO7 |  |  |  |  |  |  |  |  |  |  |  |  |  |  |  |  |
| BIO8 | -92.83 | 23.86 | **<0.001** | 3.15 |  |  |  |  | -90.97 | 32.01 | 0.009 | 19.67 | -78.51 | 15.09 | **<0.001** | 4.57 |
| BIO9 |  |  |  |  |  |  |  |  | -92.64 | 65.85 | 0.177 | 64.70 | -78.18 | 56.57 | 0.181 | 56.64 |
| BIO10 |  |  |  |  |  |  |  |  | 31.00 | 70.03 | 0.663 | 72.55 |  |  |  |  |
| BIO11 |  |  |  |  | -173.32 | 59.99 | 0.009 | 2.84 |  |  |  |  |  |  |  |  |
| BIO12 |  |  |  |  |  |  |  |  |  |  |  |  |  |  |  |  |
| BIO13 |  |  |  |  |  |  |  |  |  |  |  |  |  |  |  |  |
| BIO14 | -74.88 | 88.92 | 0.413 | 4.11 |  |  |  |  |  |  |  |  |  |  |  |  |
| BIO15 |  |  |  |  | -6.88 | 16.37 | 0.677 | 4.43 |  |  |  |  |  |  |  |  |
| BIO16 |  |  |  |  |  |  |  |  |  |  |  |  |  |  |  |  |
| BIO17 | 90.03 | 99.45 | 0.379 | 1.21 |  |  |  |  |  |  |  |  |  |  |  |  |
| BIO18 | 5.66 | 16.88 | 0.742 | 5.50 |  |  |  |  |  |  |  |  |  |  |  |  |
| BIO19 | -1.49 | 21.69 | 0.945 | 3.75 |  |  |  |  |  |  |  |  |  |  |  |  |

**Supplementary Table 10**. Comparison of generalized linear mixed models (GLMMs) for the response variable Simpson’s evenness, with predictor variables obtained via strategies 1-4 as described above according to the procedure of Kueneman et al. (2019) on full data set. location:sampling date:host species was used as nested random factor. For each fixed factor in the model, coefficients, standard error and the P value are shown. Bold P values indicate a significant effect of the fixed factor. Empty cells indicate the respective predictor was not included in the model. Bio 1 = Annual Mean Temperature. Bio 2 = Mean Diurnal Range. Bio 3 = Isothermality. Bio 4 = Temperature Seasonality. Bio 5 = Maximum Temperature of Warmest Month. Bio 6 = Minimum Temperature of Coldest Month. Bio 7 = Annual Temperature Range. Bio 8 = Mean Temperature of Wettest Quarter. Bio 9 = Mean Temperature of Driest Quarter. Bio 10 = Mean Temperature of Warmest Quarter. Bio 11 = Mean Temperature of Coldest Quarter. Bio 12 = Annual Precipitation. Bio 13 = Precipitation of Wettest Month. Bio 14 = Precipitation of Driest Month. Bio 15 = Precipitation Seasonality. Bio 16 = Precipitation of Wettest Quarter. Bio 17 = Precipitation of Driest Quarter. Bio 18 = Precipitation of Warmest Quarter. Bio 19 = Precipitation of Coldest Quarter. All variables were scaled before analyses. N = 819 except for Bd infection intensity (N = 791).

|  | **Model 1** | | | | **Model 2** | | | | **Model 3** | | | | **Model 4** | | | |
| --- | --- | --- | --- | --- | --- | --- | --- | --- | --- | --- | --- | --- | --- | --- | --- | --- |
| AIC | -1540.65 | | | | -1531.32 | | | | -1526.88 | | | | -1541.35 | | | |
| Intercept (SE) | 0.12 (0.01) | | | | 0.13 (0.01) | | | | 0.13 (0.01) | | | | 0.13 (0.01) | | | |
| Effect | Coef. | SE | P | VIF score | Coef. | SE | P | VIF score | Coef. | SE | P | VIF score | Coef. | SE | P | VIF score |
| Elevation | -0.01 | 0.03 | 0.631 | 20.70 | -0.02 | 0.02 | 0.449 | 12.21 | 0.00 | 0.01 | 0.841 | 6.87 | -0.00 | 0.01 | 0.957 | 2.95 |
| Latitude | 0.086 | 0.05 | 0.142 | 37.36 | -0.01 | 0.03 | 0.907 | 9.94 | -0.01 | 0.02 | 0.390 | 1.39 | -0.01 | 0.01 | 0.437 | 1.85 |
| Bd infection intensity | 0.00 | 0.00 | 0.750 | 1.02 | 0.00 | 0.00 | 0.761 | 1.03 | 0.00 | 0.00 | 0.737 | 1.04 | 0.00 | 0.0 | 0.723 | 1.01 |
| BIO1 |  |  |  |  |  |  |  |  |  |  |  |  |  |  |  |  |
| BIO2 |  |  |  |  |  |  |  |  |  |  |  |  |  |  |  |  |
| BIO3 |  |  |  |  |  |  |  |  |  |  |  |  |  |  |  |  |
| BIO4 |  |  |  |  | -0.01 | 0.02 | 0.850 | 8.48 |  |  |  |  |  |  |  |  |
| BIO5 |  |  |  |  |  |  |  |  | 0.02 | 0.02 | 0.266 | 12.34 |  |  |  |  |
| BIO6 | 0.16 | 0.14 | 0.257 | 331.31 |  |  |  | 7.29 |  |  |  |  |  |  |  |  |
| BIO7 | 0.03 | 0.04 | 0.452 | 30.78 |  |  |  | 1.25 |  |  |  |  |  |  |  |  |
| BIO8 |  |  |  |  |  |  |  |  | -0.02 | 0.02 | 0.322 | 11.12 |  |  |  |  |
| BIO9 | -0.21 | 0.13 | 0.147 | 184.84 |  |  |  |  |  |  |  |  |  |  |  |  |
| BIO10 |  |  |  |  | -0.02 | 0.03 | 0.543 | 15.05 |  |  |  | 1.56 |  |  |  |  |
| BIO11 | -0.00 | 0.08 | 0.970 | 106.84 |  |  |  |  |  |  |  |  |  |  |  |  |
| BIO12 |  |  |  |  |  |  |  |  |  |  |  |  |  |  |  |  |
| BIO13 |  |  |  |  | 0.01 | 0.01 | 0.470 | 1.25 |  |  |  |  |  |  |  |  |
| BIO14 |  |  |  |  |  |  |  |  |  |  |  |  |  |  |  |  |
| BIO15 |  |  |  |  |  |  |  |  | -0.03 | 0.02 | 0.073 | 9.71 | -0.04 | 0.01 | **0.033** | 7.29 |
| BIO16 |  |  |  |  |  |  |  |  |  |  |  |  |  |  |  |  |
| BIO17 |  |  |  |  | -0.02 | 0.02 | 0.2449 | 2.55 | -0.05 | 0.02 | **0.050** | 16.50 | -0.05 | 0.02 | **0.027** | 1.17 |
| BIO18 |  |  |  |  |  |  |  |  |  |  |  |  |  |  |  |  |
| BIO19 |  |  |  |  |  |  |  |  | 0.03 | 0.001 | 0.104 | 7.96 | 0.03 | 0.01 | **0.036** | 5.16 |

**Supplementary Table 11**. Results of Response screening (calculated in JMP 13.0) for all potential predictor variables considered in this study, for the response variable ‘number of observed OTUs’ from the filtered data set of amphibian skin bacterial communities after removing zero values for Bd infection intensity. Variables sorted by decreasing LogWorth (-log10 transformation of P-values); higher values correspond to lower P-values. FDR refers to P-values adjusted to control the false discovery rate for multiple tests, calculated using the Benjamini-Hochberg technique. Bio 1 = Annual Mean Temperature. Bio 2 = Mean Diurnal Range. Bio 3 = Isothermality. Bio 4 = Temperature Seasonality. Bio 5 = Maximum Temperature of Warmest Month. Bio 6 = Minimum Temperature of Coldest Month. Bio 7 = Annual Temperature Range. Bio 8 = Mean Temperature of Wettest Quarter. Bio 9 = Mean Temperature of Driest Quarter. Bio 10 = Mean Temperature of Warmest Quarter. Bio 11 = Mean Temperature of Coldest Quarter. Bio 12 = Annual Precipitation. Bio 13 = Precipitation of Wettest Month. Bio 14 = Precipitation of Driest Month. Bio 15 = Precipitation Seasonality. Bio 16 = Precipitation of Wettest Quarter. Bio 17 = Precipitation of Driest Quarter. Bio 18 = Precipitation of Warmest Quarter. Bio 19 = Precipitation of Coldest Quarter. All variables were scaled before analyses. N = 188.

| Y (response variable) | X (predictor variable) | P value | Log Worth (P value) | FDR P value | Log Worth (FDR P value) | Effect Size | F Ratio | R² |
| --- | --- | --- | --- | --- | --- | --- | --- | --- |
| **OTU** | **BIO14** | **0.000** | **14.480** | **0.000** | **13.455** | **0.545** | **64.621** | **0.076** |
| **OTU** | **BIO17** | **0.000** | **13.595** | **0.000** | **12.649** | **0.528** | **60.292** | **0.071** |
| **OTU** | **BIO15** | **0.000** | **12.881** | **0.000** | **12.032** | **0.513** | **56.816** | **0.067** |
| **OTU** | **BIO8** | **0.000** | **10.238** | **0.000** | **9.416** | **0.456** | **44.107** | **0.053** |
| **OTU** | **BIO19** | **0.000** | **4.810** | **0.000** | **4.341** | **0.303** | **18.910** | **0.023** |
| OTU | BIO11 | 0.000 | 4.157 | 0.000 | 3.744 | 0.279 | 15.989 | 0.020 |
| OTU | BIO1 | 0.000 | 3.982 | 0.000 | 3.590 | 0.272 | 15.214 | 0.019 |
| OTU | Latitude | 0.000 | 3.784 | 0.000 | 3.421 | 0.265 | 14.336 | 0.018 |
| OTU | BIO9 | 0.000 | 3.425 | 0.001 | 3.081 | 0.250 | 12.760 | 0.016 |
| OTU | BIO16 | 0.000 | 3.330 | 0.001 | 2.994 | 0.246 | 12.341 | 0.015 |
| OTU | BIO10 | 0.001 | 3.127 | 0.002 | 2.809 | 0.237 | 11.460 | 0.014 |
| OTU | BIO5 | 0.001 | 3.003 | 0.002 | 2.694 | 0.231 | 10.922 | 0.014 |
| OTU | BIO3 | 0.001 | 2.959 | 0.002 | 2.658 | 0.229 | 10.731 | 0.013 |
| OTU | BIO4 | 0.004 | 2.396 | 0.007 | 2.134 | 0.202 | 8.324 | 0.010 |
| OTU | BIO13 | 0.005 | 2.285 | 0.009 | 2.031 | 0.197 | 7.857 | 0.010 |
| OTU | BIO6 | 0.006 | 2.208 | 0.011 | 1.961 | 0.193 | 7.532 | 0.009 |
| OTU | BIO12 | 0.017 | 1.763 | 0.028 | 1.557 | 0.168 | 5.694 | 0.007 |
| OTU | BIO18 | 0.030 | 1.529 | 0.046 | 1.336 | 0.153 | 4.751 | 0.006 |
| OTU | Bdinfection | 0.061 | 1.218 | 0.088 | 1.056 | 0.132 | 3.533 | 0.004 |
| OTU | Elevation | 0.133 | 0.877 | 0.176 | 0.755 | 0.106 | 2.266 | 0.003 |
| OTU | BIO7 | 0.187 | 0.728 | 0.236 | 0.627 | 0.093 | 1.745 | 0.002 |
| OTU | BIO2 | 0.537 | 0.270 | 0.581 | 0.236 | 0.044 | 0.382 | 0.000 |

**Supplementary Table 12**. Results of Predictor screening (calculated in JMP 13.0) for non-categorical predictor variables on the response variable #OTU richness, for the full data set after removing zero values for Bd infection intensity. Bio 1 = Annual Mean Temperature. Bio 2 = Mean Diurnal Range. Bio 3 = Isothermality. Bio 4 = Temperature Seasonality. Bio 5 = Maximum Temperature of Warmest Month. Bio 6 = Minimum Temperature of Coldest Month. Bio 7 = Annual Temperature Range. Bio 8 = Mean Temperature of Wettest Quarter. Bio 9 = Mean Temperature of Driest Quarter. Bio 10 = Mean Temperature of Warmest Quarter. Bio 11 = Mean Temperature of Coldest Quarter. Bio 12 = Annual Precipitation. Bio 13 = Precipitation of Wettest Month. Bio 14 = Precipitation of Driest Month. Bio 15 = Precipitation Seasonality. Bio 16 = Precipitation of Wettest Quarter. Bio 17 = Precipitation of Driest Quarter. Bio 18 = Precipitation of Warmest Quarter. Bio 19 = Precipitation of Coldest Quarter. All variables were scaled before analyses. N = 188.

| Predictor | Contribution | Portion | Rank |
| --- | --- | --- | --- |
| **BIO13** | **0.063288** | **0.0987** | **1** |
| **BIO10** | **0.05577** | **0.0869** | **2** |
| **BIO18** | **0.05352** | **0.0834** | **3** |
| **BIO5** | **0.053502** | **0.0834** | **4** |
| **BIO4** | **0.041955** | **0.0654** | **5** |
| BIO1 | 0.040063 | 0.0625 | 6 |
| Latitude | 0.039555 | 0.0617 | 7 |
| BIO3 | 0.034881 | 0.0544 | 8 |
| BIO11 | 0.033931 | 0.0529 | 9 |
| BIO19 | 0.025918 | 0.0404 | 10 |
| BIO6 | 0.024386 | 0.038 | 11 |
| BIO12 | 0.023777 | 0.0371 | 12 |
| Elevation | 0.02296 | 0.0358 | 13 |
| BIO7 | 0.019883 | 0.031 | 14 |
| BIO2 | 0.019572 | 0.0305 | 15 |
| BIO9 | 0.016963 | 0.0264 | 16 |
| BIO14 | 0.015386 | 0.024 | 17 |
| BIO17 | 0.012795 | 0.0199 | 18 |
| BIO16 | 0.012311 | 0.0192 | 19 |
| Bdinfection | 0.008756 | 0.0136 | 20 |
| BIO15 | 0.008401 | 0.0131 | 21 |

**Supplementary Table 13**. Correlation coefficients between bioclimatic predictor variables, latitude, elevation, Bd infection intensity with #OTUs and Simpson’s evenness for all sampling sites included in this study after removing zero values for Bd infection intensity. Bio 1 = Annual Mean Temperature. Bio 2 = Mean Diurnal Range. Bio 3 = Isothermality. Bio 4 = Temperature Seasonality. Bio 5 = Maximum Temperature of Warmest Month. Bio 6 = Minimum Temperature of Coldest Month. Bio 7 = Annual Temperature Range. Bio 8 = Mean Temperature of Wettest Quarter. Bio 9 = Mean Temperature of Driest Quarter. Bio 10 = Mean Temperature of Warmest Quarter. Bio 11 = Mean Temperature of Coldest Quarter. Bio 12 = Annual Precipitation. Bio 13 = Precipitation of Wettest Month. Bio 14 = Precipitation of Driest Month. Bio 15 = Precipitation Seasonality. Bio 16 = Precipitation of Wettest Quarter. Bio 17 = Precipitation of Driest Quarter. Bio 18 = Precipitation of Warmest Quarter. Bio 19 = Precipitation of Coldest Quarter. P-values ware corrected for multiple-comparisons with Bonferroni correction. All variables were scaled before analyses. N = 188.

|  | OTU | Simpson’s evenness | Latitude | Elevation | Bdinfection | BIO1 | BIO2 | BIO3 | BIO4 | BIO5 | BIO6 | BIO7 | BIO8 | BIO9 | BIO10 | BIO11 | BIO12 | BIO13 | BIO14 | BIO15 | BIO16 | BIO17 | BIO18 | BIO19 |
| --- | --- | --- | --- | --- | --- | --- | --- | --- | --- | --- | --- | --- | --- | --- | --- | --- | --- | --- | --- | --- | --- | --- | --- | --- |
| OTU |  | 0.000 | 0.429 | 0.001 | 0.459 | 0.299 | 0.001 | 0.156 | 0.136 | 0.418 | 0.775 | 0.004 | 0.000 | 0.211 | 0.130 | 0.042 | 0.000 | 0.010 | 0.079 | 0.514 | 0.071 | 0.099 | 0.114 | 0.000 |
| Simpson’s evenness | -0.306 |  | 0.000 | 0.000 | 0.051 | 0.000 | 0.000 | 0.000 | 0.000 | 0.005 | 0.000 | 0.000 | 0.004 | 0.000 | 0.000 | 0.000 | 0.002 | 0.688 | 0.000 | 0.016 | 0.763 | 0.007 | 0.000 | 0.002 |
| Latitude | -0.028 | 0.185 |  | 0.000 | 0.000 | 0.000 | 0.000 | 0.000 | 0.000 | 0.000 | 0.000 | 0.000 | 0.000 | 0.000 | 0.000 | 0.000 | 0.000 | 0.000 | 0.000 | 0.000 | 0.000 | 0.000 | 0.000 | 0.000 |
| Elevation | -0.114 | -0.145 | -0.335 |  | 0.000 | 0.000 | 0.000 | 0.000 | 0.000 | 0.000 | 0.000 | 0.000 | 0.000 | 0.000 | 0.000 | 0.000 | 0.008 | 0.019 | 0.131 | 0.000 | 0.000 | 0.015 | 0.000 | 0.000 |
| Bdinfection | 0.026 | -0.070 | -0.302 | 0.216 |  | 0.000 | 0.000 | 0.000 | 0.000 | 0.000 | 0.000 | 0.000 | 0.000 | 0.000 | 0.000 | 0.000 | 0.011 | 0.031 | 0.000 | 0.000 | 0.019 | 0.000 | 0.000 | 0.556 |
| BIO1 | -0.037 | 0.195 | 0.699 | -0.821 | -0.314 |  | 0.000 | 0.000 | 0.000 | 0.000 | 0.000 | 0.000 | 0.000 | 0.000 | 0.000 | 0.000 | 0.009 | 0.000 | 0.000 | 0.000 | 0.000 | 0.000 | 0.000 | 0.000 |
| BIO2 | -0.122 | -0.145 | -0.451 | 0.706 | 0.157 | -0.653 |  | 0.000 | 0.000 | 0.000 | 0.000 | 0.000 | 0.000 | 0.000 | 0.000 | 0.000 | 0.028 | 0.000 | 0.135 | 0.000 | 0.000 | 0.001 | 0.000 | 0.000 |
| BIO3 | 0.050 | 0.151 | 0.936 | -0.244 | -0.296 | 0.579 | -0.343 |  | 0.000 | 0.000 | 0.000 | 0.000 | 0.340 | 0.000 | 0.000 | 0.000 | 0.000 | 0.000 | 0.000 | 0.000 | 0.000 | 0.000 | 0.000 | 0.000 |
| BIO4 | -0.053 | -0.179 | -0.931 | 0.355 | 0.315 | -0.612 | 0.513 | -0.909 |  | 0.053 | 0.000 | 0.000 | 0.056 | 0.000 | 0.000 | 0.000 | 0.000 | 0.000 | 0.000 | 0.000 | 0.000 | 0.000 | 0.000 | 0.000 |
| BIO5 | -0.029 | 0.101 | 0.206 | -0.700 | -0.156 | 0.711 | -0.189 | 0.158 | -0.069 |  | 0.000 | 0.198 | 0.000 | 0.000 | 0.000 | 0.000 | 0.000 | 0.335 | 0.554 | 0.617 | 0.699 | 0.878 | 0.000 | 0.000 |
| BIO6 | 0.010 | 0.211 | 0.746 | -0.830 | -0.321 | 0.957 | -0.730 | 0.648 | -0.712 | 0.570 |  | 0.000 | 0.000 | 0.000 | 0.000 | 0.000 | 0.000 | 0.000 | 0.000 | 0.000 | 0.000 | 0.000 | 0.000 | 0.000 |
| BIO7 | -0.101 | -0.181 | -0.833 | 0.506 | 0.263 | -0.655 | 0.765 | -0.769 | 0.899 | -0.046 | -0.791 |  | 0.159 | 0.000 | 0.000 | 0.000 | 0.000 | 0.000 | 0.000 | 0.000 | 0.000 | 0.000 | 0.000 | 0.000 |
| BIO8 | -0.127 | 0.103 | 0.189 | -0.721 | -0.197 | 0.727 | -0.240 | 0.034 | -0.068 | 0.891 | 0.578 | -0.050 |  | 0.000 | 0.000 | 0.000 | 0.000 | 0.864 | 0.268 | 0.986 | 0.007 | 0.199 | 0.505 | 0.000 |
| BIO9 | -0.044 | 0.205 | 0.835 | -0.710 | -0.338 | 0.949 | -0.611 | 0.732 | -0.759 | 0.556 | 0.971 | -0.766 | 0.563 |  | 0.000 | 0.000 | 0.000 | 0.000 | 0.000 | 0.000 | 0.000 | 0.000 | 0.000 | 0.000 |
| BIO10 | -0.054 | 0.142 | 0.465 | -0.838 | -0.252 | 0.919 | -0.515 | 0.320 | -0.345 | 0.869 | 0.810 | -0.371 | 0.903 | 0.793 |  | 0.000 | 0.219 | 0.002 | 0.000 | 0.000 | 0.000 | 0.000 | 0.000 | 0.000 |
| BIO11 | -0.072 | 0.210 | 0.790 | -0.751 | -0.341 | 0.969 | -0.620 | 0.667 | -0.718 | 0.620 | 0.970 | -0.734 | 0.654 | 0.982 | 0.847 |  | 0.000 | 0.000 | 0.000 | 0.000 | 0.000 | 0.000 | 0.000 | 0.000 |
| BIO12 | 0.157 | -0.108 | -0.296 | -0.095 | 0.090 | -0.093 | -0.078 | -0.283 | 0.350 | 0.187 | -0.162 | 0.242 | 0.170 | -0.225 | 0.044 | -0.212 |  | 0.000 | 0.000 | 0.871 | 0.000 | 0.000 | 0.000 | 0.000 |
| BIO13 | 0.092 | -0.014 | 0.369 | -0.083 | -0.077 | 0.170 | -0.337 | 0.278 | -0.280 | -0.034 | 0.187 | -0.341 | -0.006 | 0.202 | 0.112 | 0.158 | 0.599 |  | 0.000 | 0.000 | 0.000 | 0.000 | 0.000 | 0.000 |
| BIO14 | 0.063 | -0.130 | -0.754 | 0.054 | 0.241 | -0.357 | 0.053 | -0.688 | 0.645 | -0.021 | -0.416 | 0.506 | -0.039 | -0.528 | -0.202 | -0.461 | 0.332 | -0.356 |  | 0.000 | 0.000 | 0.000 | 0.000 | 0.000 |
| BIO15 | 0.023 | 0.086 | 0.767 | -0.128 | -0.190 | 0.372 | -0.262 | 0.696 | -0.648 | 0.018 | 0.436 | -0.601 | 0.001 | 0.511 | 0.214 | 0.444 | -0.006 | 0.702 | -0.875 |  | 0.000 | 0.000 | 0.000 | 0.133 |
| BIO16 | 0.064 | -0.011 | 0.377 | -0.128 | -0.083 | 0.199 | -0.293 | 0.259 | -0.267 | 0.014 | 0.208 | -0.315 | 0.097 | 0.233 | 0.174 | 0.194 | 0.594 | 0.969 | -0.421 | 0.727 |  | 0.000 | 0.000 | 0.000 |
| BIO17 | 0.059 | -0.095 | -0.796 | 0.086 | 0.225 | -0.358 | 0.122 | -0.717 | 0.713 | -0.005 | -0.436 | 0.577 | -0.046 | -0.536 | -0.215 | -0.491 | 0.343 | -0.360 | 0.940 | -0.871 | -0.425 |  | 0.000 | 0.000 |
| BIO18 | -0.056 | -0.248 | -0.665 | 0.335 | 0.175 | -0.537 | 0.436 | -0.748 | 0.705 | -0.174 | -0.633 | 0.685 | 0.024 | -0.644 | -0.279 | -0.587 | 0.542 | 0.195 | 0.377 | -0.280 | 0.256 | 0.354 |  | 0.000 |
| BIO19 | 0.212 | 0.112 | 0.196 | -0.448 | -0.021 | 0.423 | -0.603 | 0.276 | -0.219 | 0.339 | 0.426 | -0.314 | 0.144 | 0.353 | 0.351 | 0.304 | 0.346 | 0.242 | 0.135 | 0.054 | 0.147 | 0.204 | -0.460 |  |

**Supplementary Table 14**. Comparison of generalized linear mixed models (GLMMs) for the response variable OTU richness after removing zero values for Bd infection intensity, with predictor variables obtained via strategies 1-4 as described above according to the procedure of Kueneman et al. (2019). Location:sampling date:host species was used as nested random factor. For each fixed factor in the model, coefficients, standard error and the P value are shown. Bold P values indicate a significant effect of the fixed factor. Empty cells indicate the respective predictor was not included in the model. Bio 1 = Annual Mean Temperature. Bio 2 = Mean Diurnal Range. Bio 3 = Isothermality. Bio 4 = Temperature Seasonality. Bio 5 = Maximum Temperature of Warmest Month. Bio 6 = Minimum Temperature of Coldest Month. Bio 7 = Annual Temperature Range. Bio 8 = Mean Temperature of Wettest Quarter. Bio 9 = Mean Temperature of Driest Quarter. Bio 10 = Mean Temperature of Warmest Quarter. Bio 11 = Mean Temperature of Coldest Quarter. Bio 12 = Annual Precipitation. Bio 13 = Precipitation of Wettest Month. Bio 14 = Precipitation of Driest Month. Bio 15 = Precipitation Seasonality. Bio 16 = Precipitation of Wettest Quarter. Bio 17 = Precipitation of Driest Quarter. Bio 18 = Precipitation of Warmest Quarter. Bio 19 = Precipitation of Coldest Quarter. All variables were scaled before analyses. N = 188.

|  | **Model 1** | | | | **Model 2** | | | | **Model 3** | | | | **Model 4** | | | |
| --- | --- | --- | --- | --- | --- | --- | --- | --- | --- | --- | --- | --- | --- | --- | --- | --- |
| AIC | 2299.24 | | | | 2326.78 | | | | 2313.09 | | | | 2319.18 | | | |
| Intercept (SE) | 70.96(13.62) | | | | 70.92(20.97) | | | | 64.96(16.19) | | | | 66.52(15.65) | | | |
| Effect | Coef. | SE | P | VIF score | Coef. | SE | P | VIF score | Coef. | SE | P | VIF score | Coef. | SE | P | VIF score |
| Elevation | -112.94 | 23.69 | **0.002** | 6.08 | -135.72 | 67.02 | 0.063 | 8.95 | -164.91 | 51.29 | **0.010** | 10.59 | -181.95 | 42.94 | **0.001** | 10.42 |
| Latitude | 21.17 | 47.26 | 0.667 | 6.31 | 39.61 | 68.84 | 0.575 | 6.60 | -20.29 | 54.83 | 0.718 | 7.95 | -17.17 | 53.09 | 0.752 | 7.37 |
| Bd infection intensity | -0.02 | 2.46 | 0.927 | 1.06 | -0.42 | 2.48 | 0.863 | 1.07 | -0.28 | 2.47 | 0.907 | 1.08 | -0.36 | 2.47 | 0.881 | 1.08 |
| BIO1 |  |  |  |  |  |  |  |  |  |  |  |  |  |  |  |  |
| BIO2 |  |  |  |  |  |  |  |  | 134.26 | 55.24 | **0.039** | 6.24 | 149.52 | 48.79 | **0.013** | 5.75 |
| BIO3 |  |  |  |  | 70.81 | 60.49 | 0.264 | 3.34 |  |  |  |  |  |  |  |  |
| BIO4 |  |  |  |  |  |  |  |  |  |  |  |  |  |  |  |  |
| BIO5 |  |  |  |  |  |  |  |  |  |  |  |  |  |  |  |  |
| BIO6 |  |  |  |  |  |  |  |  |  |  |  |  |  |  |  |  |
| BIO7 |  |  |  |  |  |  |  |  | -261.74 | 90.79 | **0.020** | 9.54 | -287.21 | 79.98 | **0.006** | 8.94 |
| BIO8 | -137.39 | 29.43 | **0.003** | 9.59 |  |  |  |  |  |  |  |  |  |  |  |  |
| BIO9 |  |  |  |  |  |  |  |  |  |  |  |  |  |  |  |  |
| BIO10 |  |  |  |  |  |  |  |  |  |  |  |  |  |  |  |  |
| BIO11 |  |  |  |  | -207.83 | 98.43 | 0.054 | 10.79 | -252.77 | 79.37 | **0.011** | 12.06 | -277.25 | 67.89 | **0.002** | 12.01 |
| BIO12 |  |  |  |  |  |  |  |  |  |  |  |  |  |  |  |  |
| BIO13 |  |  |  |  |  |  |  |  | -12.23 | 18.43 | 0.521 | 1.28 |  |  |  |  |
| BIO14 | -176.43 | 108.42 | 0.161 | 3.62 |  |  |  |  |  |  |  |  |  |  |  |  |
| BIO15 |  |  |  |  | -10.48 | 28.1 | 0.715 | 2.29 |  |  |  |  |  |  |  |  |
| BIO16 |  |  |  |  |  |  |  |  |  |  |  |  |  |  |  |  |
| BIO17 | 216.74 | 121.89 | 0.127 | 2.98 |  |  |  |  |  |  |  |  |  |  |  |  |
| BIO18 | 16.60 | 21.91 | 0.479 | 4.62 |  |  |  |  |  |  |  |  |  |  |  |  |
| BIO19 | -21.09 | 39.87 | 0.609 | 7.38 |  |  |  |  |  |  |  |  |  |  |  |  |

**Supplementary Table 15**. Results of Response screening (calculated in JMP 13.0) for all potential predictor variables considered in this study, for the response variable ‘Simpson’s evenness’ from the filtered data set of amphibian skin bacterial communities after removing zero values for Bd infection intensity. Variables sorted by decreasing LogWorth (-log10 transformation of P-values); higher values correspond to lower P-values. FDR refers to P-values adjusted to control the false discovery rate for multiple tests, calculated using the Benjamini-Hochberg technique. Bio 1 = Annual Mean Temperature. Bio 2 = Mean Diurnal Range. Bio 3 = Isothermality. Bio 4 = Temperature Seasonality. Bio 5 = Maximum Temperature of Warmest Month. Bio 6 = Minimum Temperature of Coldest Month. Bio 7 = Annual Temperature Range. Bio 8 = Mean Temperature of Wettest Quarter. Bio 9 = Mean Temperature of Driest Quarter. Bio 10 = Mean Temperature of Warmest Quarter. Bio 11 = Mean Temperature of Coldest Quarter. Bio 12 = Annual Precipitation. Bio 13 = Precipitation of Wettest Month. Bio 14 = Precipitation of Driest Month. Bio 15 = Precipitation Seasonality. Bio 16 = Precipitation of Wettest Quarter. Bio 17 = Precipitation of Driest Quarter. Bio 18 = Precipitation of Warmest Quarter. Bio 19 = Precipitation of Coldest Quarter. All variables were scaled before analyses. N = 188.

| Y (response variable) | X (predictor variable) | P value | Log Worth (P value) | FDR P value | Log Worth (FDR P value) | Effect Size | F Ratio | R² |
| --- | --- | --- | --- | --- | --- | --- | --- | --- |
| **Simpson’s evenness** | **BIO6** | **0.000** | **6.963** | **0.000** | **6.259** | **0.247** | **28.736** | **0.035** |
| **Simpson’s evenness** | **BIO19** | **0.000** | **6.460** | **0.000** | **5.777** | **0.237** | **26.420** | **0.032** |
| **Simpson’s evenness** | **BIO9** | **0.000** | **6.435** | **0.000** | **5.771** | **0.237** | **26.303** | **0.032** |
| **Simpson’s evenness** | **Latitude** | **0.000** | **6.402** | **0.000** | **5.758** | **0.236** | **26.153** | **0.032** |
| **Simpson’s evenness** | **BIO11** | **0.000** | **6.386** | **0.000** | **5.758** | **0.236** | **26.077** | **0.032** |
| Simpson’s evenness | BIO18 | 0.000 | 6.128 | 0.000 | 5.550 | 0.231 | 24.898 | 0.031 |
| Simpson’s evenness | BIO7 | 0.000 | 5.808 | 0.000 | 5.260 | 0.224 | 23.434 | 0.029 |
| Simpson’s evenness | BIO1 | 0.000 | 5.674 | 0.000 | 5.140 | 0.221 | 22.822 | 0.028 |
| Simpson’s evenness | BIO2 | 0.000 | 5.301 | 0.000 | 4.807 | 0.213 | 21.127 | 0.026 |
| Simpson’s evenness | BIO4 | 0.000 | 4.377 | 0.000 | 3.943 | 0.191 | 16.972 | 0.021 |
| Simpson’s evenness | Elevation | 0.000 | 3.849 | 0.000 | 3.477 | 0.178 | 14.622 | 0.018 |
| Simpson’s evenness | BIO10 | 0.000 | 3.638 | 0.001 | 3.285 | 0.172 | 13.694 | 0.017 |
| Simpson’s evenness | BIO3 | 0.002 | 2.803 | 0.003 | 2.510 | 0.148 | 10.060 | 0.013 |
| Simpson’s evenness | BIO5 | 0.002 | 2.722 | 0.004 | 2.445 | 0.145 | 9.714 | 0.012 |
| Simpson’s evenness | BIO14 | 0.007 | 2.154 | 0.012 | 1.921 | 0.126 | 7.306 | 0.009 |
| Simpson’s evenness | BIO17 | 0.008 | 2.110 | 0.013 | 1.891 | 0.125 | 7.125 | 0.009 |
| Simpson’s evenness | BIO15 | 0.060 | 1.220 | 0.088 | 1.056 | 0.088 | 3.540 | 0.004 |
| Simpson’s evenness | BIO8 | 0.071 | 1.152 | 0.100 | 1.001 | 0.085 | 3.280 | 0.004 |
| Simpson’s evenness | BIO12 | 0.314 | 0.503 | 0.374 | 0.427 | 0.047 | 1.014 | 0.001 |
| Simpson’s evenness | BIO13 | 0.439 | 0.358 | 0.505 | 0.296 | 0.036 | 0.601 | 0.001 |
| Simpson’s evenness | Bdinfection | 0.522 | 0.282 | 0.571 | 0.244 | 0.030 | 0.410 | 0.001 |
| Simpson’s evenness | BIO16 | 0.516 | 0.287 | 0.571 | 0.244 | 0.030 | 0.422 | 0.001 |

**Supplementary Table 16**. Results of Predictor screening (calculated in JMP 13.0) for non-categorical predictor variables on the response variable Simpson’s evenness, for the full data set after removing zero values for Bd infection intensity. Bio 1 = Annual Mean Temperature. Bio 2 = Mean Diurnal Range. Bio 3 = Isothermality. Bio 4 = Temperature Seasonality. Bio 5 = Maximum Temperature of Warmest Month. Bio 6 = Minimum Temperature of Coldest Month. Bio 7 = Annual Temperature Range. Bio 8 = Mean Temperature of Wettest Quarter. Bio 9 = Mean Temperature of Driest Quarter. Bio 10 = Mean Temperature of Warmest Quarter. Bio 11 = Mean Temperature of Coldest Quarter. Bio 12 = Annual Precipitation. Bio 13 = Precipitation of Wettest Month. Bio 14 = Precipitation of Driest Month. Bio 15 = Precipitation Seasonality. Bio 16 = Precipitation of Wettest Quarter. Bio 17 = Precipitation of Driest Quarter. Bio 18 = Precipitation of Warmest Quarter. Bio 19 = Precipitation of Coldest Quarter. All variables were scaled before analyses. N = 188.

| Predictor | Contribution | Portion | Rank |
| --- | --- | --- | --- |
| **BIO13** | **0.063288** | **0.0987** | **1** |
| **BIO10** | **0.05577** | **0.0869** | **2** |
| **BIO18** | **0.05352** | **0.0834** | **3** |
| **BIO5** | **0.053502** | **0.0834** | **4** |
| **BIO4** | **0.041955** | **0.0654** | **5** |
| BIO1 | 0.040063 | 0.0625 | 6 |
| Latitude | 0.039555 | 0.0617 | 7 |
| BIO3 | 0.034881 | 0.0544 | 8 |
| BIO11 | 0.033931 | 0.0529 | 9 |
| BIO19 | 0.025918 | 0.0404 | 10 |
| BIO6 | 0.024386 | 0.038 | 11 |
| BIO12 | 0.023777 | 0.0371 | 12 |
| Elevation | 0.02296 | 0.0358 | 13 |
| BIO7 | 0.019883 | 0.031 | 14 |
| BIO2 | 0.019572 | 0.0305 | 15 |
| BIO9 | 0.016963 | 0.0264 | 16 |
| BIO14 | 0.015386 | 0.024 | 17 |
| BIO17 | 0.012795 | 0.0199 | 18 |
| BIO16 | 0.012311 | 0.0192 | 19 |
| Bdinfection | 0.008756 | 0.0136 | 20 |
| BIO15 | 0.008401 | 0.0131 | 21 |

**Supplementary Table 17**. Comparison of generalized linear mixed models (GLMMs) for the response variable Simpson’s eveness after removing zero values for Bd infection intensity, with predictor variables obtained via strategies 1-4 as described above according to the procedure of Kueneman et al. (2019). location:sampling date:host species was used as nested random factor. For each fixed factor in the model, coefficients, standard error and the P value are shown. Bold P values indicate a significant effect of the fixed factor. Empty cells indicate the respective predictor was not included in the model. Bio 1 = Annual Mean Temperature. Bio 2 = Mean Diurnal Range. Bio 3 = Isothermality. Bio 4 = Temperature Seasonality. Bio 5 = Maximum Temperature of Warmest Month. Bio 6 = Minimum Temperature of Coldest Month. Bio 7 = Annual Temperature Range. Bio 8 = Mean Temperature of Wettest Quarter. Bio 9 = Mean Temperature of Driest Quarter. Bio 10 = Mean Temperature of Warmest Quarter. Bio 11 = Mean Temperature of Coldest Quarter. Bio 12 = Annual Precipitation. Bio 13 = Precipitation of Wettest Month. Bio 14 = Precipitation of Driest Month. Bio 15 = Precipitation Seasonality. Bio 16 = Precipitation of Wettest Quarter. Bio 17 = Precipitation of Driest Quarter. Bio 18 = Precipitation of Warmest Quarter. Bio 19 = Precipitation of Coldest Quarter. All variables were scaled before analyses. N = 188.

| # | **Model 1** | | | | **Model 2** | | | | **Model 3** | | | | **Model 4** | | | |
| --- | --- | --- | --- | --- | --- | --- | --- | --- | --- | --- | --- | --- | --- | --- | --- | --- |
| AIC | -313.05 | | | | -302.12 | | | | -302.79 | | | | -316.33 | | | |
| Intercept (SE) | 0.12(0.01) | | | | 0.12(0.01) | | | | 0.12(0.01) | | | | 0.12(0.01) | | | |
| Effect | Coef. | SE | P | VIF score | Coef. | SE | P | VIF score | Coef. | SE | P | VIF score | Coef. | SE | P | VIF score |
| Elevation | 0.01 | 0.06 | 0.907 | 53.26 | 0.02 | 0.05 | 0.648 | 13.04 | 0.01 | 0.02 | 0.593 | 3.14 | -0.01 | 0.00 | 0.381 | 2.01 |
| Latitude | -0.02 | 0.11 | 0.802 | 41.34 | -0.03 | 0.09 | 0.737 | 6.19 | 0.01 | 0.02 | 0.756 | 4.42 | -0.01 | 0.00 | 0.509 | 3.14 |
| Bd infection intensity | 0.00 | 0.00 | 0.696 | 1.05 | 0.00 | 0.00 | 0.675 | 1.06 | 0.00 | 0.00 | 0.714 | 1.10 | 0.00 | 0.00 | 0.856 | 1.03 |
| BIO1 |  |  |  |  |  |  |  |  |  |  |  |  |  |  |  |  |
| BIO2 |  |  |  |  |  |  |  |  |  |  |  |  |  |  |  |  |
| BIO3 |  |  |  |  |  |  |  |  |  |  |  |  |  |  |  |  |
| BIO4 |  |  |  |  | -0.01 | 0.03 | 0.642 | 6.33 |  |  |  |  |  |  |  |  |
| BIO5 |  |  |  |  | -0.07 | 0.05 | 0.212 | 7.49 | 0.03 | 0.04 | 0.492 | 22.01 |  |  |  |  |
| BIO6 | 0.16 | 0.10 | 0.159 | 42.53 |  |  |  |  |  |  |  |  |  |  |  |  |
| BIO7 |  |  |  |  |  |  |  |  |  |  |  |  |  |  |  |  |
| BIO8 |  |  |  |  |  |  |  |  | -0.01 | 0.03 | 0.611 | 16.34 | -0.01 | 0.01 | 0.325 | 2.66 |
| BIO9 | 0.02 | 0.20 | 0.889 | 117.24 |  |  |  |  |  |  |  |  |  |  |  |  |
| BIO10 |  |  |  |  | 0.11 | 0.11 | 0.368 | 12.05 |  |  |  |  |  |  |  |  |
| BIO11 | -0.14 | 0.15 | 0.38 | 70.46 |  |  |  |  |  |  |  |  |  |  |  |  |
| BIO12 |  |  |  |  |  |  |  |  | 0.03 | 0.02 | 0.213 | 2.79 |  |  |  |  |
| BIO13 |  |  |  |  | -0.03 | 0.05 | 0.449 | 10.34 |  |  |  |  |  |  |  |  |
| BIO14 |  |  |  |  |  |  |  |  |  |  |  |  |  |  |  |  |
| BIO15 |  |  |  |  |  |  |  |  | -0.14 | 0.05 | **0.025** | 12.44 | -0.01 | 0.01 | 0.391 | 2.56 |
| BIO16 |  |  |  |  |  |  |  |  |  |  |  |  |  |  |  |  |
| BIO17 |  |  |  |  |  |  |  |  | -0.14 | 0.06 | **0.041** | 14.04 |  |  |  |  |
| BIO18 |  |  |  |  | 0.00 | 0.06 | 0.946 | 12.39 |  |  |  |  |  |  |  |  |
| BIO19 | -0.02 | 0.03 | 0.488 | 3.19 |  |  |  |  |  |  |  |  |  |  |  |  |

**Supplementary Table 18**. Results of Response screening (calculated in JMP 13.0) for all potential predictor variables considered in this study, for the response variable ‘*Bd* infection intensity’ of the full data set of 791 amphibian skin bacterial communities. Variables sorted by decreasing LogWorth (-log10 transformation of P-values); higher values correspond to lower P-values. FDR refers to P-values adjusted to control the false discovery rate for multiple tests, calculated using the Benjamini-Hochberg technique. Bio 1 = Annual Mean Temperature. Bio 2 = Mean Diurnal Range. Bio 3 = Isothermality. Bio 4 = Temperature Seasonality. Bio 5 = Maximum Temperature of Warmest Month. Bio 6 = Minimum Temperature of Coldest Month. Bio 7 = Annual Temperature Range. Bio 8 = Mean Temperature of Wettest Quarter. Bio 9 = Mean Temperature of Driest Quarter. Bio 10 = Mean Temperature of Warmest Quarter. Bio 11 = Mean Temperature of Coldest Quarter. Bio 12 = Annual Precipitation. Bio 13 = Precipitation of Wettest Month. Bio 14 = Precipitation of Driest Month. Bio 15 = Precipitation Seasonality. Bio 16 = Precipitation of Wettest Quarter. Bio 17 = Precipitation of Driest Quarter. Bio 18 = Precipitation of Warmest Quarter. Bio 19 = Precipitation of Coldest Quarter. All variables were scaled before analyses.

| Y (response variable) | X (predictor variable) | P value | Log Worth (P value) | FDR P value | Log Worth (FDR P value) | Effect Size | F Ratio | R² |
| --- | --- | --- | --- | --- | --- | --- | --- | --- |
| **Bd intensity** | **BIO14** | **0.000** | **4.723** | **0.000** | **4.321** | **0.151** | **18.520** | **0.023** |
| **Bd intensity** | **BIO17** | **0.000** | **4.328** | **0.000** | **3.966** | **0.144** | **16.754** | **0.021** |
| **Bd intensity** | **BIO2** | **0.001** | **3.224** | **0.001** | **2.931** | **0.122** | **11.881** | **0.015** |
| **Bd intensity** | **BIO12** | **0.002** | **2.765** | **0.003** | **2.525** | **0.111** | **9.896** | **0.012** |
| **Bd intensity** | **BIO7** | **0.006** | **2.192** | **0.011** | **1.972** | **0.097** | **7.465** | **0.009** |
| Bd intensity | BIO15 | 0.008 | 2.114 | 0.013 | 1.902 | 0.095 | 7.141 | 0.009 |
| Bd intensity | latitude | 0.046 | 1.341 | 0.067 | 1.173 | 0.071 | 4.010 | 0.005 |
| Bd intensity | BIO6 | 0.058 | 1.233 | 0.085 | 1.071 | 0.067 | 3.590 | 0.005 |
| Bd intensity | OTU | 0.061 | 1.218 | 0.086 | 1.068 | 0.067 | 3.533 | 0.004 |
| Bd intensity | BIO18 | 0.070 | 1.157 | 0.097 | 1.012 | 0.064 | 3.299 | 0.004 |
| Bd intensity | BIO4 | 0.077 | 1.113 | 0.105 | 0.980 | 0.063 | 3.135 | 0.004 |
| Bd intensity | BIO9 | 0.091 | 1.042 | 0.122 | 0.914 | 0.060 | 2.867 | 0.004 |
| Bd intensity | BIO11 | 0.120 | 0.920 | 0.157 | 0.803 | 0.055 | 2.419 | 0.003 |
| Bd intensity | BIO1 | 0.210 | 0.677 | 0.266 | 0.576 | 0.045 | 1.571 | 0.002 |
| Bd intensity | BIO3 | 0.455 | 0.342 | 0.508 | 0.294 | 0.027 | 0.558 | 0.001 |
| Bd intensity | BIO10 | 0.475 | 0.324 | 0.524 | 0.281 | 0.025 | 0.512 | 0.001 |
| Bd intensity | #simpson_e | 0.522 | 0.282 | 0.565 | 0.248 | 0.023 | 0.410 | 0.001 |
| Bd intensity | BIO8 | 0.692 | 0.160 | 0.741 | 0.130 | 0.014 | 0.157 | 0.000 |
| Bd intensity | BIO5 | 0.727 | 0.138 | 0.771 | 0.113 | 0.012 | 0.122 | 0.000 |
| Bd intensity | elevation | 0.735 | 0.134 | 0.772 | 0.113 | 0.012 | 0.114 | 0.000 |
| Bd intensity | BIO13 | 0.851 | 0.070 | 0.876 | 0.058 | 0.007 | 0.035 | 0.000 |
| Bd intensity | BIO19 | 0.922 | 0.035 | 0.940 | 0.027 | 0.003 | 0.010 | 0.000 |
| Bd intensity | BIO16 | 0.949 | 0.023 | 0.958 | 0.018 | 0.002 | 0.004 | 0.000 |

**Supplementary Table 19** Results of Response screening (calculated in JMP 13.0) for all potential predictor variables considered in this study, for the response variable ‘Bd infection status’ of the full data set of 791 amphibian skin bacterial communities. Variables sorted by decreasing LogWorth (-log10 transformation of P-values); higher values correspond to lower P-values. FDR refers to P-values adjusted to control the false discovery rate for multiple tests, calculated using the Benjamini-Hochberg technique. Bio 1 = Annual Mean Temperature. Bio 2 = Mean Diurnal Range. Bio 3 = Isothermality. Bio 4 = Temperature Seasonality. Bio 5 = Maximum Temperature of Warmest Month. Bio 6 = Minimum Temperature of Coldest Month. Bio 7 = Annual Temperature Range. Bio 8 = Mean Temperature of Wettest Quarter. Bio 9 = Mean Temperature of Driest Quarter. Bio 10 = Mean Temperature of Warmest Quarter. Bio 11 = Mean Temperature of Coldest Quarter. Bio 12 = Annual Precipitation. Bio 13 = Precipitation of Wettest Month. Bio 14 = Precipitation of Driest Month. Bio 15 = Precipitation Seasonality. Bio 16 = Precipitation of Wettest Quarter. Bio 17 = Precipitation of Driest Quarter. Bio 18 = Precipitation of Warmest Quarter. Bio 19 = Precipitation of Coldest Quarter. All variables were scaled before analyses.

| Y (response variable) | X (predictor variable) | P value | Log Worth (P value) | FDR P value | Log Worth (FDR P value) | Effect Size |
| --- | --- | --- | --- | --- | --- | --- |
| **Bd status** | **BIO11** | **0.000** | **17.481** | **0.000** | 16.155 | 0.309 |
| **Bd status** | **BIO9** | **0.000** | **16.925** | **0.000** | 15.678 | 0.304 |
| **Bd status** | **BIO6** | **0.000** | **16.854** | **0.000** | 15.674 | 0.303 |
| **Bd status** | **Latitude** | **0.000** | **14.912** | **0.000** | 13.790 | 0.285 |
| **Bd status** | **BIO4** | **0.000** | **14.587** | **0.000** | 13.516 | 0.281 |
| Bd status | BIO7 | 0.000 | 13.743 | 0.000 | 12.759 | 0.272 |
| Bd status | BIO3 | 0.000 | 13.503 | 0.000 | 12.592 | 0.270 |
| Bd status | BIO1 | 0.000 | 13.405 | 0.000 | 12.526 | 0.269 |
| Bd status | BIO17 | 0.000 | 8.334 | 0.000 | 7.564 | 0.208 |
| Bd status | BIO14 | 0.000 | 8.235 | 0.000 | 7.489 | 0.207 |
| Bd status | BIO10 | 0.000 | 7.254 | 0.000 | 6.530 | 0.193 |
| Bd status | BIO8 | 0.000 | 6.297 | 0.000 | 5.700 | 0.179 |
| Bd status | BIO18 | 0.000 | 6.294 | 0.000 | 5.700 | 0.179 |
| Bd status | BIO15 | 0.000 | 6.005 | 0.000 | 5.442 | 0.174 |
| Bd status | BIO5 | 0.000 | 5.439 | 0.000 | 4.919 | 0.165 |
| Bd status | BIO2 | 0.000 | 5.324 | 0.000 | 4.817 | 0.163 |
| Bd status | Elevation | 0.000 | 4.664 | 0.000 | 4.218 | 0.151 |
| Bd status | #OTU | 0.000 | 4.138 | 0.000 | 3.736 | 0.141 |
| Bd status | BIO19 | 0.000 | 3.950 | 0.000 | 3.568 | 0.137 |
| Bd status | BIO12 | 0.017 | 1.779 | 0.026 | 1.567 | 0.085 |
| Bd status | BIO13 | 0.029 | 1.544 | 0.044 | 1.345 | 0.078 |
| Bd status | BIO16 | 0.098 | 1.011 | 0.129 | 0.877 | 0.059 |
| Bd status | Simpson’s evenness | 0.376 | 0.425 | 0.428 | 0.354 | 0.031 |

**Supplementary Table 20.** Results of separate generalized mixed models (GLMMs) testing the effect of Mean Diurnal Temperature Range (Bio2), Annual Temperature Range (Bio 7), Annual Precipitation (Bio12), Precipitation of the Driest Month (Bio14), Precipitation of the Driest Quarter (Bio17), Latitude, OTU richness (#OTUs), and Elevation on Bd infection intensity of treefrogs in the Brazilian Atlantic Forest. For each fixed factor in the nine models, intercept, coefficients, standard error and the P value are shown. Bold P values indicate a significant effect of the fixed factor. P-values ware corrected for multiple-comparisons with Bonferroni correction. Location:sampling date:host species was used as nested random factor. Bio 2 = Mean Diurnal Range. Bio 12 = Annual Precipitation. Bio 14 = Precipitation of Driest Month. Bio 17 = Precipitation of Driest Quarter. P-values ware corrected for multiple-comparisons with Bonferroni correction. All variables were scaled before analyses. N = 791.

| **Bd infection intensity** | GLMM. family = poisson | | |
| --- | --- | --- | --- |
| Fixed factors | Intercept (SE) | Coefficient  (SE) | P |
| Elevation | 0.40 (0.21) | -0.13 (0.19) | 0.525 |
| Latitude | 0.32 (0.22) | -0.12 (0.25) | 0.630 |
| #OTUs | 0.33 (0.13) | 0.00 | 0.799 |
| BIO2 | 0.29 (0.17) | 0.32 (0.17) | 0.110 |
| BIO7 | 0.28 (0.21) | 0.27 (0.24) | 0.286 |
| BIO12 | 0.33 (0.17) | 0.41 (0.16) | **0.022** |
| BIO14 | 0.24 (0.17) | 0.35 (0.15) | 0.051 |
| BIO17 | 0.26 (0.17) | 0.33 (0.15) | 0.070 |

**Supplementary Table 21.** Bacterial indicator OTUs per latitudinal or Bd prevalence group. **A** denotes the specificity of the OTU to the sample type, i.e. the probability that the OTU belongs to the target sample type. Value of 1 indicates that current OTU specific only to given sample type. **B** indicates the probability of finding the OTU in given sample type. Value of 1 indicates that current OTU is found in all of the samples in a given sample type. NAF = North Atlantic Forest. CAF = Central Atlantic Forest. SAF = South Atlantic Forest. BD_positive = Bd status is positive. BD_negative = Bd status is negative. Function = Bd inhibitory or stimulatory function of OTU.

| **Group** | **OTU** | **A** | **B** | **stat.** | **p** | **Taxa** | | | | | | | **Function** |
| --- | --- | --- | --- | --- | --- | --- | --- | --- | --- | --- | --- | --- | --- |
|  |  |  |  |  |  | **kingdom** | **phylum** | **class** | **order** | **family** | **genus** | **species** |  |
| NAF | V272 | 0.719 | 0.724 | 0.721 | 0.0001 | Bacteria | Actinobacteria | Actinobacteria | Actinomycetales | Cellulomonadaceae | *Cellulomonas* |  |  |
| NAF | V752 | 0.778 | 0.625 | 0.697 | 0.0001 | Bacteria | Proteobacteria | Betaproteobacteria | Burkholderiales | Comamonadaceae | *Variovorax* | *paradoxus* |  |
| NAF | V173 | 0.720 | 0.603 | 0.659 | 0.0001 | Bacteria | Proteobacteria | Gammaproteobacteria | Xanthomonadales | Xanthomonadaceae | *Stenotrophomonas* |  |  |
| NAF | V756 | 0.711 | 0.560 | 0.631 | 0.0001 | Bacteria | Proteobacteria | Gammaproteobacteria | Xanthomonadales | Xanthomonadaceae | *Stenotrophomonas* |  |  |
| NAF | V466 | 0.690 | 0.509 | 0.592 | 0.0001 | Bacteria | Proteobacteria | Betaproteobacteria | Burkholderiales | Comamonadaceae | *Delftia* |  |  |
| NAF | V353 | 0.809 | 0.366 | 0.545 | 0.0001 | Bacteria | Proteobacteria | Betaproteobacteria | Burkholderiales | Oxalobacteraceae | *Herbaspirillum* |  |  |
| NAF | V1413 | 0.742 | 0.323 | 0.490 | 0.0001 | Bacteria | Actinobacteria | Actinobacteria | Actinomycetales | Microbacteriaceae | *Microbacterium* |  |  |
| NAF | V384 | 0.884 | 0.203 | 0.423 | 0.0001 | Bacteria | Firmicutes | Bacilli | Bacillales | [Exiguobacteraceae] | *Exiguobacterium* |  |  |
| NAF | V535 | 0.644 | 0.190 | 0.350 | 0.0001 | Bacteria | Proteobacteria | Alphaproteobacteria | Rhizobiales | Hyphomicrobiaceae | *Devosia* |  |  |
| NAF | V21 | 0.783 | 0.129 | 0.318 | 0.0005 | Bacteria | Proteobacteria | Alphaproteobacteria | Sphingomonadales | Sphingomonadaceae | *Sphingobium* | *yanoikuyae* |  |
| NAF | V55 | 0.971 | 0.091 | 0.296 | 0.0001 | Bacteria | Bacteroidetes | Flavobacteriia | Flavobacteriales | [Weeksellaceae] | *Cloacibacterium* |  |  |
| NAF | V155 | 0.913 | 0.086 | 0.281 | 0.0001 | Bacteria | Proteobacteria | Alphaproteobacteria | Rhizobiales | Rhizobiaceae | *Agrobacterium* |  |  |
| NAF | V1579 | 0.890 | 0.082 | 0.270 | 0.0001 | Bacteria | Proteobacteria | Betaproteobacteria | Rhodocyclales | Rhodocyclaceae | *C39* |  |  |
| NAF | V1617 | 0.902 | 0.082 | 0.272 | 0.0003 | Bacteria | Proteobacteria | Gammaproteobacteria | Enterobacteriales | Enterobacteriaceae | *Pantoea* |  |  |
| NAF | V1139 | 0.704 | 0.078 | 0.234 | 0.0088 | Bacteria | Proteobacteria | Betaproteobacteria | Burkholderiales | Comamonadaceae | *Hylemonella* |  |  |
| NAF | V1881 | 0.998 | 0.078 | 0.278 | 0.0001 | Bacteria | Proteobacteria | Epsilonproteobacteria | Campylobacterales | Campylobacteraceae | *Sulfurospirillum* |  |  |
| NAF | V2387 | 0.995 | 0.078 | 0.278 | 0.0001 | Bacteria | Proteobacteria | Gammaproteobacteria | Pseudomonadales | Pseudomonadaceae |  |  |  |
| NAF | V526 | 1.000 | 0.078 | 0.279 | 0.0001 | Bacteria | Bacteroidetes | Bacteroidia | Bacteroidales | Bacteroidaceae | *Bacteroides* |  |  |
| NAF | V839 | 0.758 | 0.078 | 0.243 | 0.0001 | Bacteria | [Thermi] | Deinococci | Deinococcales | Deinococcaceae | *Deinococcus* |  |  |
| NAF | V107 | 0.772 | 0.073 | 0.238 | 0.0066 | Bacteria | Bacteroidetes | Flavobacteriia | Flavobacteriales | [Weeksellaceae] | *Chryseobacterium* |  |  |
| NAF | V130 | 0.922 | 0.073 | 0.260 | 0.0015 | Bacteria | Actinobacteria | Actinobacteria | Actinomycetales | Intrasporangiaceae | *Phycicoccus* |  |  |
| NAF | V2393 | 1.000 | 0.073 | 0.271 | 0.0001 | Bacteria | Proteobacteria | Epsilonproteobacteria | Campylobacterales | Helicobacteraceae |  |  |  |
| NAF | V2394 | 1.000 | 0.073 | 0.271 | 0.0001 | Bacteria | Bacteroidetes | Cytophagia | Cytophagales | Cytophagaceae | *Flectobacillus* |  |  |
| NAF | V434 | 0.858 | 0.073 | 0.251 | 0.0035 | Bacteria | Proteobacteria | Betaproteobacteria | Neisseriales | Neisseriaceae |  |  |  |
| NAF | V232 | 0.948 | 0.065 | 0.248 | 0.0001 | Bacteria | Proteobacteria | Betaproteobacteria | Rhodocyclales | Rhodocyclaceae | *Dechloromonas* |  |  |
| NAF | V2419 | 1.000 | 0.065 | 0.254 | 0.0001 | Bacteria | Bacteroidetes | Flavobacteriia | Flavobacteriales | Cryomorphaceae | *Fluviicola* |  |  |
| NAF | V694 | 1.000 | 0.065 | 0.254 | 0.0001 | Bacteria | Proteobacteria | Betaproteobacteria | Rhodocyclales | Rhodocyclaceae | *Zoogloea* |  |  |
| NAF | V1297 | 0.862 | 0.060 | 0.228 | 0.0002 | Bacteria | Proteobacteria | Betaproteobacteria | Burkholderiales | Comamonadaceae | *Limnohabitans* |  |  |
| NAF | V1700 | 0.729 | 0.060 | 0.210 | 0.0004 | Bacteria | Proteobacteria | Alphaproteobacteria | Rhizobiales | Methylocystaceae | *Rhodoblastus* | *acidophilus* |  |
| NAF | V1811 | 0.932 | 0.060 | 0.237 | 0.0001 | Bacteria | Proteobacteria | Gammaproteobacteria | Methylococcales | Methylococcaceae |  |  |  |
| NAF | V2338 | 1.000 | 0.060 | 0.246 | 0.0001 | Bacteria | Proteobacteria | Gammaproteobacteria | Pseudomonadales | Pseudomonadaceae | *Pseudomonas* | *thermotolerans* |  |
| NAF | V2397 | 0.991 | 0.060 | 0.245 | 0.0001 | Bacteria | Bacteroidetes | Bacteroidia | Bacteroidales | Bacteroidaceae | *Bacteroides* |  |  |
| NAF | V2398 | 1.000 | 0.060 | 0.246 | 0.0001 | Bacteria | Bacteroidetes | Bacteroidia | Bacteroidales | [Paraprevotellaceae] | *YRC22* |  |  |
| NAF | V57 | 0.567 | 0.060 | 0.185 | 0.0107 | Bacteria | Proteobacteria | Gammaproteobacteria | Xanthomonadales | Xanthomonadaceae | *Stenotrophomonas* | *acidaminiphila* |  |
| NAF | V661 | 1.000 | 0.060 | 0.246 | 0.0001 | Bacteria | Proteobacteria | Gammaproteobacteria | Methylococcales |  |  |  |  |
| NAF | V95 | 0.946 | 0.060 | 0.239 | 0.0001 | Bacteria | Bacteroidetes | Flavobacteriia | Flavobacteriales | Flavobacteriaceae | *Flavobacterium* | *succinicans* |  |
| NAF | V108 | 0.853 | 0.056 | 0.219 | 0.0003 | Bacteria | Proteobacteria | Betaproteobacteria | Burkholderiales | Comamonadaceae | *Hydrogenophaga* |  |  |
| NAF | V2421 | 1.000 | 0.056 | 0.237 | 0.0001 | Bacteria |  |  |  |  |  |  |  |
| NAF | V68 | 0.834 | 0.056 | 0.216 | 0.0001 | Bacteria | Bacteroidetes | Sphingobacteriia | Sphingobacteriales |  |  |  |  |
| NAF | V1333 | 0.859 | 0.052 | 0.211 | 0.0002 | Bacteria | Proteobacteria | Betaproteobacteria | Burkholderiales | Comamonadaceae | *Hydrogenophaga* |  |  |
| NAF | V1485 | 0.808 | 0.052 | 0.204 | 0.0073 | Bacteria | Bacteroidetes | Flavobacteriia | Flavobacteriales | [Weeksellaceae] | *Chryseobacterium* |  |  |
| NAF | V1896 | 0.916 | 0.052 | 0.218 | 0.0001 | Bacteria | Bacteroidetes | Bacteroidia | Bacteroidales |  |  |  |  |
| NAF | V1969 | 0.940 | 0.052 | 0.220 | 0.0002 | Bacteria | Bacteroidetes | Bacteroidia | Bacteroidales | Prevotellaceae | *Prevotella* |  |  |
| NAF | V2229 | 0.980 | 0.052 | 0.225 | 0.0001 | Bacteria | Bacteroidetes | Sphingobacteriia | Sphingobacteriales |  |  |  |  |
| NAF | V2449 | 1.000 | 0.052 | 0.227 | 0.0001 | Bacteria | Proteobacteria | Alphaproteobacteria | Rhodobacterales | Rhodobacteraceae | *Rhodobacter* |  |  |
| NAF | V949 | 1.000 | 0.052 | 0.227 | 0.0001 | Bacteria | Bacteroidetes | Sphingobacteriia | Sphingobacteriales |  |  |  |  |
| NAF | V1106 | 0.754 | 0.047 | 0.189 | 0.0013 | Bacteria | Proteobacteria | Alphaproteobacteria | Rhizobiales | Methylocystaceae |  |  |  |
| NAF | V1208 | 1.000 | 0.047 | 0.218 | 0.0002 | Bacteria | Bacteroidetes | Flavobacteriia | Flavobacteriales | [Weeksellaceae] | *Chryseobacterium* |  |  |
| NAF | V1298 | 0.910 | 0.047 | 0.208 | 0.0001 | Bacteria | Proteobacteria | Gammaproteobacteria | Pseudomonadales | Moraxellaceae | *Acinetobacter* |  |  |
| NAF | V1927 | 0.958 | 0.047 | 0.213 | 0.0001 | Archaea | Euryarchaeota | Methanobacteria | Methanobacteriales | Methanobacteriaceae | *Methanobacterium* |  |  |
| NAF | V2146 | 0.936 | 0.047 | 0.211 | 0.0001 | Bacteria | Proteobacteria | Betaproteobacteria | Burkholderiales | Comamonadaceae | *Rubrivivax* |  |  |
| NAF | V2420 | 1.000 | 0.047 | 0.218 | 0.0001 | Bacteria | Firmicutes | Clostridia | Clostridiales | Clostridiaceae | *Clostridium* |  |  |
| NAF | V2571 | 0.939 | 0.047 | 0.211 | 0.0001 | Bacteria | Proteobacteria | Gammaproteobacteria | Pseudomonadales | Moraxellaceae | *Acinetobacter* | *rhizosphaerae* |  |
| NAF | V486 | 0.737 | 0.047 | 0.187 | 0.0097 | Bacteria | Proteobacteria | Betaproteobacteria | Burkholderiales | Comamonadaceae | *Rubrivivax* |  |  |
| NAF | V1103 | 0.941 | 0.043 | 0.201 | 0.0001 | Bacteria | Proteobacteria | Deltaproteobacteria | Syntrophobacterales | Syntrophaceae |  |  |  |
| NAF | V1264 | 0.972 | 0.043 | 0.205 | 0.0003 | Bacteria | Proteobacteria | Gammaproteobacteria | Xanthomonadales | Sinobacteraceae |  |  |  |
| NAF | V1427 | 0.955 | 0.043 | 0.203 | 0.0003 | Bacteria | Proteobacteria | Deltaproteobacteria | Myxococcales |  |  |  |  |
| NAF | V1528 | 0.912 | 0.043 | 0.198 | 0.0010 | Bacteria | Bacteroidetes | Sphingobacteriia | Sphingobacteriales | Sphingobacteriaceae |  |  |  |
| NAF | V1606 | 0.802 | 0.043 | 0.186 | 0.0011 | Bacteria | Bacteroidetes | Flavobacteriia | Flavobacteriales | Flavobacteriaceae | *Flavobacterium* |  |  |
| NAF | V1785 | 0.889 | 0.043 | 0.196 | 0.0002 | Bacteria | Bacteroidetes | Flavobacteriia | Flavobacteriales | Flavobacteriaceae | *Flavobacterium* |  |  |
| NAF | V1848 | 0.959 | 0.043 | 0.203 | 0.0003 | Bacteria | Bacteroidetes | Cytophagia | Cytophagales | Cytophagaceae | *Hymenobacter* |  |  |
| NAF | V1899 | 0.989 | 0.043 | 0.206 | 0.0001 | Bacteria | Acidobacteria | Holophagae | Holophagales | Holophagaceae | *Geothrix* |  |  |
| NAF | V218 | 0.982 | 0.043 | 0.206 | 0.0002 | Bacteria | Bacteroidetes | Cytophagia | Cytophagales | Cytophagaceae | *Runella* |  |  |
| NAF | V2383 | 1.000 | 0.043 | 0.208 | 0.0001 | Bacteria | Proteobacteria | Betaproteobacteria | Rhodocyclales | Rhodocyclaceae | *Dechloromonas* |  |  |
| NAF | V2426 | 0.909 | 0.043 | 0.198 | 0.0001 | Bacteria | Proteobacteria | Alphaproteobacteria | Rhodospirillales | Acetobacteraceae |  |  |  |
| NAF | V2453 | 1.000 | 0.043 | 0.208 | 0.0001 | Bacteria | Elusimicrobia | Elusimicrobia | Elusimicrobiales | Elusimicrobiaceae | *Elusimicrobium* |  |  |
| NAF | V2588 | 0.939 | 0.043 | 0.201 | 0.0001 | Bacteria | Proteobacteria | Gammaproteobacteria | Salinisphaerales | Salinisphaeraceae | *Salinisphaera* |  |  |
| NAF | V793 | 1.000 | 0.043 | 0.208 | 0.0001 | Bacteria | Proteobacteria | Betaproteobacteria | Rhodocyclales | Rhodocyclaceae | *Propionivibrio* |  |  |
| NAF | V823 | 0.804 | 0.043 | 0.186 | 0.0033 | Bacteria | Proteobacteria | Betaproteobacteria | Burkholderiales | Comamonadaceae | *Comamonas* |  |  |
| NAF | V881 | 0.840 | 0.043 | 0.190 | 0.0017 | Bacteria | Proteobacteria | Betaproteobacteria | Burkholderiales | Comamonadaceae | *Methylibium* |  |  |
| NAF | V1522 | 0.729 | 0.039 | 0.168 | 0.0264 | Bacteria | Proteobacteria | Gammaproteobacteria | Methylococcales | Methylococcaceae |  |  |  |
| NAF | V1556 | 0.828 | 0.039 | 0.179 | 0.0017 | Bacteria | Actinobacteria | Actinobacteria | Actinomycetales | Pseudonocardiaceae |  |  |  |
| NAF | V1561 | 0.915 | 0.039 | 0.188 | 0.0009 | Bacteria | Proteobacteria | Betaproteobacteria | Burkholderiales | Comamonadaceae |  |  |  |
| NAF | V1682 | 0.656 | 0.039 | 0.159 | 0.0326 | Bacteria | Proteobacteria | Betaproteobacteria | Burkholderiales | Comamonadaceae |  |  |  |
| NAF | V1775 | 0.972 | 0.039 | 0.194 | 0.0001 | Bacteria | Proteobacteria | Betaproteobacteria | Burkholderiales | Comamonadaceae |  |  |  |
| NAF | V2342 | 0.981 | 0.039 | 0.195 | 0.0001 | Bacteria | Bacteroidetes | Flavobacteriia | Flavobacteriales | Flavobacteriaceae | *Flavobacterium* | *succinicans* |  |
| NAF | V2386 | 1.000 | 0.039 | 0.197 | 0.0001 | Bacteria | Firmicutes | Clostridia | OPB54 |  |  |  |  |
| NAF | V2402 | 1.000 | 0.039 | 0.197 | 0.0002 | Bacteria | Proteobacteria | Gammaproteobacteria | Methylococcales |  |  |  |  |
| NAF | V2440 | 1.000 | 0.039 | 0.197 | 0.0003 | Bacteria | Proteobacteria | Epsilonproteobacteria | Campylobacterales | Campylobacteraceae | *Arcobacter* |  |  |
| NAF | V2589 | 1.000 | 0.039 | 0.197 | 0.0003 | Bacteria | Actinobacteria | Actinobacteria | Actinomycetales | Pseudonocardiaceae | *Saccharopolyspora* |  |  |
| NAF | V401 | 0.732 | 0.039 | 0.168 | 0.0076 | Bacteria | Proteobacteria | Alphaproteobacteria | Rhizobiales |  |  |  |  |
| NAF | V553 | 0.899 | 0.039 | 0.187 | 0.0198 | Bacteria | Proteobacteria | Betaproteobacteria | Burkholderiales | Burkholderiaceae | *Salinispora* | *tropica* |  |
| NAF | V662 | 0.890 | 0.039 | 0.186 | 0.0147 | Bacteria | Proteobacteria | Betaproteobacteria | Burkholderiales | Comamonadaceae | *Curvibacter* |  |  |
| NAF | V789 | 0.703 | 0.039 | 0.165 | 0.0100 | Bacteria | Actinobacteria | Acidimicrobiia | Acidimicrobiales | C111 |  |  |  |
| NAF | V1150 | 0.734 | 0.034 | 0.159 | 0.0365 | Bacteria | Proteobacteria | Betaproteobacteria | Burkholderiales | Comamonadaceae |  |  |  |
| NAF | V1309 | 0.893 | 0.034 | 0.176 | 0.0008 | Bacteria | Actinobacteria | Thermoleophilia | Solirubrobacterales |  |  |  |  |
| NAF | V158 | 0.924 | 0.034 | 0.178 | 0.0024 | Bacteria | Proteobacteria | Gammaproteobacteria | Oceanospirillales | Alcanivoracaceae | *Alcanivorax* |  |  |
| NAF | V1648 | 1.000 | 0.034 | 0.186 | 0.0003 | Bacteria | Actinobacteria | Actinobacteria | Actinomycetales | Nocardioidaceae |  |  |  |
| NAF | V1998 | 1.000 | 0.034 | 0.186 | 0.0003 | Bacteria | Verrucomicrobia | Opitutae | Opitutales | Opitutaceae | *Opitutus* |  |  |
| NAF | V2024 | 0.941 | 0.034 | 0.180 | 0.0007 | Bacteria | Proteobacteria | Deltaproteobacteria |  |  |  |  |  |
| NAF | V2384 | 1.000 | 0.034 | 0.186 | 0.0001 | Bacteria | Proteobacteria | Alphaproteobacteria | Rhodospirillales | Rhodospirillaceae | *Azospirillum* | *amazonense* |  |
| NAF | V2391 | 1.000 | 0.034 | 0.186 | 0.0002 | Bacteria | Verrucomicrobia | [Pedosphaerae] | [Pedosphaerales] | auto67_4W |  |  |  |
| NAF | V2400 | 1.000 | 0.034 | 0.186 | 0.0002 | Bacteria | Proteobacteria | Deltaproteobacteria | Spirobacillales |  |  |  |  |
| NAF | V2404 | 1.000 | 0.034 | 0.186 | 0.0003 | Bacteria | Firmicutes | Clostridia | Clostridiales | Clostridiaceae | *Caloramator* |  |  |
| NAF | V2405 | 1.000 | 0.034 | 0.186 | 0.0002 | Bacteria | Proteobacteria | Gammaproteobacteria | Methylococcales | Crenotrichaceae | *Crenothrix* |  |  |
| NAF | V2451 | 1.000 | 0.034 | 0.186 | 0.0003 | Bacteria | Proteobacteria | Alphaproteobacteria | Rickettsiales |  |  |  |  |
| NAF | V570 | 0.929 | 0.034 | 0.179 | 0.0054 | Bacteria | Bacteroidetes | Bacteroidia | Bacteroidales | Bacteroidaceae | *Bacteroides* |  |  |
| NAF | V145 | 0.978 | 0.030 | 0.172 | 0.0003 | Bacteria | Proteobacteria | Alphaproteobacteria | Rhodobacterales | Rhodobacteraceae | *Rhodobacter* |  |  |
| NAF | V1599 | 0.749 | 0.030 | 0.150 | 0.0239 | Bacteria | Proteobacteria | Betaproteobacteria | Burkholderiales | Alcaligenaceae | *Pigmentiphaga* |  |  |
| NAF | V1709 | 0.956 | 0.030 | 0.170 | 0.0004 | Bacteria | Proteobacteria | Alphaproteobacteria | Sphingomonadales | Sphingomonadaceae | *Novosphingobium* |  |  |
| NAF | V1863 | 0.953 | 0.030 | 0.170 | 0.0006 | Bacteria | Actinobacteria | Thermoleophilia | Gaiellales |  |  |  |  |
| NAF | V2378 | 1.000 | 0.030 | 0.174 | 0.0003 | Bacteria | Proteobacteria | Gammaproteobacteria | Pseudomonadales | Moraxellaceae | *Acinetobacter* | *rhizosphaerae* |  |
| NAF | V2389 | 1.000 | 0.030 | 0.174 | 0.0003 | Archaea | Euryarchaeota | Methanobacteria | Methanobacteriales | Methanobacteriaceae | *Methanobacterium* |  |  |
| NAF | V2414 | 1.000 | 0.030 | 0.174 | 0.0002 | Bacteria | Verrucomicrobia | Opitutae | Opitutales | Opitutaceae | *Opitutus* |  |  |
| NAF | V2425 | 1.000 | 0.030 | 0.174 | 0.0004 | Bacteria | Proteobacteria | Alphaproteobacteria | Rhodobacterales | Rhodobacteraceae | *Paracoccus* | *aminovorans* |  |
| NAF | V2445 | 1.000 | 0.030 | 0.174 | 0.0002 | Bacteria | Actinobacteria | Rubrobacteria | Rubrobacterales | Rubrobacteraceae | *Rubrobacter* |  |  |
| NAF | V2450 | 1.000 | 0.030 | 0.174 | 0.0001 | Bacteria | Proteobacteria | Alphaproteobacteria | Rickettsiales |  |  |  |  |
| NAF | V2452 | 1.000 | 0.030 | 0.174 | 0.0005 | Bacteria | Proteobacteria | Betaproteobacteria | Procabacteriales | Procabacteriaceae |  |  |  |
| NAF | V2456 | 1.000 | 0.030 | 0.174 | 0.0004 | Bacteria | Proteobacteria | Epsilonproteobacteria | Campylobacterales | Helicobacteraceae | *Wolinella* | *succinogenes* |  |
| NAF | V2564 | 0.987 | 0.030 | 0.173 | 0.0276 | Bacteria | Bacteroidetes | Flavobacteriia | Flavobacteriales | Flavobacteriaceae | *Myroides* |  |  |
| NAF | V280 | 0.773 | 0.030 | 0.153 | 0.0058 | Bacteria | Bacteroidetes | Bacteroidia | Bacteroidales | Porphyromonadaceae |  |  |  |
| NAF | V364 | 0.720 | 0.030 | 0.147 | 0.0360 | Bacteria | Firmicutes | Erysipelotrichi | Erysipelotrichales | Erysipelotrichaceae | *[Eubacterium]* | *dolichum* |  |
| NAF | V704 | 0.900 | 0.030 | 0.165 | 0.0026 | Bacteria | [Thermi] | Deinococci | Thermales | Thermaceae | *Thermus* |  |  |
| NAF | V1562 | 0.938 | 0.026 | 0.156 | 0.0034 | Bacteria | Proteobacteria | Betaproteobacteria | Rhodocyclales | Rhodocyclaceae | *Dok59* |  |  |
| NAF | V1595 | 0.905 | 0.026 | 0.153 | 0.0055 | Bacteria | Proteobacteria | Gammaproteobacteria | Xanthomonadales | Sinobacteraceae |  |  |  |
| NAF | V1699 | 0.944 | 0.026 | 0.156 | 0.0023 | Bacteria | Proteobacteria | Alphaproteobacteria | Rhodospirillales | Rhodospirillaceae | *Azospirillum* | *amazonense* |  |
| NAF | V1968 | 0.962 | 0.026 | 0.158 | 0.0010 | Bacteria | Proteobacteria | Epsilonproteobacteria | Campylobacterales | Helicobacteraceae | *Sulfuricurvum* | *kujiense* |  |
| NAF | V1972 | 1.000 | 0.026 | 0.161 | 0.0008 | Bacteria | Cyanobacteria | 4C0d-2 | YS2 |  |  |  |  |
| NAF | V2367 | 0.974 | 0.026 | 0.159 | 0.0015 | Bacteria | Bacteroidetes | Sphingobacteriia | Sphingobacteriales | Sphingobacteriaceae | *Pedobacter* |  |  |
| NAF | V2388 | 1.000 | 0.026 | 0.161 | 0.0008 | Bacteria | Proteobacteria | Betaproteobacteria |  |  |  |  |  |
| NAF | V2395 | 1.000 | 0.026 | 0.161 | 0.0010 | Bacteria | Acidobacteria | Holophagae | Holophagales | Holophagaceae |  |  |  |
| NAF | V2396 | 1.000 | 0.026 | 0.161 | 0.0006 | Bacteria | Planctomycetes | vadinHA49 | DH61 |  |  |  |  |
| NAF | V2401 | 1.000 | 0.026 | 0.161 | 0.0008 | Bacteria | Proteobacteria | Deltaproteobacteria | Spirobacillales |  |  |  |  |
| NAF | V2408 | 1.000 | 0.026 | 0.161 | 0.0014 | Bacteria | Proteobacteria | Deltaproteobacteria | *Bd*ellovibrionales | *Bd*ellovibrionaceae | *Bdellovibrio* |  |  |
| NAF | V2410 | 1.000 | 0.026 | 0.161 | 0.0008 | Bacteria | Firmicutes | Erysipelotrichi | Erysipelotrichales | Erysipelotrichaceae | *Clostridium* |  |  |
| NAF | V2454 | 1.000 | 0.026 | 0.161 | 0.0008 | Bacteria | Firmicutes | Clostridia | Clostridiales | Veillonellaceae | *Succinispira* | *mobilis* |  |
| NAF | V2457 | 1.000 | 0.026 | 0.161 | 0.0005 | Bacteria | Firmicutes | Clostridia | Clostridiales | Lachnospiraceae |  |  |  |
| NAF | V2590 | 1.000 | 0.026 | 0.161 | 0.0011 | Bacteria | Firmicutes | Bacilli | Bacillales | Bacillaceae | *Virgibacillus* |  |  |
| NAF | V1168 | 0.853 | 0.022 | 0.136 | 0.0398 | Bacteria | Proteobacteria | Gammaproteobacteria | Xanthomonadales | Xanthomonadaceae | *Stenotrophomonas* |  |  |
| NAF | V1179 | 0.807 | 0.022 | 0.132 | 0.0321 | Bacteria | Bacteroidetes | Cytophagia | Cytophagales | Cytophagaceae | *Hymenobacter* |  |  |
| NAF | V1196 | 0.772 | 0.022 | 0.129 | 0.0461 | Bacteria | Proteobacteria | Alphaproteobacteria | Caulobacterales | Caulobacteraceae | *Brevundimonas* | *diminuta* |  |
| NAF | V1261 | 0.956 | 0.022 | 0.144 | 0.0049 | Bacteria | Proteobacteria | Betaproteobacteria | Burkholderiales | Comamonadaceae | *Rubrivivax* |  |  |
| NAF | V1497 | 0.972 | 0.022 | 0.145 | 0.0079 | Bacteria | Proteobacteria | Gammaproteobacteria | Xanthomonadales | Xanthomonadaceae | *Stenotrophomonas* |  |  |
| NAF | V1535 | 1.000 | 0.022 | 0.147 | 0.0025 | Bacteria | Bacteroidetes | Sphingobacteriia | Sphingobacteriales | Sphingobacteriaceae |  |  |  |
| NAF | V1585 | 0.953 | 0.022 | 0.143 | 0.0073 | Bacteria | Proteobacteria | Gammaproteobacteria | Methylococcales | Methylococcaceae | *Methylocaldum* |  |  |
| NAF | V1644 | 0.773 | 0.022 | 0.129 | 0.0243 | Bacteria | Verrucomicrobia | Opitutae | [Cerasicoccales] | [Cerasicoccaceae] |  |  |  |
| NAF | V1711 | 0.921 | 0.022 | 0.141 | 0.0106 | Bacteria | Bacteroidetes | Cytophagia | Cytophagales | Cytophagaceae |  |  |  |
| NAF | V187 | 0.962 | 0.022 | 0.144 | 0.0045 | Bacteria | Proteobacteria | Betaproteobacteria | Rhodocyclales | Rhodocyclaceae | *Dechloromonas* |  |  |
| NAF | V1897 | 1.000 | 0.022 | 0.147 | 0.0023 | Bacteria | Proteobacteria | Gammaproteobacteria | Methylococcales | Methylococcaceae |  |  |  |
| NAF | V1963 | 0.953 | 0.022 | 0.143 | 0.0066 | Bacteria | Proteobacteria | Alphaproteobacteria | Sphingomonadales | Sphingomonadaceae | *Novosphingobium* |  |  |
| NAF | V2375 | 1.000 | 0.022 | 0.147 | 0.0021 | Bacteria | Firmicutes | Bacilli | Bacillales | Paenibacillaceae | *Saccharibacillus* | *kuerlensis* |  |
| NAF | V2385 | 1.000 | 0.022 | 0.147 | 0.0031 | Bacteria | Proteobacteria | Betaproteobacteria | Rhodocyclales | Rhodocyclaceae |  |  |  |
| NAF | V2399 | 1.000 | 0.022 | 0.147 | 0.0030 | Bacteria | Firmicutes | Clostridia | Clostridiales | Clostridiaceae | *Clostridium* |  |  |
| NAF | V2409 | 1.000 | 0.022 | 0.147 | 0.0026 | Bacteria | Proteobacteria | Gammaproteobacteria | Methylococcales | Methylococcaceae | *Methylomonas* |  |  |
| NAF | V2417 | 1.000 | 0.022 | 0.147 | 0.0023 | Bacteria | Planctomycetes | OM190 | CL500-15 |  |  |  |  |
| NAF | V2424 | 1.000 | 0.022 | 0.147 | 0.0025 | Bacteria | Proteobacteria | Betaproteobacteria | Burkholderiales | Comamonadaceae |  |  |  |
| NAF | V2442 | 1.000 | 0.022 | 0.147 | 0.0030 | Bacteria | Proteobacteria | Epsilonproteobacteria | Campylobacterales | Campylobacteraceae |  |  |  |
| NAF | V2455 | 1.000 | 0.022 | 0.147 | 0.0037 | Bacteria | Proteobacteria | Deltaproteobacteria | *Bd*ellovibrionales | Bacteriovoracaceae |  |  |  |
| NAF | V2537 | 1.000 | 0.022 | 0.147 | 0.0020 | Bacteria | Proteobacteria | Gammaproteobacteria | Xanthomonadales | Xanthomonadaceae | *Stenotrophomonas* |  |  |
| NAF | V2542 | 1.000 | 0.022 | 0.147 | 0.0024 | Bacteria | Verrucomicrobia | Verrucomicrobiae | Verrucomicrobiales | Verrucomicrobiaceae | *Akkermansia* |  |  |
| NAF | V267 | 0.834 | 0.022 | 0.134 | 0.0305 | Bacteria | Proteobacteria | Alphaproteobacteria | Rhizobiales | Xanthobacteraceae |  |  |  |
| NAF | V414 | 0.847 | 0.022 | 0.135 | 0.0352 | Bacteria | Bacteroidetes | Bacteroidia | Bacteroidales | Porphyromonadaceae | *Parabacteroides* |  |  |
| NAF | V660 | 0.944 | 0.022 | 0.143 | 0.0087 | Bacteria | Proteobacteria | Alphaproteobacteria | Sphingomonadales | Sphingomonadaceae | *Novosphingobium* |  |  |
| NAF | V1077 | 1.000 | 0.017 | 0.131 | 0.0084 | Bacteria | Bacteroidetes | Sphingobacteriia | Sphingobacteriales | Sphingobacteriaceae |  |  |  |
| NAF | V1707 | 0.902 | 0.017 | 0.125 | 0.0411 | Bacteria | Proteobacteria | Betaproteobacteria | Burkholderiales |  |  |  |  |
| NAF | V1718 | 0.921 | 0.017 | 0.126 | 0.0215 | Bacteria | Actinobacteria | Actinobacteria | Actinomycetales |  |  |  |  |
| NAF | V1882 | 1.000 | 0.017 | 0.131 | 0.0089 | Bacteria | Proteobacteria | Deltaproteobacteria | Myxococcales |  |  |  |  |
| NAF | V1891 | 1.000 | 0.017 | 0.131 | 0.0094 | Bacteria | Proteobacteria | Gammaproteobacteria | Methylococcales | Methylococcaceae | *Methylocaldum* |  |  |
| NAF | V2031 | 0.938 | 0.017 | 0.127 | 0.0192 | Bacteria | Bacteroidetes | Bacteroidia | Bacteroidales |  |  |  |  |
| NAF | V2290 | 0.921 | 0.017 | 0.126 | 0.0212 | Bacteria | Acidobacteria | RB25 |  |  |  |  |  |
| NAF | V2300 | 1.000 | 0.017 | 0.131 | 0.0101 | Bacteria | Proteobacteria | Betaproteobacteria | ASSO-13 |  |  |  |  |
| NAF | V2301 | 0.870 | 0.017 | 0.122 | 0.0291 | Bacteria | Proteobacteria | Betaproteobacteria | Burkholderiales | Oxalobacteraceae | *Janthinobacterium* |  |  |
| NAF | V2323 | 1.000 | 0.017 | 0.131 | 0.0106 | Bacteria | Proteobacteria | Gammaproteobacteria | Xanthomonadales | Xanthomonadaceae | *Stenotrophomonas* |  |  |
| NAF | V2326 | 0.870 | 0.017 | 0.122 | 0.0290 | Bacteria | Proteobacteria | Alphaproteobacteria | Rhizobiales | Phyllobacteriaceae |  |  |  |
| NAF | V2330 | 0.938 | 0.017 | 0.127 | 0.0206 | Bacteria | Chloroflexi | Anaerolineae | CFB-26 |  |  |  |  |
| NAF | V2376 | 1.000 | 0.017 | 0.131 | 0.0123 | Bacteria | Acidobacteria | [Chloracidobacteria] | RB41 | Ellin6075 |  |  |  |
| NAF | V2392 | 1.000 | 0.017 | 0.131 | 0.0093 | Bacteria | Firmicutes | Clostridia | OPB54 |  |  |  |  |
| NAF | V2407 | 1.000 | 0.017 | 0.131 | 0.0104 | Bacteria | Proteobacteria | Gammaproteobacteria | Methylococcales | Crenotrichaceae | *Crenothrix* |  |  |
| NAF | V2418 | 1.000 | 0.017 | 0.131 | 0.0091 | Bacteria | Verrucomicrobia | Verrucomicrobiae | Verrucomicrobiales | Verrucomicrobiaceae | *Prosthecobacter* | *debontii* |  |
| NAF | V2422 | 1.000 | 0.017 | 0.131 | 0.0105 | Bacteria | Verrucomicrobia | Verruco-5 | WCHB1-41 | RFP12 |  |  |  |
| NAF | V2429 | 1.000 | 0.017 | 0.131 | 0.0083 | Bacteria | Gemmatimonadetes | Gemmatimonadetes |  |  |  |  |  |
| NAF | V2432 | 1.000 | 0.017 | 0.131 | 0.0084 | Bacteria | Gemmatimonadetes | Gemmatimonadetes |  |  |  |  |  |
| NAF | V2439 | 1.000 | 0.017 | 0.131 | 0.0099 | Bacteria | Proteobacteria | Alphaproteobacteria | Sphingomonadales | Sphingomonadaceae | *Sphingomonas* |  |  |
| NAF | V2444 | 1.000 | 0.017 | 0.131 | 0.0101 | Bacteria | Firmicutes | Clostridia | Clostridiales | Clostridiaceae | *Clostridium* |  |  |
| NAF | V2448 | 1.000 | 0.017 | 0.131 | 0.0102 | Bacteria | Proteobacteria | Alphaproteobacteria | Rhizobiales | Methylocystaceae |  |  |  |
| NAF | V2459 | 1.000 | 0.017 | 0.131 | 0.0104 | Bacteria | Proteobacteria | Alphaproteobacteria | Rhizobiales | Methylocystaceae |  |  |  |
| NAF | V2462 | 1.000 | 0.017 | 0.131 | 0.0115 | Bacteria | Proteobacteria | Alphaproteobacteria | Rickettsiales | Rickettsiaceae |  |  |  |
| NAF | V2474 | 1.000 | 0.017 | 0.131 | 0.0093 | Bacteria | Proteobacteria | Gammaproteobacteria | Xanthomonadales | Sinobacteraceae |  |  |  |
| NAF | V2478 | 1.000 | 0.017 | 0.131 | 0.0095 | Bacteria | Proteobacteria | Deltaproteobacteria | Desulfuromonadales | Geobacteraceae | *Geobacter* |  |  |
| NAF | V2569 | 1.000 | 0.017 | 0.131 | 0.0110 | Bacteria | Bacteroidetes | Sphingobacteriia | Sphingobacteriales | Sphingobacteriaceae | *Sphingobacterium* |  |  |
| NAF | V2573 | 1.000 | 0.017 | 0.131 | 0.0098 | Bacteria | Proteobacteria | Gammaproteobacteria | Xanthomonadales | Xanthomonadaceae | *Stenotrophomonas* |  |  |
| NAF | V439 | 0.863 | 0.017 | 0.122 | 0.0479 | Bacteria | Firmicutes | Clostridia | Clostridiales | Ruminococcaceae | *Oscillospira* |  |  |
| NAF | V453 | 1.000 | 0.017 | 0.131 | 0.0097 | Bacteria | Bacteroidetes | Bacteroidia | Bacteroidales | Bacteroidaceae | *Bacteroides* |  |  |
| NAF | V516 | 0.978 | 0.017 | 0.130 | 0.0226 | Bacteria | Proteobacteria | Betaproteobacteria | Burkholderiales | Comamonadaceae | *Methylibium* |  |  |
| NAF | V699 | 0.899 | 0.017 | 0.125 | 0.0449 | Bacteria | Proteobacteria | Betaproteobacteria | Burkholderiales | Burkholderiaceae | *Burkholderia* | *andropogonis* |  |
| NAF | V775 | 0.953 | 0.017 | 0.128 | 0.0248 | Bacteria | Proteobacteria | Betaproteobacteria | Rhodocyclales | Rhodocyclaceae | *Uliginosibacterium* |  |  |
| NAF | V787 | 0.987 | 0.017 | 0.130 | 0.0191 | Bacteria | Proteobacteria | Alphaproteobacteria | Rhizobiales | Methylocystaceae |  |  |  |
| NAF | V950 | 1.000 | 0.017 | 0.131 | 0.0095 | Bacteria | Proteobacteria | Deltaproteobacteria | Myxococcales |  |  |  |  |
| NAF | V1255 | 1.000 | 0.013 | 0.114 | 0.0389 | Bacteria | Proteobacteria | Alphaproteobacteria | Sphingomonadales |  |  |  |  |
| NAF | V1307 | 1.000 | 0.013 | 0.114 | 0.0380 | Bacteria | WPS-2 |  |  |  |  |  |  |
| NAF | V1383 | 0.949 | 0.013 | 0.111 | 0.0415 | Bacteria | Planctomycetes | OM190 | CL500-15 |  |  |  |  |
| NAF | V1671 | 1.000 | 0.013 | 0.114 | 0.0360 | Bacteria | Actinobacteria | Actinobacteria | Actinomycetales | Nocardioidaceae | *Pimelobacter* |  |  |
| NAF | V1729 | 1.000 | 0.013 | 0.114 | 0.0367 | Bacteria | Proteobacteria | Gammaproteobacteria | Xanthomonadales | Sinobacteraceae | *Nevskia* | *ramosa* |  |
| NAF | V1912 | 1.000 | 0.013 | 0.114 | 0.0353 | Bacteria | Bacteroidetes | Sphingobacteriia | Sphingobacteriales | Sphingobacteriaceae |  |  |  |
| NAF | V2030 | 1.000 | 0.013 | 0.114 | 0.0380 | Bacteria | Bacteroidetes | Bacteroidia | Bacteroidales | Bacteroidaceae | *Bacteroides* | *fragilis* |  |
| NAF | V2166 | 1.000 | 0.013 | 0.114 | 0.0398 | Bacteria | Proteobacteria | Alphaproteobacteria | Rhizobiales | Hyphomicrobiaceae | *Rhodoplanes* |  |  |
| NAF | V2205 | 1.000 | 0.013 | 0.114 | 0.0362 | Bacteria | Actinobacteria | Actinobacteria |  |  |  |  |  |
| NAF | V2379 | 1.000 | 0.013 | 0.114 | 0.0338 | Bacteria | Firmicutes | Clostridia | Clostridiales | Veillonellaceae |  |  |  |
| NAF | V2406 | 1.000 | 0.013 | 0.114 | 0.0362 | Bacteria | Proteobacteria | Gammaproteobacteria | Methylococcales | Methylococcaceae | *Methylomonas* |  |  |
| NAF | V2411 | 1.000 | 0.013 | 0.114 | 0.0332 | Bacteria | Proteobacteria | Deltaproteobacteria | Myxococcales | Polyangiaceae |  |  |  |
| NAF | V2412 | 1.000 | 0.013 | 0.114 | 0.0355 | Bacteria | Acidobacteria | [Chloracidobacteria] | RB41 | Ellin6075 |  |  |  |
| NAF | V2415 | 1.000 | 0.013 | 0.114 | 0.0371 | Bacteria | Proteobacteria | Deltaproteobacteria | Myxococcales |  |  |  |  |
| NAF | V2416 | 1.000 | 0.013 | 0.114 | 0.0382 | Bacteria | Proteobacteria | Gammaproteobacteria | Methylococcales | Methylococcaceae |  |  |  |
| NAF | V2427 | 1.000 | 0.013 | 0.114 | 0.0388 | Bacteria | Verrucomicrobia | [Pedosphaerae] | [Pedosphaerales] | auto67_4W |  |  |  |
| NAF | V2430 | 1.000 | 0.013 | 0.114 | 0.0359 | Bacteria | Firmicutes | Clostridia | OPB54 |  |  |  |  |
| NAF | V2431 | 1.000 | 0.013 | 0.114 | 0.0330 | Bacteria | Proteobacteria | Deltaproteobacteria | Myxococcales |  |  |  |  |
| NAF | V2435 | 1.000 | 0.013 | 0.114 | 0.0364 | Bacteria |  |  |  |  |  |  |  |
| NAF | V2436 | 1.000 | 0.013 | 0.114 | 0.0383 | Bacteria | Proteobacteria | Epsilonproteobacteria | Campylobacterales | Helicobacteraceae | *Sulfuricurvum* | *kujiense* |  |
| NAF | V2437 | 1.000 | 0.013 | 0.114 | 0.0378 | Bacteria | Actinobacteria | Actinobacteria | Actinomycetales | Cellulomonadaceae | *Cellulomonas* |  |  |
| NAF | V2438 | 1.000 | 0.013 | 0.114 | 0.0360 | Bacteria | Bacteroidetes | Bacteroidia | Bacteroidales | Porphyromonadaceae | *Paludibacter* |  |  |
| NAF | V2443 | 1.000 | 0.013 | 0.114 | 0.0358 | Bacteria | Bacteroidetes | Flavobacteriia | Flavobacteriales | Flavobacteriaceae | *Flavobacterium* |  |  |
| NAF | V2460 | 1.000 | 0.013 | 0.114 | 0.0360 | Bacteria | Verrucomicrobia | [Pedosphaerae] | [Pedosphaerales] | auto67_4W |  |  |  |
| NAF | V2472 | 1.000 | 0.013 | 0.114 | 0.0352 | Bacteria | Actinobacteria | Actinobacteria | Actinomycetales |  |  |  |  |
| NAF | V2473 | 1.000 | 0.013 | 0.114 | 0.0360 | Bacteria | Proteobacteria | Betaproteobacteria |  |  |  |  |  |
| NAF | V2481 | 1.000 | 0.013 | 0.114 | 0.0347 | Bacteria | Proteobacteria | Betaproteobacteria | Neisseriales | Neisseriaceae | *Chitinibacter* | *tainanensis* |  |
| NAF | V2492 | 1.000 | 0.013 | 0.114 | 0.0383 | Bacteria | Proteobacteria | Deltaproteobacteria | Desulfovibrionales | Desulfovibrionaceae |  |  |  |
| NAF | V2501 | 1.000 | 0.013 | 0.114 | 0.0384 | Bacteria | Proteobacteria | Betaproteobacteria | Burkholderiales |  |  |  |  |
| NAF | V2509 | 1.000 | 0.013 | 0.114 | 0.0389 | Bacteria | Actinobacteria | Actinobacteria | Actinomycetales | ACK-M1 |  |  |  |
| NAF | V2511 | 1.000 | 0.013 | 0.114 | 0.0359 | Bacteria | Verrucomicrobia | [Spartobacteria] | [Chthoniobacterales] | [Chthoniobacteraceae] |  |  |  |
| NAF | V2514 | 1.000 | 0.013 | 0.114 | 0.0357 | Bacteria | Proteobacteria | Gammaproteobacteria |  |  |  |  |  |
| NAF | V2519 | 1.000 | 0.013 | 0.114 | 0.0385 | Bacteria | Actinobacteria | Actinobacteria | Actinomycetales | Frankiaceae |  |  |  |
| NAF | V2547 | 1.000 | 0.013 | 0.114 | 0.0380 | Bacteria | Firmicutes | Clostridia | Clostridiales | Veillonellaceae |  |  |  |
| NAF | V2548 | 1.000 | 0.013 | 0.114 | 0.0357 | Bacteria | Bacteroidetes | Bacteroidia | Bacteroidales | Bacteroidaceae | *Bacteroides* | *caccae* |  |
| NAF | V294 | 1.000 | 0.013 | 0.114 | 0.0320 | Bacteria | Firmicutes | Clostridia | Clostridiales | Clostridiaceae | *Clostridium* | *intestinale* |  |
| CAF | V47 | 0.802 | 0.558 | 0.669 | 0.0001 | Bacteria | Proteobacteria | Gammaproteobacteria | Pseudomonadales | Pseudomonadaceae | *Pseudomonas* |  |  |
| CAF | V224 | 0.768 | 0.445 | 0.584 | 0.0001 | Bacteria | Actinobacteria | Actinobacteria | Actinomycetales | Microbacteriaceae | *Microbacterium* | *maritypicum* |  |
| CAF | V477 | 0.994 | 0.314 | 0.558 | 0.0001 | Bacteria | Proteobacteria | Gammaproteobacteria | Pseudomonadales | Pseudomonadaceae |  |  |  |
| CAF | V795 | 0.923 | 0.275 | 0.504 | 0.0001 | Bacteria | Proteobacteria | Gammaproteobacteria | Xanthomonadales | Xanthomonadaceae | *Stenotrophomonas* | *maltophilia* |  |
| CAF | V323 | 0.959 | 0.234 | 0.474 | 0.0001 | Bacteria | Proteobacteria | Gammaproteobacteria | Pseudomonadales | Moraxellaceae | *Acinetobacter* | *lwoffii* |  |
| CAF | V452 | 0.831 | 0.193 | 0.400 | 0.0002 | Bacteria | Proteobacteria | Betaproteobacteria | Burkholderiales | Oxalobacteraceae | *Janthinobacterium* |  |  |
| CAF | V373 | 0.839 | 0.190 | 0.399 | 0.0001 | Bacteria | Actinobacteria | Actinobacteria | Actinomycetales | Intrasporangiaceae |  |  |  |
| CAF | V883 | 0.960 | 0.175 | 0.410 | 0.0001 | Bacteria | Actinobacteria | Actinobacteria | Actinomycetales |  |  |  |  |
| CAF | V1474 | 0.988 | 0.159 | 0.397 | 0.0001 | Bacteria | Proteobacteria | Gammaproteobacteria | Pseudomonadales | Pseudomonadaceae |  |  |  |
| CAF | V857 | 0.858 | 0.154 | 0.364 | 0.0001 | Bacteria | Proteobacteria | Alphaproteobacteria | Rhizobiales | Methylobacteriaceae | *Methylobacterium* | *adhaesivum* |  |
| CAF | V505 | 0.810 | 0.136 | 0.332 | 0.0001 | Bacteria | Actinobacteria | Actinobacteria | Actinomycetales | Nocardiaceae | *Rhodococcus* |  |  |
| CAF | V313 | 0.986 | 0.136 | 0.367 | 0.0001 | Bacteria | Actinobacteria | Rubrobacteria | Rubrobacterales | Rubrobacteraceae | *Rubrobacter* |  |  |
| CAF | V132 | 0.835 | 0.126 | 0.324 | 0.0004 | Bacteria | Proteobacteria | Betaproteobacteria | Rhodocyclales | Rhodocyclaceae | *Hydrogenophilus* |  |  |
| CAF | V586 | 0.972 | 0.108 | 0.324 | 0.0001 | Bacteria | Proteobacteria | Gammaproteobacteria | Xanthomonadales | Xanthomonadaceae |  |  |  |
| CAF | V585 | 0.949 | 0.098 | 0.304 | 0.0001 | Bacteria | Proteobacteria | Betaproteobacteria | Burkholderiales | Comamonadaceae |  |  |  |
| CAF | V15 | 0.957 | 0.098 | 0.306 | 0.0003 | Bacteria | Bacteroidetes | Flavobacteriia | Flavobacteriales | [Weeksellaceae] | *Chryseobacterium* |  |  |
| CAF | V1023 | 0.783 | 0.098 | 0.277 | 0.0008 | Bacteria | Proteobacteria | Alphaproteobacteria | Sphingomonadales | Sphingomonadaceae | *Sphingomonas* |  |  |
| CAF | V1328 | 0.748 | 0.093 | 0.263 | 0.0006 | Bacteria | Proteobacteria | Betaproteobacteria | Burkholderiales | Comamonadaceae | *Comamonas* |  |  |
| CAF | V1389 | 0.964 | 0.087 | 0.290 | 0.0001 | Bacteria | Proteobacteria | Gammaproteobacteria | Pseudomonadales | Pseudomonadaceae | *Pseudomonas* |  |  |
| CAF | V813 | 0.844 | 0.082 | 0.264 | 0.0001 | Bacteria | Proteobacteria | Betaproteobacteria | Burkholderiales | Comamonadaceae | *Polaromonas* |  |  |
| CAF | V338 | 0.979 | 0.077 | 0.275 | 0.0008 | Bacteria | Firmicutes | Bacilli | Bacillales | Staphylococcaceae | *Staphylococcus* | *equorum* |  |
| CAF | V73 | 0.773 | 0.077 | 0.244 | 0.0009 | Bacteria | Firmicutes | Bacilli | Bacillales | Paenibacillaceae | *Paenibacillus* | *amylolyticus* |  |
| CAF | V2176 | 1.000 | 0.075 | 0.273 | 0.0001 | Bacteria | Proteobacteria | Betaproteobacteria | Rhodocyclales | Rhodocyclaceae |  |  |  |
| CAF | V1468 | 0.772 | 0.069 | 0.232 | 0.0016 | Bacteria | Actinobacteria | Actinobacteria | Actinomycetales | Nocardiaceae |  |  |  |
| CAF | V334 | 0.968 | 0.069 | 0.259 | 0.0001 | Bacteria | Proteobacteria | Alphaproteobacteria | Rhizobiales | Methylocystaceae | *Pleomorphomonas* |  |  |
| CAF | V702 | 0.990 | 0.069 | 0.262 | 0.0001 | Bacteria | Proteobacteria | Gammaproteobacteria | Pseudomonadales | Pseudomonadaceae | *Pseudomonas* | *viridiflava* |  |
| CAF | V217 | 0.907 | 0.067 | 0.246 | 0.0013 | Bacteria | Proteobacteria | Betaproteobacteria | Neisseriales | Neisseriaceae | *Vogesella* |  |  |
| CAF | V1183 | 0.994 | 0.062 | 0.248 | 0.0004 | Bacteria | Firmicutes | Bacilli | Bacillales |  |  |  |  |
| CAF | V540 | 0.778 | 0.062 | 0.219 | 0.0418 | Bacteria | Proteobacteria | Gammaproteobacteria | Pseudomonadales | Pseudomonadaceae | *Pseudomonas* | *viridiflava* |  |
| CAF | V1233 | 0.865 | 0.057 | 0.221 | 0.0010 | Bacteria | Proteobacteria | Betaproteobacteria | Burkholderiales | Burkholderiaceae | *Lautropia* |  |  |
| CAF | V48 | 0.853 | 0.057 | 0.220 | 0.0029 | Bacteria | Proteobacteria | Gammaproteobacteria | Pseudomonadales | Pseudomonadaceae |  |  |  |
| CAF | V818 | 0.858 | 0.057 | 0.220 | 0.0045 | Bacteria | Proteobacteria | Alphaproteobacteria | Rhizobiales | Methylobacteriaceae | *Methylobacterium* | *adhaesivum* |  |
| CAF | V1444 | 0.886 | 0.054 | 0.219 | 0.0103 | Bacteria | Proteobacteria | Gammaproteobacteria | Xanthomonadales | Xanthomonadaceae | *Xanthomonas* |  |  |
| CAF | V2089 | 0.909 | 0.054 | 0.222 | 0.0043 | Bacteria | Bacteroidetes | Bacteroidia | Bacteroidales | Bacteroidaceae | *Bacteroides* |  |  |
| CAF | V2184 | 0.963 | 0.054 | 0.228 | 0.0002 | Bacteria | Proteobacteria | Alphaproteobacteria | Rhizobiales |  |  |  |  |
| CAF | V802 | 0.814 | 0.054 | 0.210 | 0.0018 | Bacteria | Proteobacteria | Alphaproteobacteria | Rhizobiales | Methylobacteriaceae | *Methylobacterium* |  |  |
| CAF | V955 | 0.724 | 0.054 | 0.198 | 0.0078 | Bacteria | Verrucomicrobia | [Spartobacteria] | [Chthoniobacterales] | [Chthoniobacteraceae] | *DA101* |  |  |
| CAF | V1163 | 0.967 | 0.051 | 0.223 | 0.0242 | Bacteria | Bacteroidetes | Flavobacteriia | Flavobacteriales | [Weeksellaceae] | *Chryseobacterium* |  |  |
| CAF | V847 | 0.742 | 0.049 | 0.190 | 0.0110 | Bacteria | Proteobacteria | Alphaproteobacteria | Rhizobiales | Methylobacteriaceae | *Methylobacterium* | *adhaesivum* |  |
| CAF | V923 | 0.776 | 0.046 | 0.189 | 0.0090 | Bacteria | Proteobacteria | Deltaproteobacteria | Myxococcales | Cystobacteraceae | *Cystobacter* |  |  |
| CAF | V1220 | 1.000 | 0.044 | 0.209 | 0.0003 | Bacteria | Proteobacteria | Betaproteobacteria | Burkholderiales | Comamonadaceae | *Rhodoferax* |  |  |
| CAF | V481 | 0.944 | 0.044 | 0.203 | 0.0021 | Bacteria | Proteobacteria | Betaproteobacteria | Burkholderiales | Comamonadaceae | *Paucibacter* |  |  |
| CAF | V1670 | 1.000 | 0.041 | 0.203 | 0.0006 | Bacteria | Proteobacteria | Gammaproteobacteria | Enterobacteriales | Enterobacteriaceae | *Erwinia* |  |  |
| CAF | V640 | 0.843 | 0.039 | 0.180 | 0.0062 | Bacteria | Bacteroidetes | Cytophagia | Cytophagales | Cytophagaceae | *Hymenobacter* |  |  |
| CAF | V706 | 0.967 | 0.039 | 0.193 | 0.0178 | Bacteria | Actinobacteria | Actinobacteria | Actinomycetales | Micrococcaceae | *Kocuria* |  |  |
| CAF | V822 | 1.000 | 0.039 | 0.196 | 0.0005 | Bacteria | Proteobacteria | Alphaproteobacteria | Sphingomonadales | Sphingomonadaceae | *Sphingomonas* |  |  |
| CAF | V1394 | 1.000 | 0.036 | 0.190 | 0.0009 | Bacteria | Proteobacteria | Gammaproteobacteria | Xanthomonadales | Xanthomonadaceae | *Lysobacter* |  |  |
| CAF | V1674 | 0.852 | 0.033 | 0.169 | 0.0067 | Bacteria | Bacteroidetes | Cytophagia | Cytophagales | Cytophagaceae | *Hymenobacter* |  |  |
| CAF | V2179 | 1.000 | 0.033 | 0.183 | 0.0010 | Bacteria | Proteobacteria | Alphaproteobacteria | Rhizobiales | Hyphomicrobiaceae | *Rhodoplanes* |  |  |
| CAF | V391 | 0.849 | 0.033 | 0.168 | 0.0164 | Bacteria | Proteobacteria | Betaproteobacteria | Burkholderiales | Comamonadaceae | *Variovorax* |  |  |
| CAF | V622 | 1.000 | 0.033 | 0.183 | 0.0019 | Bacteria | Bacteroidetes | Cytophagia | Cytophagales | Cytophagaceae | *Hymenobacter* |  |  |
| CAF | V792 | 0.837 | 0.033 | 0.167 | 0.0193 | Bacteria | Firmicutes | Bacilli | Lactobacillales | Aerococcaceae | *Abiotrophia* |  |  |
| CAF | V812 | 0.843 | 0.033 | 0.168 | 0.0278 | Bacteria | Proteobacteria | Alphaproteobacteria | Rhizobiales | Methylobacteriaceae | *Methylobacterium* | *adhaesivum* |  |
| CAF | V880 | 0.944 | 0.033 | 0.178 | 0.0028 | Bacteria | Proteobacteria | Gammaproteobacteria | Thiotrichales | Piscirickettsiaceae |  |  |  |
| CAF | V1186 | 0.995 | 0.031 | 0.175 | 0.0279 | Bacteria | Firmicutes | Bacilli | Bacillales | Bacillaceae | *Bacillus* | *muralis* |  |
| CAF | V1213 | 0.971 | 0.031 | 0.173 | 0.0060 | Bacteria | Firmicutes | Bacilli | Bacillales | Planococcaceae | *Planomicrobium* |  |  |
| CAF | V1237 | 1.000 | 0.031 | 0.176 | 0.0031 | Bacteria | Actinobacteria | Actinobacteria | Actinomycetales | Dietziaceae | *Dietzia* |  |  |
| CAF | V1323 | 0.929 | 0.031 | 0.169 | 0.0114 | Bacteria | Bacteroidetes | Sphingobacteriia | Sphingobacteriales | Sphingobacteriaceae | *Sphingobacterium* |  |  |
| CAF | V1477 | 1.000 | 0.031 | 0.176 | 0.0095 | Bacteria | Proteobacteria | Gammaproteobacteria | Pseudomonadales | Pseudomonadaceae | *Pseudomonas* | *umsongensis* |  |
| CAF | V1894 | 0.939 | 0.031 | 0.170 | 0.0049 | Bacteria | Proteobacteria | Deltaproteobacteria | Myxococcales | Myxococcaceae | *Anaeromyxobacter* |  |  |
| CAF | V2195 | 1.000 | 0.031 | 0.176 | 0.0021 | Bacteria | Proteobacteria | Gammaproteobacteria | Pseudomonadales | Pseudomonadaceae | *Azorhizophilus* |  |  |
| CAF | V494 | 0.930 | 0.031 | 0.169 | 0.0125 | Bacteria | Actinobacteria | Thermoleophilia | Solirubrobacterales |  |  |  |  |
| CAF | V512 | 0.951 | 0.031 | 0.171 | 0.0107 | Bacteria | Proteobacteria | Alphaproteobacteria | Rhizobiales | Beijerinckiaceae | *Beijerinckia* |  |  |
| CAF | V566 | 1.000 | 0.031 | 0.176 | 0.0018 | Bacteria | Proteobacteria | Gammaproteobacteria | Pseudomonadales | Pseudomonadaceae |  |  |  |
| CAF | V653 | 0.835 | 0.031 | 0.161 | 0.0492 | Bacteria | Proteobacteria | Alphaproteobacteria | Rickettsiales | Rickettsiaceae |  |  |  |
| CAF | V76 | 0.962 | 0.031 | 0.172 | 0.0185 | Bacteria | Proteobacteria | Gammaproteobacteria | Xanthomonadales | Xanthomonadaceae | *Stenotrophomonas* | *acidaminiphila* |  |
| CAF | V1069 | 0.874 | 0.028 | 0.157 | 0.0348 | Bacteria | Proteobacteria | Betaproteobacteria | Burkholderiales | Comamonadaceae | *Leptothrix* |  |  |
| CAF | V1335 | 0.877 | 0.028 | 0.158 | 0.0165 | Bacteria | Proteobacteria | Gammaproteobacteria | Pseudomonadales | Moraxellaceae | *Psychrobacter* |  |  |
| CAF | V1691 | 0.901 | 0.028 | 0.160 | 0.0230 | Bacteria | Gemmatimonadetes | Gemmatimonadetes | Gemmatimonadales | A1-B1 |  |  |  |
| CAF | V1902 | 1.000 | 0.028 | 0.168 | 0.0032 | Bacteria | Firmicutes | Bacilli | Bacillales | Paenibacillaceae | *Paenibacillus* |  |  |
| CAF | V724 | 0.976 | 0.028 | 0.166 | 0.0170 | Bacteria | Firmicutes | Bacilli | Lactobacillales | Carnobacteriaceae | *Granulicatella* |  |  |
| CAF | V1098 | 1.000 | 0.026 | 0.160 | 0.0062 | Bacteria | Proteobacteria | Betaproteobacteria | Burkholderiales | Burkholderiaceae | *Burkholderia* | *andropogonis* |  |
| CAF | V1392 | 0.884 | 0.026 | 0.151 | 0.0281 | Bacteria | Proteobacteria | Gammaproteobacteria | Pasteurellales | Pasteurellaceae | *Actinobacillus* | *parahaemolyticus* |  |
| CAF | V1467 | 1.000 | 0.026 | 0.160 | 0.0090 | Bacteria | Planctomycetes | Planctomycetia | Planctomycetales | Planctomycetaceae | *Planctomyces* |  |  |
| CAF | V1473 | 0.974 | 0.026 | 0.158 | 0.0125 | Bacteria | Actinobacteria | Actinobacteria | Actinomycetales | Actinosynnemataceae |  |  |  |
| CAF | V1879 | 0.835 | 0.026 | 0.147 | 0.0256 | Bacteria | Proteobacteria | Alphaproteobacteria | Rhizobiales | Methylobacteriaceae | *Methylobacterium* | *adhaesivum* |  |
| CAF | V501 | 0.915 | 0.026 | 0.153 | 0.0188 | Bacteria | Proteobacteria | Betaproteobacteria | Burkholderiales | Comamonadaceae | *Rhodoferax* |  |  |
| CAF | V744 | 0.936 | 0.026 | 0.155 | 0.0420 | Bacteria | Fusobacteria | Fusobacteriia | Fusobacteriales | Fusobacteriaceae | *u114* |  |  |
| CAF | V832 | 1.000 | 0.026 | 0.160 | 0.0036 | Bacteria | Actinobacteria | Thermoleophilia | Solirubrobacterales | Solirubrobacteraceae |  |  |  |
| CAF | V904 | 0.836 | 0.026 | 0.147 | 0.0331 | Bacteria | Proteobacteria | Alphaproteobacteria | Rhizobiales | Hyphomicrobiaceae | *Hyphomicrobium* |  |  |
| CAF | V1084 | 1.000 | 0.023 | 0.152 | 0.0086 | Bacteria | Proteobacteria | Deltaproteobacteria | Syntrophobacterales | Syntrophobacteraceae |  |  |  |
| CAF | V1193 | 1.000 | 0.023 | 0.152 | 0.0088 | Bacteria | Bacteroidetes | [Saprospirae] | [Saprospirales] | Chitinophagaceae | *Sediminibacterium* |  |  |
| CAF | V1436 | 0.868 | 0.023 | 0.142 | 0.0252 | Bacteria | Firmicutes | Bacilli | Bacillales |  |  |  |  |
| CAF | V1523 | 1.000 | 0.023 | 0.152 | 0.0074 | Bacteria | Firmicutes | Bacilli | Lactobacillales | Lactobacillaceae |  |  |  |
| CAF | V1680 | 0.923 | 0.023 | 0.146 | 0.0396 | Bacteria | Proteobacteria | Betaproteobacteria | Burkholderiales | Comamonadaceae |  |  |  |
| CAF | V1956 | 0.868 | 0.023 | 0.142 | 0.0479 | Bacteria | Bacteroidetes | Sphingobacteriia | Sphingobacteriales | Sphingobacteriaceae | *Sphingobacterium* | *faecium* |  |
| CAF | V2178 | 0.835 | 0.023 | 0.139 | 0.0376 | Bacteria | Chloroflexi | Anaerolineae | pLW-97 |  |  |  |  |
| CAF | V909 | 0.893 | 0.023 | 0.144 | 0.0219 | Bacteria | Bacteroidetes | Cytophagia | Cytophagales | Cytophagaceae | *Hymenobacter* |  |  |
| CAF | V966 | 0.949 | 0.023 | 0.148 | 0.0284 | Bacteria | Bacteroidetes | [Saprospirae] | [Saprospirales] | Chitinophagaceae | *Sediminibacterium* |  |  |
| CAF | V1212 | 0.965 | 0.021 | 0.141 | 0.0447 | Bacteria | Proteobacteria | Alphaproteobacteria | Rhodobacterales | Rhodobacteraceae | *Rhodobacter* |  |  |
| CAF | V1295 | 1.000 | 0.021 | 0.143 | 0.0168 | Bacteria | Proteobacteria | Betaproteobacteria |  |  |  |  |  |
| CAF | V1339 | 1.000 | 0.021 | 0.143 | 0.0164 | Bacteria | Proteobacteria | Alphaproteobacteria | Rhizobiales | Methylobacteriaceae | *Methylobacterium* |  |  |
| CAF | V1635 | 0.917 | 0.021 | 0.137 | 0.0439 | Bacteria | Actinobacteria | Actinobacteria | Actinomycetales | Corynebacteriaceae | *Corynebacterium* |  |  |
| CAF | V2101 | 0.945 | 0.021 | 0.139 | 0.0268 | Bacteria | Proteobacteria | Betaproteobacteria |  |  |  |  |  |
| CAF | V2156 | 0.979 | 0.021 | 0.142 | 0.0257 | Bacteria | Proteobacteria | Gammaproteobacteria | Salinisphaerales | Salinisphaeraceae | *Salinisphaera* |  |  |
| CAF | V2181 | 1.000 | 0.021 | 0.143 | 0.0145 | Bacteria | Proteobacteria | Betaproteobacteria | SC-I-84 |  |  |  |  |
| CAF | V393 | 0.899 | 0.021 | 0.136 | 0.0412 | Bacteria | Proteobacteria | Alphaproteobacteria | Rhizobiales | Rhizobiaceae | *Agrobacterium* |  |  |
| CAF | V449 | 0.929 | 0.021 | 0.138 | 0.0304 | Bacteria | Firmicutes | Clostridia | Clostridiales | Peptococcaceae | *rc4-4* |  |  |
| CAF | V62 | 0.911 | 0.021 | 0.137 | 0.0380 | Bacteria | Proteobacteria | Betaproteobacteria | Burkholderiales | Comamonadaceae |  |  |  |
| CAF | V878 | 1.000 | 0.021 | 0.143 | 0.0180 | Bacteria | Actinobacteria | Actinobacteria | Actinomycetales | Nocardioidaceae |  |  |  |
| CAF | V902 | 1.000 | 0.021 | 0.143 | 0.0184 | Bacteria | Actinobacteria | Actinobacteria | Actinomycetales | Nocardioidaceae |  |  |  |
| CAF | V969 | 1.000 | 0.021 | 0.143 | 0.0144 | Bacteria | Proteobacteria | Alphaproteobacteria | Rhizobiales | Hyphomicrobiaceae | *Rhodoplanes* |  |  |
| CAF | V105 | 1.000 | 0.018 | 0.134 | 0.0347 | Bacteria | Proteobacteria | Alphaproteobacteria | Rhodospirillales | Rhodospirillaceae | *Magnetospirillum* |  |  |
| CAF | V1519 | 1.000 | 0.018 | 0.134 | 0.0237 | Bacteria | Proteobacteria | Alphaproteobacteria | Rhodospirillales | Acetobacteraceae |  |  |  |
| CAF | V1693 | 1.000 | 0.018 | 0.134 | 0.0209 | Bacteria | Acidobacteria | Acidobacteriia | Acidobacteriales | Koribacteraceae | *Candidatus Koribacter* |  |  |
| CAF | V1704 | 1.000 | 0.018 | 0.134 | 0.0251 | Bacteria | Bacteroidetes | Cytophagia | Cytophagales | Cytophagaceae | *Hymenobacter* |  |  |
| CAF | V2186 | 1.000 | 0.018 | 0.134 | 0.0258 | Bacteria | Chloroflexi | Anaerolineae | pLW-97 |  |  |  |  |
| CAF | V2197 | 1.000 | 0.018 | 0.134 | 0.0299 | Bacteria | Proteobacteria | Alphaproteobacteria | Rhodospirillales | Rhodospirillaceae |  |  |  |
| CAF | V2254 | 1.000 | 0.018 | 0.134 | 0.0305 | Bacteria | Firmicutes | Bacilli | Lactobacillales | Streptococcaceae | *Lactococcus* | *garvieae* |  |
| CAF | V587 | 1.000 | 0.018 | 0.134 | 0.0247 | Bacteria | Proteobacteria | Betaproteobacteria |  |  |  |  |  |
| CAF | V827 | 0.926 | 0.018 | 0.129 | 0.0467 | Bacteria | Proteobacteria | Gammaproteobacteria | Xanthomonadales | Xanthomonadaceae | *Lysobacter* |  |  |
| CAF | V841 | 0.905 | 0.018 | 0.128 | 0.0452 | Bacteria | Verrucomicrobia | [Spartobacteria] | [Chthoniobacterales] | [Chthoniobacteraceae] | *DA101* |  |  |
| CAF | V958 | 1.000 | 0.018 | 0.134 | 0.0212 | Bacteria | Verrucomicrobia | [Spartobacteria] | [Chthoniobacterales] | [Chthoniobacteraceae] |  |  |  |
| CAF | V1001 | 1.000 | 0.015 | 0.124 | 0.0416 | Bacteria | Acidobacteria | [Chloracidobacteria] | RB41 | Ellin6075 |  |  |  |
| CAF | V1157 | 1.000 | 0.015 | 0.124 | 0.0410 | Bacteria | Bacteroidetes | Flavobacteriia | Flavobacteriales | [Weeksellaceae] | *Chryseobacterium* |  |  |
| CAF | V118 | 1.000 | 0.015 | 0.124 | 0.0438 | Bacteria | Actinobacteria | Actinobacteria | Actinomycetales | Cellulomonadaceae | *Cellulomonas* | *xylanilytica* |  |
| CAF | V1225 | 1.000 | 0.015 | 0.124 | 0.0410 | Bacteria | Proteobacteria | Alphaproteobacteria | Sphingomonadales | Erythrobacteraceae | *Erythromicrobium* |  |  |
| CAF | V1247 | 1.000 | 0.015 | 0.124 | 0.0400 | Bacteria | Proteobacteria | Gammaproteobacteria | Pasteurellales | Pasteurellaceae | *Aggregatibacter* |  |  |
| CAF | V1282 | 1.000 | 0.015 | 0.124 | 0.0430 | Bacteria | Actinobacteria | Actinobacteria | Actinomycetales | Microbacteriaceae |  |  |  |
| CAF | V1443 | 1.000 | 0.015 | 0.124 | 0.0487 | Bacteria | Actinobacteria | Actinobacteria | Actinomycetales | Dermacoccaceae | *Dermacoccus* |  |  |
| CAF | V1564 | 1.000 | 0.015 | 0.124 | 0.0427 | Bacteria | Actinobacteria | Thermoleophilia | Gaiellales | Gaiellaceae |  |  |  |
| CAF | V1584 | 1.000 | 0.015 | 0.124 | 0.0388 | Bacteria | Bacteroidetes | [Saprospirae] | [Saprospirales] | Chitinophagaceae |  |  |  |
| CAF | V1675 | 1.000 | 0.015 | 0.124 | 0.0478 | Bacteria | Bacteroidetes | [Saprospirae] | [Saprospirales] | Chitinophagaceae | *Sediminibacterium* |  |  |
| CAF | V1705 | 1.000 | 0.015 | 0.124 | 0.0396 | Bacteria | Proteobacteria | Betaproteobacteria | Rhodocyclales | Rhodocyclaceae | *Petrobacter* | *succinatimandens* |  |
| CAF | V1724 | 1.000 | 0.015 | 0.124 | 0.0442 | Bacteria | NC10 | Dez 24 | JH-WHS47 |  |  |  |  |
| CAF | V1777 | 1.000 | 0.015 | 0.124 | 0.0368 | Bacteria | Proteobacteria | Betaproteobacteria | Burkholderiales | Comamonadaceae |  |  |  |
| CAF | V1784 | 1.000 | 0.015 | 0.124 | 0.0401 | Bacteria | Bacteroidetes | [Saprospirae] | [Saprospirales] | Chitinophagaceae |  |  |  |
| CAF | V1817 | 1.000 | 0.015 | 0.124 | 0.0488 | Bacteria | Bacteroidetes |  |  |  |  |  |  |
| CAF | V1903 | 1.000 | 0.015 | 0.124 | 0.0417 | Bacteria | Actinobacteria | Actinobacteria | Actinomycetales | Microbacteriaceae | *Mycetocola* |  |  |
| CAF | V2183 | 1.000 | 0.015 | 0.124 | 0.0388 | Bacteria | Proteobacteria | Deltaproteobacteria | Myxococcales | Myxococcaceae | *Anaeromyxobacter* |  |  |
| CAF | V2190 | 1.000 | 0.015 | 0.124 | 0.0368 | Bacteria | Proteobacteria | Alphaproteobacteria | Rhodospirillales | Rhodospirillaceae |  |  |  |
| CAF | V2233 | 1.000 | 0.015 | 0.124 | 0.0477 | Bacteria |  |  |  |  |  |  |  |
| CAF | V2309 | 1.000 | 0.015 | 0.124 | 0.0436 | Bacteria | Actinobacteria | Rubrobacteria | Rubrobacterales | Rubrobacteraceae | *Rubrobacter* |  |  |
| CAF | V475 | 1.000 | 0.015 | 0.124 | 0.0398 | Bacteria | Proteobacteria | Alphaproteobacteria | Sphingomonadales | Sphingomonadaceae | *Kaistobacter* |  |  |
| CAF | V607 | 1.000 | 0.015 | 0.124 | 0.0483 | Bacteria | Firmicutes | Bacilli | Bacillales | Bacillaceae | *Geobacillus* |  |  |
| CAF | V666 | 1.000 | 0.015 | 0.124 | 0.0371 | Bacteria | Proteobacteria | Betaproteobacteria | Neisseriales | Neisseriaceae | *Chitinibacter* | *tainanensis* |  |
| CAF | V723 | 1.000 | 0.015 | 0.124 | 0.0400 | Bacteria | Bacteroidetes | Cytophagia | Cytophagales | Cytophagaceae | *Hymenobacter* |  |  |
| CAF | V892 | 1.000 | 0.015 | 0.124 | 0.0430 | Bacteria | Actinobacteria | Actinobacteria | Actinomycetales | Geodermatophilaceae |  |  |  |
| SAF | V127 | 0.986 | 0.369 | 0.603 | 0.0001 | Bacteria | Proteobacteria | Gammaproteobacteria | Oceanospirillales | Halomonadaceae | *Halomonas* |  |  |
| SAF | V240 | 0.983 | 0.364 | 0.598 | 0.0001 | Bacteria | Firmicutes | Erysipelotrichi | Erysipelotrichales | Erysipelotrichaceae | *[Eubacterium]* | *dolichum* |  |
| SAF | V160 | 0.983 | 0.359 | 0.594 | 0.0001 | Bacteria | Proteobacteria | Gammaproteobacteria | Oceanospirillales | Halomonadaceae | *Halomonas* |  |  |
| SAF | V711 | 0.643 | 0.348 | 0.473 | 0.0001 | Bacteria | Proteobacteria | Gammaproteobacteria | Pseudomonadales | Pseudomonadaceae | *Pseudomonas* | *veronii* |  |
| SAF | V184 | 0.941 | 0.298 | 0.530 | 0.0001 | Bacteria | Actinobacteria | Actinobacteria | Actinomycetales | Corynebacteriaceae | *Corynebacterium* |  |  |
| SAF | V183 | 0.986 | 0.283 | 0.528 | 0.0001 | Bacteria | Proteobacteria | Gammaproteobacteria | Vibrionales | Vibrionaceae | *Vibrio* | *rumoiensis* |  |
| SAF | V20 | 0.866 | 0.268 | 0.481 | 0.0001 | Bacteria | Actinobacteria | Actinobacteria | Actinomycetales | Corynebacteriaceae | *Corynebacterium* |  |  |
| SAF | V1555 | 0.976 | 0.268 | 0.511 | 0.0001 | Bacteria | Firmicutes | Bacilli | Bacillales | Listeriaceae | *Brochothrix* |  |  |
| SAF | V98 | 0.818 | 0.207 | 0.411 | 0.0028 | Bacteria | Proteobacteria | Gammaproteobacteria | Enterobacteriales | Enterobacteriaceae | *Escherichia* | *coli* |  |
| SAF | V176 | 0.897 | 0.187 | 0.410 | 0.0001 | Bacteria | Actinobacteria | Actinobacteria | Actinomycetales | Pseudonocardiaceae | *Actinomycetospora* |  |  |
| SAF | V129 | 0.877 | 0.162 | 0.376 | 0.0001 | Bacteria | Proteobacteria | Alphaproteobacteria | Rhizobiales | Brucellaceae | *Ochrobactrum* |  |  |
| SAF | V287 | 0.950 | 0.162 | 0.392 | 0.0001 | Bacteria | Proteobacteria | Betaproteobacteria | Burkholderiales | Alcaligenaceae | *Achromobacter* |  |  |
| SAF | V630 | 0.819 | 0.162 | 0.364 | 0.0001 | Bacteria | Actinobacteria | Actinobacteria | Actinomycetales | Corynebacteriaceae | *Corynebacterium* |  |  |
| SAF | V1494 | 0.987 | 0.152 | 0.387 | 0.0001 | Bacteria | Proteobacteria | Betaproteobacteria | Burkholderiales | Burkholderiaceae | *Burkholderia* |  |  |
| SAF | V605 | 0.865 | 0.146 | 0.356 | 0.0001 | Bacteria | Proteobacteria | Alphaproteobacteria | Sphingomonadales | Sphingomonadaceae | *Sphingomonas* |  |  |
| SAF | V23 | 0.492 | 0.126 | 0.249 | 0.0496 | Bacteria | Proteobacteria | Gammaproteobacteria | Pseudomonadales | Pseudomonadaceae | *Pseudomonas* | *pseudoalcaligenes* |  |
| SAF | V318 | 0.755 | 0.126 | 0.309 | 0.0001 | Bacteria | Proteobacteria | Betaproteobacteria | Neisseriales | Neisseriaceae |  |  |  |
| SAF | V634 | 0.677 | 0.126 | 0.292 | 0.0001 | Bacteria | Firmicutes | Bacilli | Lactobacillales | Streptococcaceae | *Streptococcus* |  |  |
| SAF | V845 | 0.873 | 0.126 | 0.332 | 0.0001 | Bacteria | Actinobacteria | Actinobacteria | Actinomycetales | Microbacteriaceae | *Yonghaparkia* |  |  |
| SAF | V115 | 0.829 | 0.121 | 0.317 | 0.0006 | Bacteria | Proteobacteria | Alphaproteobacteria | Rhizobiales | Methylobacteriaceae |  |  |  |
| SAF | V238 | 0.704 | 0.111 | 0.280 | 0.0003 | Bacteria | Bacteroidetes | Flavobacteriia | Flavobacteriales | [Weeksellaceae] | *Elizabethkingia* | *meningoseptica* |  |
| SAF | V1358 | 0.961 | 0.111 | 0.327 | 0.0001 | Bacteria | Actinobacteria | Actinobacteria | Actinomycetales | Corynebacteriaceae | *Corynebacterium* |  |  |
| SAF | V14 | 0.587 | 0.106 | 0.249 | 0.0024 | Bacteria | Actinobacteria | Actinobacteria | Actinomycetales | Propionibacteriaceae | *Propionibacterium* | *acnes* |  |
| SAF | V483 | 0.979 | 0.106 | 0.322 | 0.0001 | Bacteria | Firmicutes | Bacilli | Lactobacillales | Aerococcaceae | *Alloiococcus* |  |  |
| SAF | V1144 | 0.636 | 0.101 | 0.253 | 0.0015 | Bacteria | Proteobacteria | Alphaproteobacteria | Rhizobiales | Methylobacteriaceae | *Methylobacterium* | *organophilum* |  |
| SAF | V1115 | 0.815 | 0.096 | 0.280 | 0.0001 | Bacteria | FBP |  |  |  |  |  |  |
| SAF | V349 | 0.814 | 0.091 | 0.272 | 0.0001 | Bacteria | Proteobacteria | Gammaproteobacteria | Pseudomonadales | Moraxellaceae | *Acinetobacter* |  |  |
| SAF | V569 | 0.875 | 0.091 | 0.282 | 0.0001 | Bacteria | Verrucomicrobia | [Spartobacteria] | [Chthoniobacterales] | [Chthoniobacteraceae] |  |  |  |
| SAF | V61 | 0.663 | 0.091 | 0.246 | 0.0390 | Bacteria | Proteobacteria | Betaproteobacteria | Burkholderiales | Comamonadaceae | *Comamonas* |  |  |
| SAF | V713 | 0.896 | 0.091 | 0.285 | 0.0001 | Bacteria | Actinobacteria | Actinobacteria | Actinomycetales | Corynebacteriaceae | *Corynebacterium* |  |  |
| SAF | V730 | 0.747 | 0.091 | 0.261 | 0.0009 | Bacteria | Proteobacteria | Alphaproteobacteria | Rhizobiales | Methylobacteriaceae |  |  |  |
| SAF | V886 | 0.901 | 0.086 | 0.278 | 0.0001 | Bacteria | Proteobacteria | Gammaproteobacteria | Pseudomonadales | Pseudomonadaceae | *Pseudomonas* | *stutzeri* |  |
| SAF | V1124 | 0.857 | 0.086 | 0.271 | 0.0001 | Bacteria | Acidobacteria | Acidobacteriia | Acidobacteriales | Acidobacteriaceae | *Terriglobus* |  |  |
| SAF | V557 | 0.841 | 0.081 | 0.261 | 0.0003 | Bacteria | Proteobacteria | Alphaproteobacteria | Rhizobiales | Methylocystaceae |  |  |  |
| SAF | V1133 | 0.961 | 0.076 | 0.270 | 0.0001 | Bacteria | Proteobacteria | Alphaproteobacteria | Caulobacterales | Caulobacteraceae |  |  |  |
| SAF | V450 | 0.979 | 0.076 | 0.272 | 0.0001 | Bacteria | Proteobacteria | Alphaproteobacteria | Rhizobiales | Brucellaceae |  |  |  |
| SAF | V534 | 0.937 | 0.076 | 0.266 | 0.0003 | Bacteria | Actinobacteria | Actinobacteria | Actinomycetales | Pseudonocardiaceae | *Pseudonocardia* |  |  |
| SAF | V263 | 0.968 | 0.076 | 0.271 | 0.0001 | Bacteria | Actinobacteria | Actinobacteria | Actinomycetales | Dietziaceae |  |  |  |
| SAF | V1159 | 0.861 | 0.071 | 0.247 | 0.0001 | Bacteria | Acidobacteria | Acidobacteriia | Acidobacteriales | Acidobacteriaceae | *Granulicella* | *paludicola* |  |
| SAF | V2130 | 1.000 | 0.071 | 0.266 | 0.0001 | Bacteria | Actinobacteria | Actinobacteria | Actinomycetales | Corynebacteriaceae | *Corynebacterium* |  |  |
| SAF | V260 | 0.805 | 0.071 | 0.239 | 0.0001 | Bacteria | Actinobacteria | Actinobacteria | Actinomycetales | Promicromonosporaceae | *Cellulosimicrobium* |  |  |
| SAF | V592 | 0.791 | 0.071 | 0.236 | 0.0002 | Bacteria | Firmicutes | Clostridia | Clostridiales | [Tissierellaceae] | *Anaerococcus* |  |  |
| SAF | V1146 | 0.683 | 0.066 | 0.212 | 0.0011 | Bacteria | Actinobacteria | Actinobacteria | Actinomycetales | Frankiaceae |  |  |  |
| SAF | V1347 | 0.928 | 0.066 | 0.247 | 0.0002 | Bacteria | Proteobacteria | Alphaproteobacteria | Rhizobiales | Methylocystaceae |  |  |  |
| SAF | V1390 | 0.998 | 0.066 | 0.256 | 0.0001 | Bacteria | Actinobacteria | Actinobacteria | Bifidobacteriales | Bifidobacteriaceae |  |  |  |
| SAF | V1573 | 0.786 | 0.066 | 0.227 | 0.0001 | Bacteria | Proteobacteria | Alphaproteobacteria | Rhizobiales | Beijerinckiaceae | *Beijerinckia* |  |  |
| SAF | V256 | 0.909 | 0.066 | 0.244 | 0.0001 | Bacteria | Proteobacteria | Gammaproteobacteria | Xanthomonadales | Xanthomonadaceae | *Pseudoxanthomonas* |  |  |
| SAF | V487 | 0.955 | 0.066 | 0.250 | 0.0001 | Bacteria | Proteobacteria | Betaproteobacteria | Burkholderiales | Comamonadaceae | *Rubrivivax* |  |  |
| SAF | V637 | 0.968 | 0.066 | 0.252 | 0.0001 | Bacteria | Proteobacteria | Betaproteobacteria | Burkholderiales | Burkholderiaceae | *Pandoraea* |  |  |
| SAF | V1005 | 0.721 | 0.061 | 0.209 | 0.0017 | Bacteria | Actinobacteria | Actinobacteria | Actinomycetales | Micrococcaceae | *Rothia* | *mucilaginosa* |  |
| SAF | V1060 | 0.940 | 0.061 | 0.239 | 0.0002 | Bacteria | Verrucomicrobia | [Spartobacteria] | [Chthoniobacterales] | [Chthoniobacteraceae] |  |  |  |
| SAF | V1185 | 0.890 | 0.061 | 0.232 | 0.0006 | Bacteria | Firmicutes | Clostridia | Clostridiales | Veillonellaceae | *Veillonella* | *dispar* |  |
| SAF | V1381 | 0.851 | 0.061 | 0.227 | 0.0002 | Bacteria | Actinobacteria | Actinobacteria | Actinomycetales | Corynebacteriaceae | *Corynebacterium* |  |  |
| SAF | V2466 | 0.949 | 0.061 | 0.240 | 0.0001 | Bacteria | Proteobacteria | Betaproteobacteria | Burkholderiales | Alcaligenaceae |  |  |  |
| SAF | V257 | 0.552 | 0.061 | 0.183 | 0.0059 | Bacteria | Actinobacteria | Actinobacteria | Actinomycetales | Nocardiaceae | *Rhodococcus* | *equi* |  |
| SAF | V2648 | 1.000 | 0.061 | 0.246 | 0.0001 | Bacteria | Actinobacteria | Actinobacteria | Actinomycetales | Yaniellaceae | *Auritibacter* | *ignavus* |  |
| SAF | V530 | 0.844 | 0.061 | 0.226 | 0.0004 | Bacteria | Proteobacteria | Alphaproteobacteria | Sphingomonadales | Sphingomonadaceae | *Sphingomonas* |  |  |
| SAF | V584 | 0.899 | 0.061 | 0.233 | 0.0001 | Bacteria | Firmicutes | Bacilli | Lactobacillales | Lactobacillaceae | *Lactobacillus* | *salivarius* |  |
| SAF | V786 | 0.916 | 0.061 | 0.236 | 0.0006 | Bacteria | Proteobacteria | Deltaproteobacteria | Myxococcales | Polyangiaceae | *Byssovorax* | *cruenta* |  |
| SAF | V577 | 0.858 | 0.061 | 0.228 | 0.0001 | Bacteria | Proteobacteria | Alphaproteobacteria | Caulobacterales | Caulobacteraceae |  |  |  |
| SAF | V1028 | 0.901 | 0.056 | 0.224 | 0.0004 | Bacteria | Proteobacteria | Alphaproteobacteria | Caulobacterales | Caulobacteraceae |  |  |  |
| SAF | V1035 | 0.908 | 0.056 | 0.225 | 0.0001 | Bacteria | Proteobacteria | Alphaproteobacteria | Rhizobiales | Methylocystaceae |  |  |  |
| SAF | V1055 | 0.869 | 0.056 | 0.220 | 0.0001 | Bacteria | Proteobacteria | Alphaproteobacteria | Rhizobiales | Beijerinckiaceae | *Beijerinckia* |  |  |
| SAF | V1320 | 0.810 | 0.056 | 0.212 | 0.0009 | Bacteria | Proteobacteria | Alphaproteobacteria | Sphingomonadales | Sphingomonadaceae | *Sphingomonas* |  |  |
| SAF | V511 | 0.773 | 0.056 | 0.207 | 0.0013 | Bacteria | Proteobacteria | Betaproteobacteria | Rhodocyclales | Rhodocyclaceae | *Zoogloea* |  |  |
| SAF | V524 | 0.993 | 0.056 | 0.235 | 0.0002 | Bacteria | Firmicutes | Clostridia | Clostridiales | Veillonellaceae | *Veillonella* |  |  |
| SAF | V612 | 0.807 | 0.056 | 0.212 | 0.0016 | Bacteria | Firmicutes | Clostridia | Clostridiales | [Tissierellaceae] | *Finegoldia* |  |  |
| SAF | V621 | 0.830 | 0.056 | 0.215 | 0.0134 | Bacteria | Actinobacteria | Actinobacteria | Actinomycetales | Corynebacteriaceae | *Corynebacterium* | *variabile* |  |
| SAF | V1013 | 0.917 | 0.051 | 0.215 | 0.0003 | Bacteria | Proteobacteria | Alphaproteobacteria | Rhizobiales | Xanthobacteraceae | *Labrys* |  |  |
| SAF | V2140 | 0.996 | 0.051 | 0.224 | 0.0001 | Bacteria | Proteobacteria | Alphaproteobacteria | Rhizobiales | Beijerinckiaceae | *Beijerinckia* |  |  |
| SAF | V2740 | 1.000 | 0.051 | 0.225 | 0.0001 | Bacteria |  |  |  |  |  |  |  |
| SAF | V292 | 0.997 | 0.051 | 0.224 | 0.0001 | Bacteria | Firmicutes | Bacilli | Bacillales | Bacillaceae | *Bacillus* | *clausii* |  |
| SAF | V376 | 0.939 | 0.051 | 0.218 | 0.0005 | Bacteria | Actinobacteria | Actinobacteria | Actinomycetales | Corynebacteriaceae | *Corynebacterium* |  |  |
| SAF | V708 | 0.969 | 0.051 | 0.221 | 0.0053 | Bacteria | Proteobacteria | Gammaproteobacteria | Pseudomonadales | Pseudomonadaceae | *Pseudomonas* |  |  |
| SAF | V1152 | 0.901 | 0.045 | 0.202 | 0.0002 | Bacteria | Proteobacteria | Alphaproteobacteria | Sphingomonadales | Sphingomonadaceae | *Sphingomonas* |  |  |
| SAF | V1285 | 0.860 | 0.045 | 0.198 | 0.0001 | Bacteria | Proteobacteria | Alphaproteobacteria | Rhizobiales |  |  |  |  |
| SAF | V1976 | 0.991 | 0.045 | 0.212 | 0.0003 | Bacteria | Proteobacteria | Gammaproteobacteria | Pseudomonadales | Pseudomonadaceae | *Pseudomonas* |  |  |
| SAF | V614 | 0.761 | 0.045 | 0.186 | 0.0026 | Bacteria | Actinobacteria | Actinobacteria | Actinomycetales | Corynebacteriaceae | *Corynebacterium* |  |  |
| SAF | V1039 | 0.978 | 0.040 | 0.199 | 0.0003 | Bacteria | Proteobacteria | Alphaproteobacteria | Rhizobiales | Beijerinckiaceae | *Beijerinckia* |  |  |
| SAF | V106 | 0.876 | 0.040 | 0.188 | 0.0014 | Bacteria | Proteobacteria | Alphaproteobacteria | Rhizobiales | Rhizobiaceae |  |  |  |
| SAF | V1096 | 0.977 | 0.040 | 0.199 | 0.0001 | Bacteria | Proteobacteria | Alphaproteobacteria | Rhizobiales | Methylocystaceae |  |  |  |
| SAF | V1134 | 0.949 | 0.040 | 0.196 | 0.0001 | Bacteria | Acidobacteria | Acidobacteriia | Acidobacteriales | Acidobacteriaceae |  |  |  |
| SAF | V1143 | 0.935 | 0.040 | 0.194 | 0.0002 | Bacteria | Armatimonadetes | [Fimbriimonadia] | [Fimbriimonadales] | [Fimbriimonadaceae] | *Fimbriimonas* |  |  |
| SAF | V1388 | 0.873 | 0.040 | 0.188 | 0.0067 | Bacteria | Proteobacteria | Alphaproteobacteria | Rhizobiales | Aurantimonadaceae |  |  |  |
| SAF | V1398 | 0.791 | 0.040 | 0.179 | 0.0035 | Bacteria | Actinobacteria | Actinobacteria | Actinomycetales |  |  |  |  |
| SAF | V1421 | 0.753 | 0.040 | 0.174 | 0.0091 | Bacteria | Proteobacteria | Gammaproteobacteria | Xanthomonadales | Xanthomonadaceae |  |  |  |
| SAF | V2057 | 1.000 | 0.040 | 0.201 | 0.0001 | Bacteria | Bacteroidetes | Flavobacteriia | Flavobacteriales | [Weeksellaceae] | *Chryseobacterium* |  |  |
| SAF | V456 | 0.868 | 0.040 | 0.187 | 0.0033 | Bacteria | Firmicutes | Clostridia | Clostridiales | Peptostreptococcaceae | *Clostridium* | *ruminantium* |  |
| SAF | V551 | 0.765 | 0.040 | 0.176 | 0.0467 | Bacteria | Proteobacteria | Betaproteobacteria | Burkholderiales | Burkholderiaceae | *Burkholderia* |  |  |
| SAF | V555 | 0.806 | 0.040 | 0.181 | 0.0142 | Bacteria | Actinobacteria | Actinobacteria | Actinomycetales |  |  |  |  |
| SAF | V609 | 0.835 | 0.040 | 0.184 | 0.0028 | Bacteria | Proteobacteria | Alphaproteobacteria | Rhizobiales | Xanthobacteraceae | *Labrys* |  |  |
| SAF | V967 | 0.857 | 0.040 | 0.186 | 0.0018 | Bacteria | Actinobacteria | Thermoleophilia | Solirubrobacterales | Patulibacteraceae |  |  |  |
| SAF | V1049 | 0.876 | 0.035 | 0.176 | 0.0116 | Bacteria | Firmicutes | Clostridia | Clostridiales | [Tissierellaceae] | *Anaerococcus* |  |  |
| SAF | V1257 | 0.887 | 0.035 | 0.177 | 0.0064 | Bacteria | Proteobacteria | Gammaproteobacteria | Xanthomonadales | Xanthomonadaceae | *Luteimonas* |  |  |
| SAF | V1330 | 0.776 | 0.035 | 0.166 | 0.0200 | Bacteria | Actinobacteria | Actinobacteria | Actinomycetales | Pseudonocardiaceae | *Actinomycetospora* |  |  |
| SAF | V1336 | 0.854 | 0.035 | 0.174 | 0.0042 | Bacteria | Proteobacteria | Betaproteobacteria | Rhodocyclales | Rhodocyclaceae | *C39* |  |  |
| SAF | V1461 | 0.906 | 0.035 | 0.179 | 0.0004 | Bacteria | Actinobacteria | Actinobacteria | Actinomycetales | Pseudonocardiaceae | *Pseudonocardia* |  |  |
| SAF | V1578 | 0.781 | 0.035 | 0.166 | 0.0098 | Bacteria | Acidobacteria | Acidobacteriia | Acidobacteriales | Acidobacteriaceae |  |  |  |
| SAF | V1928 | 0.832 | 0.035 | 0.172 | 0.0041 | Bacteria | Proteobacteria | Alphaproteobacteria | Rhizobiales |  |  |  |  |
| SAF | V1949 | 0.666 | 0.035 | 0.153 | 0.0130 | Bacteria | Proteobacteria | Alphaproteobacteria | Rhizobiales |  |  |  |  |
| SAF | V1989 | 0.696 | 0.035 | 0.157 | 0.0066 | Bacteria | Proteobacteria | Alphaproteobacteria | Caulobacterales | Caulobacteraceae |  |  |  |
| SAF | V2007 | 0.994 | 0.035 | 0.187 | 0.0021 | Bacteria | Firmicutes | Bacilli | Bacillales | Paenibacillaceae | *Paenibacillus* |  |  |
| SAF | V2061 | 0.944 | 0.035 | 0.183 | 0.0003 | Bacteria | Actinobacteria | Actinobacteria | Actinomycetales | Nocardioidaceae | *Nocardioides* |  |  |
| SAF | V2125 | 0.648 | 0.035 | 0.151 | 0.0083 | Bacteria | Actinobacteria | Actinobacteria | Actinomycetales | Micromonosporaceae |  |  |  |
| SAF | V2325 | 0.987 | 0.035 | 0.187 | 0.0003 | Bacteria | Proteobacteria | Gammaproteobacteria | Pseudomonadales | Pseudomonadaceae | *Pseudomonas* |  |  |
| SAF | V2352 | 0.728 | 0.035 | 0.160 | 0.0042 | Bacteria | Firmicutes | Bacilli | Lactobacillales | Aerococcaceae | *Alloiococcus* | *otitis* |  |
| SAF | V2673 | 1.000 | 0.035 | 0.188 | 0.0001 | Bacteria | Proteobacteria | Alphaproteobacteria |  |  |  |  |  |
| SAF | V554 | 0.857 | 0.035 | 0.174 | 0.0435 | Bacteria | Actinobacteria | Thermoleophilia | Solirubrobacterales | Conexibacteraceae |  |  |  |
| SAF | V87 | 0.868 | 0.035 | 0.175 | 0.0019 | Bacteria | Actinobacteria | Actinobacteria | Actinomycetales | Nocardioidaceae | *Nocardioides* |  |  |
| SAF | V1189 | 0.873 | 0.030 | 0.163 | 0.0022 | Bacteria | Actinobacteria | Actinobacteria | Actinomycetales | Micrococcaceae | *Arthrobacter* | *psychrolactophilus* |  |
| SAF | V1214 | 0.959 | 0.030 | 0.170 | 0.0011 | Bacteria | Proteobacteria | Alphaproteobacteria | Rhizobiales | Hyphomicrobiaceae | *Devosia* |  |  |
| SAF | V1287 | 0.878 | 0.030 | 0.163 | 0.0040 | Bacteria | Proteobacteria | Alphaproteobacteria | Sphingomonadales | Sphingomonadaceae | *Sphingomonas* |  |  |
| SAF | V1393 | 0.969 | 0.030 | 0.171 | 0.0005 | Bacteria | Bacteroidetes | [Saprospirae] | [Saprospirales] | Chitinophagaceae |  |  |  |
| SAF | V1475 | 0.977 | 0.030 | 0.172 | 0.0043 | Bacteria | Proteobacteria | Betaproteobacteria | Burkholderiales | Comamonadaceae |  |  |  |
| SAF | V1534 | 0.951 | 0.030 | 0.170 | 0.0008 | Bacteria | Proteobacteria | Alphaproteobacteria | Sphingomonadales | Sphingomonadaceae |  |  |  |
| SAF | V1559 | 0.969 | 0.030 | 0.171 | 0.0017 | Bacteria | Proteobacteria | Alphaproteobacteria | Rhodospirillales | Acetobacteraceae | *Roseomonas* | *mucosa* |  |
| SAF | V1567 | 0.775 | 0.030 | 0.153 | 0.0138 | Bacteria | Proteobacteria | Alphaproteobacteria | Rhizobiales | Phyllobacteriaceae | *Mesorhizobium* |  |  |
| SAF | V1625 | 0.949 | 0.030 | 0.170 | 0.0022 | Bacteria | Actinobacteria | Actinobacteria | Actinomycetales | Kineosporiaceae |  |  |  |
| SAF | V1630 | 0.908 | 0.030 | 0.166 | 0.0018 | Bacteria | Firmicutes | Erysipelotrichi | Erysipelotrichales | Erysipelotrichaceae | *[Eubacterium]* |  |  |
| SAF | V1647 | 1.000 | 0.030 | 0.174 | 0.0004 | Bacteria | Bacteroidetes | Sphingobacteriia | Sphingobacteriales | Sphingobacteriaceae | *Sphingobacterium* |  |  |
| SAF | V1933 | 0.970 | 0.030 | 0.171 | 0.0007 | Bacteria | Actinobacteria | Thermoleophilia | Solirubrobacterales | Patulibacteraceae |  |  |  |
| SAF | V2174 | 1.000 | 0.030 | 0.174 | 0.0002 | Bacteria | Bacteroidetes | [Saprospirae] | [Saprospirales] | Chitinophagaceae | *Niabella* |  |  |
| SAF | V2645 | 1.000 | 0.030 | 0.174 | 0.0004 | Bacteria | Proteobacteria | Gammaproteobacteria | Vibrionales | Vibrionaceae | *Vibrio* | *rumoiensis* |  |
| SAF | V2659 | 1.000 | 0.030 | 0.174 | 0.0003 | Bacteria | Proteobacteria | Alphaproteobacteria | Rhizobiales | Beijerinckiaceae | *Beijerinckia* |  |  |
| SAF | V350 | 0.995 | 0.030 | 0.174 | 0.0082 | Bacteria | Proteobacteria | Betaproteobacteria | Burkholderiales | Burkholderiaceae | *Burkholderia* |  |  |
| SAF | V525 | 0.736 | 0.030 | 0.149 | 0.0380 | Bacteria | Bacteroidetes | Bacteroidia | Bacteroidales | Bacteroidaceae | *Bacteroides* |  |  |
| SAF | V538 | 0.865 | 0.030 | 0.162 | 0.0380 | Bacteria | Actinobacteria | Actinobacteria | Actinomycetales | Microbacteriaceae | *Cryocola* |  |  |
| SAF | V590 | 0.952 | 0.030 | 0.170 | 0.0026 | Bacteria | Actinobacteria | Actinobacteria | Actinomycetales | Pseudonocardiaceae | *Pseudonocardia* |  |  |
| SAF | V938 | 0.914 | 0.030 | 0.166 | 0.0011 | Bacteria | Proteobacteria | Alphaproteobacteria | Sphingomonadales | Sphingomonadaceae | *Sphingomonas* |  |  |
| SAF | V1111 | 1.000 | 0.025 | 0.159 | 0.0016 | Bacteria | Bacteroidetes | Cytophagia | Cytophagales | Cytophagaceae | *Spirosoma* |  |  |
| SAF | V1136 | 1.000 | 0.025 | 0.159 | 0.0009 | Bacteria | Proteobacteria | Betaproteobacteria | Burkholderiales | Alcaligenaceae | *Alcaligenes* | *faecalis* |  |
| SAF | V1251 | 0.783 | 0.025 | 0.141 | 0.0455 | Bacteria | Proteobacteria | Alphaproteobacteria | Sphingomonadales | Sphingomonadaceae | *Sphingomonas* |  |  |
| SAF | V1288 | 1.000 | 0.025 | 0.159 | 0.0008 | Bacteria | Proteobacteria | Alphaproteobacteria | Rhodospirillales | Acetobacteraceae |  |  |  |
| SAF | V1386 | 0.783 | 0.025 | 0.141 | 0.0222 | Bacteria | Proteobacteria | Alphaproteobacteria | Sphingomonadales | Sphingomonadaceae | *Sphingomonas* |  |  |
| SAF | V1395 | 1.000 | 0.025 | 0.159 | 0.0006 | Bacteria | Actinobacteria | Actinobacteria | Actinomycetales |  |  |  |  |
| SAF | V1401 | 0.739 | 0.025 | 0.137 | 0.0347 | Bacteria | Proteobacteria | Alphaproteobacteria | Rhodospirillales | Rhodospirillaceae |  |  |  |
| SAF | V1407 | 0.878 | 0.025 | 0.149 | 0.0063 | Bacteria | Proteobacteria | Alphaproteobacteria | Rhodospirillales | Acetobacteraceae |  |  |  |
| SAF | V1457 | 0.962 | 0.025 | 0.156 | 0.0192 | Bacteria | Proteobacteria | Alphaproteobacteria | Rhodobacterales | Rhodobacteraceae | *Rhodobacter* |  |  |
| SAF | V1484 | 0.901 | 0.025 | 0.151 | 0.0118 | Bacteria | Actinobacteria | Thermoleophilia | Solirubrobacterales |  |  |  |  |
| SAF | V1612 | 0.936 | 0.025 | 0.154 | 0.0028 | Bacteria | Actinobacteria | Actinobacteria | Actinomycetales | Frankiaceae |  |  |  |
| SAF | V1804 | 1.000 | 0.025 | 0.159 | 0.0006 | Bacteria | Actinobacteria | Thermoleophilia | Solirubrobacterales | Conexibacteraceae | *Conexibacter* |  |  |
| SAF | V190 | 0.919 | 0.025 | 0.152 | 0.0124 | Bacteria | Proteobacteria | Alphaproteobacteria | Rhizobiales | Rhizobiaceae | *Agrobacterium* |  |  |
| SAF | V2108 | 1.000 | 0.025 | 0.159 | 0.0005 | Bacteria | Actinobacteria | Actinobacteria | Actinomycetales | Dermabacteraceae | *Dermabacter* |  |  |
| SAF | V2307 | 1.000 | 0.025 | 0.159 | 0.0006 | Bacteria | Proteobacteria | Alphaproteobacteria | Sphingomonadales | Sphingomonadaceae | *Sphingomonas* |  |  |
| SAF | V2365 | 0.998 | 0.025 | 0.159 | 0.0009 | Bacteria | Bacteroidetes | Sphingobacteriia | Sphingobacteriales | Sphingobacteriaceae |  |  |  |
| SAF | V2483 | 1.000 | 0.025 | 0.159 | 0.0010 | Bacteria | Bacteroidetes | Flavobacteriia | Flavobacteriales | [Weeksellaceae] | *Chryseobacterium* |  |  |
| SAF | V2496 | 1.000 | 0.025 | 0.159 | 0.0007 | Bacteria | Proteobacteria | Betaproteobacteria | Burkholderiales | Alcaligenaceae | *Pigmentiphaga* |  |  |
| SAF | V2503 | 0.921 | 0.025 | 0.153 | 0.0047 | Bacteria | Actinobacteria | Actinobacteria | Actinomycetales | Frankiaceae |  |  |  |
| SAF | V2507 | 1.000 | 0.025 | 0.159 | 0.0010 | Bacteria | Proteobacteria | Deltaproteobacteria | *Bd*ellovibrionales | *Bd*ellovibrionaceae | *Bdellovibrio* |  |  |
| SAF | V2562 | 0.957 | 0.025 | 0.155 | 0.0016 | Bacteria | Proteobacteria | Alphaproteobacteria | Rhizobiales | Methylocystaceae |  |  |  |
| SAF | V2601 | 0.884 | 0.025 | 0.149 | 0.0028 | Bacteria | Actinobacteria | Thermoleophilia | Solirubrobacterales | Conexibacteraceae |  |  |  |
| SAF | V2644 | 1.000 | 0.025 | 0.159 | 0.0008 | Bacteria | Firmicutes | Clostridia | Clostridiales | Clostridiaceae | *Clostridium* | *neonatale* |  |
| SAF | V2660 | 1.000 | 0.025 | 0.159 | 0.0007 | Bacteria | Proteobacteria | Alphaproteobacteria | Rhodospirillales | Acetobacteraceae |  |  |  |
| SAF | V2682 | 1.000 | 0.025 | 0.159 | 0.0007 | Bacteria | Actinobacteria | Actinobacteria | Actinomycetales | Sporichthyaceae |  |  |  |
| SAF | V277 | 0.739 | 0.025 | 0.137 | 0.0274 | Bacteria | Proteobacteria | Betaproteobacteria | Burkholderiales | Comamonadaceae | *Hylemonella* |  |  |
| SAF | V521 | 0.959 | 0.025 | 0.156 | 0.0015 | Bacteria | Firmicutes | Clostridia | Clostridiales | [Tissierellaceae] | *Anaerococcus* |  |  |
| SAF | V710 | 0.889 | 0.025 | 0.150 | 0.0221 | Bacteria | Proteobacteria | Gammaproteobacteria | Xanthomonadales | Sinobacteraceae |  |  |  |
| SAF | V727 | 0.902 | 0.025 | 0.151 | 0.0165 | Bacteria | Actinobacteria | Actinobacteria | Actinomycetales | Mycobacteriaceae | *Mycobacterium* | *celatum* |  |
| SAF | V734 | 1.000 | 0.025 | 0.159 | 0.0014 | Bacteria | Proteobacteria | Alphaproteobacteria | Rhizobiales | Methylocystaceae |  |  |  |
| SAF | V75 | 0.919 | 0.025 | 0.152 | 0.0457 | Bacteria | Bacteroidetes | Flavobacteriia | Flavobacteriales | [Weeksellaceae] | *Chryseobacterium* |  |  |
| SAF | V941 | 0.882 | 0.025 | 0.149 | 0.0115 | Bacteria | Actinobacteria | Thermoleophilia | Solirubrobacterales | Solirubrobacteraceae |  |  |  |
| SAF | V973 | 1.000 | 0.025 | 0.159 | 0.0008 | Bacteria | Proteobacteria | Alphaproteobacteria | Rhodospirillales | Acetobacteraceae |  |  |  |
| SAF | V983 | 0.855 | 0.025 | 0.147 | 0.0075 | Bacteria | Proteobacteria | Alphaproteobacteria | Rhizobiales |  |  |  |  |
| SAF | V1019 | 1.000 | 0.020 | 0.142 | 0.0031 | Bacteria | Proteobacteria | Alphaproteobacteria | Rhodospirillales | Acetobacteraceae |  |  |  |
| SAF | V1125 | 0.828 | 0.020 | 0.129 | 0.0196 | Bacteria | Acidobacteria | Acidobacteriia | Acidobacteriales | Acidobacteriaceae |  |  |  |
| SAF | V1141 | 1.000 | 0.020 | 0.142 | 0.0031 | Bacteria | Proteobacteria | Alphaproteobacteria | Rhodospirillales | Rhodospirillaceae |  |  |  |
| SAF | V1165 | 0.812 | 0.020 | 0.128 | 0.0251 | Bacteria | Proteobacteria | Alphaproteobacteria | Sphingomonadales | Sphingomonadaceae | *Sphingomonas* |  |  |
| SAF | V1332 | 0.837 | 0.020 | 0.130 | 0.0476 | Bacteria | Proteobacteria | Gammaproteobacteria | Methylococcales | Methylococcaceae | *Methylocaldum* |  |  |
| SAF | V1354 | 0.946 | 0.020 | 0.138 | 0.0068 | Bacteria | Bacteroidetes | Bacteroidia | Bacteroidales | Prevotellaceae | *Prevotella* | *melaninogenica* |  |
| SAF | V1428 | 0.895 | 0.020 | 0.134 | 0.0173 | Bacteria | Bacteroidetes | Sphingobacteriia | Sphingobacteriales | Sphingobacteriaceae |  |  |  |
| SAF | V1445 | 1.000 | 0.020 | 0.142 | 0.0039 | Bacteria | Proteobacteria | Alphaproteobacteria | Rhodospirillales | Acetobacteraceae | *Rhodopila* | *globiformis* |  |
| SAF | V1486 | 0.940 | 0.020 | 0.138 | 0.0092 | Bacteria | Bacteroidetes | Sphingobacteriia | Sphingobacteriales | Sphingobacteriaceae |  |  |  |
| SAF | V1532 | 1.000 | 0.020 | 0.142 | 0.0035 | Bacteria | Proteobacteria | Alphaproteobacteria | Rhizobiales |  |  |  |  |
| SAF | V1553 | 0.847 | 0.020 | 0.131 | 0.0254 | Bacteria | Chloroflexi | Ellin6529 |  |  |  |  |  |
| SAF | V1629 | 0.950 | 0.020 | 0.139 | 0.0079 | Bacteria | Proteobacteria | Gammaproteobacteria | Enterobacteriales | Enterobacteriaceae | *Pantoea* |  |  |
| SAF | V1667 | 0.876 | 0.020 | 0.133 | 0.0249 | Bacteria | Firmicutes | Erysipelotrichi | Erysipelotrichales | Erysipelotrichaceae | *[Eubacterium]* | *dolichum* |  |
| SAF | V1929 | 0.818 | 0.020 | 0.129 | 0.0373 | Bacteria | Proteobacteria | Alphaproteobacteria | Rhizobiales |  |  |  |  |
| SAF | V2006 | 1.000 | 0.020 | 0.142 | 0.0043 | Bacteria | Actinobacteria | Actinobacteria | Actinomycetales |  |  |  |  |
| SAF | V2017 | 0.808 | 0.020 | 0.128 | 0.0359 | Bacteria | Proteobacteria | Alphaproteobacteria | Sphingomonadales | Sphingomonadaceae | *Sphingomonas* | *wittichii* |  |
| SAF | V2020 | 1.000 | 0.020 | 0.142 | 0.0029 | Bacteria | Proteobacteria | Betaproteobacteria | Burkholderiales | Comamonadaceae |  |  |  |
| SAF | V2044 | 1.000 | 0.020 | 0.142 | 0.0042 | Bacteria | Bacteroidetes | Sphingobacteriia | Sphingobacteriales | Sphingobacteriaceae |  |  |  |
| SAF | V2098 | 1.000 | 0.020 | 0.142 | 0.0032 | Bacteria | Bacteroidetes | [Saprospirae] | [Saprospirales] | Chitinophagaceae | *Flavisolibacter* |  |  |
| SAF | V2116 | 1.000 | 0.020 | 0.142 | 0.0031 | Bacteria | Actinobacteria | Thermoleophilia | Solirubrobacterales | Solirubrobacteraceae |  |  |  |
| SAF | V2135 | 1.000 | 0.020 | 0.142 | 0.0039 | Bacteria | Firmicutes |  |  |  |  |  |  |
| SAF | V2165 | 1.000 | 0.020 | 0.142 | 0.0038 | Bacteria | Proteobacteria | Alphaproteobacteria | Caulobacterales | Caulobacteraceae | *Brevundimonas* | *diminuta* |  |
| SAF | V2167 | 0.979 | 0.020 | 0.141 | 0.0083 | Bacteria | Actinobacteria | Thermoleophilia | Solirubrobacterales |  |  |  |  |
| SAF | V2170 | 1.000 | 0.020 | 0.142 | 0.0037 | Bacteria | Proteobacteria | Alphaproteobacteria | Caulobacterales | Caulobacteraceae |  |  |  |
| SAF | V2171 | 0.869 | 0.020 | 0.132 | 0.0145 | Bacteria | Actinobacteria | Actinobacteria | Actinomycetales | Intrasporangiaceae |  |  |  |
| SAF | V2340 | 1.000 | 0.020 | 0.142 | 0.0039 | Bacteria | Bacteroidetes | Bacteroidia | Bacteroidales | Prevotellaceae | *Prevotella* | *intermedia* |  |
| SAF | V2377 | 1.000 | 0.020 | 0.142 | 0.0031 | Bacteria | Firmicutes | Bacilli | Lactobacillales |  |  |  |  |
| SAF | V2380 | 1.000 | 0.020 | 0.142 | 0.0034 | Bacteria | Proteobacteria | Gammaproteobacteria | Pseudomonadales | Moraxellaceae | *Acinetobacter* | *venetianus* |  |
| SAF | V2566 | 1.000 | 0.020 | 0.142 | 0.0039 | Bacteria | Proteobacteria | Alphaproteobacteria | Sphingomonadales | Sphingomonadaceae | *Sphingomonas* |  |  |
| SAF | V2585 | 0.725 | 0.020 | 0.121 | 0.0252 | Bacteria | Proteobacteria | Gammaproteobacteria | Salinisphaerales | Salinisphaeraceae | *Salinisphaera* |  |  |
| SAF | V2624 | 1.000 | 0.020 | 0.142 | 0.0029 | Bacteria | Firmicutes | Clostridia | Clostridiales | Clostridiaceae |  |  |  |
| SAF | V2626 | 1.000 | 0.020 | 0.142 | 0.0032 | Bacteria | Bacteroidetes | Bacteroidia | Bacteroidales | Bacteroidaceae | *Bacteroides* |  |  |
| SAF | V2639 | 1.000 | 0.020 | 0.142 | 0.0035 | Bacteria | Actinobacteria | Actinobacteria | Actinomycetales | Microbacteriaceae |  |  |  |
| SAF | V2688 | 1.000 | 0.020 | 0.142 | 0.0034 | Bacteria | [Thermi] | Deinococci | Deinococcales | Deinococcaceae | *Deinococcus* |  |  |
| SAF | V2716 | 1.000 | 0.020 | 0.142 | 0.0040 | Bacteria | Acidobacteria | Acidobacteriia | Acidobacteriales | Acidobacteriaceae |  |  |  |
| SAF | V385 | 0.840 | 0.020 | 0.130 | 0.0423 | Bacteria | Proteobacteria | Alphaproteobacteria | Rhizobiales | Bradyrhizobiaceae | *Bradyrhizobium* |  |  |
| SAF | V558 | 1.000 | 0.020 | 0.142 | 0.0035 | Bacteria | Proteobacteria | Alphaproteobacteria | Sphingomonadales | Sphingomonadaceae | *Sphingomonas* |  |  |
| SAF | V560 | 1.000 | 0.020 | 0.142 | 0.0030 | Bacteria | Proteobacteria | Alphaproteobacteria | Rhizobiales | Methylobacteriaceae | *Methylobacterium* |  |  |
| SAF | V588 | 0.878 | 0.020 | 0.133 | 0.0143 | Bacteria | Proteobacteria | Alphaproteobacteria | Rhizobiales |  |  |  |  |
| SAF | V651 | 0.987 | 0.020 | 0.141 | 0.0113 | Bacteria | Firmicutes | Bacilli | Lactobacillales |  |  |  |  |
| SAF | V705 | 0.912 | 0.020 | 0.136 | 0.0353 | Bacteria | Proteobacteria | Alphaproteobacteria | Rhodobacterales | Rhodobacteraceae | *Paracoccus* |  |  |
| SAF | V921 | 0.954 | 0.020 | 0.139 | 0.0284 | Bacteria | Armatimonadetes | [Fimbriimonadia] | [Fimbriimonadales] | [Fimbriimonadaceae] | *Fimbriimonas* |  |  |
| SAF | V984 | 0.972 | 0.020 | 0.140 | 0.0089 | Bacteria | Actinobacteria | Thermoleophilia | Solirubrobacterales |  |  |  |  |
| SAF | V1040 | 1.000 | 0.015 | 0.123 | 0.0127 | Bacteria | Proteobacteria | Alphaproteobacteria | Rhizobiales |  |  |  |  |
| SAF | V1095 | 1.000 | 0.015 | 0.123 | 0.0150 | Bacteria | Proteobacteria | Alphaproteobacteria | Sphingomonadales | Sphingomonadaceae | *Sphingomonas* |  |  |
| SAF | V1114 | 1.000 | 0.015 | 0.123 | 0.0135 | Bacteria | Proteobacteria | Alphaproteobacteria | Sphingomonadales | Sphingomonadaceae | *Sphingomonas* |  |  |
| SAF | V1131 | 1.000 | 0.015 | 0.123 | 0.0135 | Bacteria | Acidobacteria | Acidobacteriia | Acidobacteriales | Acidobacteriaceae |  |  |  |
| SAF | V1142 | 0.951 | 0.015 | 0.120 | 0.0289 | Bacteria | Verrucomicrobia | [Spartobacteria] | [Chthoniobacterales] | [Chthoniobacteraceae] |  |  |  |
| SAF | V1149 | 0.961 | 0.015 | 0.121 | 0.0418 | Bacteria | Proteobacteria | Alphaproteobacteria | Rhizobiales | Methylocystaceae |  |  |  |
| SAF | V1241 | 0.936 | 0.015 | 0.119 | 0.0351 | Bacteria | Proteobacteria | Alphaproteobacteria | Sphingomonadales | Sphingomonadaceae | *Sphingomonas* |  |  |
| SAF | V1245 | 1.000 | 0.015 | 0.123 | 0.0120 | Bacteria | Actinobacteria | Actinobacteria | Actinomycetales | Intrasporangiaceae | *Kytococcus* |  |  |
| SAF | V134 | 0.927 | 0.015 | 0.119 | 0.0412 | Bacteria | Proteobacteria | Gammaproteobacteria | Xanthomonadales | Xanthomonadaceae | *Dokdonella* |  |  |
| SAF | V1345 | 0.956 | 0.015 | 0.120 | 0.0231 | Bacteria | Actinobacteria | Thermoleophilia | Solirubrobacterales | Patulibacteraceae |  |  |  |
| SAF | V1375 | 0.904 | 0.015 | 0.117 | 0.0301 | Bacteria | Cyanobacteria | Nostocophycideae | Nostocales | Scytonemataceae | *Brasilonema* | *roberti-lammi* |  |
| SAF | V1376 | 0.945 | 0.015 | 0.120 | 0.0361 | Bacteria | Proteobacteria | Gammaproteobacteria | Enterobacteriales | Enterobacteriaceae |  |  |  |
| SAF | V1530 | 0.946 | 0.015 | 0.120 | 0.0243 | Bacteria | Firmicutes | Bacilli | Bacillales | Bacillaceae | *Bacillus* |  |  |
| SAF | V1540 | 1.000 | 0.015 | 0.123 | 0.0141 | Bacteria | Bacteroidetes | Bacteroidia | Bacteroidales | Bacteroidaceae | *Bacteroides* |  |  |
| SAF | V1560 | 1.000 | 0.015 | 0.123 | 0.0137 | Bacteria | Actinobacteria | Actinobacteria | Actinomycetales | Nocardioidaceae |  |  |  |
| SAF | V1586 | 1.000 | 0.015 | 0.123 | 0.0127 | Bacteria | Proteobacteria | Alphaproteobacteria | Sphingomonadales | Sphingomonadaceae | *Sphingomonas* |  |  |
| SAF | V1732 | 0.973 | 0.015 | 0.121 | 0.0247 | Bacteria | Proteobacteria | Alphaproteobacteria | Rhizobiales | Beijerinckiaceae | *Beijerinckia* |  |  |
| SAF | V1838 | 1.000 | 0.015 | 0.123 | 0.0140 | Bacteria | Proteobacteria | Betaproteobacteria | Burkholderiales | Burkholderiaceae | *Salinispora* |  |  |
| SAF | V188 | 1.000 | 0.015 | 0.123 | 0.0120 | Bacteria | Proteobacteria | Alphaproteobacteria | Rhodobacterales | Rhodobacteraceae | *Paracoccus* | *aminovorans* |  |
| SAF | V1991 | 0.959 | 0.015 | 0.121 | 0.0165 | Bacteria | Proteobacteria | Betaproteobacteria | Burkholderiales | Comamonadaceae |  |  |  |
| SAF | V1995 | 0.932 | 0.015 | 0.119 | 0.0215 | Bacteria | Firmicutes | Bacilli | Lactobacillales | Lactobacillaceae | *Lactobacillus* | *reuteri* |  |
| SAF | V2009 | 0.906 | 0.015 | 0.117 | 0.0452 | Bacteria | Actinobacteria | Actinobacteria | Actinomycetales | Micromonosporaceae |  |  |  |
| SAF | V2014 | 1.000 | 0.015 | 0.123 | 0.0136 | Bacteria | Proteobacteria | Alphaproteobacteria | Caulobacterales | Caulobacteraceae |  |  |  |
| SAF | V2018 | 1.000 | 0.015 | 0.123 | 0.0136 | Bacteria | Proteobacteria | Alphaproteobacteria | Sphingomonadales | Sphingomonadaceae | *Sphingomonas* | *wittichii* |  |
| SAF | V2066 | 0.975 | 0.015 | 0.122 | 0.0244 | Bacteria | Proteobacteria | Alphaproteobacteria | Rhodospirillales | Acetobacteraceae |  |  |  |
| SAF | V2072 | 1.000 | 0.015 | 0.123 | 0.0162 | Bacteria | Actinobacteria | Actinobacteria | Actinomycetales |  |  |  |  |
| SAF | V2094 | 1.000 | 0.015 | 0.123 | 0.0134 | Bacteria | Proteobacteria | Gammaproteobacteria | Pseudomonadales | Pseudomonadaceae | *Pseudomonas* | *viridiflava* |  |
| SAF | V2129 | 0.913 | 0.015 | 0.118 | 0.0209 | Bacteria | Armatimonadetes | Armatimonadia | Armatimonadales | Armatimonadaceae |  |  |  |
| SAF | V2133 | 1.000 | 0.015 | 0.123 | 0.0155 | Bacteria | Proteobacteria | Gammaproteobacteria | Oceanospirillales | Halomonadaceae | *Kushneria* |  |  |
| SAF | V2139 | 0.962 | 0.015 | 0.121 | 0.0214 | Bacteria | Proteobacteria | Alphaproteobacteria | Caulobacterales | Caulobacteraceae |  |  |  |
| SAF | V2144 | 1.000 | 0.015 | 0.123 | 0.0138 | Bacteria | Proteobacteria | Betaproteobacteria | Burkholderiales | Burkholderiaceae | *Burkholderia* | *tuberum* |  |
| SAF | V2149 | 1.000 | 0.015 | 0.123 | 0.0135 | Bacteria | Planctomycetes | Planctomycetia | Gemmatales | Isosphaeraceae |  |  |  |
| SAF | V2169 | 1.000 | 0.015 | 0.123 | 0.0138 | Bacteria | Actinobacteria | Actinobacteria | Actinomycetales | Frankiaceae |  |  |  |
| SAF | V2244 | 1.000 | 0.015 | 0.123 | 0.0126 | Bacteria | Actinobacteria | Thermoleophilia | Solirubrobacterales | Solirubrobacteraceae |  |  |  |
| SAF | V2341 | 1.000 | 0.015 | 0.123 | 0.0159 | Bacteria | Proteobacteria | Alphaproteobacteria | Caulobacterales | Caulobacteraceae |  |  |  |
| SAF | V2359 | 0.854 | 0.015 | 0.114 | 0.0419 | Bacteria | Firmicutes | Bacilli | Turicibacterales | Turicibacteraceae | *Turicibacter* |  |  |
| SAF | V2434 | 0.891 | 0.015 | 0.116 | 0.0334 | Bacteria | Proteobacteria | Deltaproteobacteria | Myxococcales | 0319-6G20 |  |  |  |
| SAF | V2491 | 1.000 | 0.015 | 0.123 | 0.0136 | Bacteria | Actinobacteria | Thermoleophilia | Solirubrobacterales | Conexibacteraceae |  |  |  |
| SAF | V2498 | 0.904 | 0.015 | 0.117 | 0.0217 | Bacteria | Acidobacteria | Acidobacteriia | Acidobacteriales | Acidobacteriaceae |  |  |  |
| SAF | V2510 | 1.000 | 0.015 | 0.123 | 0.0143 | Bacteria | Proteobacteria | Alphaproteobacteria | Rhizobiales |  |  |  |  |
| SAF | V2535 | 0.884 | 0.015 | 0.116 | 0.0281 | Bacteria | Proteobacteria | Alphaproteobacteria | Rhodospirillales | Acetobacteraceae |  |  |  |
| SAF | V2556 | 0.913 | 0.015 | 0.118 | 0.0325 | Bacteria | Actinobacteria | Thermoleophilia | Solirubrobacterales | Conexibacteraceae |  |  |  |
| SAF | V2559 | 0.928 | 0.015 | 0.119 | 0.0299 | Bacteria | Actinobacteria | Actinobacteria | Actinomycetales | Pseudonocardiaceae | *Pseudonocardia* |  |  |
| SAF | V2580 | 0.988 | 0.015 | 0.122 | 0.0197 | Bacteria | Actinobacteria | Actinobacteria | Actinomycetales | Microbacteriaceae | *Salinibacterium* |  |  |
| SAF | V2606 | 1.000 | 0.015 | 0.123 | 0.0141 | Bacteria | Proteobacteria | Alphaproteobacteria | Sphingomonadales | Sphingomonadaceae |  |  |  |
| SAF | V2612 | 1.000 | 0.015 | 0.123 | 0.0141 | Bacteria | Proteobacteria | Gammaproteobacteria | Xanthomonadales | Xanthomonadaceae | *Pseudoxanthomonas* |  |  |
| SAF | V2619 | 1.000 | 0.015 | 0.123 | 0.0153 | Bacteria | Cyanobacteria | Nostocophycideae | Nostocales | Nostocaceae |  |  |  |
| SAF | V2627 | 1.000 | 0.015 | 0.123 | 0.0109 | Bacteria | Bacteroidetes | Cytophagia | Cytophagales | Cytophagaceae | *Spirosoma* |  |  |
| SAF | V2633 | 1.000 | 0.015 | 0.123 | 0.0141 | Bacteria | Actinobacteria | Actinobacteria | Actinomycetales | Nocardioidaceae |  |  |  |
| SAF | V2640 | 1.000 | 0.015 | 0.123 | 0.0134 | Bacteria | Firmicutes | Clostridia | Clostridiales | Lachnospiraceae |  |  |  |
| SAF | V2655 | 1.000 | 0.015 | 0.123 | 0.0164 | Bacteria | Firmicutes | Bacilli | Lactobacillales | Aerococcaceae | *Alloiococcus* | *otitis* |  |
| SAF | V2685 | 1.000 | 0.015 | 0.123 | 0.0138 | Bacteria | Proteobacteria | Alphaproteobacteria | Sphingomonadales | Sphingomonadaceae | *Sphingomonas* | *wittichii* |  |
| SAF | V2686 | 1.000 | 0.015 | 0.123 | 0.0137 | Bacteria | Proteobacteria |  |  |  |  |  |  |
| SAF | V2687 | 1.000 | 0.015 | 0.123 | 0.0113 | Bacteria | Proteobacteria | Betaproteobacteria | Burkholderiales | Oxalobacteraceae |  |  |  |
| SAF | V2691 | 1.000 | 0.015 | 0.123 | 0.0147 | Bacteria | Proteobacteria | Alphaproteobacteria | Sphingomonadales | Sphingomonadaceae |  |  |  |
| SAF | V2699 | 1.000 | 0.015 | 0.123 | 0.0151 | Bacteria | Proteobacteria | Deltaproteobacteria | Myxococcales | Myxococcaceae | *Anaeromyxobacter* |  |  |
| SAF | V2700 | 1.000 | 0.015 | 0.123 | 0.0161 | Bacteria | Actinobacteria | Thermoleophilia | Solirubrobacterales | Conexibacteraceae |  |  |  |
| SAF | V2705 | 1.000 | 0.015 | 0.123 | 0.0151 | Bacteria | Acidobacteria | Acidobacteriia | Acidobacteriales | Acidobacteriaceae |  |  |  |
| SAF | V2706 | 1.000 | 0.015 | 0.123 | 0.0147 | Bacteria | Bacteroidetes | Bacteroidia | Bacteroidales | Prevotellaceae | *Prevotella* |  |  |
| SAF | V2708 | 1.000 | 0.015 | 0.123 | 0.0158 | Bacteria | Firmicutes | Clostridia | Clostridiales | [Tissierellaceae] | *Anaerococcus* |  |  |
| SAF | V2710 | 1.000 | 0.015 | 0.123 | 0.0140 | Bacteria | Proteobacteria | Alphaproteobacteria | Rhodospirillales | Acetobacteraceae |  |  |  |
| SAF | V2715 | 1.000 | 0.015 | 0.123 | 0.0180 | Bacteria | Proteobacteria | Alphaproteobacteria | Rhodospirillales | Acetobacteraceae |  |  |  |
| SAF | V2717 | 1.000 | 0.015 | 0.123 | 0.0151 | Bacteria | Actinobacteria | Actinobacteria | Actinomycetales | Nocardioidaceae | *Aeromicrobium* |  |  |
| SAF | V2721 | 1.000 | 0.015 | 0.123 | 0.0142 | Bacteria | Proteobacteria | Alphaproteobacteria | Rhizobiales |  |  |  |  |
| SAF | V2725 | 1.000 | 0.015 | 0.123 | 0.0154 | Bacteria | Cyanobacteria | Oscillatoriophycideae |  |  |  |  |  |
| SAF | V2726 | 1.000 | 0.015 | 0.123 | 0.0115 | Bacteria | Acidobacteria | Acidobacteriia | Acidobacteriales | Acidobacteriaceae |  |  |  |
| SAF | V2727 | 1.000 | 0.015 | 0.123 | 0.0149 | Bacteria | Cyanobacteria | Oscillatoriophycideae |  |  |  |  |  |
| SAF | V2744 | 1.000 | 0.015 | 0.123 | 0.0142 | Bacteria | Proteobacteria | Betaproteobacteria | Burkholderiales | Comamonadaceae |  |  |  |
| SAF | V2746 | 1.000 | 0.015 | 0.123 | 0.0144 | Bacteria | Proteobacteria | Alphaproteobacteria | Rhizobiales |  |  |  |  |
| SAF | V2747 | 1.000 | 0.015 | 0.123 | 0.0169 | Bacteria | Proteobacteria | Alphaproteobacteria | BD7-3 |  |  |  |  |
| SAF | V2752 | 1.000 | 0.015 | 0.123 | 0.0148 | Bacteria | Proteobacteria | Alphaproteobacteria | Rhodospirillales | Acetobacteraceae |  |  |  |
| SAF | V649 | 0.940 | 0.015 | 0.119 | 0.0493 | Bacteria | Bacteroidetes | Bacteroidia | Bacteroidales | Bacteroidaceae | *Bacteroides* |  |  |
| SAF | V656 | 1.000 | 0.015 | 0.123 | 0.0121 | Bacteria | Firmicutes | Bacilli | Lactobacillales |  |  |  |  |
| SAF | V684 | 0.940 | 0.015 | 0.119 | 0.0173 | Bacteria | Actinobacteria | Thermoleophilia | Solirubrobacterales |  |  |  |  |
| SAF | V687 | 1.000 | 0.015 | 0.123 | 0.0158 | Bacteria | Proteobacteria | Alphaproteobacteria | Sphingomonadales | Sphingomonadaceae | *Sphingomonas* | *wittichii* |  |
| SAF | V735 | 0.965 | 0.015 | 0.121 | 0.0211 | Bacteria | Fusobacteria | Fusobacteriia | Fusobacteriales | Fusobacteriaceae | *Fusobacterium* |  |  |
| SAF | V908 | 0.932 | 0.015 | 0.119 | 0.0231 | Bacteria | Cyanobacteria | Oscillatoriophycideae | Chroococcales | Xenococcaceae |  |  |  |
| SAF | V997 | 1.000 | 0.015 | 0.123 | 0.0139 | Bacteria | Proteobacteria | Alphaproteobacteria | Sphingomonadales | Sphingomonadaceae | *Sphingomonas* | *wittichii* |  |
| BD_positive | V531 | 0.743 | 0.218 | 0.403 | 0.001 | Bacteria | Proteobacteria | Gammaproteobacteria | Pseudomonadales | Moraxellaceae | *Acinetobacter* | *johnsonii* | inhibitory |
| BD_positive | V123 | 0.708 | 0.186 | 0.363 | 0.001 | Bacteria | Proteobacteria | Betaproteobacteria | Burkholderiales | Comamonadaceae | *Leptothrix* |  | inhibitory |
| BD_positive | V132 | 0.844 | 0.170 | 0.379 | 0.001 | Bacteria | Proteobacteria | Betaproteobacteria | Rhodocyclales | Rhodocyclaceae | *Hydrogenophilus* |  | na |
| BD_positive | V1474 | 0.831 | 0.170 | 0.376 | 0.001 | Bacteria | Proteobacteria | Gammaproteobacteria | Pseudomonadales | Pseudomonadaceae |  |  | na |
| BD_positive | V506 | 0.670 | 0.117 | 0.280 | 0.014 | Bacteria | Proteobacteria | Betaproteobacteria | Burkholderiales | Oxalobacteraceae | *Massilia* | *alkalitolerans* | inhibitory |
| BD_positive | V155 | 0.873 | 0.112 | 0.312 | 0.001 | Bacteria | Proteobacteria | Alphaproteobacteria | Rhizobiales | Rhizobiaceae | *Agrobacterium* |  | na |
| BD_positive | V313 | 0.898 | 0.106 | 0.309 | 0.002 | Bacteria | Actinobacteria | Rubrobacteria | Rubrobacterales | Rubrobacteraceae | *Rubrobacter* |  | inhibitory |
| BD_positive | V1294 | 0.868 | 0.106 | 0.304 | 0.001 | Bacteria | Proteobacteria | Alphaproteobacteria | Rhizobiales | Methylocystaceae | *Methylosinus* |  | na |
| BD_positive | V21 | 0.880 | 0.106 | 0.306 | 0.005 | Bacteria | Proteobacteria | Alphaproteobacteria | Sphingomonadales | Sphingomonadaceae | *Sphingobium* | *yanoikuyae* | na |
| BD_positive | V669 | 0.610 | 0.106 | 0.255 | 0.040 | Bacteria | Proteobacteria | Gammaproteobacteria | Enterobacteriales | Enterobacteriaceae |  |  | na |
| BD_positive | V55 | 0.901 | 0.101 | 0.302 | 0.001 | Bacteria | Bacteroidetes | Flavobacteriia | Flavobacteriales | [Weeksellaceae] | *Cloacibacterium* |  | na |
| BD_positive | V27 | 0.859 | 0.096 | 0.287 | 0.001 | Bacteria | Proteobacteria | Alphaproteobacteria | Rhodobacterales | Rhodobacteraceae | *Paracoccus* |  | inhibitory |
| BD_positive | V329 | 0.809 | 0.096 | 0.278 | 0.023 | Bacteria | Proteobacteria | Alphaproteobacteria | Sphingomonadales | Sphingomonadaceae | *Novosphingobium* |  | na |
| BD_positive | V1112 | 0.685 | 0.090 | 0.249 | 0.042 | Bacteria | Actinobacteria | Actinobacteria | Actinomycetales | Brevibacteriaceae | *Brevibacterium* | *aureum* | inhibitory |
| BD_positive | V122 | 0.916 | 0.090 | 0.288 | 0.001 | Bacteria | Firmicutes | Clostridia | Clostridiales | Clostridiaceae | *Clostridium* | *butyricum* | inhibitory |
| BD_positive | V1023 | 0.795 | 0.090 | 0.268 | 0.003 | Bacteria | Proteobacteria | Alphaproteobacteria | Sphingomonadales | Sphingomonadaceae | *Sphingomonas* |  | na |
| BD_positive | V434 | 0.838 | 0.090 | 0.275 | 0.002 | Bacteria | Proteobacteria | Betaproteobacteria | Neisseriales | Neisseriaceae |  |  | na |
| BD_positive | V586 | 0.709 | 0.085 | 0.246 | 0.012 | Bacteria | Proteobacteria | Gammaproteobacteria | Xanthomonadales | Xanthomonadaceae |  |  | na |
| BD_positive | V1004 | 0.756 | 0.080 | 0.246 | 0.014 | Bacteria | Proteobacteria | Gammaproteobacteria | Enterobacteriales | Enterobacteriaceae |  |  | inhibitory |
| BD_positive | V1173 | 0.814 | 0.080 | 0.255 | 0.001 | Bacteria | Firmicutes | Bacilli | Bacillales | Planococcaceae |  |  | na |
| BD_positive | V1881 | 0.929 | 0.080 | 0.272 | 0.001 | Bacteria | Proteobacteria | Epsilonproteobacteria | Campylobacterales | Campylobacteraceae | *Sulfurospirillum* |  | na |
| BD_positive | V636 | 0.808 | 0.080 | 0.254 | 0.003 | Bacteria | Proteobacteria | Betaproteobacteria | Burkholderiales | Comamonadaceae | *Rhodoferax* |  | na |
| BD_positive | V1297 | 0.879 | 0.074 | 0.256 | 0.001 | Bacteria | Proteobacteria | Betaproteobacteria | Burkholderiales | Comamonadaceae | *Limnohabitans* |  | inhibitory |
| BD_positive | V2387 | 0.910 | 0.074 | 0.260 | 0.001 | Bacteria | Proteobacteria | Gammaproteobacteria | Pseudomonadales | Pseudomonadaceae |  |  | inhibitory |
| BD_positive | V526 | 0.902 | 0.074 | 0.259 | 0.001 | Bacteria | Bacteroidetes | Bacteroidia | Bacteroidales | Bacteroidaceae | *Bacteroides* |  | inhibitory |
| BD_positive | V540 | 0.850 | 0.074 | 0.252 | 0.002 | Bacteria | Proteobacteria | Gammaproteobacteria | Pseudomonadales | Pseudomonadaceae | *Pseudomonas* | *viridiflava* | na |
| BD_positive | V585 | 0.833 | 0.074 | 0.249 | 0.003 | Bacteria | Proteobacteria | Betaproteobacteria | Burkholderiales | Comamonadaceae |  |  | na |
| BD_positive | V1300 | 0.800 | 0.069 | 0.235 | 0.001 | Bacteria | Verrucomicrobia | [Pedosphaerae] | [Pedosphaerales] | auto67_4W |  |  | na |
| BD_positive | V203 | 0.612 | 0.069 | 0.206 | 0.016 | Bacteria | Proteobacteria | Betaproteobacteria | Methylophilales | Methylophilaceae | *Methylotenera* | *mobilis* | na |
| BD_positive | V232 | 0.904 | 0.069 | 0.250 | 0.001 | Bacteria | Proteobacteria | Betaproteobacteria | Rhodocyclales | Rhodocyclaceae | *Dechloromonas* |  | na |
| BD_positive | V2393 | 0.890 | 0.069 | 0.248 | 0.001 | Bacteria | Proteobacteria | Epsilonproteobacteria | Campylobacterales | Helicobacteraceae |  |  | na |
| BD_positive | V2394 | 0.897 | 0.069 | 0.249 | 0.001 | Bacteria | Bacteroidetes | Cytophagia | Cytophagales | Cytophagaceae | *Flectobacillus* |  | na |
| BD_positive | V438 | 0.841 | 0.069 | 0.241 | 0.009 | Bacteria | Proteobacteria | Betaproteobacteria | Neisseriales | Neisseriaceae |  |  | na |
| BD_positive | V486 | 0.893 | 0.069 | 0.248 | 0.001 | Bacteria | Proteobacteria | Betaproteobacteria | Burkholderiales | Comamonadaceae | *Rubrivivax* |  | na |
| BD_positive | V641 | 0.709 | 0.069 | 0.221 | 0.012 | Bacteria | Actinobacteria | Actinobacteria | Actinomycetales |  |  |  | na |
| BD_positive | V68 | 0.860 | 0.069 | 0.244 | 0.001 | Bacteria | Bacteroidetes | Sphingobacteriia | Sphingobacteriales |  |  |  | na |
| BD_positive | V1579 | 0.918 | 0.064 | 0.242 | 0.001 | Bacteria | Proteobacteria | Betaproteobacteria | Rhodocyclales | Rhodocyclaceae | *C39* |  | inhibitory |
| BD_positive | V208 | 0.736 | 0.064 | 0.217 | 0.005 | Bacteria | Proteobacteria | Alphaproteobacteria | Rhodobacterales | Rhodobacteraceae | *Rhodobacter* |  | inhibitory |
| BD_positive | V38 | 0.777 | 0.064 | 0.223 | 0.004 | Bacteria | Proteobacteria | Gammaproteobacteria | Xanthomonadales | Xanthomonadaceae | *Pseudoxanthomonas* | *mexicana* | inhibitory |
| BD_positive | V694 | 0.912 | 0.064 | 0.241 | 0.001 | Bacteria | Proteobacteria | Betaproteobacteria | Rhodocyclales | Rhodocyclaceae | *Zoogloea* |  | na |
| BD_positive | V881 | 0.929 | 0.064 | 0.244 | 0.001 | Bacteria | Proteobacteria | Betaproteobacteria | Burkholderiales | Comamonadaceae | *Methylibium* |  | na |
| BD_positive | V95 | 0.846 | 0.064 | 0.232 | 0.001 | Bacteria | Bacteroidetes | Flavobacteriia | Flavobacteriales | Flavobacteriaceae | *Flavobacterium* | *succinicans* | na |
| BD_positive | V986 | 0.709 | 0.064 | 0.213 | 0.005 | Bacteria | Actinobacteria | Actinobacteria | Bifidobacteriales | Bifidobacteriaceae | *Bifidobacterium* | *adolescentis* | na |
| BD_positive | V1073 | 0.773 | 0.059 | 0.213 | 0.025 | Bacteria | Bacteroidetes | Cytophagia | Cytophagales | Cytophagaceae | *Spirosoma* |  | inhibitory |
| BD_positive | V1163 | 0.923 | 0.059 | 0.232 | 0.009 | Bacteria | Bacteroidetes | Flavobacteriia | Flavobacteriales | [Weeksellaceae] | *Chryseobacterium* |  | inhibitory |
| BD_positive | V1333 | 0.847 | 0.059 | 0.223 | 0.002 | Bacteria | Proteobacteria | Betaproteobacteria | Burkholderiales | Comamonadaceae | *Hydrogenophaga* |  | na |
| BD_positive | V1700 | 0.834 | 0.059 | 0.221 | 0.003 | Bacteria | Proteobacteria | Alphaproteobacteria | Rhizobiales | Methylocystaceae | *Rhodoblastus* | *acidophilus* | na |
| BD_positive | V1811 | 0.890 | 0.059 | 0.228 | 0.001 | Bacteria | Proteobacteria | Gammaproteobacteria | Methylococcales | Methylococcaceae |  |  | na |
| BD_positive | V2338 | 0.922 | 0.059 | 0.232 | 0.001 | Bacteria | Proteobacteria | Gammaproteobacteria | Pseudomonadales | Pseudomonadaceae | *Pseudomonas* | *thermotolerans* | na |
| BD_positive | V2397 | 0.905 | 0.059 | 0.230 | 0.001 | Bacteria | Bacteroidetes | Bacteroidia | Bacteroidales | Bacteroidaceae | *Bacteroides* |  | na |
| BD_positive | V2398 | 0.888 | 0.059 | 0.228 | 0.001 | Bacteria | Bacteroidetes | Bacteroidia | Bacteroidales | [Paraprevotellaceae] | *YRC22* |  | na |
| BD_positive | V2419 | 0.911 | 0.059 | 0.231 | 0.001 | Bacteria | Bacteroidetes | Flavobacteriia | Flavobacteriales | Cryomorphaceae | *Fluviicola* |  | na |
| BD_positive | V639 | 0.770 | 0.059 | 0.212 | 0.012 | Bacteria | Actinobacteria | Actinobacteria | Actinomycetales | Microbacteriaceae | *Frigoribacterium* |  | na |
| BD_positive | V661 | 0.977 | 0.059 | 0.239 | 0.001 | Bacteria | Proteobacteria | Gammaproteobacteria | Methylococcales |  |  |  | na |
| BD_positive | V994 | 0.696 | 0.059 | 0.202 | 0.015 | Bacteria | Proteobacteria | Betaproteobacteria | Burkholderiales | Comamonadaceae | *Rubrivivax* |  | na |
| BD_positive | V1682 | 0.816 | 0.053 | 0.208 | 0.001 | Bacteria | Proteobacteria | Betaproteobacteria | Burkholderiales | Comamonadaceae |  |  | na |
| BD_positive | V1733 | 0.739 | 0.053 | 0.198 | 0.004 | Bacteria | Proteobacteria | Betaproteobacteria | Burkholderiales | Comamonadaceae | *Diaphorobacter* |  | na |
| BD_positive | V1740 | 0.738 | 0.053 | 0.198 | 0.010 | Bacteria | Proteobacteria | Betaproteobacteria | Burkholderiales | Comamonadaceae | *Rubrivivax* |  | na |
| BD_positive | V2229 | 0.847 | 0.053 | 0.212 | 0.001 | Bacteria | Bacteroidetes | Sphingobacteriia | Sphingobacteriales |  |  |  | na |
| BD_positive | V2421 | 0.941 | 0.053 | 0.224 | 0.001 | Bacteria |  |  |  |  |  |  | na |
| BD_positive | V261 | 0.837 | 0.053 | 0.211 | 0.007 | Bacteria | Proteobacteria | Alphaproteobacteria | Caulobacterales | Caulobacteraceae | *Phenylobacterium* |  | na |
| BD_positive | V831 | 0.852 | 0.053 | 0.213 | 0.003 | Bacteria | Proteobacteria | Betaproteobacteria | Burkholderiales | Comamonadaceae | *Methylibium* |  | na |
| BD_positive | V1056 | 0.810 | 0.048 | 0.197 | 0.002 | Bacteria | Bacteroidetes | [Saprospirae] | [Saprospirales] | Chitinophagaceae |  |  | inhibitory |
| BD_positive | V108 | 0.850 | 0.048 | 0.202 | 0.009 | Bacteria | Proteobacteria | Betaproteobacteria | Burkholderiales | Comamonadaceae | *Hydrogenophaga* |  | inhibitory |
| BD_positive | V1254 | 0.856 | 0.048 | 0.202 | 0.003 | Bacteria | Gemmatimonadetes | Gemmatimonadetes | Gemmatimonadales | A1-B1 |  |  | inhibitory |
| BD_positive | V1298 | 0.875 | 0.048 | 0.205 | 0.001 | Bacteria | Proteobacteria | Gammaproteobacteria | Pseudomonadales | Moraxellaceae | *Acinetobacter* |  | inhibitory |
| BD_positive | V1314 | 0.720 | 0.048 | 0.186 | 0.017 | Bacteria | Proteobacteria | Alphaproteobacteria | Rhizobiales |  |  |  | na |
| BD_positive | V1896 | 0.914 | 0.048 | 0.209 | 0.001 | Bacteria | Bacteroidetes | Bacteroidia | Bacteroidales |  |  |  | na |
| BD_positive | V1899 | 0.962 | 0.048 | 0.215 | 0.001 | Bacteria | Acidobacteria | Holophagae | Holophagales | Holophagaceae | *Geothrix* |  | na |
| BD_positive | V1927 | 0.962 | 0.048 | 0.215 | 0.001 | Archaea | Euryarchaeota | Methanobacteria | Methanobacteriales | Methanobacteriaceae | *Methanobacterium* |  | na |
| BD_positive | V1969 | 0.914 | 0.048 | 0.209 | 0.001 | Bacteria | Bacteroidetes | Bacteroidia | Bacteroidales | Prevotellaceae | *Prevotella* |  | na |
| BD_positive | V209 | 0.876 | 0.048 | 0.205 | 0.001 | Bacteria | Proteobacteria | Gammaproteobacteria | Pseudomonadales | Pseudomonadaceae | *Pseudomonas* | *fragi* | na |
| BD_positive | V2176 | 0.930 | 0.048 | 0.211 | 0.003 | Bacteria | Proteobacteria | Betaproteobacteria | Rhodocyclales | Rhodocyclaceae |  |  | na |
| BD_positive | V239 | 0.791 | 0.048 | 0.195 | 0.006 | Bacteria | Actinobacteria | Actinobacteria | Actinomycetales | Corynebacteriaceae | *Corynebacterium* |  | na |
| BD_positive | V2449 | 0.943 | 0.048 | 0.212 | 0.002 | Bacteria | Proteobacteria | Alphaproteobacteria | Rhodobacterales | Rhodobacteraceae | *Rhodobacter* |  | na |
| BD_positive | V264 | 0.720 | 0.048 | 0.186 | 0.010 | Bacteria | Actinobacteria | Rubrobacteria | Rubrobacterales | Rubrobacteraceae | *Rubrobacter* |  | na |
| BD_positive | V361 | 0.759 | 0.048 | 0.191 | 0.033 | Bacteria | Bacteroidetes | Bacteroidia | Bacteroidales | Porphyromonadaceae | *Parabacteroides* |  | na |
| BD_positive | V401 | 0.839 | 0.048 | 0.200 | 0.002 | Bacteria | Proteobacteria | Alphaproteobacteria | Rhizobiales |  |  |  | na |
| BD_positive | V662 | 0.862 | 0.048 | 0.203 | 0.007 | Bacteria | Proteobacteria | Betaproteobacteria | Burkholderiales | Comamonadaceae | *Curvibacter* |  | na |
| BD_positive | V782 | 0.925 | 0.048 | 0.210 | 0.001 | Bacteria | Firmicutes | Clostridia | Clostridiales | Lachnospiraceae |  |  | na |
| BD_positive | V789 | 0.842 | 0.048 | 0.201 | 0.001 | Bacteria | Actinobacteria | Acidimicrobiia | Acidimicrobiales | C111 |  |  | na |
| BD_positive | V793 | 0.967 | 0.048 | 0.215 | 0.001 | Bacteria | Proteobacteria | Betaproteobacteria | Rhodocyclales | Rhodocyclaceae | *Propionivibrio* |  | na |
| BD_positive | V818 | 0.715 | 0.048 | 0.185 | 0.026 | Bacteria | Proteobacteria | Alphaproteobacteria | Rhizobiales | Methylobacteriaceae | *Methylobacterium* | *adhaesivum* | na |
| BD_positive | V855 | 0.713 | 0.048 | 0.185 | 0.024 | Bacteria | Actinobacteria | Thermoleophilia | Solirubrobacterales | Solirubrobacteraceae | *Solirubrobacter* |  | na |
| BD_positive | V949 | 0.900 | 0.048 | 0.208 | 0.001 | Bacteria | Bacteroidetes | Sphingobacteriia | Sphingobacteriales |  |  |  | na |
| BD_positive | V1018 | 0.830 | 0.043 | 0.188 | 0.014 | Bacteria | Proteobacteria | Alphaproteobacteria | Sphingomonadales | Sphingomonadaceae | *Sphingomonas* |  | inhibitory |
| BD_positive | V1106 | 0.778 | 0.043 | 0.182 | 0.005 | Bacteria | Proteobacteria | Alphaproteobacteria | Rhizobiales | Methylocystaceae |  |  | inhibitory |
| BD_positive | V1183 | 0.829 | 0.043 | 0.188 | 0.047 | Bacteria | Firmicutes | Bacilli | Bacillales |  |  |  | inhibitory |
| BD_positive | V1309 | 0.941 | 0.043 | 0.200 | 0.001 | Bacteria | Actinobacteria | Thermoleophilia | Solirubrobacterales |  |  |  | inhibitory |
| BD_positive | V1319 | 0.777 | 0.043 | 0.182 | 0.001 | Bacteria | Proteobacteria | Betaproteobacteria | Burkholderiales |  |  |  | na |
| BD_positive | V1427 | 0.897 | 0.043 | 0.195 | 0.001 | Bacteria | Proteobacteria | Deltaproteobacteria | Myxococcales |  |  |  | na |
| BD_positive | V1783 | 0.899 | 0.043 | 0.196 | 0.001 | Bacteria | Proteobacteria | Alphaproteobacteria | Rhodospirillales |  |  |  | na |
| BD_positive | V1785 | 0.849 | 0.043 | 0.190 | 0.003 | Bacteria | Bacteroidetes | Flavobacteriia | Flavobacteriales | Flavobacteriaceae | *Flavobacterium* |  | na |
| BD_positive | V2146 | 0.865 | 0.043 | 0.192 | 0.001 | Bacteria | Proteobacteria | Betaproteobacteria | Burkholderiales | Comamonadaceae | *Rubrivivax* |  | na |
| BD_positive | V2383 | 0.899 | 0.043 | 0.196 | 0.001 | Bacteria | Proteobacteria | Betaproteobacteria | Rhodocyclales | Rhodocyclaceae | *Dechloromonas* |  | na |
| BD_positive | V2386 | 0.993 | 0.043 | 0.206 | 0.001 | Bacteria | Firmicutes | Clostridia | OPB54 |  |  |  | na |
| BD_positive | V2420 | 0.928 | 0.043 | 0.199 | 0.001 | Bacteria | Firmicutes | Clostridia | Clostridiales | Clostridiaceae | *Clostridium* |  | na |
| BD_positive | V334 | 0.876 | 0.043 | 0.193 | 0.017 | Bacteria | Proteobacteria | Alphaproteobacteria | Rhizobiales | Methylocystaceae | *Pleomorphomonas* |  | na |
| BD_positive | V465 | 0.562 | 0.043 | 0.155 | 0.050 | Bacteria | Proteobacteria | Betaproteobacteria | Neisseriales | Neisseriaceae |  |  | na |
| BD_positive | V696 | 0.882 | 0.043 | 0.194 | 0.044 | Bacteria | Proteobacteria | Gammaproteobacteria | Legionellales | Coxiellaceae | *Rickettsiella* |  | na |
| BD_positive | V700 | 0.878 | 0.043 | 0.193 | 0.001 | Bacteria | Proteobacteria | Betaproteobacteria | Burkholderiales | Comamonadaceae |  |  | na |
| BD_positive | V704 | 0.982 | 0.043 | 0.204 | 0.001 | Bacteria | [Thermi] | Deinococci | Thermales | Thermaceae | *Thermus* |  | na |
| BD_positive | V842 | 0.752 | 0.043 | 0.179 | 0.012 | Bacteria | Proteobacteria | Alphaproteobacteria | Rhizobiales |  |  |  | na |
| BD_positive | V920 | 0.702 | 0.043 | 0.173 | 0.021 | Bacteria | Proteobacteria | Gammaproteobacteria | Xanthomonadales | Xanthomonadaceae | *Stenotrophomonas* | *maltophilia* | na |
| BD_positive | V955 | 0.738 | 0.043 | 0.177 | 0.020 | Bacteria | Verrucomicrobia | [Spartobacteria] | [Chthoniobacterales] | [Chthoniobacteraceae] | *DA101* |  | na |
| BD_positive | V1034 | 0.959 | 0.037 | 0.189 | 0.028 | Bacteria | Bacteroidetes | Flavobacteriia | Flavobacteriales | [Weeksellaceae] | *Chryseobacterium* |  | inhibitory |
| BD_positive | V1128 | 0.681 | 0.037 | 0.159 | 0.040 | Bacteria | Bacteroidetes | [Saprospirae] | [Saprospirales] | Chitinophagaceae | *Sediminibacterium* |  | inhibitory |
| BD_positive | V1150 | 0.791 | 0.037 | 0.172 | 0.015 | Bacteria | Proteobacteria | Betaproteobacteria | Burkholderiales | Comamonadaceae |  |  | inhibitory |
| BD_positive | V1252 | 0.737 | 0.037 | 0.166 | 0.025 | Bacteria | Proteobacteria | Betaproteobacteria | Burkholderiales | Comamonadaceae | *Methylibium* |  | na |
| BD_positive | V1264 | 0.932 | 0.037 | 0.186 | 0.004 | Bacteria | Proteobacteria | Gammaproteobacteria | Xanthomonadales | Sinobacteraceae |  |  | na |
| BD_positive | V1315 | 0.984 | 0.037 | 0.191 | 0.001 | Bacteria | Proteobacteria | Betaproteobacteria | Rhodocyclales | Rhodocyclaceae | *Uliginosibacterium* |  | na |
| BD_positive | V1394 | 0.762 | 0.037 | 0.168 | 0.018 | Bacteria | Proteobacteria | Gammaproteobacteria | Xanthomonadales | Xanthomonadaceae | *Lysobacter* |  | na |
| BD_positive | V158 | 0.957 | 0.037 | 0.189 | 0.001 | Bacteria | Proteobacteria | Gammaproteobacteria | Oceanospirillales | Alcanivoracaceae | *Alcanivorax* |  | na |
| BD_positive | V1607 | 0.722 | 0.037 | 0.164 | 0.031 | Bacteria | Bacteroidetes | Cytophagia | Cytophagales | Cytophagaceae |  |  | na |
| BD_positive | V161 | 0.681 | 0.037 | 0.159 | 0.039 | Bacteria | Proteobacteria | Alphaproteobacteria | Caulobacterales | Caulobacteraceae | *Mycoplana* |  | na |
| BD_positive | V1636 | 0.689 | 0.037 | 0.160 | 0.026 | Bacteria | Proteobacteria | Alphaproteobacteria | Rhodospirillales | Acetobacteraceae |  |  | na |
| BD_positive | V166 | 0.743 | 0.037 | 0.166 | 0.022 | Bacteria | Proteobacteria | Alphaproteobacteria | Rhodobacterales | Rhodobacteraceae | *Rhodobacter* |  | na |
| BD_positive | V1717 | 0.826 | 0.037 | 0.175 | 0.027 | Bacteria | Proteobacteria | Betaproteobacteria | Burkholderiales | Comamonadaceae | *Hylemonella* |  | na |
| BD_positive | V1775 | 0.951 | 0.037 | 0.188 | 0.002 | Bacteria | Proteobacteria | Betaproteobacteria | Burkholderiales | Comamonadaceae |  |  | na |
| BD_positive | V1998 | 0.992 | 0.037 | 0.192 | 0.001 | Bacteria | Verrucomicrobia | Opitutae | Opitutales | Opitutaceae | *Opitutus* |  | na |
| BD_positive | V2027 | 0.795 | 0.037 | 0.172 | 0.004 | Bacteria | Bacteroidetes | Bacteroidia | Bacteroidales | [Paraprevotellaceae] | *[Prevotella]* |  | na |
| BD_positive | V2179 | 0.810 | 0.037 | 0.174 | 0.009 | Bacteria | Proteobacteria | Alphaproteobacteria | Rhizobiales | Hyphomicrobiaceae | *Rhodoplanes* |  | na |
| BD_positive | V2384 | 0.985 | 0.037 | 0.192 | 0.001 | Bacteria | Proteobacteria | Alphaproteobacteria | Rhodospirillales | Rhodospirillaceae | *Azospirillum* | *amazonense* | na |
| BD_positive | V2391 | 0.987 | 0.037 | 0.192 | 0.001 | Bacteria | Verrucomicrobia | [Pedosphaerae] | [Pedosphaerales] | auto67_4W |  |  | na |
| BD_positive | V2400 | 0.976 | 0.037 | 0.191 | 0.001 | Bacteria | Proteobacteria | Deltaproteobacteria | Spirobacillales |  |  |  | na |
| BD_positive | V2426 | 0.781 | 0.037 | 0.171 | 0.004 | Bacteria | Proteobacteria | Alphaproteobacteria | Rhodospirillales | Acetobacteraceae |  |  | na |
| BD_positive | V2440 | 0.972 | 0.037 | 0.190 | 0.001 | Bacteria | Proteobacteria | Epsilonproteobacteria | Campylobacterales | Campylobacteraceae | *Arcobacter* |  | na |
| BD_positive | V2445 | 1.000 | 0.037 | 0.193 | 0.001 | Bacteria | Actinobacteria | Rubrobacteria | Rubrobacterales | Rubrobacteraceae | *Rubrobacter* |  | na |
| BD_positive | V2453 | 0.839 | 0.037 | 0.177 | 0.003 | Bacteria | Elusimicrobia | Elusimicrobia | Elusimicrobiales | Elusimicrobiaceae | *Elusimicrobium* |  | na |
| BD_positive | V399 | 0.807 | 0.037 | 0.173 | 0.003 | Bacteria | Proteobacteria | Gammaproteobacteria | Enterobacteriales | Enterobacteriaceae | *Dickeya* |  | na |
| BD_positive | V480 | 0.793 | 0.037 | 0.172 | 0.026 | Bacteria | Proteobacteria | Gammaproteobacteria | Pseudomonadales | Pseudomonadaceae | *Pseudomonas* | *viridiflava* | na |
| BD_positive | V51 | 0.865 | 0.037 | 0.179 | 0.004 | Bacteria | Proteobacteria | Betaproteobacteria | Rhodocyclales | Rhodocyclaceae | *Zoogloea* |  | na |
| BD_positive | V512 | 0.738 | 0.037 | 0.166 | 0.017 | Bacteria | Firmicutes | Bacilli | Bacillales | Bacillaceae | *Bacillus* | *foraminis* | na |
| BD_positive | V741 | 0.930 | 0.037 | 0.186 | 0.009 | Bacteria | Firmicutes | Clostridia | Clostridiales | Veillonellaceae |  |  | na |
| BD_positive | V766 | 0.658 | 0.037 | 0.157 | 0.027 | Bacteria | Proteobacteria | Betaproteobacteria | MND1 |  |  |  | na |
| BD_positive | V779 | 0.803 | 0.037 | 0.173 | 0.003 | Bacteria | Bacteroidetes | [Saprospirae] | [Saprospirales] | Chitinophagaceae |  |  | na |
| BD_positive | V847 | 0.810 | 0.037 | 0.174 | 0.024 | Bacteria | Proteobacteria | Alphaproteobacteria | Rhizobiales | Methylobacteriaceae | *Methylobacterium* | *adhaesivum* | na |
| BD_positive | V887 | 0.676 | 0.037 | 0.159 | 0.024 | Bacteria | Proteobacteria | Betaproteobacteria | Burkholderiales | Comamonadaceae | *Methylibium* |  | na |
| BD_positive | V929 | 0.794 | 0.037 | 0.172 | 0.017 | Bacteria | Proteobacteria | Alphaproteobacteria | Rhizobiales | Hyphomicrobiaceae | *Rhodoplanes* |  | na |
| BD_positive | V948 | 0.803 | 0.037 | 0.173 | 0.014 | Bacteria | Proteobacteria | Alphaproteobacteria | Rhizobiales | Methylobacteriaceae | *Methylobacterium* | *organophilum* | na |
| BD_positive | V1103 | 0.797 | 0.032 | 0.159 | 0.017 | Bacteria | Proteobacteria | Deltaproteobacteria | Syntrophobacterales | Syntrophaceae |  |  | inhibitory |
| BD_positive | V1145 | 0.737 | 0.032 | 0.153 | 0.012 | Bacteria | Proteobacteria | Gammaproteobacteria | Xanthomonadales | Sinobacteraceae |  |  | inhibitory |
| BD_positive | V1400 | 0.828 | 0.032 | 0.163 | 0.025 | Bacteria | Proteobacteria | Alphaproteobacteria | Rhizobiales | Methylobacteriaceae | *Methylobacterium* | *organophilum* | inhibitory |
| BD_positive | V1436 | 0.828 | 0.032 | 0.163 | 0.004 | Bacteria | Firmicutes | Bacilli | Bacillales |  |  |  | inhibitory |
| BD_positive | V1463 | 0.786 | 0.032 | 0.158 | 0.010 | Bacteria | Proteobacteria | Alphaproteobacteria | Caulobacterales | Caulobacteraceae |  |  | inhibitory |
| BD_positive | V1663 | 0.918 | 0.032 | 0.171 | 0.009 | Bacteria | Proteobacteria | Alphaproteobacteria | Rhodobacterales | Rhodobacteraceae | *Rhodobacter* |  | na |
| BD_positive | V1670 | 0.782 | 0.032 | 0.158 | 0.045 | Bacteria | Actinobacteria | Actinobacteria | Actinomycetales | Nocardioidaceae | *Pimelobacter* |  | na |
| BD_positive | V1699 | 0.970 | 0.032 | 0.176 | 0.003 | Bacteria | Proteobacteria | Alphaproteobacteria | Rhodospirillales | Rhodospirillaceae | *Azospirillum* | *amazonense* | na |
| BD_positive | V1708 | 0.846 | 0.032 | 0.164 | 0.014 | Bacteria | Acidobacteria | Acidobacteria-6 | CCU21 |  |  |  | na |
| BD_positive | V1709 | 0.951 | 0.032 | 0.174 | 0.001 | Bacteria | Proteobacteria | Alphaproteobacteria | Sphingomonadales | Sphingomonadaceae | *Novosphingobium* |  | na |
| BD_positive | V187 | 1.000 | 0.032 | 0.179 | 0.001 | Bacteria | Proteobacteria | Betaproteobacteria | Rhodocyclales | Rhodocyclaceae | *Dechloromonas* |  | na |
| BD_positive | V1887 | 0.931 | 0.032 | 0.172 | 0.017 | Bacteria | Proteobacteria | Betaproteobacteria | Burkholderiales | Comamonadaceae | *Methylibium* |  | na |
| BD_positive | V1963 | 1.000 | 0.032 | 0.179 | 0.002 | Bacteria | Proteobacteria | Alphaproteobacteria | Sphingomonadales | Sphingomonadaceae | *Novosphingobium* |  | na |
| BD_positive | V1972 | 1.000 | 0.032 | 0.179 | 0.001 | Bacteria | Cyanobacteria | 4C0d-2 | YS2 |  |  |  | na |
| BD_positive | V218 | 0.870 | 0.032 | 0.167 | 0.008 | Bacteria | Bacteroidetes | Cytophagia | Cytophagales | Cytophagaceae | *Runella* |  | na |
| BD_positive | V2184 | 0.861 | 0.032 | 0.166 | 0.009 | Bacteria | Proteobacteria | Alphaproteobacteria | Rhizobiales |  |  |  | na |
| BD_positive | V2288 | 0.805 | 0.032 | 0.160 | 0.015 | Bacteria | Firmicutes | Bacilli | Bacillales | Bacillaceae | *Anoxybacillus* | *kestanbolensis* | na |
| BD_positive | V2342 | 0.898 | 0.032 | 0.169 | 0.005 | Bacteria | Bacteroidetes | Flavobacteriia | Flavobacteriales | Flavobacteriaceae | *Flavobacterium* | *succinicans* | na |
| BD_positive | V2402 | 0.865 | 0.032 | 0.166 | 0.005 | Bacteria | Proteobacteria | Gammaproteobacteria | Methylococcales |  |  |  | na |
| BD_positive | V2404 | 0.951 | 0.032 | 0.174 | 0.001 | Bacteria | Firmicutes | Clostridia | Clostridiales | Clostridiaceae | *Caloramator* |  | na |
| BD_positive | V2405 | 0.911 | 0.032 | 0.171 | 0.001 | Bacteria | Proteobacteria | Gammaproteobacteria | Methylococcales | Crenotrichaceae | *Crenothrix* |  | na |
| BD_positive | V2414 | 0.970 | 0.032 | 0.176 | 0.001 | Bacteria | Verrucomicrobia | Opitutae | Opitutales | Opitutaceae | *Opitutus* |  | na |
| BD_positive | V2425 | 0.978 | 0.032 | 0.177 | 0.001 | Bacteria | Proteobacteria | Alphaproteobacteria | Rhodobacterales | Rhodobacteraceae | *Paracoccus* | *aminovorans* | na |
| BD_positive | V2452 | 0.865 | 0.032 | 0.166 | 0.003 | Bacteria | Proteobacteria | Betaproteobacteria | Procabacteriales | Procabacteriaceae |  |  | na |
| BD_positive | V2456 | 0.975 | 0.032 | 0.176 | 0.002 | Bacteria | Proteobacteria | Epsilonproteobacteria | Campylobacterales | Helicobacteraceae | *Wolinella* | *succinogenes* | na |
| BD_positive | V372 | 0.781 | 0.032 | 0.158 | 0.007 | Bacteria | Actinobacteria | Actinobacteria | Actinomycetales | Mycobacteriaceae | *Mycobacterium* |  | na |
| BD_positive | V571 | 0.849 | 0.032 | 0.165 | 0.038 | Bacteria | Proteobacteria | Betaproteobacteria | Burkholderiales | Comamonadaceae | *Methylibium* |  | na |
| BD_positive | V59 | 0.849 | 0.032 | 0.165 | 0.020 | Bacteria | Proteobacteria | Alphaproteobacteria | Rhodobacterales | Rhodobacteraceae | *Rhodobacter* |  | na |
| BD_positive | V675 | 0.777 | 0.032 | 0.157 | 0.024 | Bacteria | Proteobacteria | Betaproteobacteria | Neisseriales | Neisseriaceae |  |  | na |
| BD_positive | V736 | 0.783 | 0.032 | 0.158 | 0.037 | Bacteria | Proteobacteria | Alphaproteobacteria | Sphingomonadales | Sphingomonadaceae | *Novosphingobium* | *capsulatum* | na |
| BD_positive | V776 | 0.714 | 0.032 | 0.151 | 0.030 | Bacteria | Proteobacteria | Alphaproteobacteria | Rhizobiales |  |  |  | na |
| BD_positive | V880 | 0.664 | 0.032 | 0.146 | 0.038 | Bacteria | Proteobacteria | Gammaproteobacteria | Thiotrichales | Piscirickettsiaceae |  |  | na |
| BD_positive | V904 | 0.914 | 0.032 | 0.171 | 0.002 | Bacteria | Proteobacteria | Alphaproteobacteria | Rhizobiales | Hyphomicrobiaceae | *Hyphomicrobium* |  | na |
| BD_positive | V930 | 0.922 | 0.032 | 0.172 | 0.003 | Bacteria | Acidobacteria | Acidobacteria-6 | iii1-15 |  |  |  | na |
| BD_positive | V1061 | 0.967 | 0.027 | 0.160 | 0.002 | Bacteria | Proteobacteria | Alphaproteobacteria | Rhodobacterales | Rhodobacteraceae |  |  | inhibitory |
| BD_positive | V1082 | 0.820 | 0.027 | 0.148 | 0.006 | Bacteria | Proteobacteria | Betaproteobacteria | Burkholderiales | Comamonadaceae |  |  | inhibitory |
| BD_positive | V119 | 0.892 | 0.027 | 0.154 | 0.011 | Bacteria | Proteobacteria | Betaproteobacteria | Burkholderiales | Comamonadaceae | *Hydrogenophaga* |  | inhibitory |
| BD_positive | V1227 | 0.946 | 0.027 | 0.159 | 0.007 | Bacteria | Proteobacteria | Alphaproteobacteria | Sphingomonadales | Sphingomonadaceae | *Novosphingobium* |  | inhibitory |
| BD_positive | V1248 | 0.720 | 0.027 | 0.138 | 0.021 | Bacteria | Proteobacteria | Betaproteobacteria | Burkholderiales | Comamonadaceae | *Rubrivivax* |  | inhibitory |
| BD_positive | V1382 | 0.712 | 0.027 | 0.138 | 0.040 | Bacteria | Cyanobacteria | Nostocophycideae | Nostocales | Nostocaceae |  |  | inhibitory |
| BD_positive | V145 | 0.787 | 0.027 | 0.145 | 0.026 | Bacteria | Proteobacteria | Alphaproteobacteria | Rhodobacterales | Rhodobacteraceae | *Rhodobacter* |  | na |
| BD_positive | V1477 | 0.982 | 0.027 | 0.162 | 0.015 | Bacteria | Proteobacteria | Gammaproteobacteria | Pseudomonadales | Pseudomonadaceae | *Pseudomonas* | *umsongensis* | na |
| BD_positive | V1562 | 0.948 | 0.027 | 0.159 | 0.003 | Bacteria | Proteobacteria | Betaproteobacteria | Rhodocyclales | Rhodocyclaceae | *Dok59* |  | na |
| BD_positive | V1585 | 0.975 | 0.027 | 0.161 | 0.002 | Bacteria | Proteobacteria | Gammaproteobacteria | Methylococcales | Methylococcaceae | *Methylocaldum* |  | na |
| BD_positive | V1687 | 0.800 | 0.027 | 0.146 | 0.006 | Bacteria | Proteobacteria | Betaproteobacteria | Burkholderiales | Comamonadaceae |  |  | na |
| BD_positive | V1692 | 0.700 | 0.027 | 0.136 | 0.025 | Bacteria | Actinobacteria | Actinobacteria | Actinomycetales | ACK-M1 |  |  | na |
| BD_positive | V1694 | 0.980 | 0.027 | 0.161 | 0.002 | Bacteria | Proteobacteria | Betaproteobacteria | Rhodocyclales | Rhodocyclaceae | *Uliginosibacterium* |  | na |
| BD_positive | V1747 | 0.823 | 0.027 | 0.148 | 0.026 | Bacteria | Proteobacteria | Gammaproteobacteria | Xanthomonadales | Sinobacteraceae |  |  | na |
| BD_positive | V1863 | 0.914 | 0.027 | 0.156 | 0.010 | Bacteria | Actinobacteria | Thermoleophilia | Gaiellales |  |  |  | na |
| BD_positive | V1968 | 0.957 | 0.027 | 0.160 | 0.003 | Bacteria | Proteobacteria | Epsilonproteobacteria | Campylobacterales | Helicobacteraceae | *Sulfuricurvum* | *kujiense* | na |
| BD_positive | V1992 | 0.865 | 0.027 | 0.152 | 0.014 | Bacteria | Proteobacteria | Betaproteobacteria |  |  |  |  | na |
| BD_positive | V2024 | 0.828 | 0.027 | 0.148 | 0.026 | Bacteria | Proteobacteria | Deltaproteobacteria |  |  |  |  | na |
| BD_positive | V2031 | 1.000 | 0.027 | 0.163 | 0.002 | Bacteria | Bacteroidetes | Bacteroidia | Bacteroidales |  |  |  | na |
| BD_positive | V2142 | 0.820 | 0.027 | 0.148 | 0.006 | Bacteria | Proteobacteria | Deltaproteobacteria | Myxococcales |  |  |  | na |
| BD_positive | V2378 | 0.937 | 0.027 | 0.158 | 0.002 | Bacteria | Proteobacteria | Gammaproteobacteria | Pseudomonadales | Moraxellaceae | *Acinetobacter* | *rhizosphaerae* | na |
| BD_positive | V2388 | 0.951 | 0.027 | 0.159 | 0.002 | Bacteria | Proteobacteria | Betaproteobacteria |  |  |  |  | na |
| BD_positive | V2389 | 0.951 | 0.027 | 0.159 | 0.006 | Archaea | Euryarchaeota | Methanobacteria | Methanobacteriales | Methanobacteriaceae | *Methanobacterium* |  | na |
| BD_positive | V2395 | 0.960 | 0.027 | 0.160 | 0.004 | Bacteria | Acidobacteria | Holophagae | Holophagales | Holophagaceae |  |  | na |
| BD_positive | V2399 | 1.000 | 0.027 | 0.163 | 0.003 | Bacteria | Firmicutes | Clostridia | Clostridiales | Clostridiaceae | *Clostridium* |  | na |
| BD_positive | V2401 | 0.922 | 0.027 | 0.157 | 0.004 | Bacteria | Proteobacteria | Deltaproteobacteria | Spirobacillales |  |  |  | na |
| BD_positive | V2408 | 0.828 | 0.027 | 0.148 | 0.005 | Bacteria | Proteobacteria | Deltaproteobacteria | *Bd*ellovibrionales | *Bd*ellovibrionaceae | *Bdellovibrio* |  | na |
| BD_positive | V2409 | 1.000 | 0.027 | 0.163 | 0.001 | Bacteria | Proteobacteria | Gammaproteobacteria | Methylococcales | Methylococcaceae | *Methylomonas* |  | na |
| BD_positive | V2442 | 1.000 | 0.027 | 0.163 | 0.002 | Bacteria | Proteobacteria | Epsilonproteobacteria | Campylobacterales | Campylobacteraceae |  |  | na |
| BD_positive | V2450 | 0.858 | 0.027 | 0.151 | 0.008 | Bacteria | Proteobacteria | Alphaproteobacteria | Rickettsiales |  |  |  | na |
| BD_positive | V278 | 0.979 | 0.027 | 0.161 | 0.046 | Bacteria | Proteobacteria | Gammaproteobacteria | Pseudomonadales | Moraxellaceae | *Acinetobacter* |  | na |
| BD_positive | V280 | 0.807 | 0.027 | 0.146 | 0.022 | Bacteria | Bacteroidetes | Bacteroidia | Bacteroidales | Porphyromonadaceae |  |  | na |
| BD_positive | V408 | 0.733 | 0.027 | 0.140 | 0.039 | Bacteria | Bacteroidetes | Bacteroidia | Bacteroidales | Bacteroidaceae | *Bacteroides* |  | na |
| BD_positive | V445 | 0.828 | 0.027 | 0.148 | 0.031 | Bacteria | Firmicutes | Clostridia | Clostridiales | Lachnospiraceae |  |  | na |
| BD_positive | V461 | 0.865 | 0.027 | 0.152 | 0.003 | Bacteria | Proteobacteria | Betaproteobacteria | Rhodocyclales | Rhodocyclaceae | *Zoogloea* |  | na |
| BD_positive | V606 | 0.724 | 0.027 | 0.139 | 0.025 | Bacteria | Actinobacteria | Actinobacteria | Actinomycetales | Promicromonosporaceae |  |  | na |
| BD_positive | V674 | 0.876 | 0.027 | 0.153 | 0.017 | Bacteria | Proteobacteria | Betaproteobacteria | Burkholderiales | Oxalobacteraceae |  |  | na |
| BD_positive | V691 | 0.878 | 0.027 | 0.153 | 0.028 | Bacteria | Proteobacteria | Gammaproteobacteria | Pseudomonadales | Moraxellaceae |  |  | na |
| BD_positive | V701 | 0.821 | 0.027 | 0.148 | 0.012 | Bacteria | Proteobacteria | Alphaproteobacteria | Sphingomonadales | Sphingomonadaceae |  |  | na |
| BD_positive | V722 | 1.000 | 0.027 | 0.163 | 0.001 | Bacteria | Firmicutes | Bacilli | Bacillales | Bacillaceae | *Bacillus* | *thermoamylovorans* | na |
| BD_positive | V743 | 0.995 | 0.027 | 0.163 | 0.001 | Bacteria | Bacteroidetes | Bacteroidia | Bacteroidales | Bacteroidaceae | *Bacteroides* |  | na |
| BD_positive | V76 | 0.977 | 0.027 | 0.161 | 0.042 | Bacteria | Proteobacteria | Gammaproteobacteria | Xanthomonadales | Xanthomonadaceae | *Stenotrophomonas* | *acidaminiphila* | na |
| BD_positive | V765 | 0.933 | 0.027 | 0.158 | 0.006 | Bacteria | Proteobacteria | Betaproteobacteria | Burkholderiales |  |  |  | na |
| BD_positive | V77 | 1.000 | 0.027 | 0.163 | 0.001 | Bacteria | Proteobacteria | Betaproteobacteria | Rhodocyclales | Rhodocyclaceae | *Dechloromonas* | *fungiphilus* | na |
| BD_positive | V925 | 0.865 | 0.027 | 0.152 | 0.021 | Bacteria | Bacteroidetes | [Saprospirae] | [Saprospirales] | Saprospiraceae | *Haliscomenobacter* |  | na |
| BD_positive | V976 | 0.822 | 0.027 | 0.148 | 0.033 | Bacteria | Proteobacteria | Betaproteobacteria |  |  |  |  | na |
| BD_positive | V1001 | 0.898 | 0.021 | 0.138 | 0.022 | Bacteria | Acidobacteria | [Chloracidobacteria] | RB41 | Ellin6075 |  |  | inhibitory |
| BD_positive | V1084 | 0.846 | 0.021 | 0.134 | 0.032 | Bacteria | Proteobacteria | Deltaproteobacteria | Syntrophobacterales | Syntrophobacteraceae |  |  | inhibitory |
| BD_positive | V1210 | 0.951 | 0.021 | 0.142 | 0.035 | Bacteria | Proteobacteria | Gammaproteobacteria | Pseudomonadales | Pseudomonadaceae | *Pseudomonas* |  | inhibitory |
| BD_positive | V1261 | 0.964 | 0.021 | 0.143 | 0.009 | Bacteria | Proteobacteria | Betaproteobacteria | Burkholderiales | Comamonadaceae | *Rubrivivax* |  | inhibitory |
| BD_positive | V1273 | 0.935 | 0.021 | 0.141 | 0.008 | Bacteria | Actinobacteria | Actinobacteria | Actinomycetales | Micrococcaceae |  |  | inhibitory |
| BD_positive | V1448 | 0.889 | 0.021 | 0.138 | 0.017 | Bacteria | Firmicutes | Clostridia | Clostridiales | Clostridiaceae | *SMB53* |  | inhibitory |
| BD_positive | V1519 | 0.696 | 0.021 | 0.122 | 0.040 | Bacteria | Proteobacteria | Alphaproteobacteria | Rhodospirillales | Acetobacteraceae |  |  | inhibitory |
| BD_positive | V1569 | 0.976 | 0.021 | 0.144 | 0.032 | Bacteria | Bacteroidetes | Cytophagia | Cytophagales | Cytophagaceae | *Larkinella* |  | inhibitory |
| BD_positive | V163 | 0.822 | 0.021 | 0.132 | 0.041 | Bacteria | Firmicutes | Erysipelotrichi | Erysipelotrichales | Erysipelotrichaceae | *[Eubacterium]* |  | na |
| BD_positive | V1707 | 0.946 | 0.021 | 0.142 | 0.032 | Bacteria | Proteobacteria | Betaproteobacteria | Burkholderiales |  |  |  | na |
| BD_positive | V1711 | 0.842 | 0.021 | 0.134 | 0.033 | Bacteria | Bacteroidetes | Cytophagia | Cytophagales | Cytophagaceae |  |  | na |
| BD_positive | V1818 | 0.855 | 0.021 | 0.135 | 0.029 | Bacteria | Proteobacteria | Alphaproteobacteria | Rhodobacterales | Rhodobacteraceae | *Rhodobacter* |  | na |
| BD_positive | V1841 | 0.962 | 0.021 | 0.143 | 0.008 | Bacteria | Acidobacteria | Acidobacteriia | Acidobacteriales | Acidobacteriaceae |  |  | na |
| BD_positive | V1882 | 1.000 | 0.021 | 0.146 | 0.003 | Bacteria | Proteobacteria | Deltaproteobacteria | Myxococcales |  |  |  | na |
| BD_positive | V1891 | 1.000 | 0.021 | 0.146 | 0.004 | Bacteria | Proteobacteria | Gammaproteobacteria | Methylococcales | Methylococcaceae | *Methylocaldum* |  | na |
| BD_positive | V1897 | 0.977 | 0.021 | 0.144 | 0.006 | Bacteria | Proteobacteria | Gammaproteobacteria | Methylococcales | Methylococcaceae |  |  | na |
| BD_positive | V1900 | 0.957 | 0.021 | 0.143 | 0.011 | Bacteria | Proteobacteria | Deltaproteobacteria | Desulfuromonadales | Geobacteraceae | *Geobacter* |  | na |
| BD_positive | V1904 | 0.937 | 0.021 | 0.141 | 0.008 | Bacteria | Bacteroidetes | Bacteroidia | Bacteroidales | Porphyromonadaceae | *Parabacteroides* |  | na |
| BD_positive | V1917 | 0.916 | 0.021 | 0.140 | 0.029 | Bacteria | Actinobacteria | Actinobacteria | Actinomycetales | Microbacteriaceae | *Cryocola* |  | na |
| BD_positive | V213 | 0.762 | 0.021 | 0.127 | 0.034 | Bacteria | Proteobacteria | Deltaproteobacteria | Desulfovibrionales | Desulfovibrionaceae | *Desulfovibrio* |  | na |
| BD_positive | V2147 | 0.789 | 0.021 | 0.130 | 0.027 | Bacteria | Proteobacteria | Deltaproteobacteria | Myxococcales |  |  |  | na |
| BD_positive | V2246 | 1.000 | 0.021 | 0.146 | 0.002 | Bacteria | WS3 | PRR-12 | Sediment-1 |  |  |  | na |
| BD_positive | V2308 | 1.000 | 0.021 | 0.146 | 0.006 | Bacteria | Proteobacteria | Alphaproteobacteria | Rhodobacterales | Rhodobacteraceae | *Paracoccus* |  | na |
| BD_positive | V2309 | 0.762 | 0.021 | 0.127 | 0.018 | Bacteria | Actinobacteria | Rubrobacteria | Rubrobacterales | Rubrobacteraceae | *Rubrobacter* |  | na |
| BD_positive | V2330 | 1.000 | 0.021 | 0.146 | 0.005 | Bacteria | Chloroflexi | Anaerolineae | CFB-26 |  |  |  | na |
| BD_positive | V2396 | 0.880 | 0.021 | 0.137 | 0.026 | Bacteria | Planctomycetes | vadinHA49 | DH61 |  |  |  | na |
| BD_positive | V2410 | 0.762 | 0.021 | 0.127 | 0.029 | Bacteria | Firmicutes | Erysipelotrichi | Erysipelotrichales | Erysipelotrichaceae | *Clostridium* |  | na |
| BD_positive | V2417 | 0.906 | 0.021 | 0.139 | 0.011 | Bacteria | Planctomycetes | OM190 | CL500-15 |  |  |  | na |
| BD_positive | V2418 | 1.000 | 0.021 | 0.146 | 0.005 | Bacteria | Verrucomicrobia | Verrucomicrobiae | Verrucomicrobiales | Verrucomicrobiaceae | *Prosthecobacter* | *debontii* | na |
| BD_positive | V2422 | 1.000 | 0.021 | 0.146 | 0.004 | Bacteria | Verrucomicrobia | Verruco-5 | WCHB1-41 | RFP12 |  |  | na |
| BD_positive | V2439 | 1.000 | 0.021 | 0.146 | 0.004 | Bacteria | Proteobacteria | Alphaproteobacteria | Sphingomonadales | Sphingomonadaceae | *Sphingomonas* |  | na |
| BD_positive | V2454 | 0.855 | 0.021 | 0.135 | 0.027 | Bacteria | Firmicutes | Clostridia | Clostridiales | Veillonellaceae | *Succinispira* | *mobilis* | na |
| BD_positive | V2474 | 1.000 | 0.021 | 0.146 | 0.003 | Bacteria | Proteobacteria | Gammaproteobacteria | Xanthomonadales | Sinobacteraceae |  |  | na |
| BD_positive | V25 | 0.925 | 0.021 | 0.140 | 0.014 | Bacteria | Proteobacteria | Epsilonproteobacteria | Campylobacterales | Campylobacteraceae | *Arcobacter* | *cryaerophilus* | na |
| BD_positive | V366 | 0.906 | 0.021 | 0.139 | 0.026 | Bacteria | Bacteroidetes | Bacteroidia | Bacteroidales | Porphyromonadaceae | *Parabacteroides* |  | na |
| BD_positive | V419 | 0.919 | 0.021 | 0.140 | 0.032 | Bacteria | Firmicutes | Clostridia | Clostridiales | Lachnospiraceae | *Clostridium* |  | na |
| BD_positive | V46 | 0.937 | 0.021 | 0.141 | 0.012 | Bacteria | Proteobacteria | Betaproteobacteria | Rhodocyclales | Rhodocyclaceae | *Zoogloea* |  | na |
| BD_positive | V516 | 0.989 | 0.021 | 0.145 | 0.005 | Bacteria | Proteobacteria | Betaproteobacteria | Burkholderiales | Comamonadaceae | *Methylibium* |  | na |
| BD_positive | V617 | 0.923 | 0.021 | 0.140 | 0.039 | Bacteria | Actinobacteria | Actinobacteria | Actinomycetales | Nocardiaceae | *Rhodococcus* |  | na |
| BD_positive | V660 | 0.953 | 0.021 | 0.142 | 0.020 | Bacteria | Proteobacteria | Alphaproteobacteria | Sphingomonadales | Sphingomonadaceae | *Novosphingobium* |  | na |
| BD_positive | V746 | 1.000 | 0.021 | 0.146 | 0.007 | Bacteria | Bacteroidetes | Bacteroidia | Bacteroidales | Bacteroidaceae | *Bacteroides* |  | na |
| BD_positive | V866 | 0.786 | 0.021 | 0.129 | 0.036 | Bacteria | Proteobacteria | Betaproteobacteria | Burkholderiales | Comamonadaceae |  |  | na |
| BD_positive | V872 | 0.733 | 0.021 | 0.125 | 0.029 | Bacteria | Acidobacteria | Acidobacteria-6 | iii1-15 |  |  |  | na |
| BD_positive | V952 | 0.967 | 0.021 | 0.143 | 0.015 | Bacteria | Proteobacteria | Betaproteobacteria | Burkholderiales | Comamonadaceae |  |  | na |
| BD_positive | V958 | 0.895 | 0.021 | 0.138 | 0.025 | Bacteria | Verrucomicrobia | [Spartobacteria] | [Chthoniobacterales] | [Chthoniobacteraceae] |  |  | na |
| BD_positive | V982 | 0.762 | 0.021 | 0.127 | 0.048 | Bacteria | Bacteroidetes | Flavobacteriia | Flavobacteriales | [Weeksellaceae] | *Chryseobacterium* |  | na |
| BD_positive | V996 | 0.792 | 0.021 | 0.130 | 0.035 | Bacteria | Proteobacteria | Gammaproteobacteria | Xanthomonadales | Xanthomonadaceae | *Stenotrophomonas* |  | na |
| BD_positive | V1007 | 1.000 | 0.016 | 0.126 | 0.011 | Bacteria | Bacteroidetes | Bacteroidia | Bacteroidales | Prevotellaceae | *Prevotella* | *melaninogenica* | inhibitory |
| BD_positive | V1045 | 0.991 | 0.016 | 0.126 | 0.039 | Bacteria | Bacteroidetes | [Saprospirae] | [Saprospirales] | Chitinophagaceae | *Chitinophaga* |  | inhibitory |
| BD_positive | V1046 | 0.941 | 0.016 | 0.123 | 0.030 | Bacteria | Proteobacteria | Betaproteobacteria | Burkholderiales | Comamonadaceae |  |  | inhibitory |
| BD_positive | V105 | 0.932 | 0.016 | 0.122 | 0.031 | Bacteria | Proteobacteria | Alphaproteobacteria | Rhodospirillales | Rhodospirillaceae | *Magnetospirillum* |  | inhibitory |
| BD_positive | V1160 | 0.889 | 0.016 | 0.119 | 0.042 | Bacteria | Proteobacteria | Betaproteobacteria | Burkholderiales | Comamonadaceae | *Variovorax* | *paradoxus* | inhibitory |
| BD_positive | V1303 | 0.970 | 0.016 | 0.124 | 0.030 | Bacteria | Proteobacteria | Alphaproteobacteria | Rhizobiales | Rhizobiaceae |  |  | inhibitory |
| BD_positive | V1317 | 0.800 | 0.016 | 0.113 | 0.049 | Bacteria | Actinobacteria | Actinobacteria | Actinomycetales |  |  |  | inhibitory |
| BD_positive | V1431 | 0.928 | 0.016 | 0.122 | 0.031 | Bacteria | Firmicutes | Bacilli | Bacillales | Planococcaceae | *Paenisporosarcina* |  | inhibitory |
| BD_positive | V1643 | 0.993 | 0.016 | 0.126 | 0.039 | Bacteria | Proteobacteria | Alphaproteobacteria | Rhodospirillales | Acetobacteraceae |  |  | inhibitory |
| BD_positive | V1701 | 1.000 | 0.016 | 0.126 | 0.010 | Bacteria | Proteobacteria | Betaproteobacteria | SC-I-84 |  |  |  | inhibitory |
| BD_positive | V1729 | 1.000 | 0.016 | 0.126 | 0.017 | Bacteria | Proteobacteria | Gammaproteobacteria | Xanthomonadales | Sinobacteraceae | *Nevskia* | *ramosa* | na |
| BD_positive | V1744 | 0.706 | 0.016 | 0.106 | 0.041 | Bacteria | Planctomycetes | Planctomycetia | Gemmatales | Gemmataceae | *Gemmata* |  | na |
| BD_positive | V1849 | 0.967 | 0.016 | 0.124 | 0.041 | Bacteria | Proteobacteria | Betaproteobacteria | Burkholderiales | Comamonadaceae |  |  | na |
| BD_positive | V1850 | 1.000 | 0.016 | 0.126 | 0.014 | Bacteria | Proteobacteria | Betaproteobacteria | Burkholderiales | Comamonadaceae | *Rubrivivax* |  | na |
| BD_positive | V1867 | 0.967 | 0.016 | 0.124 | 0.026 | Bacteria | Proteobacteria | Deltaproteobacteria | Myxococcales |  |  |  | na |
| BD_positive | V1883 | 0.972 | 0.016 | 0.125 | 0.028 | Bacteria | Proteobacteria | Deltaproteobacteria | Myxococcales | Myxococcaceae | *Anaeromyxobacter* |  | na |
| BD_positive | V1903 | 0.967 | 0.016 | 0.124 | 0.029 | Bacteria | Actinobacteria | Actinobacteria | Actinomycetales | Microbacteriaceae | *Mycetocola* |  | na |
| BD_positive | V2028 | 0.798 | 0.016 | 0.113 | 0.037 | Bacteria | Bacteroidetes | Bacteroidia | Bacteroidales | Porphyromonadaceae | *Parabacteroides* | *gordonii* | na |
| BD_positive | V2040 | 1.000 | 0.016 | 0.126 | 0.016 | Bacteria | Bacteroidetes | Bacteroidia | Bacteroidales | Bacteroidaceae | *Bacteroides* |  | na |
| BD_positive | V2055 | 1.000 | 0.016 | 0.126 | 0.017 | Bacteria | Proteobacteria | Deltaproteobacteria | Myxococcales |  |  |  | na |
| BD_positive | V2086 | 1.000 | 0.016 | 0.126 | 0.014 | Bacteria | Proteobacteria | Betaproteobacteria | Burkholderiales | Comamonadaceae |  |  | na |
| BD_positive | V2090 | 0.918 | 0.016 | 0.121 | 0.037 | Bacteria | Bacteroidetes | Cytophagia | Cytophagales | Cytophagaceae |  |  | na |
| BD_positive | V2131 | 0.928 | 0.016 | 0.122 | 0.032 | Bacteria | Proteobacteria | Alphaproteobacteria | Rhodobacterales | Rhodobacteraceae | *Paracoccus* |  | na |
| BD_positive | V2205 | 1.000 | 0.016 | 0.126 | 0.014 | Bacteria | Actinobacteria | Actinobacteria |  |  |  |  | na |
| BD_positive | V2220 | 1.000 | 0.016 | 0.126 | 0.017 | Bacteria | Chloroflexi | Thermomicrobia | JG30-KF-CM45 |  |  |  | na |
| BD_positive | V2254 | 0.985 | 0.016 | 0.125 | 0.027 | Bacteria | Firmicutes | Bacilli | Lactobacillales | Streptococcaceae | *Lactococcus* | *garvieae* | na |
| BD_positive | V2260 | 1.000 | 0.016 | 0.126 | 0.011 | Bacteria | Bacteroidetes | Sphingobacteriia | Sphingobacteriales | Sphingobacteriaceae | *Pedobacter* |  | na |
| BD_positive | V2265 | 0.972 | 0.016 | 0.125 | 0.024 | Bacteria | Proteobacteria | Alphaproteobacteria | Rhodobacterales | Rhodobacteraceae | *Paracoccus* |  | na |
| BD_positive | V2290 | 0.906 | 0.016 | 0.120 | 0.049 | Bacteria | Acidobacteria | RB25 |  |  |  |  | na |
| BD_positive | V2392 | 0.941 | 0.016 | 0.123 | 0.023 | Bacteria | Firmicutes | Clostridia | OPB54 |  |  |  | na |
| BD_positive | V2407 | 0.967 | 0.016 | 0.124 | 0.039 | Bacteria | Proteobacteria | Gammaproteobacteria | Methylococcales | Crenotrichaceae | *Crenothrix* |  | na |
| BD_positive | V2411 | 1.000 | 0.016 | 0.126 | 0.011 | Bacteria | Proteobacteria | Deltaproteobacteria | Myxococcales | Polyangiaceae |  |  | na |
| BD_positive | V2415 | 1.000 | 0.016 | 0.126 | 0.010 | Bacteria | Proteobacteria | Deltaproteobacteria | Myxococcales |  |  |  | na |
| BD_positive | V2416 | 1.000 | 0.016 | 0.126 | 0.014 | Bacteria | Proteobacteria | Gammaproteobacteria | Methylococcales | Methylococcaceae |  |  | na |
| BD_positive | V2429 | 0.967 | 0.016 | 0.124 | 0.025 | Bacteria | Gemmatimonadetes | Gemmatimonadetes |  |  |  |  | na |
| BD_positive | V2432 | 0.957 | 0.016 | 0.124 | 0.029 | Bacteria | Gemmatimonadetes | Gemmatimonadetes |  |  |  |  | na |
| BD_positive | V2433 | 0.951 | 0.016 | 0.123 | 0.033 | Bacteria | Firmicutes | Clostridia | Clostridiales | Eubacteriaceae | *Pseudoramibacter_Eubacterium* |  | na |
| BD_positive | V2436 | 1.000 | 0.016 | 0.126 | 0.016 | Bacteria | Proteobacteria | Epsilonproteobacteria | Campylobacterales | Helicobacteraceae | *Sulfuricurvum* | *kujiense* | na |
| BD_positive | V2437 | 1.000 | 0.016 | 0.126 | 0.015 | Bacteria | Actinobacteria | Actinobacteria | Actinomycetales | Cellulomonadaceae | *Cellulomonas* |  | na |
| BD_positive | V2438 | 1.000 | 0.016 | 0.126 | 0.013 | Bacteria | Bacteroidetes | Bacteroidia | Bacteroidales | Porphyromonadaceae | *Paludibacter* |  | na |
| BD_positive | V2443 | 1.000 | 0.016 | 0.126 | 0.018 | Bacteria | Bacteroidetes | Flavobacteriia | Flavobacteriales | Flavobacteriaceae | *Flavobacterium* |  | na |
| BD_positive | V2444 | 0.967 | 0.016 | 0.124 | 0.016 | Bacteria | Firmicutes | Clostridia | Clostridiales | Clostridiaceae | *Clostridium* |  | na |
| BD_positive | V2448 | 0.951 | 0.016 | 0.123 | 0.032 | Bacteria | Proteobacteria | Alphaproteobacteria | Rhizobiales | Methylocystaceae |  |  | na |
| BD_positive | V2455 | 0.876 | 0.016 | 0.118 | 0.041 | Bacteria | Proteobacteria | Deltaproteobacteria | *Bd*ellovibrionales | Bacteriovoracaceae |  |  | na |
| BD_positive | V2459 | 0.977 | 0.016 | 0.125 | 0.024 | Bacteria | Proteobacteria | Alphaproteobacteria | Rhizobiales | Methylocystaceae |  |  | na |
| BD_positive | V2462 | 0.935 | 0.016 | 0.122 | 0.032 | Bacteria | Proteobacteria | Alphaproteobacteria | Rickettsiales | Rickettsiaceae |  |  | na |
| BD_positive | V2467 | 0.658 | 0.016 | 0.102 | 0.045 | Bacteria | Proteobacteria | Alphaproteobacteria | Rhizobiales | Rhizobiaceae |  |  | na |
| BD_positive | V2472 | 1.000 | 0.016 | 0.126 | 0.018 | Bacteria | Actinobacteria | Actinobacteria | Actinomycetales |  |  |  | na |
| BD_positive | V2473 | 1.000 | 0.016 | 0.126 | 0.014 | Bacteria | Proteobacteria | Betaproteobacteria |  |  |  |  | na |
| BD_positive | V2476 | 1.000 | 0.016 | 0.126 | 0.019 | Bacteria | Acidobacteria | Acidobacteria-6 | iii1-15 |  |  |  | na |
| BD_positive | V2501 | 1.000 | 0.016 | 0.126 | 0.016 | Bacteria | Proteobacteria | Betaproteobacteria | Burkholderiales |  |  |  | na |
| BD_positive | V2509 | 1.000 | 0.016 | 0.126 | 0.015 | Bacteria | Actinobacteria | Actinobacteria | Actinomycetales | ACK-M1 |  |  | na |
| BD_positive | V2519 | 1.000 | 0.016 | 0.126 | 0.020 | Bacteria | Actinobacteria | Actinobacteria | Actinomycetales | Frankiaceae |  |  | na |
| BD_positive | V294 | 1.000 | 0.016 | 0.126 | 0.014 | Bacteria | Firmicutes | Clostridia | Clostridiales | Clostridiaceae | *Clostridium* | *intestinale* | na |
| BD_positive | V300 | 1.000 | 0.016 | 0.126 | 0.009 | Bacteria |  |  |  |  |  |  | na |
| BD_positive | V342 | 0.961 | 0.016 | 0.124 | 0.030 | Bacteria | Firmicutes | Bacilli | Bacillales | Staphylococcaceae | *Staphylococcus* | *equorum* | na |
| BD_positive | V436 | 0.797 | 0.016 | 0.113 | 0.047 | Bacteria | Firmicutes | Clostridia | Clostridiales | Ruminococcaceae | *Oscillospira* |  | na |
| BD_positive | V437 | 0.918 | 0.016 | 0.121 | 0.049 | Bacteria | Proteobacteria | Deltaproteobacteria | Desulfovibrionales | Desulfovibrionaceae | *Bilophila* |  | na |
| BD_positive | V440 | 0.981 | 0.016 | 0.125 | 0.024 | Bacteria | Firmicutes | Clostridia | Clostridiales | Eubacteriaceae | *Pseudoramibacter_Eubacterium* |  | na |
| BD_positive | V468 | 0.966 | 0.016 | 0.124 | 0.048 | Bacteria | Firmicutes | Clostridia | Clostridiales | Ruminococcaceae | *Oscillospira* |  | na |
| BD_positive | V546 | 0.988 | 0.016 | 0.126 | 0.021 | Bacteria | Proteobacteria | Betaproteobacteria | Burkholderiales | Comamonadaceae |  |  | na |
| BD_positive | V601 | 1.000 | 0.016 | 0.126 | 0.009 | Bacteria | Actinobacteria | Actinobacteria | Actinomycetales | Intrasporangiaceae |  |  | na |
| BD_positive | V616 | 0.988 | 0.016 | 0.126 | 0.042 | Bacteria | Proteobacteria | Gammaproteobacteria | Pasteurellales | Pasteurellaceae | *Aggregatibacter* | *segnis* | na |
| BD_positive | V64 | 1.000 | 0.016 | 0.126 | 0.019 | Bacteria | Proteobacteria | Betaproteobacteria | Burkholderiales | Comamonadaceae |  |  | na |
| BD_positive | V650 | 0.946 | 0.016 | 0.123 | 0.032 | Bacteria | Proteobacteria | Betaproteobacteria | Burkholderiales | Comamonadaceae | *Rubrivivax* |  | na |
| BD_positive | V663 | 0.931 | 0.016 | 0.122 | 0.026 | Bacteria | Proteobacteria | Betaproteobacteria | Neisseriales | Neisseriaceae |  |  | na |
| BD_positive | V740 | 0.984 | 0.016 | 0.125 | 0.020 | Bacteria | Firmicutes | Erysipelotrichi | Erysipelotrichales | Erysipelotrichaceae | *Anaerorhabdus* | *furcosa* | na |
| BD_positive | V742 | 0.965 | 0.016 | 0.124 | 0.022 | Bacteria | Bacteroidetes | Bacteroidia | Bacteroidales | Porphyromonadaceae | *Parabacteroides* |  | na |
| BD_positive | V767 | 0.865 | 0.016 | 0.117 | 0.043 | Bacteria | Firmicutes | Clostridia | Clostridiales | Lachnospiraceae |  |  | na |
| BD_positive | V770 | 1.000 | 0.016 | 0.126 | 0.014 | Bacteria | Actinobacteria | Actinobacteria | Actinomycetales | Mycobacteriaceae | *Mycobacterium* |  | na |
| BD_positive | V775 | 0.946 | 0.016 | 0.123 | 0.050 | Bacteria | Proteobacteria | Betaproteobacteria | Rhodocyclales | Rhodocyclaceae | *Uliginosibacterium* |  | na |
| BD_positive | V781 | 0.967 | 0.016 | 0.124 | 0.021 | Bacteria | Bacteroidetes | Bacteroidia | Bacteroidales | Bacteroidaceae | *Bacteroides* |  | na |
| BD_positive | V794 | 0.972 | 0.016 | 0.125 | 0.039 | Bacteria | Bacteroidetes | Bacteroidia | Bacteroidales | Bacteroidaceae | *Bacteroides* | *caccae* | na |
| BD_positive | V80 | 1.000 | 0.016 | 0.126 | 0.013 | Bacteria | Proteobacteria | Gammaproteobacteria | Enterobacteriales | Enterobacteriaceae |  |  | na |
| BD_positive | V924 | 0.982 | 0.016 | 0.125 | 0.034 | Bacteria | Acidobacteria | Acidobacteria-6 | iii1-15 |  |  |  | na |
| BD_positive | V950 | 0.906 | 0.016 | 0.120 | 0.046 | Bacteria | Proteobacteria | Deltaproteobacteria | Myxococcales |  |  |  | na |
| BD_positive | V1438 | 1.000 | 0.011 | 0.103 | 0.043 | Bacteria | Bacteroidetes | Flavobacteriia | Flavobacteriales | [Weeksellaceae] | *Chryseobacterium* |  | na |
| BD_positive | V1770 | 1.000 | 0.011 | 0.103 | 0.042 | Bacteria | Proteobacteria | Alphaproteobacteria | Rhizobiales |  |  |  | na |
| BD_positive | V1945 | 1.000 | 0.011 | 0.103 | 0.048 | Bacteria | Acidobacteria | Acidobacteriia | Acidobacteriales | Acidobacteriaceae |  |  | na |
| BD_positive | V2079 | 1.000 | 0.011 | 0.103 | 0.047 | Bacteria | Firmicutes | Clostridia | Clostridiales |  |  |  | na |
| BD_positive | V2381 | 1.000 | 0.011 | 0.103 | 0.049 | Bacteria | Proteobacteria | Betaproteobacteria | Burkholderiales | Alcaligenaceae | *Pigmentiphaga* |  | na |
| BD_positive | V677 | 0.957 | 0.011 | 0.101 | 0.050 | Bacteria | Proteobacteria | Alphaproteobacteria | Sphingomonadales | Sphingomonadaceae |  |  | na |
| BD_positive | V757 | 1.000 | 0.011 | 0.103 | 0.047 | Bacteria | Proteobacteria | Deltaproteobacteria | Myxococcales | Myxococcaceae | *Anaeromyxobacter* |  | na |
| BD_negative | V620 | 0.916 | 0.201 | 0.429 | 0.001 | Bacteria | Proteobacteria | Gammaproteobacteria | Pseudomonadales | Pseudomonadaceae | *Pseudomonas* |  | inhibitory |
| BD_negative | V160 | 0.966 | 0.181 | 0.418 | 0.001 | Bacteria | Proteobacteria | Gammaproteobacteria | Oceanospirillales | Halomonadaceae | *Halomonas* |  | na |
| BD_negative | V240 | 0.969 | 0.174 | 0.411 | 0.001 | Bacteria | Firmicutes | Erysipelotrichi | Erysipelotrichales | Erysipelotrichaceae | *[Eubacterium]* | *dolichum* | na |
| BD_negative | V1555 | 0.988 | 0.114 | 0.336 | 0.002 | Bacteria | Firmicutes | Bacilli | Bacillales | Listeriaceae | *Brochothrix* |  | na |
| BD_negative | V183 | 0.987 | 0.103 | 0.319 | 0.001 | Bacteria | Proteobacteria | Gammaproteobacteria | Vibrionales | Vibrionaceae | *Vibrio* | *rumoiensis* | na |
| BD_negative | V15 | 0.996 | 0.068 | 0.260 | 0.016 | Bacteria | Bacteroidetes | Flavobacteriia | Flavobacteriales | [Weeksellaceae] | *Chryseobacterium* |  | inhibitory |
| BD_negative | V1006 | 0.911 | 0.065 | 0.243 | 0.026 | Bacteria | Proteobacteria | Betaproteobacteria | Burkholderiales | Comamonadaceae | *Diaphorobacter* |  | inhibitory |
| BD_negative | V1494 | 0.984 | 0.063 | 0.249 | 0.029 | Bacteria | Proteobacteria | Betaproteobacteria | Burkholderiales | Burkholderiaceae | *Burkholderia* |  | inhibitory |
| BD_negative | V483 | 0.982 | 0.058 | 0.239 | 0.038 | Bacteria | Firmicutes | Bacilli | Lactobacillales | Aerococcaceae | *Alloiococcus* |  | na |
| BD_negative | V845 | 0.878 | 0.053 | 0.216 | 0.044 | Bacteria | Actinobacteria | Actinobacteria | Actinomycetales | Microbacteriaceae | *Yonghaparkia* |  | inhibitory |
| BD_negative | V1348 | 0.932 | 0.053 | 0.222 | 0.033 | Bacteria | Bacteroidetes | Sphingobacteriia | Sphingobacteriales | Sphingobacteriaceae | *Sphingobacterium* |  | inhibitory |
| BD_negative | V1358 | 1.000 | 0.050 | 0.223 | 0.011 | Bacteria | Actinobacteria | Actinobacteria | Actinomycetales | Corynebacteriaceae | *Corynebacterium* |  | na |
| BD_negative | V1124 | 0.920 | 0.043 | 0.199 | 0.047 | Bacteria | Acidobacteria | Acidobacteriia | Acidobacteriales | Acidobacteriaceae | *Terriglobus* |  | na |
| BD_negative | V263 | 1.000 | 0.032 | 0.178 | 0.034 | Bacteria | Actinobacteria | Actinobacteria | Actinomycetales | Dietziaceae |  |  | inhibitory |
| BD_negative | V577 | 1.000 | 0.032 | 0.178 | 0.038 | Bacteria | Proteobacteria | Alphaproteobacteria | Caulobacterales | Caulobacteraceae |  |  | inhibitory |


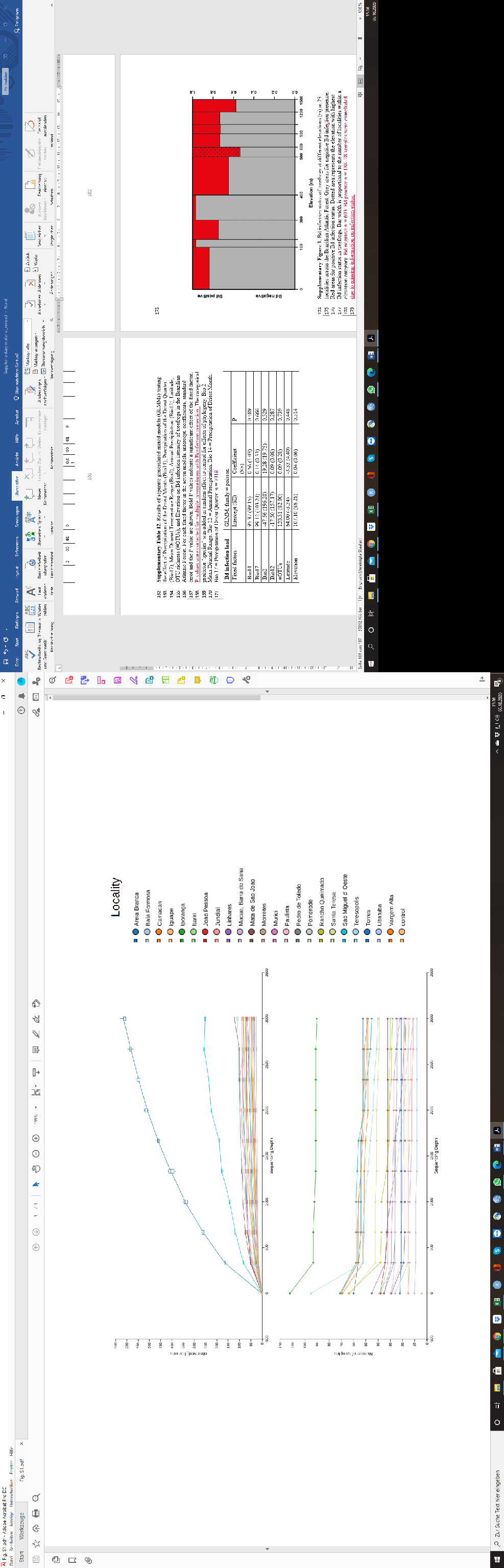
**Supplementary Figure 1.** Multiple rarefaction curve.


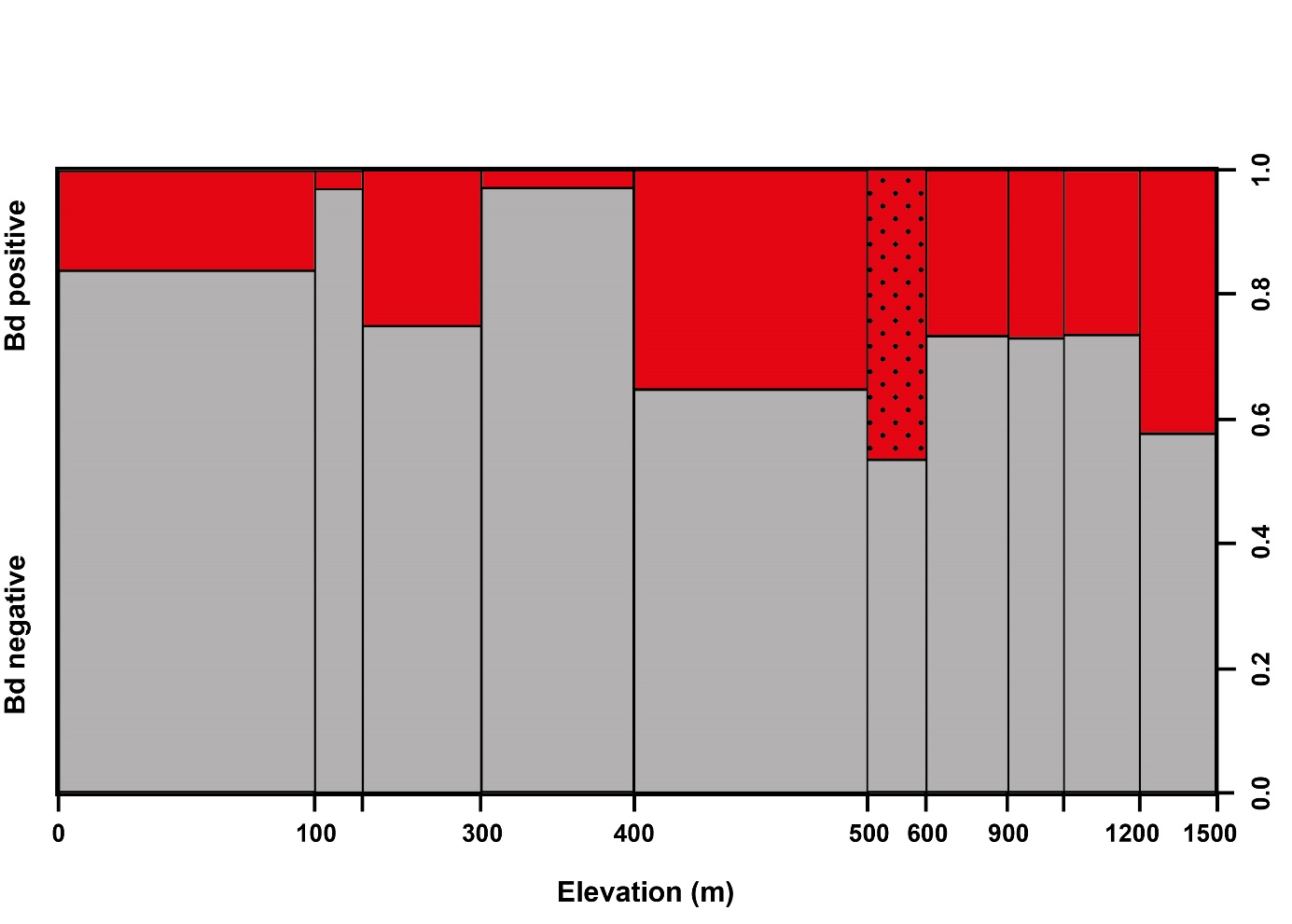
**Supplementary Figure 2.** Bd infection status of treefrogs at different elevations (m) at 23 localities across the Brazilian Atlantic Forest. Grey areas for negative Bd infection presence. Red areas for positive Bd infection status. Dotted area represents the elevation with highest Bd infection status in treefrogs. Bar width is proportional to the number of localities within a elevation category. Bd negative N = 603. Bd positive N = 188. 28 samples were excluded due to missing information on infection status.


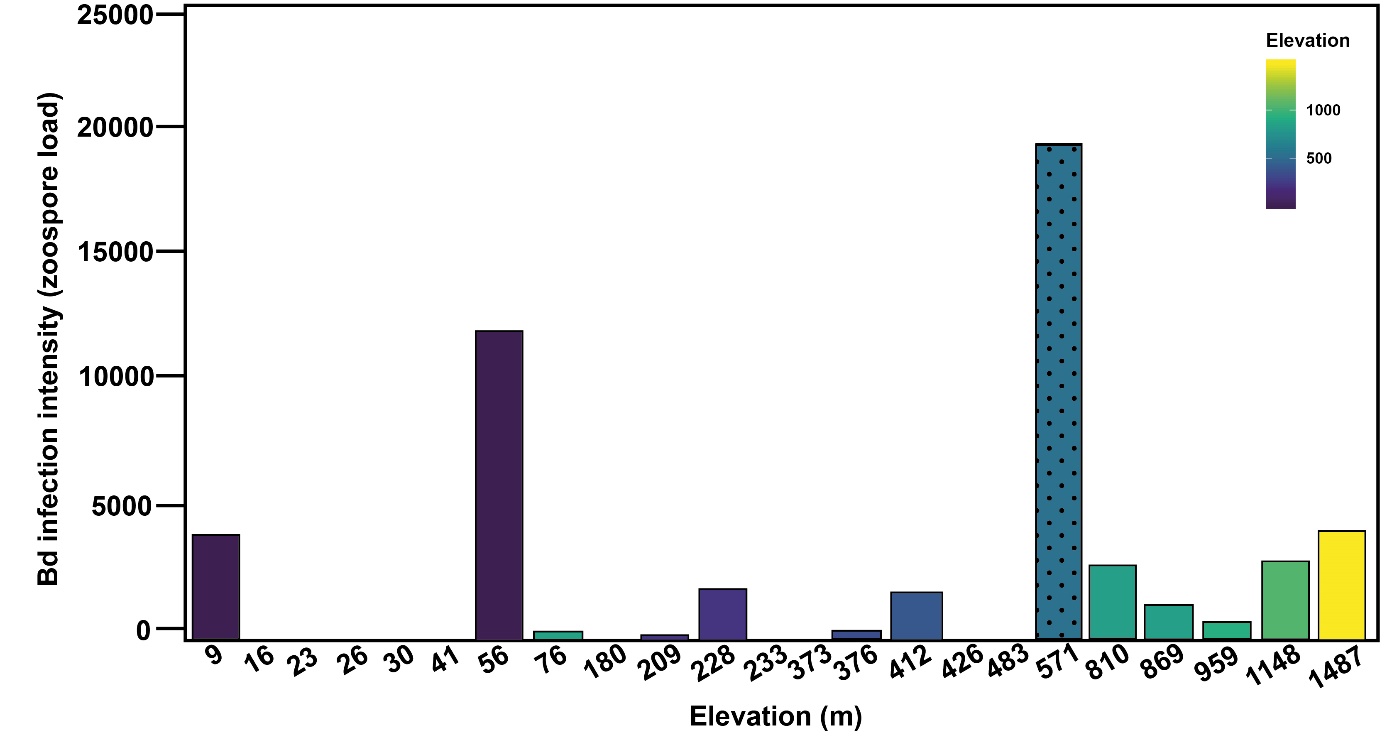
**Supplementary Figure 3.** Bd infection intensity (zoopspores per sample) of treefrogs at different elevations (m) at 23 localities across the Atlantic Forest. Dotted area represents the elevation with highest Bd infection intensity in treefrogs. Treefrogs of ten localities revealed no Bd infection intensity. Bd positive N = 188. 28 samples were excluded due to missing information on infection status.


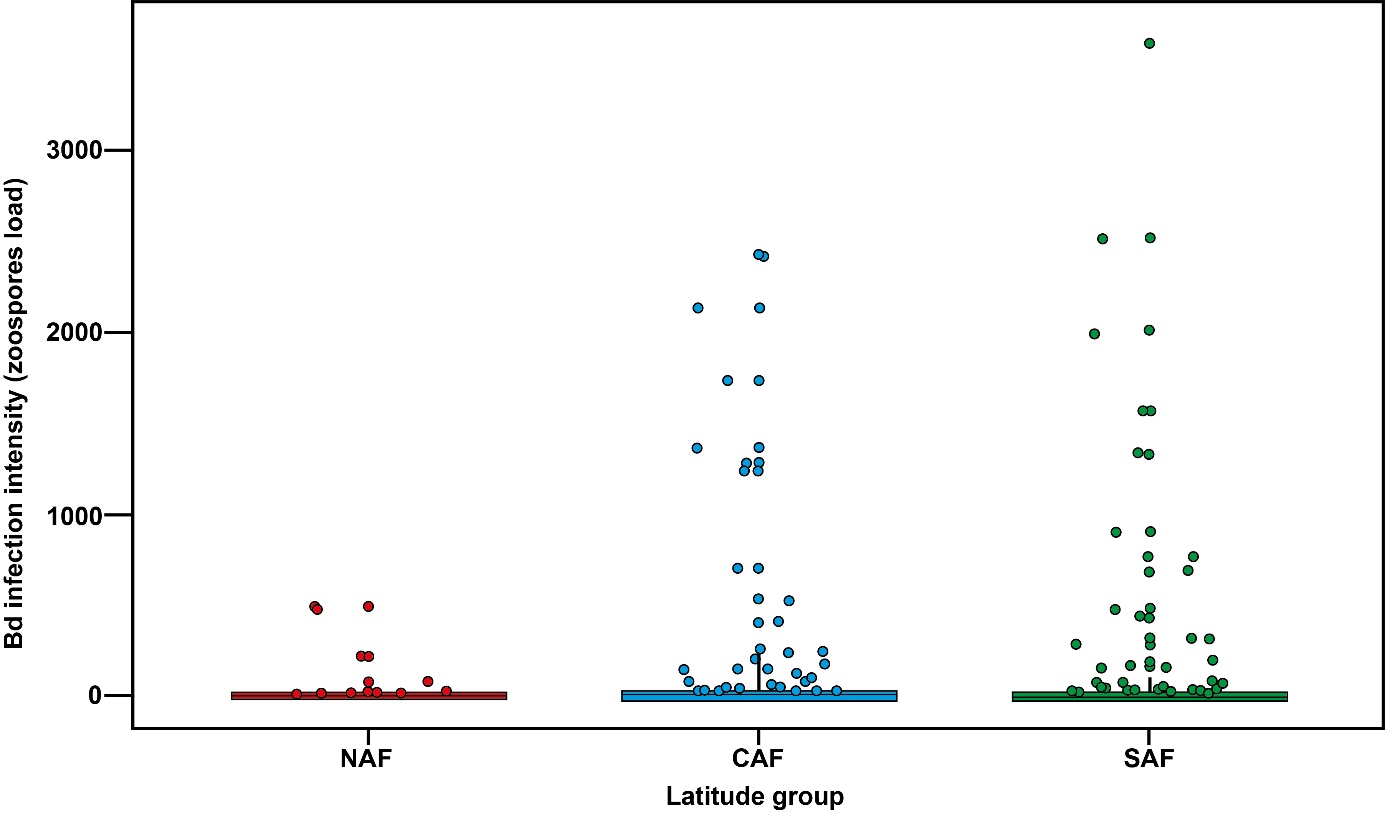
**Supplementary Figure 4.** Bd infection intensity (zoospores per sample) of treefrogs in different latitudinal groups in the Atlantic Forest. Treefrogs were infected by Bd in all latitudinal groups. NAF = North Atlantic Forest. CAF = Central Atlantic Forest. SAF = South Atlantic Forest. Bd negative N = 603. Bd positive n = 188. 28 samples were included due to missing information on infection status


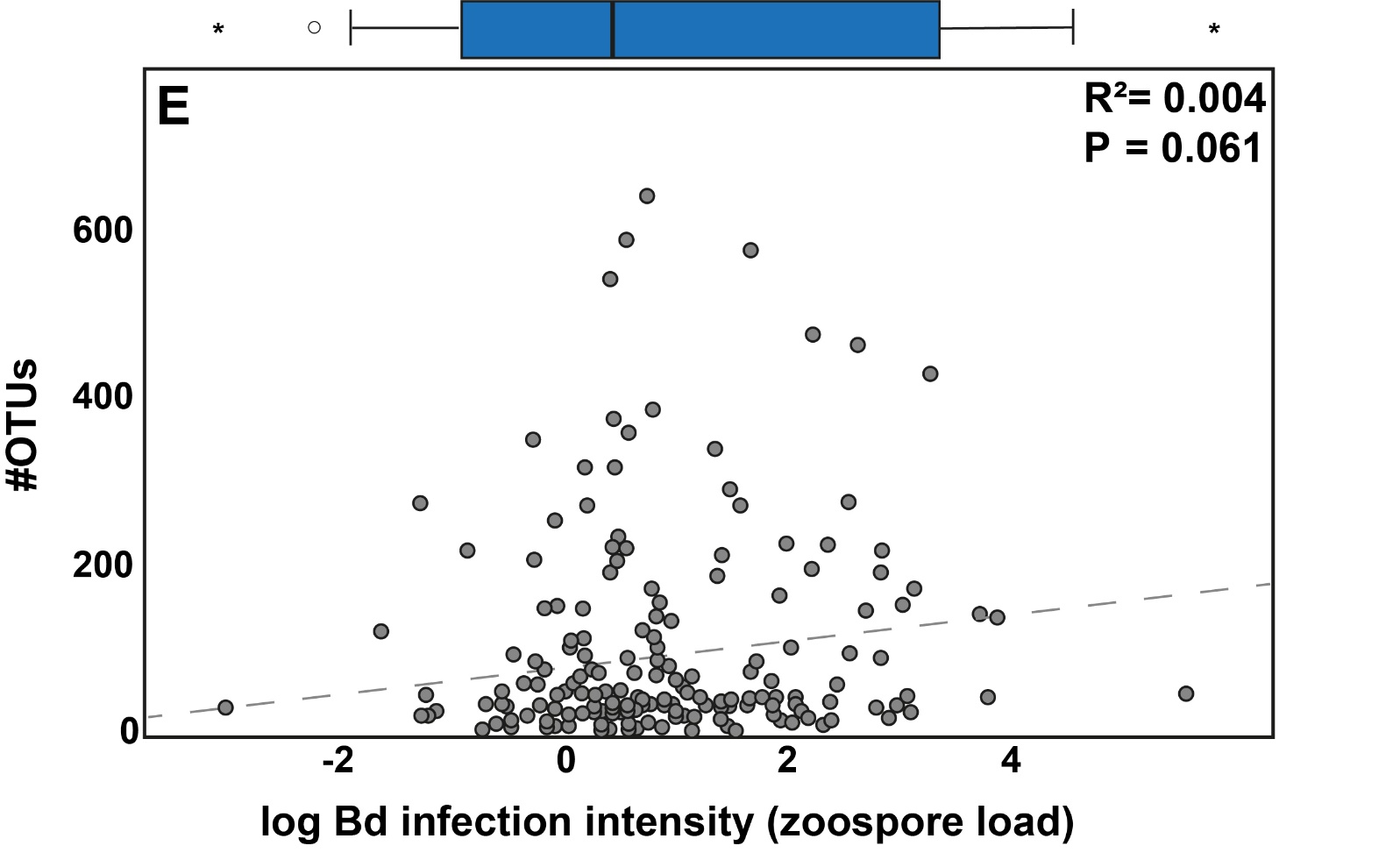


**Supplementary Figure 5.** Log-transformed Bd infection intensity (zoospores per sample) is not correlated with species richness (i.e. #OTUs) of skin microbiomes in treefrogs in the Brazilian Atlantic Forest. Zeros (N = 603 for Bd negative and N = 28 for BD NA) were removed. Regression line for significant correlation. Error bar = median. Box = 1. and 3. quartiles. Dots = outliers, minimum and maximum values. Whiskers = 1.5‐fold interquartile range. N = 188.
